# Oxygen-enabled Fe-catalyzed heteroatom α-C(sp^3^)–H functionalization with nucleophiles

**DOI:** 10.1039/d6sc05655e

**Published:** 2026-09-18

**Authors:** Xinlong Zhou, Jiayin Li, Nan Huang, Jingjing Zhao, Baokun Qiao, Hegui Gong, Pan Li

**Affiliations:** a College of Chemistry and Molecular Sciences, Henan University Kaifeng 475004 P. R. China panli@henu.edu.cn hegui_gong@henu.edu.cn

## Abstract

Here, we report an iron-catalyzed aerobic transformation that activates N/O/S-α-C(sp^3^)–H bonds under blue light irradiation, forming hemiacetal-type analogues, amenable for trapping with diverse commercially available N-, O-, S-, and C-nucleophiles. The method achieves azolation, amination, acetalization, hydroxylation, thiolation, alkylation, and arylations across ethers, thioethers and pyrrolidines with high chemoselectivity and regioselectivity. Mechanistic studies indicate that this system synergizes iron-catalyzed photoinduced LMCT, followed by chlorine radical-initiated HAT and subsequent O_2_ trapping of the resulting alkyl radicals to afford key hemiacetal-type intermediates. Significantly, FeCl_3_ serves a triple catalytic role by generating chlorine radicals, functioning as a redox shuttle and acting as a Lewis acid activator.

## Abstract 2

Here, we report an iron-catalyzed aerobic transformation that activates N/O/S-α-C(sp^3^)–H bonds under blue light irradiation, forming hemiacetal-like intermediates, amenable for trapping with diverse commercially available nucleophiles.

## Graphical abstract

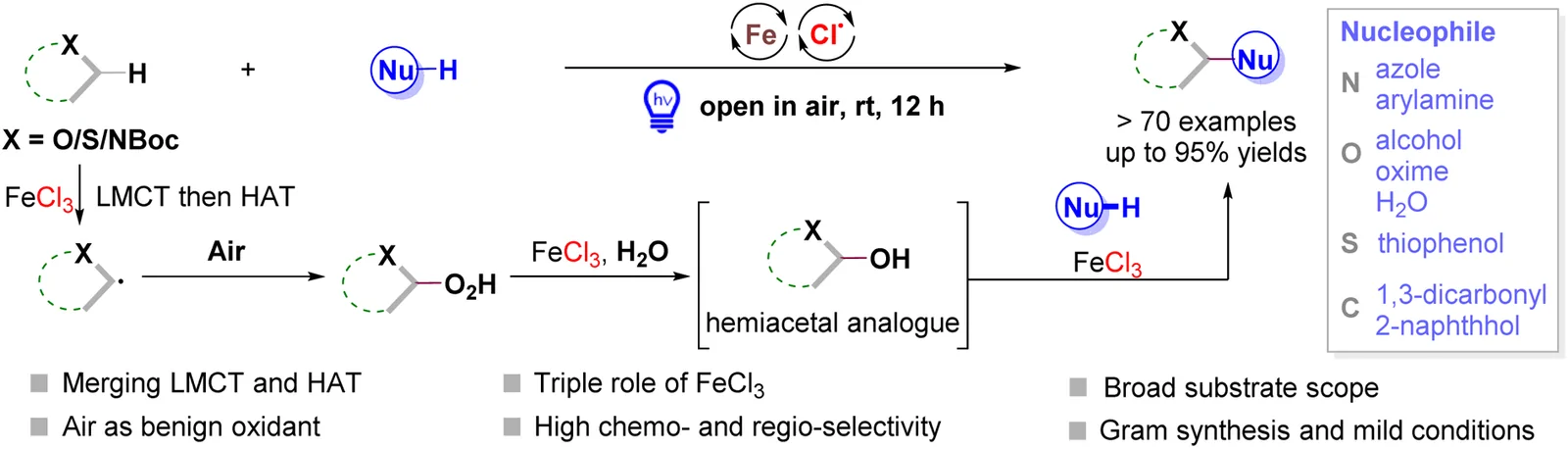

## Introduction

**Scheme 1:**
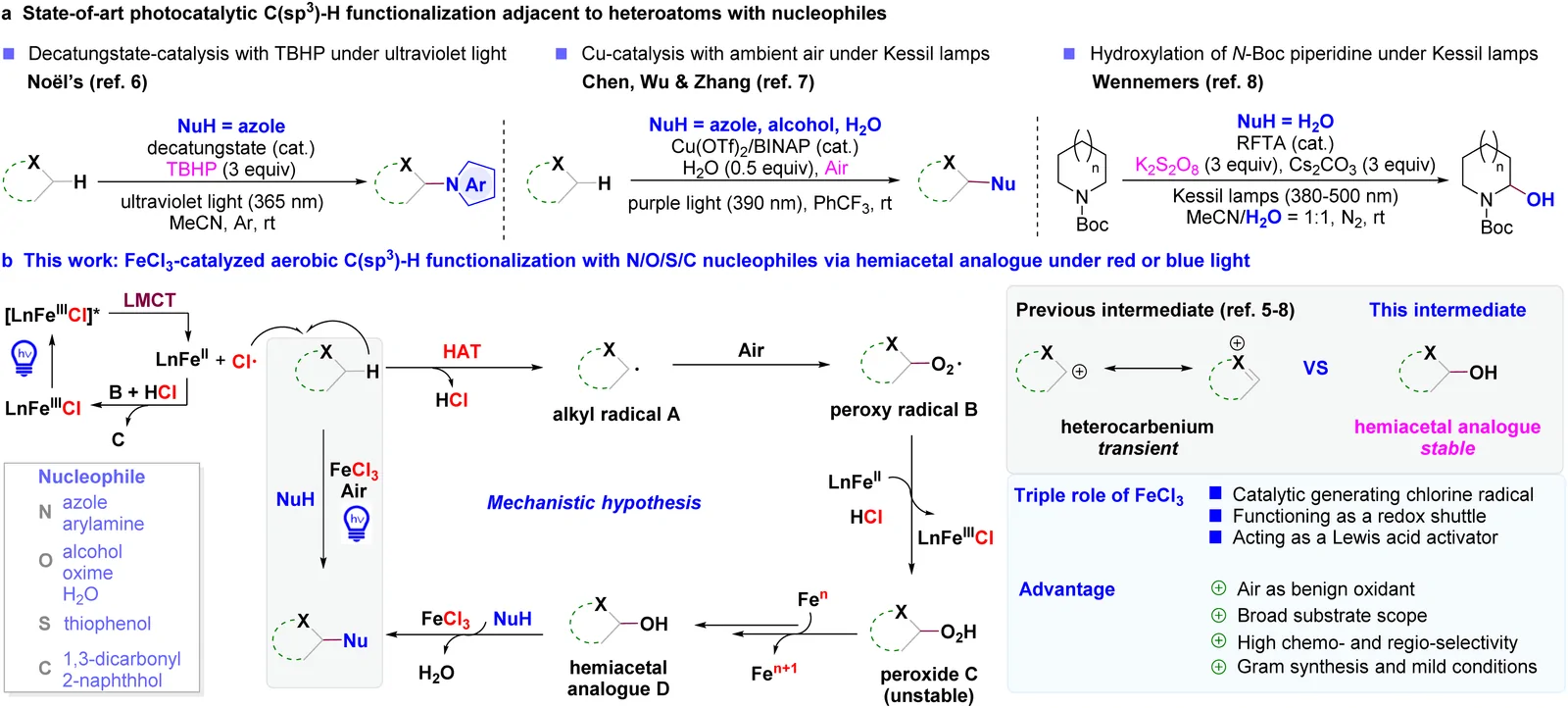
Photocatalytic C(sp^3^)–H functionalization with nucleophiles.

The direct functionalization of ubiquitous aliphatic C(sp^3^)–H bonds is a powerful yet formidable strategy for building complex molecular architectures, challenged by the inherent inertness of these bonds—namely their high bond dissociation energy (BDE), lack of polarity, and similar reaction sites.^1^ This context has incited the development of numerous elegant approaches building upon hydrogen atom transfer (HAT)^2^ of C(sp^3^)–H bonds^3^ followed by oxidation to carbocations and trapping with nucleophiles.^4^ However, these methods often require stoichiometric amounts of harsh chemical oxidants, or suffer from narrow substrate scope.^5^ Representative of this approach are several pioneering photocatalytic systems, which facilitate heteroatom α-C(sp^3^)-H bond/nucleophile coupling (Scheme 1a). These include decatungstate-catalyzed C(sp^3^)–H azolation mediated by *tert*-butyl hydroperoxide (TBHP) and ultraviolet light,^6^ Cu-catalyzed C(sp^3^)–H azolation, acetalization, and hydroxylation under ambient air and purple light,^7^ and α-hydroxylation of *N*-Boc-piperidine with water under K_2_S_2_O_8_ and Kessil lamps.^8^ Despite these,^9^ a fundamental limitation persists: the generated heterocarbenium intermediates are too transient and reactive for broad interception, restricting the scope of nucleophiles, particularly for readily oxidized amines and thiophenols. A general solution demands an operationally simple and cost-effective platform that stabilizes these species under mild conditions to accommodate a diverse nucleophile scope.

Photoinduced ligand-to-metal charge transfer (LMCT)^10^ in earth-abundant metals offers a sustainable strategy for generating reactive radicals from inert C(sp^3^)–H bonds.^11^ Inspired by advances in LMCT-driven iron photocatalysis,^12^ we envisioned a unified photoinduced FeCl_3_/air platform that could: (i) achieve site-selective hydrogen atom abstraction at to O, N, or S heteroatoms; (ii) utilize O_2_ to convert the resulting alkyl radicals into electrophilic peroxides that evolve into hemiacetal analogues; and (iii) leverage FeCl_3_ to serve dually as a Lewis acid and redox mediator, enabling the capture of a broad range of N/O/S/C nucleophiles (Scheme 1b). This design promises exceptionally mild oxidative conditions that stabilize the key hemiacetal analogues and ensure broad nucleophile compatibility, thereby overcoming limitations of previous methods. To the best of our knowledge, photoinduced C(sp^3^)–H bond/nucleophile coupling building upon Fe and O_2_ conditions have not been implemented.^13^ Building upon our longstanding interest in photochemistry and heteroatom α-C(sp^3^)–H functionalization,^14^ we report a sequential strategy featuring Fe-catalyzed aerobic oxidative hydroxylation of C(sp^3^)–H bonds, which enables subsequent nucleophilic functionalization. Its key innovations include the use of O_2_ as a terminal oxidant, the multifaceted role of FeCl_3_ acting as a triple catalyst, and exceptional compatibility with diverse N-, O-, S-, and C-nucleophiles.

## Results and discussion

We began our investigations by examining the coupling reaction between tetrahydrofuran (1–1) and benzotriazole (BT, 2–1), with selected results summarized in Table 1. After extensive experimentation, we successfully obtained the highly regioselective *N*^1^-product 3 in 90% yield and *N*^2^-product 3’ in 5% yield by employing 20 mol% FeCl_3_ open in air under red LED irradiation for 12 hours (Table 1, entry 1).^15^ Similarly, the reaction proceeded successfully when green or blue LEDs were used in place of red LEDs. Halogenated iron salts, such as FeCl_2_ and FeBr_2_, also facilitated the reaction smoothly, leading to the expected product 3 in 70% and 55% yield, respectively (Table 1, entries 2 and 3). By contrast, non-halogenated iron salts, such as Fe(acac)_3_ and FeSO_4_, were inactive, suggesting that the presence of a halide ligand is essential for this transformation (Table 1, entries 4 and 5). Further screening of other chlorinated metal salts^11^ revealed that CuCl_2_, CoCl_2_, and NiCl_2_ produced the desired product 3 in 60%, 55%, and 25% yield, respectively (Table 1, entries 6−8). Screening various solvents using 5 equivalents of tetrahydrofuran (THF) led to the desired product 3 in yields of 90%, 74%, and 63% when MeCN, toluene, and dichloromethane (DCM) were used, respectively (Table 1, entries 9−11). When the FeCl_3_ concentration was reduced to 10 mol%, the yield of product 3 dropped to 75% (Table 1, entry 12). Control experiments revealed that the desired product 3 was obtained in 85% yield under an O_2_ atmosphere. In contrast, the yield drastically dropped to 12% under an argon atmosphere. Furthermore, the reaction did not proceed in the absence of either FeCl_3_ or visible light. Nonetheless, a 25% yield of 3 was obtained at 65 °C in the dark, implying an alternative thermal reaction pathway.

**Table 1:** Screening of Reaction Conditions^a^

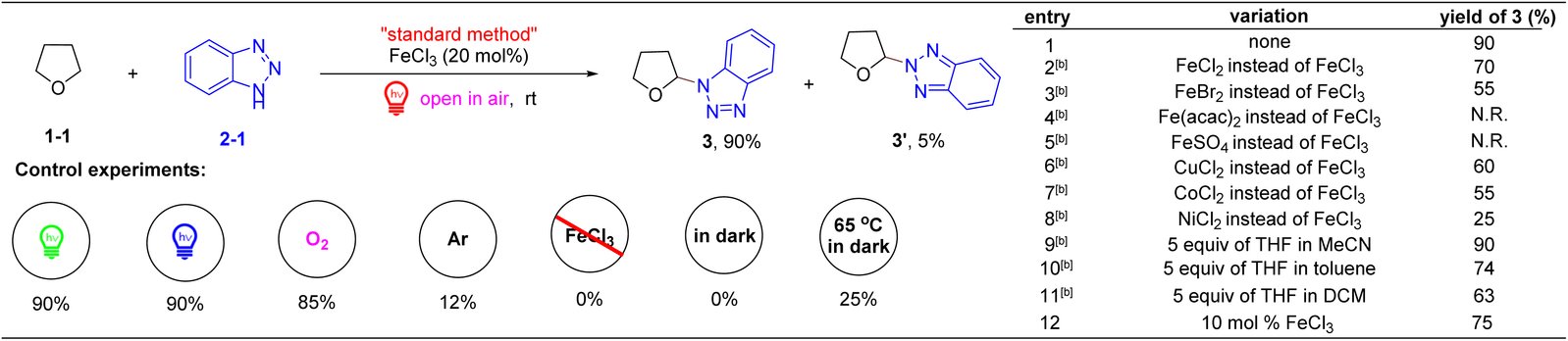

a Reaction conditions: BT (0.4 mmol), FeCl_3_ (20 mol%), THF (4 mL), red LEDs (*λ*_max_ = 642 nm, Δ*λ* = 23 nm), open in air, rt, 12 h. Yields of isolated products. N.R. indicates “no reaction”.

b Using blue light instead of red light. Green LEDs (*λ*_max_ = 520 nm, Δ*λ* = 33 nm); Blue LEDs (*λ*_max_ = 450 nm, Δ*λ* = 25 nm).

**Scheme 2:**
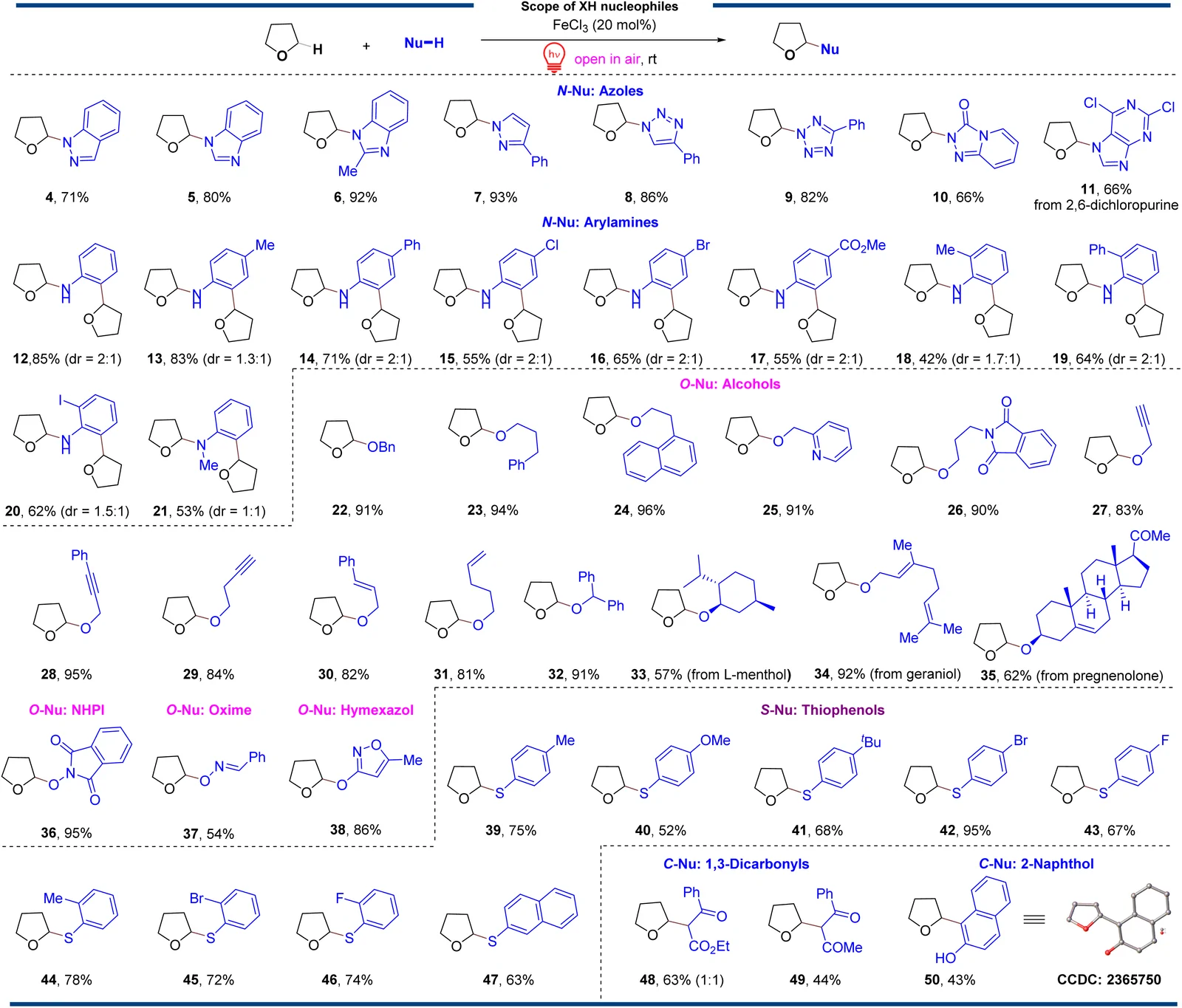
Scope of XH nucleophiles. Conditions A: nucleophiles (0.4 mmol), FeCl_3_ (20 mol%), THF (4 mL), red LEDs (*λ*_max_ = 642 nm, Δ*λ* = 23 nm), open in air, rt, 12 h. Yields of isolated products.

With the optimal conditions established, we evaluated the reaction scope using a diverse range of N-, O-, S-, and C-nucleophiles in THF (Scheme 2). Various N-heterocycles including indazole, benzimidazole, pyrazole, triazole, tetrazole, pyridintriazolone, and 2,6-dichloropurine underwent efficient coupling, giving the desired *N*-alkylated products 4–11 in yields ranging from 93% to 66%. Notably, aryl primary amines bearing electron-donating (Me, Ph) or electron-withdrawing (Cl, Br, I, CO_2_Me) groups at the *para*- or *ortho*-positions, as well as *N*-methylaniline, successfully participated in the transformation. This reactivity afforded the corresponding dual-THF products 12–21 in 42–85% yield, demonstrating an unprecedented extension to amine nucleophiles. The scope was subsequently extended to O-nucleophiles. Primary and secondary alcohols, encompassing benzyl, alkyl, alkynyl, vinyl, and heterocyclic groups, provided the desired coupling products 22–32 in 81–96% yield. This versatility was further highlighted by the successful coupling of complex natural products, including L-menthol, geraniol and pregnenolone, which delivered products 33–35 in good to excellent yield. Other O-nucleophiles, such as *N*-hydroxyphthalimide (NHPI) and oximes, also proved compatible, furnishing products 36 and 37 in 95% and 54% yield, respectively. Hymexazol underwent exclusive O-alkylation to yield product 38 in 86% yield, a selectivity we attribute to favorable amide tautomerism over *N*-alkylation. We were pleased to find that common S-nucleophiles were also suitable substrates.^16^ Thiophenols with electron-donating (Me, OMe, *t*Bu) or electron-withdrawing (Br, F) substituents at the *para*- or *ortho*-positions, along with naphthalene-2-thiol, reacted smoothly to afford the desired products 39–47 in 52–95% yield. Finally, we explored C-nucleophiles. As ambident nucleophiles, 1,3-dicarbonyl compounds yielded exclusively the C-alkylation products 48 and 49 in 63% and 44% yield, respectively. Furthermore, naphthalen-2-ol exhibited exclusive C-alkylation selectivity, affording product 50 in 43% yield. The structure of 50 was unambiguously confirmed by X-ray crystallographic analysis (CCDC 2365750). Notably, some nucleophiles, such as 4-methoxybenzenethiol, oxime, and β-dicarbonyl compound, are prone to decomposition under the FeCl_3_/air/light conditions, leading to only moderate yields (see SI for details on their compatibility and reactivity).

**Scheme 3:**
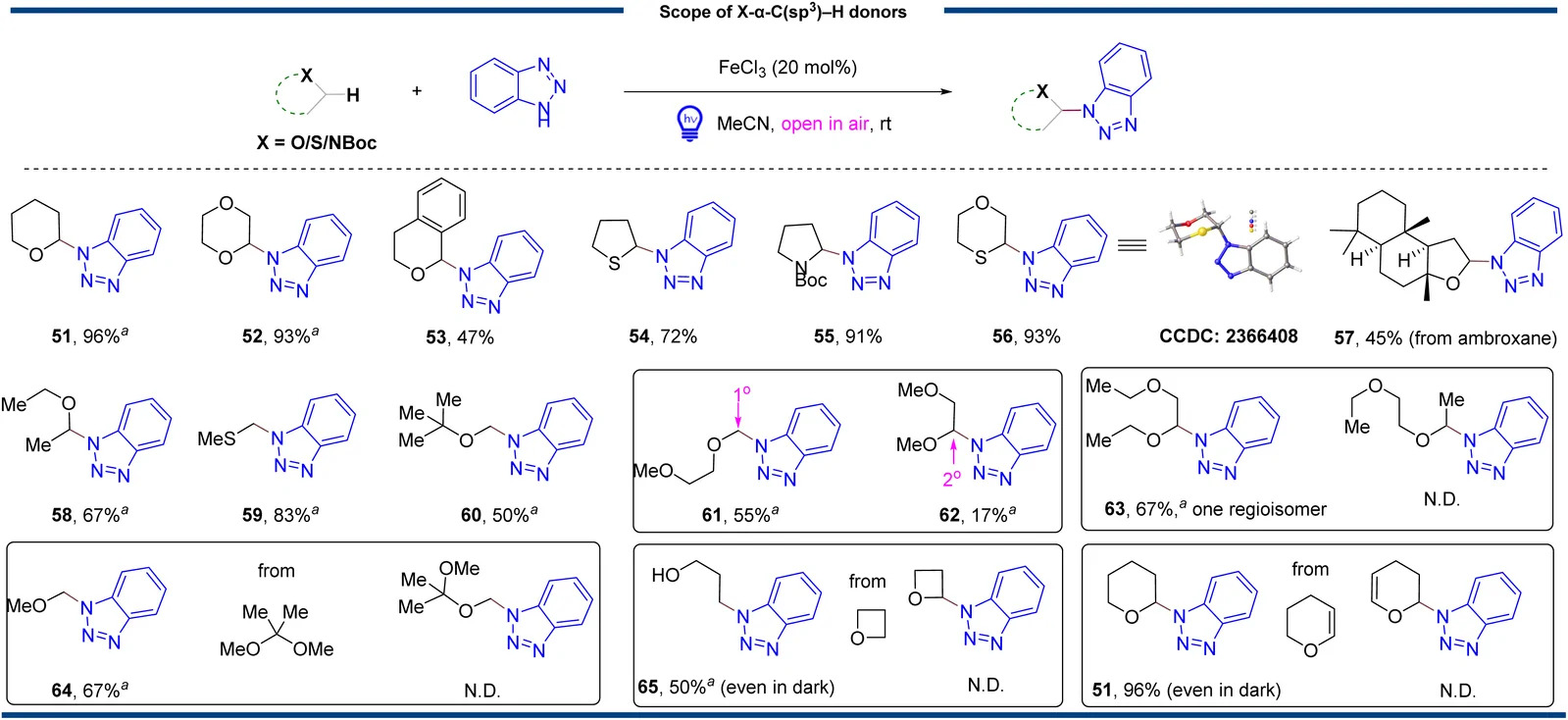
Scope of X-α-C(sp^3^)–H donors. Conditions B: BT (0.4 mmol), sp^3^ C–H precursor (2 mmol), FeCl_3_ (20 mol%), MeCN (4 mL), blue LEDs (*λ*_max_ = 450 nm, Δ*λ* = 25 nm), open in air, rt, 12 h. Yields of isolated products. N.D. indicates “not detected”. ^*a*^sp^3^ C–H donor as the solvent.

Encouraged by these results, we next explored the scope with various other C(sp^3^)–H donors in reactions with BT (Scheme 3). Unfortunately, these substrates were largely unreactive under the standard red-light conditions. To understand this lack of reactivity, we turned to UV/Vis absorption spectroscopy. The spectra revealed that FeCl_3_ in THF has absorption in the red-light region (Fig. S5). While tetrahydro-2*H*-pyran (THP) and 1,4-dioxane showed weak absorption in the blue light region, neither exhibited significant absorption under red light, consistent with the observed reactivity. Accordingly, we investigated other C(sp^3^)–H donors under blue LED irradiation. As anticipated, efficient α-C–H functionalization was achieved for cyclic ethers, including tetrahydropyran and 1,4-dioxane, furnishing the desired products 51 and 52 in excellent yields. Isochromane also participated, albeit with lower efficiency, to deliver the highly regioselective product 53 in 47% yield.^17^ The scope successfully extended beyond oxygen. α-C–H functionalization proximal to sulfur and nitrogen was demonstrated using tetrahydrothiophene and *N*-Boc pyrrolidine, which provided products 54 and 55 in 72% and 91% yield, respectively. Notably, 1,4-oxathiane exhibited exclusive selectivity for α-to-S C–H functionalization, yielding product 56 in 93% yield instead of the isomeric α-to-O product. The structure of 56 was unambiguously confirmed by X-ray crystallographic analysis (CCDC 2366408). This methodology proved applicable to complex molecular architectures, as evidenced by the successful coupling of the terpenoid ambroxide to give product 57 in 45% yield. Acyclic substrates were also viable partners. Symmetrical ethers and thioethers, such as diethyl ether and dimethyl sulfide, underwent smooth reaction to provide products 58 and 59 in 67% and 83% yield, respectively. The unsymmetrical ether, methyl *tert*-butyl ether (MTBE), furnished product 60 in 50% yield. 1,2-Dimethoxyethane (DME) yielded a mixture of two regioselective products (61 and 62) in 55% and 17% yield, respectively, whereas diethoxyethane provided only the single regioisomer 63 in 67% yield. Unexpectedly, 2,2-dimethoxypropane also participated, affording product 64 in 67% yield. Intriguingly, the reaction pathway diverged for strained and activated systems. Strained oxetane underwent rapid ring-opening to yield product 65 in 50% yield, a transformation that proceeded even in the dark without light irradiation. This reaction is consistent with well-established Lewis acid-promoted ring-opening of strained ethers and does not involve a radical pathway. Similarly, the activated vinyl ether 3,4-dihydro-2*H*-pyran rapidly underwent nucleophilic addition at the α-position, yielding product 51 in 96% yield under dark conditions. This transformation represents a classical Lewis acid-promoted activation of an enol ether, which rapidly generates the THP-type protected product.

**Scheme 4:**
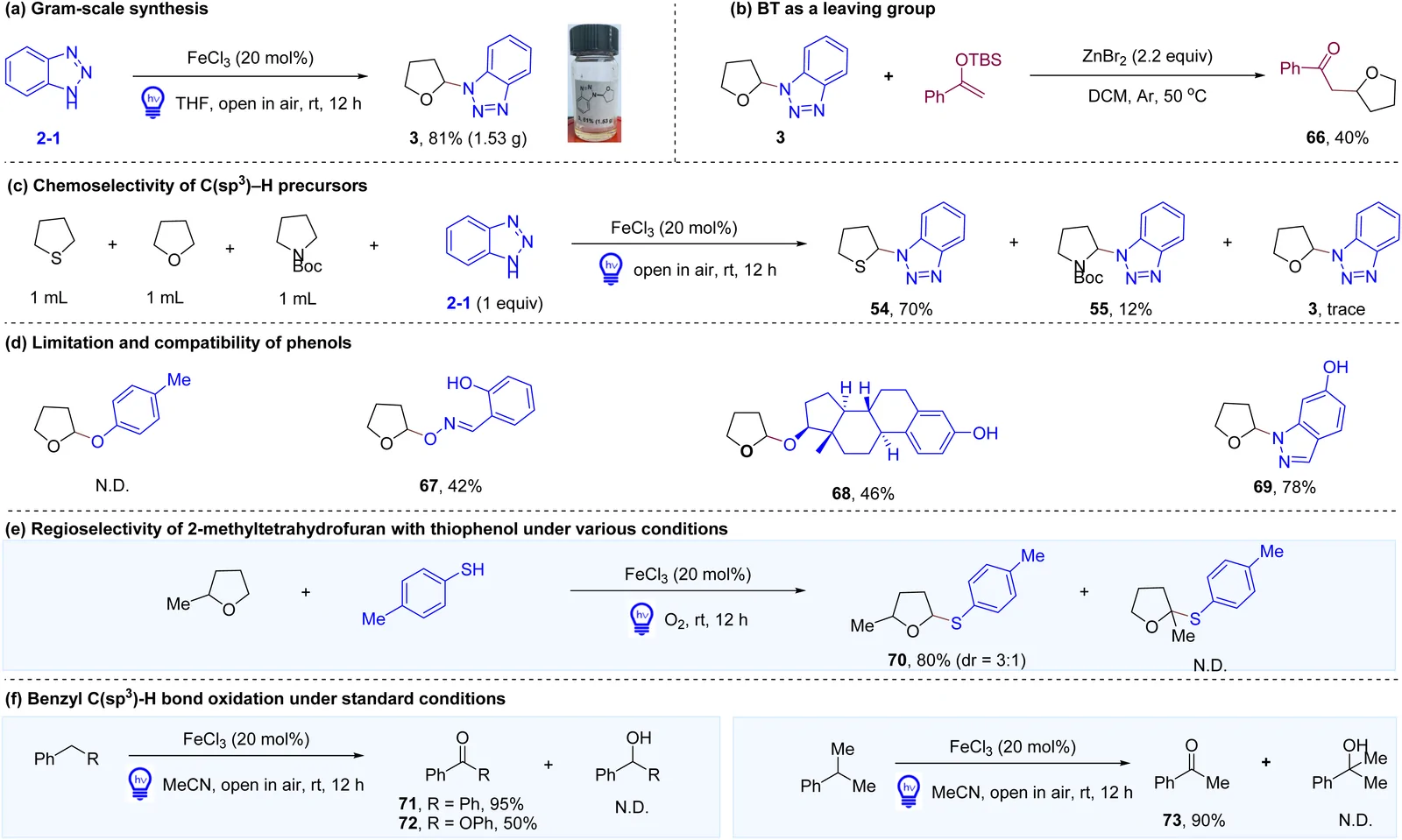
Synthetic advantage.

To demonstrate the synthetic utility of this methodology, we conducted a gram-scale reaction (Scheme 4a). Pleasingly, scaling the benchmark reaction to 10 mmol provided the desired product 3 (1.53 g) in 81% yield, confirming its practicality for larger-scale synthesis. The bench-stable product 3 showed no decomposition at elevated temperature; however, in the presence of FeCl_3_ at room temperature, 10% decomposition was observed until equilibrium was reached. Capitalizing on the ability of benzotriazole to serve as a leaving group, we found that product 3 could undergo further diversification. Treatment with a silyl enol ether using ZnBr_2_ yielded functionalized product 66 in 40% yield (Scheme 4b). A chemoselectivity study using an equimolar mixture of C(sp^3^)–H substrates revealed a distinct reactivity order: tetrahydrothiophene, *N*-Boc pyrrolidine, and tetrahydrofuran provided products 54, 55 and 3 in 70%, 12%, and trace yields, respectively (Scheme 4c). While phenols themselves were inactive, nucleophiles containing phenolic hydroxyl groups were well-tolerated, reacting smoothly to afford products 67–69 in moderate to good yields (Scheme 4d). This underscores the excellent functional group compatibility of this system. We further evaluated the regioselectivity of 2-methyltetrahydrofuran with thiophenol afforded product 70 in 80% yield with a diastereomeric ratio of 3 : 1, and no additional regioisomer was detected (Scheme 4e). Finally, we explored benzylic C(sp^3^)–H substrates (Scheme 4f). Interestingly, secondary benzylic C–H bonds underwent direct oxidation to ketones 71 and 72 in 95% and 50% yield, respectively, with no detectable alcohol intermediates.^18^ Isopropylbenzene, a substrate with tertiary benzylic C–H bonds, also yielded acetophenone 73 in 90% yield instead of the expected 2-phenylpropan-2-ol, suggesting an oxidation pathway that proceeds beyond simple hydroxylation.^19^

**Scheme 5:**
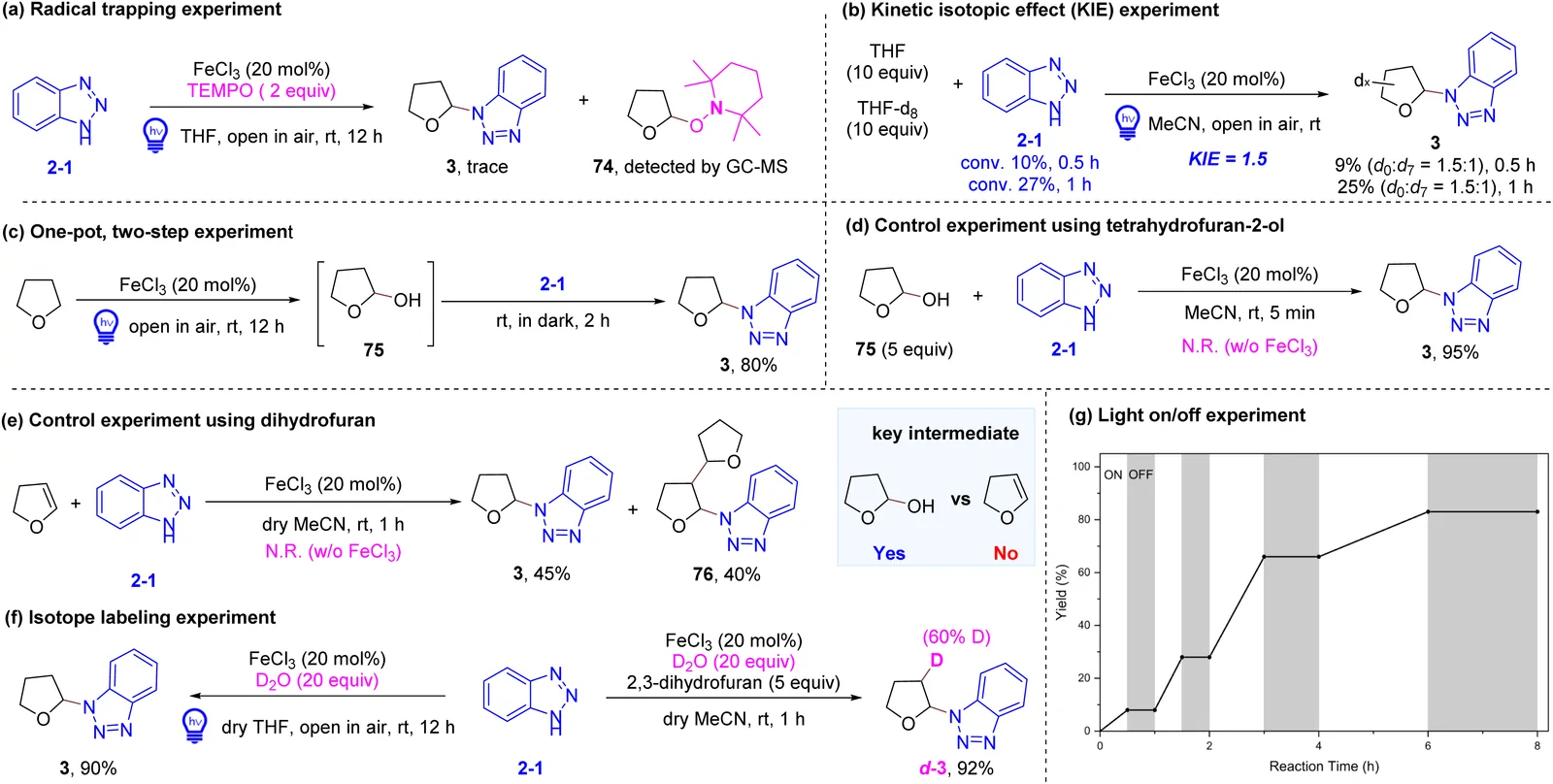
Experimental studies.

To elucidate the reaction mechanism, a series of control experiments were performed (Scheme 5). The model reaction was completely suppressed in the presence of the radical scavenger TEMPO ((2,2,6,6-tetramethylpiperidin-1-yl)oxyl). Furthermore, the TEMPO-alkyl adduct 74 was detected by GC-MS, providing direct evidence for the involvement of an alkyl radical intermediate (Scheme 5a). A kinetic isotope effect (KIE) study was conducted, in which an intermolecular competition experiment using THF/THF-*d*_8_ under standard conditions gave a KIE value (*k*_H_/*k*_D_) of approximately 1.5 (Scheme 5b). A one-pot, two-step experiment provided insight into the sequence of events. Gram-scale conversion of tetrahydrofuran into 2-hydroxytetrahydrofuran 75 was achieved (see the SI for details). Tetrahydrofuran-2-ol 75 exists in equilibrium with 4-hydroxybutanal, which accounts for less than 16% of the mixture, whereas the ring-opened form of tetrahydro-2*H*-pyran-2-ol accounts for less than 1%. Subsequent addition of BT afforded the final product 3 in 80% yield even in dark (Scheme 5c). Commercially available 75 reacted rapidly with BT in the presence of FeCl_3_ to yield product 3 in 95% yield. This reaction did not proceed in the absence of FeCl_3_, underscoring the essential role of the Lewis acid (Scheme 5d). In a parallel experiment, dihydrofuran reacted with BT to furnish product 3 in 45% yield alongside another product 76 in 40% yield (Scheme 5e). This reaction also required FeCl_3_. Isotopic labeling experiments were conducted to further interrogate the pathway. The addition of 20 equivalents of D_2_O to the standard model reaction yielded the non-deuterated product 3 in 90% yield (Scheme 5f, left). In contrast, when the same amount of D_2_O was added to the reaction of dihydrofuran with BT, the deuterated product d-3 was obtained in 92% yield with 60% deuterium incorporation (Scheme 5f, right). Collectively, these results rule out dihydrofuran as a key intermediate in the standard catalytic cycle. Instead, they provide strong evidence that the reaction proceeds *via* the formation of 2-hydroxytetrahydrofuran rather than 2,3-dihydrofuran. Additionally, the apparent quantum yield of the model reaction was determined to be 0.01 (see SI for details), indicating that continuous irradiation is required for efficient product formation, consistent with the ON/OFF light experiment (Scheme 5g). Collectively, these results suggest that a radical-chain pathway is unlikely to be the predominant mechanism.

**Scheme 6:**
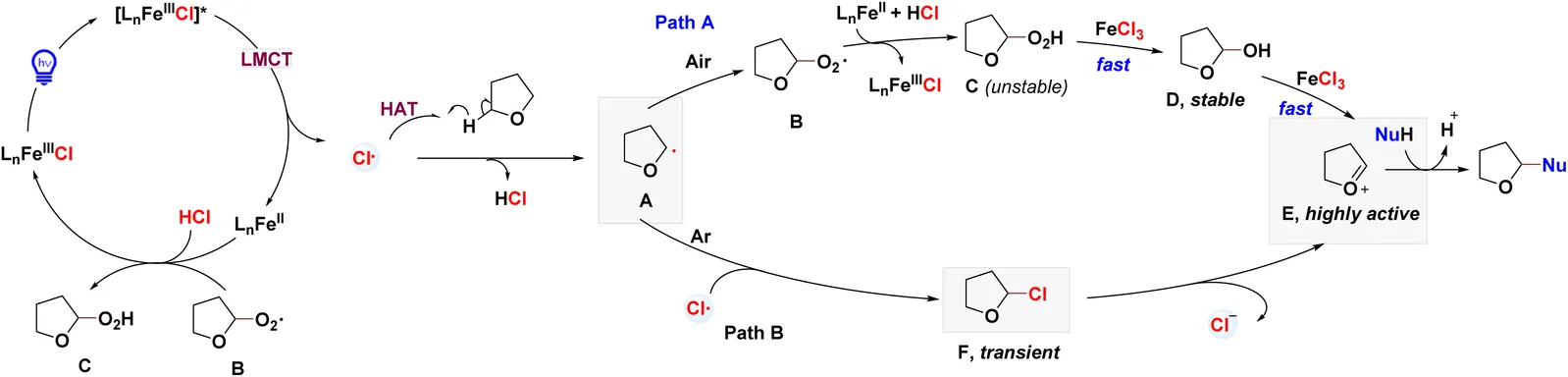
Plausible mechanism.

Based on our mechanistic studies, we propose a plausible reaction mechanism for the transformation in THF (Scheme 6). The catalytic cycle is initiated by photoexcitation of LnFe^III^Cl complex, generating the excited state [LnFe^III^Cl]*, which undergoes an intramolecular LMCT process to produce a reduced L_n_Fe^II^ species and a highly reactive chlorine radical (˙Cl). The ˙Cl species rapidly abstracts an α-hydrogen atom from THF *via* a HAT process, generating the corresponding alkyl radical A and HCl. Subsequently, radical A reacts with O_2_ to afford the peroxyl radical intermediate B. This species is further converted in the presence of L_n_Fe^II^ and HCl into the corresponding peroxide C, while regenerating the active LnFe^III^Cl catalyst to complete the catalytic cycle. In the presence of the FeCl_3_, the unstable peroxide C undergoes homolytic cleavage to generate the key hemiacetal intermediate D. In the presence of FeCl_3_, D will readily convert into the oxocarbenium ion E. Alternatively, radical A may be trapped by a ˙Cl radical, forming the transient chlorinated intermediate F. This species rapidly fragments, releasing the oxocarbenium ion E and a chloride anion (Scheme 6, Path B). Finally, the oxocarbenium ion E is attacked by various nucleophile, leading to the desired functionalized product.

## Conclusions

In summary, we have developed an iron-catalyzed aerobic system of unmatched simplicity and economy that, under blue light irradiation, enables nucleophilic α-C(sp^3^)–H functionalization involving relatively stabilized hemiacetal-type analogues. This mechanistic strategy facilitates late-stage functionalization across a broad substrate scope using diverse N-, O-, S-, and C-centered nucleophiles, thereby establishing a versatile and sustainable platform for heteroatom α-C(sp^3^)–H azolation, amination, acetalization, hydroxylation, thiolation, alkylation, and arylation. The system exhibits high chemo- and regioselectivity, broad functional group tolerance, scalability, and operational simplicity. Significantly, FeCl_3_ plays a triple catalytic role by generating chlorine radicals, functioning as a redox shuttle and acting as a Lewis acid activator.

## Author contributions

X. Z., J. L., N. H., J. Z. and B. Q. performed all experiments including condition optimizations, exploring the scope and investigating the mechanism. H. G. and P. L. supervised the project. The manuscript was written by P. L. and edited by all the co-authors.

## Conflicts of interest

There are no conflicts to declare.

## Supplementary Material

SC-OLF-D6SC05655E-s001

SC-OLF-D6SC05655E-s002

## Data Availability

The data underlying this study are available in the published article and its supporting information (SI). Supplementary information is available. See DOI: https://doi.org/10.1039/d6sc05655e.
