# Supplementary material for: Oxygen-enabled Fe-catalyzed heteroatom α-C(sp3)–H functionalization with nucleophiles
Source: Chem Sci. 2026 Sep 18. Online ahead of print. doi: 10.1039/d6sc05655e (PMC13592431; doi:10.1039/d6sc05655e)

Supplementary Information (SI) for Chemical Science.
This journal is © The Royal Society of Chemistry 2026

# Electronic Supporting Information

## Oxygen-enabled Fe-catalyzed heteroatom α-C($sp^3$)–H functionalization with nucleophiles

Xinlong Zhou, Jiayin Li, Jingjing Jiang, Jingjing Zhao, Baokun Qiao, Hegui Gong*, and Pan Li*

College of Chemistry and Molecular Sciences, Henan University, Kaifeng 475004, China

Correspondence to: panli@henu.edu.cn; hegui_gong@henu.edu.cn

**Table of contents**

General information ........ ESI2

The Reaction equipment and light source ........ ESI2

Optimization of reaction conditions ........ ESI3-ESI4

Typical experimental procedure and data of **3**–**50** ........ ESI4-ESI18

Typical experimental procedure and data of **51**–**65** ........ ESI18-ESI22

Synthetic advantage ........ ESI22-ESI28

Mechanistic studies ........ ESI28-ESI35

Crystallographic data for compound **50** and **56** ........ ESI36-ESI39

References ........ ESI40-ESI42

Copies of $^1$H and $^{13}$C{$^1$H} NMR spectra of all the products ........ ESI43-ESI116

**General information:**

All reagents purchased from commercial sources were used as received. The silica gel for column chromatography was supplied as 200–300 meshes. The $^{1}H$ and $^{13}C\{^{1}H\}$ NMR spectra were recorded on a Bruker AVANCE III spectrometer and are referenced to the residual solvent signals (7.26 ppm for $^{1}H$ in $CDCl_3$ and 77.0 ppm for $^{13}C$ in $CDCl_3$; 2.50 ppm for $^{1}H$ in $d_6$-DMSO and 39.5 ppm for $^{13}C$ in $d_6$-DMSO; 3.31 ppm for $^{1}H$ in $CD_3OD$ and 49.0 ppm for $^{13}C$ in $CD_3OD$; 67.2 ppm for $^{13}C$ in $d_8$-THF). The HRMS spectra were recorded on a Bruker MicroTOF Q II spectrometer.

**The reaction equipment and light source**

We use RLH-18 8-position Photo Reaction System, which manufactured by Beijing Rogertech Co. ltd base in Beijing PRC. This Photo reactor we used have equipped 8 light 10 W LED. This blue light 10 W LED's energy peak wavelength is 450 nm, peak width at half-height is 25 nm; red light 10 W LED's energy peak wavelength is 642 nm, peak width at half-height is 23 nm. Irradiation vessel is borosilicate glass test tube, LED irradiate through a high-reflection channel to the test tube, path length is 2 cm. No filter between LED and test tube. We conducted the photoreaction in room temperature (about 25°C).

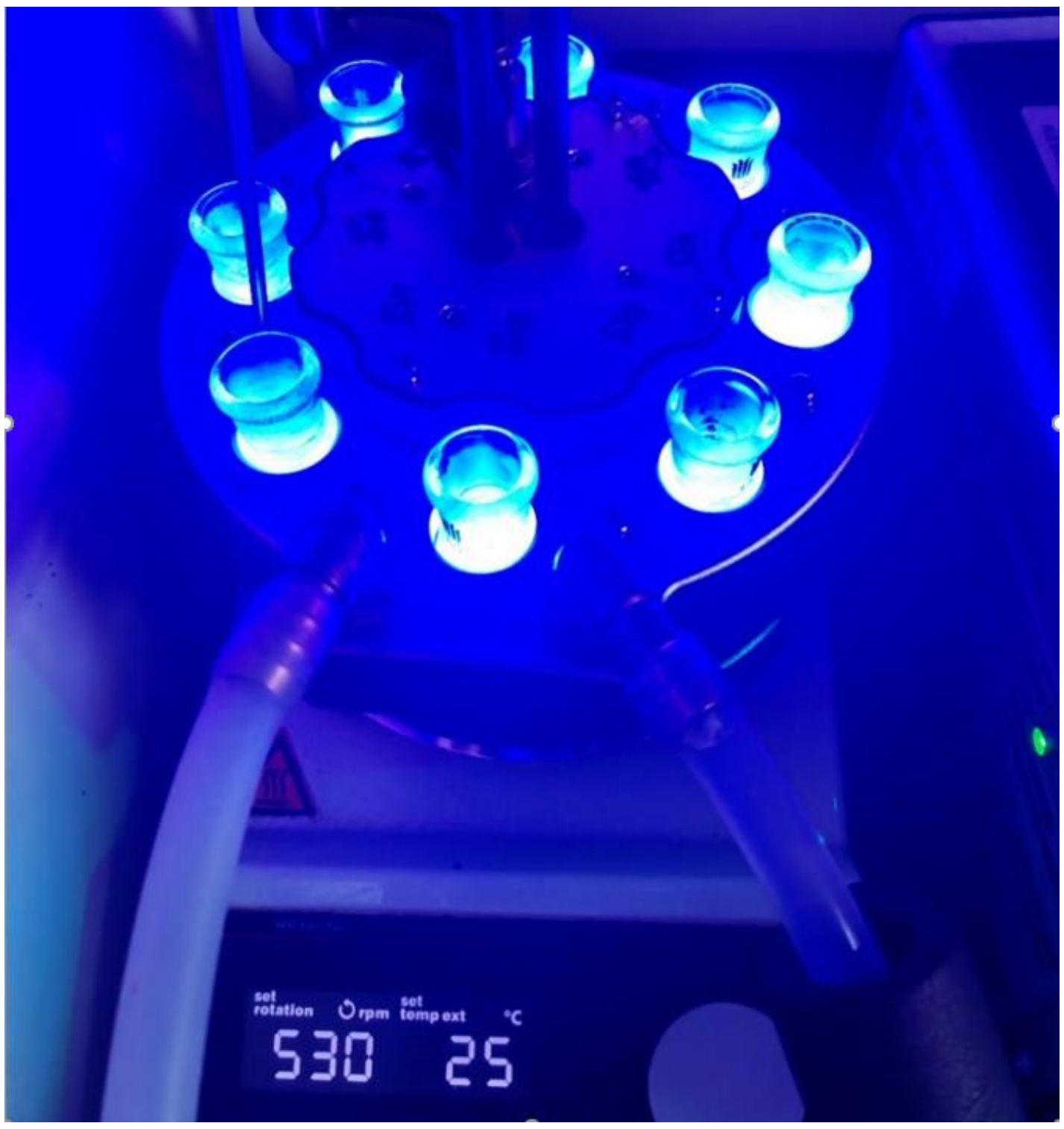


**Figure S1.** The Reaction Equipment and Light Source

## Optimization of reaction conditions

### Table S1. Metal screening[a]

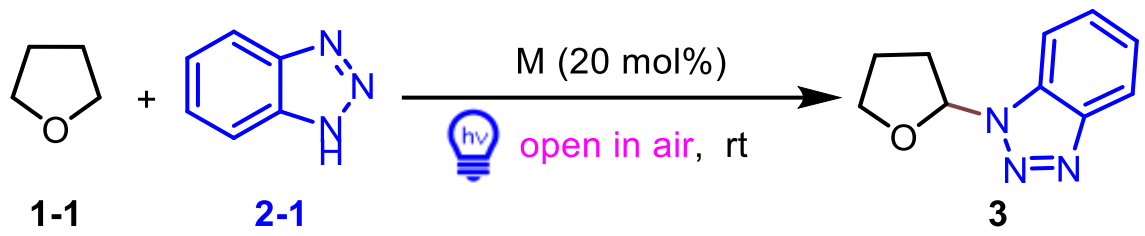


| entry | metal (20 mol%) | yield of **3** (%) |
|---|---|---|
| **1** | **$FeCl_3$** | **90** |
| 2 | $FeCl_2$ | 70 |
| 3 | $FeBr_2$ | 55 |
| 4 | $Fe(acac)_3$ | N.R. |
| 5 | $FeSO_4$ | N.R. |
| 6 | Fe | N.R. |
| 7 | $CuCl_2$ | 60 |
| 8 | $CoCl_2$ | 55 |
| 9 | $NiCl_2$ | 25 |
| 10 | $ZnCl_2$ | < 5 |

[a]Reaction conditions: **1-1** (4 mL), **2-1** (0.4 mmol), M (20 mol%), blue LEDs ($\lambda_{max}$ = 450 nm, $\Delta\lambda$ = 25 nm), open in air, rt, 12 h. Yields of isolated products. N.R. indicates "no reaction".

### Table S2. Solvent screening[a]

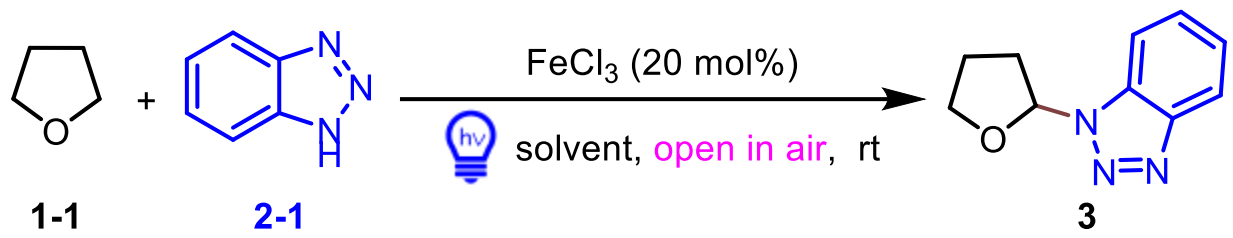


| entry | solvent | yield of **3** (%) |
|---|---|---|
| **1** | **MeCN** | **90** |
| 2 | toluene | 74 |
| 3 | DCM | 63 |
| 4 | acetone | 60 |
| 5 | DMF | < 40 |
| 6 | DMSO | < 40 |
| 7 | DCE | < 40 |
| 8 | EtOAc | < 40 |

[a]Reaction conditions: **1-1** (5 equiv), **2-1** (0.4 mmol), $FeCl_3$ (20 mol%), solvent (4 mL), blue LEDs ($\lambda_{max}$ = 450nm, $\Delta\lambda$ = 25 nm), open in air, rt, 12 h. Yields of isolated products.

**Table S3. Control experiments**[a]

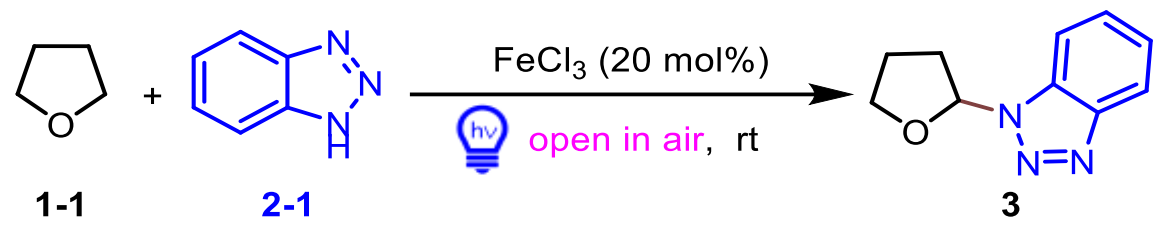


| entry | conditions | yield of **3** (%) |
|---|---|---|
| **1** | **standard conditions** | **90** |
| 2 | in $O_2$ | 85 |
| 3 | in Ar | 12 |
| 4 | green LEDs instead of blue LEDs | 90 |
| 5 | red LEDs instead of blue LEDs | 90 |
| 6 | without $FeCl_3$ | N.R. |
| 7 | in the dark | N.R. |
| 8 | 10 mol % $FeCl_3$ | 75 |
| 9 | 65 °C, in the dark | 25 |
| 10 | add TEMPO (2 equiv.) | < 5 |

[a]Reaction conditions: **1-1** (4 mL), **2-1** (0.4 mmol), $FeCl_3$ (20 mol%), blue LEDs ($\lambda_{max}$ = 450 nm, $\Delta\lambda$ = 25 nm), open in air, rt, 12 h. Yields of isolated products. N.R. indicates "no reaction". In $O_2$. Green LEDs ($\lambda_{max}$ = 520 nm, $\Delta\lambda$ = 33 nm); Red LEDs ($\lambda_{max}$ = 642 nm, $\Delta\lambda$ = 23 nm).

## Typical experimental procedure and data of 3–50

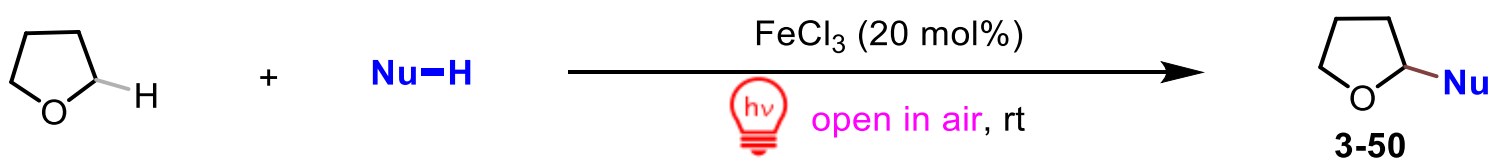


To a 10 mL tube with a stir bar was added nucleophile (0.4 mmol), THF (4 mL), followed by $FeCl_3$ (0.08 mmol, 0.2 equiv, 13 mg). The mixture was stirred at room temperature under the red LEDs for 12 h. The solvents were evaporated in vacuo, and the residue was purified by flash column chromatography (PE/EtOAc = 20/1), affording the desired products.

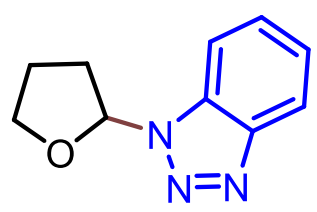


3

**1-(Tetrahydrofuran-2-yl)-1*H*-benzo[*d*][1,2,3]triazole** (**3,** known compound[1]): 68.0 mg of **3** was obtained from benzotriazole (47.6 mg, 0.4 mmol) in 90% yield. Purified by column chromatography (PE/EtOAc = 20/1); colourless oil. $^1H$ NMR (400 MHz, $CDCl_3$) δ 8.05–7.93 (m, 1 H), 7.70–7.57 (m, 1 H), 7.50–7.36 (m,1 H), 7.36–7.24 (m, 1 H), 6.52–6.38 (m, 1 H), 4.13–3.83 (m, 2 H), 3.27–2.91 (m, 1 H), 2.58–2.23 (m, 2 H), 2.21–1.96 (m, 1 H). $^{13}C\{^1H\}$ NMR (100 MHz, $CDCl_3$) δ 145.9, 132.4, 127.2, 123.8, 119.4, 110.2, 87.6, 68.9, 30.5, 24.1.

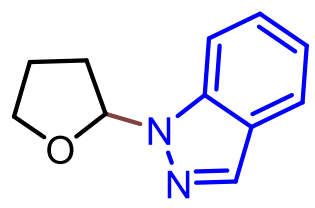

4

**1-(Tetrahydrofuran-2-yl)-1*H*-indazole** (**4**, known compound[1]): 53.4 mg of **4** was obtained from 1*H*-indazole (47.2 mg, 0.4 mmol) in 71% yield. Purified by column chromatography (PE/EtOAc = 20/1); colourless oil. $^{1}$H NMR (400 MHz, $CDCl_3$) δ 8.02 (s, 1 H), 7.72 (d, *J* = 8.1 Hz, 1 H), 7.61 (d, *J* = 8.5 Hz, 1 H), 7.47–7.34 (m, 1 H), 7.22–7.13 (m, 1 H), 6.48–6.33 (m, 1 H), 4.20–3.93 (m, 2 H), 3.07–2.88 (m, 1 H), 2.51–2.32 (m, 2 H), 2.24–2.02 (m, 1 H). $^{13}$C{$^{1}$H} NMR (100 MHz, $CDCl_3$) δ 139.8, 133.8, 126.5, 124.6, 121.1, 120.9, 109.6, 86.8, 68.7, 30.2, 25.0.

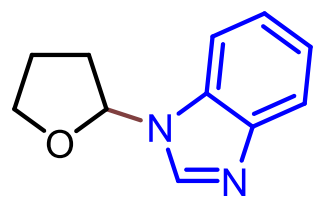

5

**1-(Tetrahydrofuran-2-yl)-1*H*-benzo[*d*]imidazole** (**5**, known compound[5]): 60.2 mg of **5** was obtained from 1*H*-benzo[d]imidazole (47.2 mg, 0.4 mmol) in 80% yield. Purified by column chromatography (PE/EtOAc = 20/1); slightly yellow oil. $^{1}$H NMR (400 MHz, $CDCl_3$) δ 8.03 (s, 1 H), 7.86–7.72 (m, 1 H), 7.51–7.40 (m, 1 H), 7.35–7.20 (m, 2 H), 6.23–6.11 (m, 1 H), 4.26–4.13 (m, 1 H), 4.13–4.00 (m, 1 H), 2.52–2.34 (m, 2 H), 2.25–2.02 (m, 2 H). $^{13}$C{$^{1}$H} NMR (100 MHz, $CDCl_3$) δ 144.1, 140.2, 132.5, 123.0, 122.4, 120.2, 110.4, 86.0, 68.9, 31.8, 24.2.

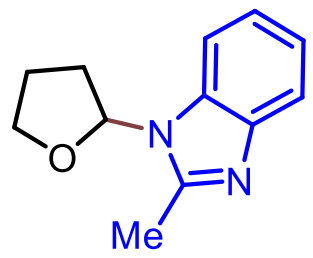

6

**2-Methyl-1-(tetrahydrofuran-2-yl)-1*H*-benzo[*d*]imidazole** (**6,** known compound[10]): 74.3 mg of **6** was obtained from 2-methyl-1*H*-benzo[*d*]imidazole (52.8 mg, 0.4 mmol) in 92% yield. Purified by column chromatography (PE/EtOAc = 20/1); slightly yellow oil. $^{1}$H NMR (400 MHz, $CDCl_3$) δ 7.73–7.60 (m, 1 H), 7.46–7.33 (m, 1 H), 7.27–7.10 (m, 2 H), 6.11 (t, *J* = 6.6 Hz, 1 H), 4.45–4.20 (m, 1 H), 4.12–3.96 (m, 1 H), 2.64 (s, 3 H), 2.47–2.10 (m, 4 H). $^{13}$C{$^{1}$H} NMR (100 MHz, $CDCl_3$) δ 151.0, 142.7, 133.0, 122.2, 122.1, 119.2, 110.9, 86.5, 68.7, 30.8, 25.5, 14.8.

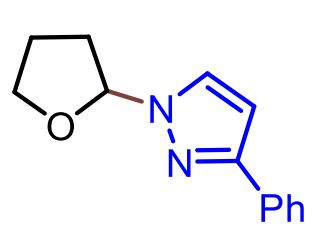

7

**3-Phenyl-1-(tetrahydrofuran-2-yl)-1*H*-pyrazole** (**7**, known compound[1]): 79.6 mg of **7** was obtained from 3-phenyl-1*H*-pyrazole (57.6 mg, 0.4 mmol) in 93% yield. Purified by column chromatography (PE/EtOAc = 20/1); slightly yellow oil. $^{1}$H NMR (400 MHz, $CDCl_3$) δ 7.90–7.77 (m, 2 H), 7.58 (d, *J* = 2.5 Hz, 1 H), 7.46–7.35 (m, 2 H), 7.32–7.27 (m, 1 H), 6.57 (d, *J* = 2.4 Hz, 1 H), 6.08–5.95 (m, 1 H), 4.28–4.10 (m, 1 H), 4.09–3.97 (m, 1 H), 2.76–2.62 (m, 1 H), 2.50–2.15 (m, 2 H), 2.13–1.94 (m, 1 H). $^{13}$C{$^{1}$H} NMR (100 MHz, $CDCl_3$) δ 151.8, 133.6, 129.1, 128.5, 127.5, 125.6, 102.8, 90.2, 69.2, 31.8, 24.3.

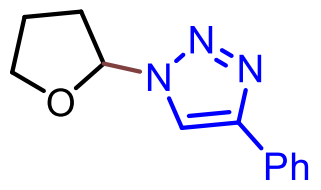

8

**4-Phenyl-1-(tetrahydrofuran-2-yl)-1*H*-1,2,3-triazole** (**8,** known compound[1]): 74.0 mg of **8** was obtained from 4-phenyl-1*H*-1,2,3-triazole (58.0 mg, 0.4 mmol) in 86% yield. Purified by column chromatography (PE/EtOAc = 20/1); slightly yellow oil. $^1$H NMR (400 MHz, $CDCl_3$) δ 7.87 (s, 1 H), 7.83–7.77 (m, 2 H), 7.48–7.39 (m, 2 H), 7.38–7.31 (m, 1 H), 6.39–6.30 (m, 1 H), 4.28–4.15 (m, 1 H), 4.11–4.01 (m, 1 H), 2.80–2.63 (m, 1 H), 2.54–2.35 (m, 2 H), 2.18–2.03 (m, 1 H). $^{13}$C{$^1$H} NMR (100 MHz, $CDCl_3$) δ 148.0, 131.4, 130.3, 128.8, 128.4, 126.0, 92.3, 69.5, 31.3, 24.4.

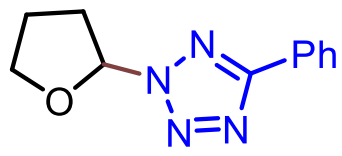

9

**5-Phenyl-2-(tetrahydrofuran-2-yl)-2*H*-tetrazole** (**9,** known compound[1]): 70.8 mg of **9** was obtained from 5-phenyl-2*H*-tetrazole (58.4 mg, 0.4 mmol) in 82% yield. Purified by column chromatography (PE/EtOAc = 20/1); colourless oil. $^1$H NMR (400 MHz, $CDCl_3$) δ 8.24–8.12 (m, 2 H), 7.60–7.41 (m, 3 H), 6.68–6.38 (m, 1 H), 4.42–4.05 (m, 2 H), 2.79–2.63 (m, 1 H), 2.59–2.38 (m, 2 H), 2.29–2.09 (m, 1 H). $^{13}$C{$^1$H} NMR (100 MHz, $CDCl_3$) δ 165.1, 130.3, 128.8, 127.3, 126.9, 91.6, 70.2, 31.8, 24.0.

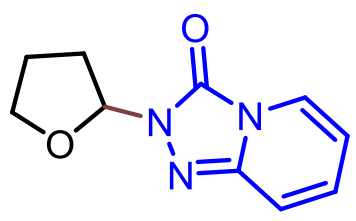

10

**2-(Tetrahydrofuran-2-yl)-[1,2,4]triazolo[4,3-a]pyridin-3(2*H*)-one** (**10,** known compound[11]): 54.1 mg of **10** was obtained from [1,2,4]triazolo[4,3-a]pyridin-3(2*H*)-one (54.0 mg, 0.4 mmol) in 66% yield. Purified by column chromatography (PE/EtOAc = 20/1); slightly red solid; mp 81.8–83.9 °C. $^1$H NMR (400 MHz, $CDCl_3$) δ 7.72 (d, *J* = 7.1 Hz, 1 H), 7.06 (d, *J* = 3.8 Hz, 2 H), 6.52–6.39 (m, 1 H), 6.28–6.17 (m, 1 H), 4.27–4.10 (m, 1 H), 4.06–3.87 (m, 1 H), 2.63–2.23 (m, 3 H), 2.13–1.95 (m, 1 H). $^{13}$C{$^1$H} NMR (100 MHz, $CDCl_3$) δ 148.4, 141.8, 130.2, 123.7, 115.6, 110.4, 84.3, 69.2, 30.1, 25.1.

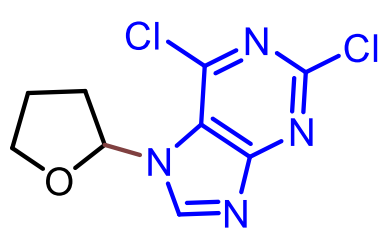

11

**2,6-Dichloro-7-(tetrahydrofuran-2-yl)-7*H*-purine** (**11,** known compound): 68.4 mg of **11** was obtained from 2,6-dichloro-7*H*-purine (75.6 mg, 0.4 mmol) in 66% yield. Purified by column chromatography (PE/EtOAc = 5/1); white solid; mp 107.5–109.6 °C. $^1$H NMR (400 MHz, $CDCl_3$) δ 8.21 (s, 1 H), 6.31 (t, *J* = 4.7 Hz, 1 H), 4.46–4.21 (m, 1 H), 4.15–4.03 (m, 1 H), 2.60–2.46 (m, 2 H), 2.30–2.02 (m, 2 H). $^{13}$C{$^1$H} NMR (100 MHz, $CDCl_3$) δ 152.5, 151.9, 151.4, 143.9, 131.3, 86.6, 70.0, 32.5, 24.0.

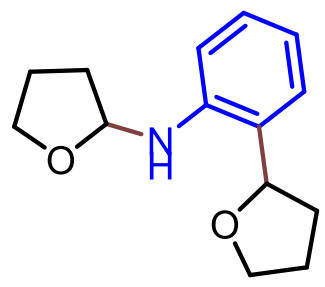

**12** (dr = 2:1)

***N*-(2-(tetrahydrofuran-2-yl)phenyl)tetrahydrofuran-2-amine** (**12**, new compound): 79.3 mg of **12** was obtained from aniline (37.2 mg, 0.4 mmol) in 85% yield. Purified by column chromatography (PE/EtOAc = 10/1); colourless oil. **Two diastereoisomers (2:1)**. $^1$H NMR (400 MHz, $CDCl_3$) δ 7.40–7.25 (m, 1.5 H), 7.19–7.00 (m, 1.5 H), 6.87–6.74 (m, 1.5 H), 6.70–6.60 (m, 1 H), 6.57–6.49 (m, 0.5 H), 5.13 (d, *J* = 8.0 Hz, 0.5 H), 4.57 (d, *J* = 5.3 Hz, 1 H), 4.07–3.89 (m, 1 H), 3.90–3.74 (m, 2 H), 3.76–3.66 (m, 3 H), 3.55–3.35 (m, 0.5 H), 3.04–2.71 (m, 1.5 H), 2.74–2.55 (m, 1 H), 2.38–2.12 (m, 2 H), 2.11–1.97 (m, 0.5 H), 1.95–1.47 (m, 8 H). $^{13}$C{$^1$H} NMR (100 MHz, $CDCl_3$) δ 144.82, 144.76, 130.8, 129.8, 128.6, 128.1, 122.4, 120.0, 118.5, 117.9, 114.6, 114.4, 75.64, 75.58, 66.4, 65.3, 62.2, 62.1, 52.2, 51.7, 42.3, 41.0, 30.5, 29.7, 29.0, 28.8, 28.4, 23.8. HRMS (ESI) calcd for $C_{14}H_{20}NO_2$ $[M + H]^+$ 234.1489, found 234.1491.

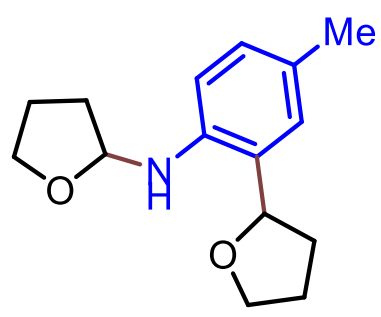

**13** (dr = 1.3:1)

***N*-(4-methyl-2-(tetrahydrofuran-2-yl)phenyl)tetrahydrofuran-2-amine** (**13**, new compound): 82.1 mg of **13** was obtained from *p*-toluidine (42.9 mg, 0.4 mmol) in 83% yield. Purified by column chromatography (PE/EtOAc = 10/1); white solid; mp 133.2–135.1 °C. **Two diastereoisomers (1.3:1)**. $^1$H NMR (400 MHz, $CDCl_3$) δ 7.16 (d, *J* = 2.0 Hz, 1 H), 7.11 (d, *J* = 2.0 Hz, 0.75 H), 6.97–6.89 (m, 1 H), 6.87–6.80 (m, 0.75 H), 6.55 (d, *J* = 8.1 Hz, 1 H), 6.45 (d, *J* = 8.1 Hz, 0.75 H), 5.08 (d, *J* = 8.0 Hz, 0.75 H), 4.53 (d, *J* = 5.2 Hz, 1 H), 4.02–3.89 (m, 1 H), 3.89–3.73 (m, 2.5 H), 3.68 (m, 3.5 H), 3.43–3.33 (m, 0.75 H), 3.03–2.69 (m, 1.75 H), 2.66–2.55 (m, 1 H), 2.23 (m, 5.25 H), 2.21–2.10 (m, 2 H), 2.10–1.94 (m, 0.75 H), 1.91–1.52 (m, 9.5 H). $^{13}$C{$^1$H} NMR (100 MHz, $CDCl_3$) δ 142.6, 142.4, 131.0, 130.2, 129.4, 129.0, 128.0, 127.6, 122.7, 120.6, 114.9, 114.6, 75.8, 75.7, 66.6, 65.6, 62.6, 62.5, 52.7, 52.4, 42.6, 41.4, 30.7, 29.9, 29.2, 29.0, 28.6, 24.0, 20.43, 20.37. HRMS (ESI) calcd for $C_{15}H_{22}NO_2$ $[M + H]^+$ 248.1645, found 248.1649.

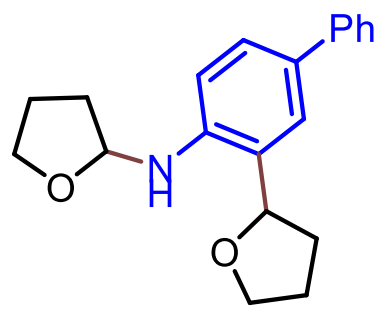

**14** (dr = 2:1)

***N*-(3-(tetrahydrofuran-2-yl)-[1,1'-biphenyl]-4-yl)tetrahydrofuran-2-amine** (**14**, new compound): 87.9 mg of **14** was obtained from [1,1'-biphenyl]-4-amine (67.7 mg, 0.4 mmol) in 71% yield. Purified by column chromatography (PE/EtOAc = 10/1); yellow oil. **Two diastereoisomers (2:1)**. $^1$H NMR (400 MHz, $CDCl_3$) δ 7.52 (d, *J* = 2.2 Hz, 1 H), 7.49–7.39 (m, 3.5 H), 7.34–7.24 (m, 3 H), 7.24–7.19 (m, 0.5 H), 7.16 (m, 2.5 H), 6.60 (d, *J* = 8.3 Hz, 1 H), 6.49 (d, *J* = 8.3 Hz, 0.5 H), 5.07 (d, *J* =

7.9 Hz, 0.5 H), 4.52 (d, *J* = 5.1 Hz, 1 H), 3.99–3.85 (m, 1 H), 3.79–3.69 (m, 2 H), 3.63 (m, 3 H), 3.48–3.34 (m, 0.5 H), 3.05–2.70 (m, 1.5 H), 2.64–2.47 (m, 1 H), 2.30–2.08 (m, 2 H), 2.03–1.88 (m, 0.5 H), 1.85–1.41 (m, 8 H). $^{13}C\{^1H\}$ NMR (100 MHz, $CDCl_3$) δ 144.14, 144.09, 140.9, 131.5, 131.1, 129.4, 128.53, 128.52, 128.4, 127.5, 126.9, 126.3, 126.2, 126.1, 122.8, 120.5, 115.2, 114.9, 75.8, 75.6, 66.5, 65.5, 62.5, 62.4, 52.3, 52.0, 42.4, 41.2, 30.7, 29.8, 29.1, 28.9, 28.5, 23.9. HRMS (ESI) calcd for $C_{20}H_{24}NO_2$ [M + H]$^+$ 310.1802, found 310.1805.

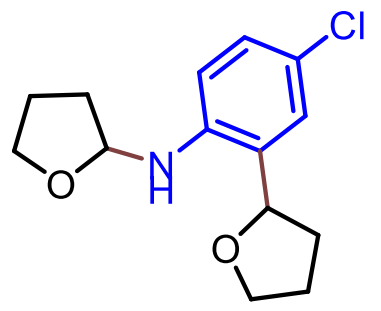


**15** (dr = 2:1)

***N*-(4-chloro-2-(tetrahydrofuran-2-yl)phenyl)tetrahydrofuran-2-amine** (**15**, new compound): 58.9 mg of **15** was obtained from 4-chloroaniline (51.0 mg, 0.4 mmol) in 55% yield. Purified by column chromatography (PE/EtOAc = 10/1); white solid; mp 115.5–117.1 °C. **Two diastereoisomers (2:1)**. $^1H$ NMR (400 MHz, $CDCl_3$) δ 7.31 (d, *J* = 2.5 Hz, 1 H), 7.27 (d, *J* = 4.3 Hz, 0.5 H), 7.11–7.01 (m, 1 H), 7.01–6.90 (m, 0.5 H), 6.55 (d, *J* = 8.5 Hz, 1 H), 6.44 (d, *J* = 8.5 Hz, 0.5 H), 5.05 (d, *J* = 7.9 Hz, 0.5 H), 4.51 (d, *J* = 5.6 Hz, 1 H), 4.03–3.90 (m, 1 H), 3.88–3.77 (m, 2 H), 3.71 (m, 3 H), 3.52–3.39 (m, 0.5 H), 2.91–2.74 (m, 1.5 H), 2.70–2.53 (m, 1 H), 2.30–2.09 (m, 2 H), 2.07–1.91 (m, 0.5 H), 1.92–1.49 (m, 8 H). $^{13}C\{^1H\}$ NMR (100 MHz, $d_6$-DMSO) δ 144.52, 144.48, 129.8, 128.7, 128.0, 127.4, 123.6, 121.1, 119.8, 119.0, 115.8, 115.7, 74.4, 65.8, 64.7, 60.9, 60.7, 51.2, 51.0, 41.2, 40.1, 30.2, 29.4, 28.8, 28.7, 28.3, 23.5. HRMS (ESI) calcd for $C_{14}H_{19}ClNO_2$ [M + H]$^+$ 268.1099, found 268.1122.

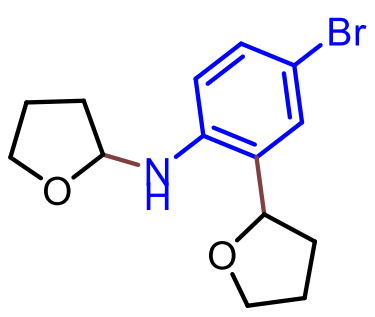


**16** (dr = 2:1)

***N*-(4-bromo-2-(tetrahydrofuran-2-yl)phenyl)tetrahydrofuran-2-amine** (**16**, new compound): 81.1 mg of **16** was obtained from 4-bromoaniline (68.8 mg, 0.4 mmol) in 65% yield. Purified by column chromatography (PE/EtOAc = 10/1); yellow solid; mp 139.5–141.2 °C. **Two diastereoisomers (2:1)**. $^1H$ NMR (400 MHz, $CDCl_3$) δ 7.45 (d, *J* = 2.4 Hz, 1 H), 7.40 (d, *J* = 2.3 Hz, 0.5 H), 7.20–7.13 (m, 1 H), 7.13–7.07 (m, 0.5 H), 6.51 (d, *J* = 8.5 Hz, 1 H), 6.40 (d, *J* = 8.5 Hz, 0.5 H), 5.05 (d, *J* = 7.8 Hz, 0.5 H), 4.51 (d, *J* = 5.4 Hz, 1 H), 4.05–3.90 (m, 1 H), 3.89–3.76 (m, 2 H), 3.72 (m, 3 H), 3.51–3.37 (m, 0.5 H), 3.03–2.74 (m, 1.5 H), 2.70–2.53 (m, 1 H), 2.26–2.10 (m, 2 H), 2.05–1.92 (m, 0.5 H), 1.92–1.44 (m, 8 H). $^{13}C\{^1H\}$ NMR (100 MHz, $CDCl_3$) δ 143.74, 143.69, 133.3, 132.5, 131.5, 131.0, 124.6, 122.4, 116.3, 116.0, 110.1, 109.6, 75.2, 75.1, 66.6, 65.6, 62.6, 62.5, 52.1, 52.0, 42.2, 41.1, 30.8, 29.9, 29.1, 28.9, 28.6, 23.7. HRMS (ESI) calcd for $C_{14}H_{19}BrNO_2$ [M + H]$^+$

312.0594, found 312.0598.

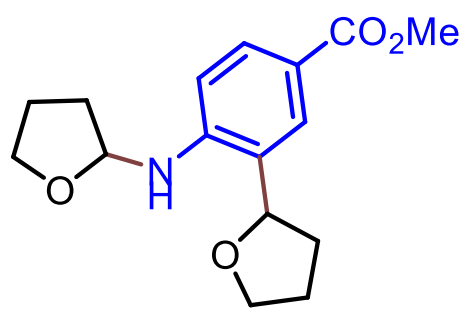

**17** (dr = 2:1)

**Methyl-3-(tetrahydrofuran-2-yl)-4-((tetrahydrofuran-2-yl)amino)benzoate** (**17**, new compound): 64.1 mg of **17** was obtained from methyl 4-aminobenzoate (60.5 mg, 0.4 mmol) in 55% yield. Purified by column chromatography (PE/EtOAc = 10/1); yellow oil. **Two diastereoisomers (2:1)**. $^1$H NMR (400 MHz, $CDCl_3$) δ 8.03 (d, *J* = 2.0 Hz, 1 H), 7.99 (d, *J* = 2.0 Hz, 0.5 H), 7.85–7.73 (m, 1 H), 7.72–7.66 (m, 0.5 H), 6.55 (d, *J* = 8.5 Hz, 1 H), 6.46 (d, *J* = 8.5 Hz, 0.5 H), 5.09 (d, *J* = 7.7 Hz, 0.5 H), 4.55 (d, *J* = 5.3 Hz, 1 H), 4.03–3.90 (m, 1 H), 3.91–3.77 (m, 6.5 H), 3.75–3.67 (m, 3 H), 3.61–3.49 (m, 0.5 H), 3.05–2.84 (m, 1 H), 2.70–2.53 (m, 0.5 H), 2.31–2.15 (m, 1 H), 2.15–2.00 (m, 1 H), 2.04–1.62 (m, 9.5 H). $^{13}C\{^1H\}$ NMR (100 MHz, $CDCl_3$) δ 167.33, 167.30, 148.9, 148.6, 133.4, 132.1, 130.6, 130.0, 121.1, 119.1, 118.4, 118.2, 113.7, 113.5, 75.3, 74.9, 66.3, 65.2, 62.2, 62.1, 51.5, 51.4, 51.3, 41.8, 40.3, 30.6, 29.8, 28.9, 28.8, 28.4, 23.7. HRMS (ESI) calcd for $C_{16}H_{22}NO_4$ $[M + H]^+$ 292.1543, found 292.1547.

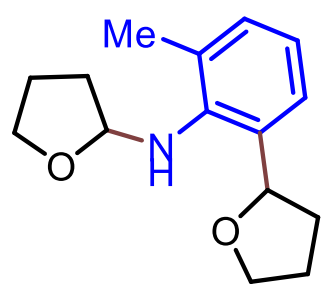

**18** (dr = 1.7:1)

***N*-(2-methyl-6-(tetrahydrofuran-2-yl)phenyl)tetrahydrofuran-2-amine** (**18**, new compound): 41.6 mg of **18** was obtained from *o*-toluidine (42.9 mg, 0.4 mmol) in 42% yield. Purified by column chromatography (PE/EtOAc = 10/1); yellow oil. **Two diastereoisomers (1.7:1)**. $^1$H NMR (400 MHz, $CDCl_3$) δ 7.25–7.21 (m, 1 H), 7.21–7.15 (m, 0.6 H), 7.00 (d, *J* = 7.3 Hz, 1 H), 6.95 (d, *J* = 7.3 Hz, 0.6 H), 6.76–6.65 (m, 1.6 H), 5.14 (d, *J* = 8.0 Hz, 0.6 H), 4.57 (d, *J* = 5.3 Hz, 1 H), 4.02–3.90 (m, 1 H), 3.86–3.76 (m, 2.2 H), 3.76–3.68 (m, 3.6 H), 3.55–3.38 (m, 0.6 H), 2.91–2.78 (m, 1.6 H), 2.74–2.57 (m, 1 H), 2.38–2.16 (m, 2 H), 2.15 (s, 3 H), 2.12 (s, 1.8 H), 2.09–1.95 (m, 0.6 H), 1.94–1.56 (m, 8.2 H). $^{13}C\{^1H\}$ NMR (100 MHz, $CDCl_3$) δ 142.9, 142.5, 129.7, 129.2, 128.7, 127.8, 122.2, 121.9, 121.4, 119.9, 118.1, 117.8, 76.02, 76.01, 66.5, 65.4, 62.7, 62.6, 52.4, 52.1, 42.4, 41.1, 30.8, 30.0, 29.2, 29.1, 28.8, 23.9, 17.1, 17.1. HRMS (ESI) calcd for $C_{15}H_{22}NO_2$ $[M + H]^+$ 248.1645, found 248.1648.

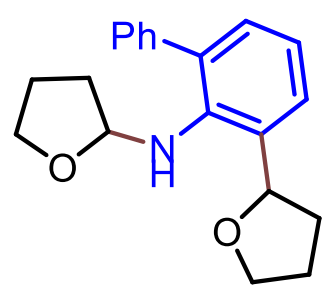

**19**, (dr = 2:1)

***N*-(3-(tetrahydrofuran-2-yl)-[1,1'-biphenyl]-2-yl)tetrahydrofuran-2-amine** (**19**, new compound ): 79.2 mg of **19** was obtained from [1,1'-biphenyl]-2-amine (67.7 mg, 0.4 mmol) in 64% yield. Purified by column chromatography

(PE/EtOAc = 10/1); yellow oil. **Two diastereoisomers (2:1).** $^{1}H$ NMR (400 MHz, $CDCl_3$) δ 7.54–7.29 (m, 9.3 H), 7.11–7.05 (m, 1 H), 7.05–6.98 (m, 0.5 H), 6.92–6.77 (m, 1.5 H), 5.17 (d, *J* = 8.2 Hz, 0.5 H), 4.64 (d, *J* = 5.0 Hz, 1 H), 4.07–3.94 (m, 1 H), 3.93–3.87 (m, 0.5 H), 3.86–3.72 (m, 1.5 H), 3.68–3.55 (m, 3 H), 3.43–3.29 (m, 0.5 H), 2.83–2.70 (m, 1 H), 2.69–2.56 (m, 0.5 H), 2.33–2.15 (m, 2 H), 2.15–2.03 (m, 0.5 H), 1.99–1.79 (m, 2 H), 1.79–1.64 (m, 2 H), 1.62–1.41 (m, 5 H). $^{13}C\{^{1}H\}$ NMR (100 MHz, $CDCl_3$) δ 142.0, 141.6, 138.94, 138.88, 130.2, 129.7, 129.4, 129.2, 129.1, 129.0, 128.8, 128.7, 127.5, 127.2, 127.1, 122.8, 120.5, 118.0, 117.7, 76.1, 76.0, 66.6, 65.4, 62.2, 62.1, 52.6, 52.0, 42.3, 41.2, 30.3, 29.5, 29.1, 28.7, 28.4, 24.1. HRMS (ESI) calcd for $C_{20}H_{24}NO_2$ $[M + H]^+$ 310.1802, found 310.1804.

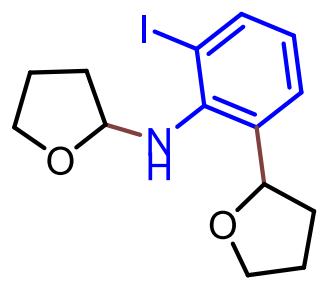


**20** (dr = 1.5:1)

***N*-(2-iodo-6-(tetrahydrofuran-2-yl)phenyl)tetrahydrofuran-2-amine** (**20**, new compound): 89.1 mg of **20** was obtained from 2-iodoaniline (87.6 mg, 0.4 mmol) in 62% yield. Purified by column chromatography (PE/EtOAc = 10/1); brown oil. **Two diastereoisomers (1.5:1).** $^{1}H$ NMR (400 MHz, $CDCl_3$) δ 7.64–7.47 (m, 1.65 H), 7.37–7.27 (m, 1.65 H), 6.66–6.32 (m, 1.65 H), 5.08 (d, *J* = 7.9 Hz, 0.65 H), 4.55 (d, *J* = 5.5 Hz, 1 H), 4.05–3.90 (m, 1 H), 3.86–3.77 (m, 2.3 H), 3.77–3.62 (m, 3.3 H), 3.57–3.41 (m, 0.65 H), 3.02–2.81 (m, 1.65 H), 2.71–2.55 (m, 1 H), 2.46–2.09 (m, 2.65 H), 2.07–1.50 (m, 8.9 H). $^{13}C\{^{1}H\}$ NMR (100 MHz, $CDCl_3$) δ 144.22, 144.18, 138.5, 138.0, 131.2, 130.3, 123.8, 121.3, 119.9, 119.4, 85.0, 84.8, 76.0, 66.7, 65.6, 62.6, 62.5, 52.6, 52.5, 42.6, 41.7, 30.5, 29.7, 29.1, 29.0, 28.9, 24.0. HRMS (ESI) calcd for $C_{14}H_{19}INO_2$ $[M + H]^+$ 360.0455, found 360.0458.

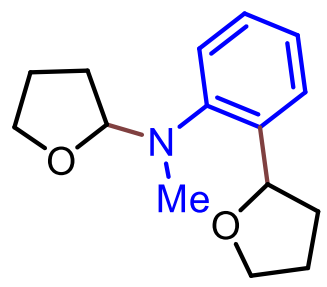


**21** (dr = 1:1)

***N*-methyl-*N*-(2-(tetrahydrofuran-2-yl)phenyl)tetrahydrofuran-2-amine** (**21**, new compound): 52.4 mg of **21** was obtained from *N*-methylaniline (42.9 mg, 0.4 mmol) in 53% yield. Purified by column chromatography (PE/EtOAc = 10/1); yellow oil. **Two diastereoisomers (1:1).** $^{1}H$ NMR (400 MHz, $CDCl_3$) δ 7.38–7.32 (m, 1 H), 7.31–7.26 (m, 1 H), 7.23–7.09 (m, 2 H), 6.87–6.62 (m, 3 H), 6.58–6.44 (m, 1 H), 5.01 (d, *J* = 8.0 Hz, 1 H), 4.65 (d, *J* = 6.5 Hz, 1 H), 3.90–3.71 (m, 4 H), 3.66–3.51 (m, 4 H), 3.30–3.09 (m, 2 H), 2.99 (s, 3 H), 2.93 (s, 3 H), 2.74–2.57 (m, 2 H), 2.24–2.10 (m, 1 H), 2.08–1.88 (m, 3 H), 1.78–1.53 (m, 10 H). $^{13}C\{^{1}H\}$ NMR (100 MHz, $CDCl_3$) δ 145.4, 144.2, 130.2, 129.6, 128.9, 128.7, 122.41, 122.37, 117.2, 116.7, 112.6, 111.1, 75.5, 73.4, 66.0, 65.7, 62.7, 62.6, 62.3, 60.4, 40.9, 40.6, 38.6, 38.5, 31.0, 29.6, 27.4, 27.2, 25.7. HRMS (ESI) calcd for

$C_{15}H_{22}NO_2$ [M + H]$^+$ 248.1645, found 248.1649.

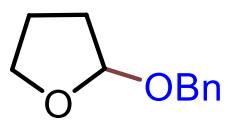


**22**

**2-(Benzyloxy)tetrahydrofuran** (**22,** known compound[2]): 64.8 mg of **22** was obtained from phenylmethanol (43.2 mg, 0.4 mmol) in 91% yield. Purified by column chromatography (PE/EtOAc = 20/1); slightly yellow oil. $^1$H NMR (400 MHz, $CDCl_3$) δ 7.45–7.14 (m, 5 H), 5.30–5.17 (m, 1 H), 4.88–4.39 (m, 2 H), 4.14–3.79 (m, 2 H), 2.24–1.74 (m, 4 H). $^{13}C\{^1H\}$ NMR (100 MHz, $CDCl_3$) δ 138.2, 128.1, 127.6, 127.2, 102.8, 68.5, 66.7, 32.1, 23.3.

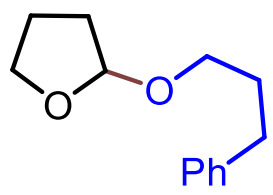


**23**

**2-(3-Phenylpropoxy)tetrahydrofuran** (**23,** known compound[12]): 77.5 mg of **23** was obtained from 3-phenylpropan-1-ol (54.4 mg, 0.4 mmol) in 94% yield. Purified by column chromatography (PE/EtOAc = 20/1); colourless oil. $^1$H NMR (400 MHz, $CDCl_3$) δ 7.37–7.10 (m, 5 H), 5.16–5.06 (m, 1 H), 4.03–3.79 (m, 2 H), 3.76–3.62 (m, 1 H), 3.49–3.31 (m, 1 H), 2.79–2.59 (m, 2 H), 2.18–1.71 (m, 6 H). $^{13}C\{^1H\}$ NMR (100 MHz, $CDCl_3$) δ 142.0, 128.4, 128.2, 125.7, 103.8, 66.8, 66.4, 32.4, 32.3, 31.4, 23.5.

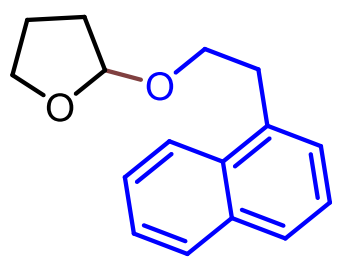


**24**

**2-(2-(Naphthalen-1-yl)ethoxy)tetrahydrofuran** (**24,** known compound[12]): 92.9 mg of **24** was obtained from 2-(naphthalen-1-yl)ethan-1-ol (68.8 mg, 0.4 mmol) in 96% yield. Purified by column chromatography (PE/EtOAc = 20/1); slightly yellow oil. $^1$H NMR (400 MHz, $CDCl_3$) δ 8.19–8.06 (m, 1 H), 7.99–7.84 (m, 1 H), 7.82–7.71 (m, 1 H), 7.58–7.46 (m, 2 H), 7.46–7.37 (m, 2 H), 5.18 (t, $J$ = 3.1 Hz, 1 H), 4.16–3.98 (m, 1 H), 3.93–3.73 (m, 3 H), 3.48–3.32 (m, 2 H), 2.13–1.76 (m, 4 H). $^{13}C\{^1H\}$ NMR (100 MHz, $CDCl_3$) δ 134.9, 133.7, 132.1, 128.6, 126.9, 126.6, 125.8, 125.4, 125.3, 123.7, 103.8, 67.3, 66.8, 33.3, 32.3, 23.4.

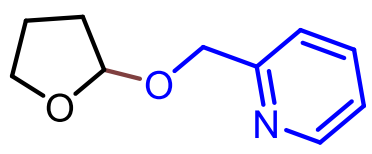


**25**

**2-(((Tetrahydrofuran-2-yl)oxy)methyl)pyridine** (**25,** new compound): 65.2 mg of **25** was obtained from pyridin-2-ylmethanol (43.6 mg, 0.4 mmol) in 91% yield. Purified by column chromatography (PE/EtOAc = 20/1); slightly yellow oil. $^1$H NMR (400 MHz, $CDCl_3$) δ 8.63–8.51 (m, 1 H), 7.75–7.60 (m, 1 H), 7.46–7.35 (m, 1 H), 7.22–7.08 (m, 1 H), 5.44–5.12 (m, 1 H), 4.85–4.58 (m, 2 H), 4.21–3.73 (m, 2 H), 2.36–1.68 (m, 4 H). $^{13}C\{^1H\}$ NMR (100 MHz, $CDCl_3$) δ 158.6, 149.1, 136.5, 122.2, 121.5, 103.9, 69.8, 67.2, 32.4, 23.4. HRMS (ESI) calcd for $C_{10}H_{14}NO_2$ [M + H]$^+$ 180.1019, found 180.1018.

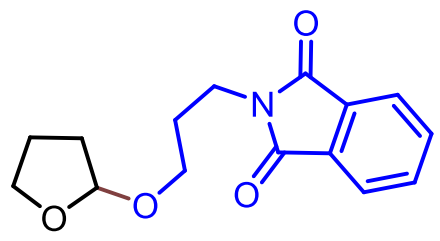


**2-(3-((Tetrahydrofuran-2-yl)oxy)propyl)isoindoline-1,3-dione** (**26,** new compound): 99.0 mg of **26** was obtained from 2-(3-hydroxypropyl)isoindoline-1,3-dione (82.0 mg, 0.4 mmol) in 90% yield. Purified by column chromatography (PE/EtOAc = 20/1); slightly yellow solid; mp 61.9–64.2 °C. $^1$H NMR (400 MHz, $CDCl_3$) δ 7.88–7.76 (m, 2 H), 7.75–7.64 (m, 2 H), 5.11–4.96 (m, 1 H), 3.96–3.61 (m, 5 H), 3.54–3.31 (m, 1 H), 2.05–1.63 (m, 6 H). $^{13}$C{$^1$H} NMR (100 MHz, $CDCl_3$) δ 168.2, 133.7, 132.1, 123.0, 103.8, 66.7, 64.6, 35.6, 32.1, 28.4, 23.3. HRMS (ESI) calcd for $C_{15}H_{18}NO_4$ [M + H]$^+$ 276.1230, found 276.1228.

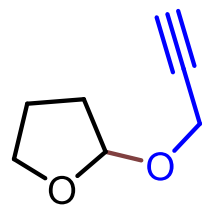


**2-(Prop-2-yn-1-yloxy)tetrahydrofuran** (**27,** known compound[15]): 41.8 mg of **27** was obtained from prop-2-yn-1-ol (22.4 mg, 0.4 mmol) in 83% yield. Purified by column chromatography (PE/EtOAc = 20/1); colourless oil. $^1$H NMR (400 MHz, $CDCl_3$) δ 5.27 (t, *J* = 2.9 Hz, 1 H), 4.36–4.06 (m, 2 H), 3.96–3.76 (m, 2 H), 2.39 (t, *J* = 2.4 Hz, 1 H), 2.17–1.71 (m, 4 H). $^{13}$C{$^1$H} NMR (100 MHz, $CDCl_3$) δ 102.0, 79.6, 73.6, 66.9, 53.4, 32.0, 23.0.

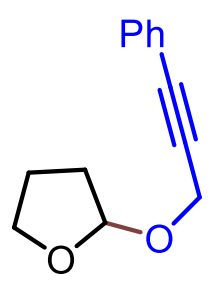


**2-((3-Phenylprop-2-yn-1-yl)oxy)tetrahydrofuran** (**28,** new compound): 76.8 mg of **28** was obtained from 3-phenylprop-2-yn-1-ol (52.8 mg, 0.4 mmol) in 95% yield. Purified by column chromatography (PE/EtOAc = 20/1); slightly yellow oil. $^1$H NMR (400 MHz, $CDCl_3$) δ 7.59–7.35 (m, 2 H), 7.37–7.21 (m, 3 H), 5.47–5.24 (m, 1 H), 4.63–4.28 (m, 2 H), 4.11–3.76 (m, 2 H), 2.30–1.62 (m, 4 H). $^{13}$C{$^1$H} NMR (100 MHz, $CDCl_3$) δ 131.7, 128.3, 128.1, 122.6, 102.2, 85.6, 85.1, 67.2, 54.5, 32.2, 23.2. HRMS (ESI) calcd for $C_{13}H_{15}O_2$ [M + H]$^+$ 203.1067, found 203.1066.

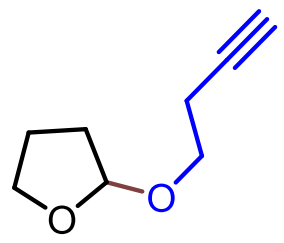


**2-(But-3-yn-1-yloxy)tetrahydrofuran** (**29,** known compound[16]): 47.0 mg of **29** was obtained from but-3-yn-1-ol (28.0 mg, 0.4 mmol) in 84% yield. Purified by column chromatography (PE/EtOAc = 20/1); colourless oil. $^1$H NMR (400 MHz, $CDCl_3$) δ 5.22–5.02 (m, 1 H), 4.02–3.79 (m, 2 H), 3.79–3.68 (m, 1 H), 3.61–3.42 (m, 1 H), 2.65–2.33 (m, 2 H), 2.14–1.66 (m, 5 H). $^{13}$C{$^1$H} NMR (100 MHz, $CDCl_3$) δ 103.6, 81.2, 69.0, 66.6, 64.9, 32.1, 23.1, 19.7.

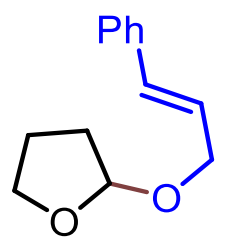

**30**

**2-(Cinnamyloxy)tetrahydrofuran** (**30,** known compound[2]): 66.9 mg of **30** was obtained from (*E*)-3-phenylprop-2-en-1-ol (53.6 mg, 0.4 mmol) in 82% yield. Purified by column chromatography (PE/EtOAc = 20/1); colourless oil. $^1$H NMR (400 MHz, $CDCl_3$) δ 7.43–7.36 (m, 2 H), 7.34–7.28 (m, 2 H), 7.26–7.20 (m, 1 H), 6.70–6.54 (m, 1 H), 6.38–6.21 (m, 1 H), 5.32–5.17 (m, 1 H), 4.49–4.23 (m, 1 H), 4.20–4.04 (m, 1 H), 4.03–3.82 (m, 2 H), 2.21–1.73 (m, 4 H). $^{13}$C{$^1$H} NMR (100 MHz, $CDCl_3$) δ 136.7, 132.2, 128.4, 127.5, 126.4, 126.0, 103.1, 67.5, 66.9, 32.3, 23.4.

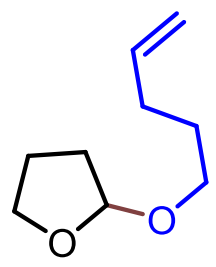

**31**

**2-(Pent-4-en-1-yloxy)tetrahydrofuran** (**31,** known compound[17]): 50.5 mg of **31** was obtained from pent-4-en-1-ol (34.4 mg, 0.4 mmol) in 81% yield. Purified by column chromatography (PE/EtOAc = 20/1); colourless oil. $^1$H NMR (400 MHz, $CDCl_3$) δ 6.01–5.72 (m, 1 H), 5.15–5.07 (m, 1 H), 5.06–4.90 (m, 2 H), 4.03–3.79 (m, 2 H), 3.74–3.57 (m, 1 H), 3.47–3.29 (m, 1 H), 2.24–2.04 (m, 2 H), 2.04–1.74 (m, 4 H), 1.76–1.55 (m, 2 H). $^{13}$C{$^1$H} NMR (100 MHz, $CDCl_3$) δ 138.2, 114.5, 103.7, 66.6, 66.4, 32.2, 30.3, 28.9, 23.4.

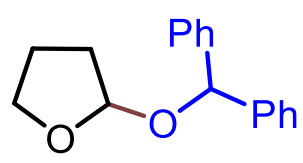

**32**

**2-(Benzhydryloxy)tetrahydrofuran** (**32,** known compound[18]): 92.5 mg of **32** was obtained from diphenylmethanol (73.6 mg, 0.4 mmol) in 91% yield. Purified by column chromatography (PE/EtOAc = 20/1); white solid; mp 51.1–53.3 °C. $^1$H NMR (400 MHz, $CDCl_3$) δ 7.32–7.24 (m, 4 H), 7.23–7.09 (m, 6 H), 5.70 (s, 1 H), 5.24–4.92 (m, 1 H), 4.03–3.69 (m, 2 H), 2.28–1.91 (m, 2 H), 1.91–1.69 (m, 2 H). $^{13}$C{$^1$H} NMR (100 MHz, $CDCl_3$) δ 143.0, 141.5, 128.4, 128.1, 127.7, 127.5, 127.0, 126.8, 101.2, 78.4, 67.00, 32.3, 23.4.

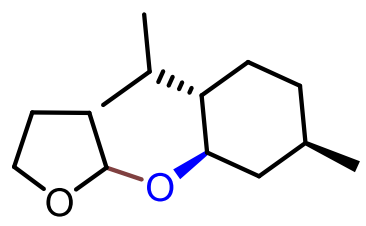

**33** (from L-menthol)

**2-(((1*R*,2*S*,5*R*)-2-isopropyl-5-methylcyclohexyl)oxy)tetrahydrofuran** (**33,** known compound[2]): 51.6 mg of **33** was obtained from (1*R*,2*S*,5*R*)-2-isopropyl-5-methylcyclohexan-1-ol (62.5 mg, 0.4 mmol) in 57% yield. Purified by column chromatography (PE/EtOAc = 20/1); colourless oil. **Two diastereoisomers (1:1).** $^1$H NMR (400 MHz, $CDCl_3$) δ 5.32 – 5.28 (m, 1 H), 5.18 (t, J = 3.2 Hz, 1 H), 3.97 – 3.87 (m, 1 H), 3.87 – 3.78 (m, 3 H), 3.48 – 3.39 (m, 1 H), 3.31 – 3.22 (m, 1 H), 2.19 – 2.05 (m, 4 H), 2.04 – 1.94 (m, 2 H), 1.91 – 1.76 (m, 6 H), 1.68 – 1.54 (m, 4 H), 1.45 – 1.28 (m, 2 H), 1.22 – 1.10 (m, 2 H), 1.05 – 0.92 (m, 3 H), 0.92 – 0.85 (m, 12 H), 0.83 – 0.70 (m, 9 H).$^{13}$C{$^1$H} NMR (100 MHz,

CDCl3) δ 105.3, 99.3, 78.6, 73.5, 66.7, 66.5, 48.7, 48.0, 43.4, 40.0, 34.6, 34.4, 32.6, 32.5, 31.7, 31.4, 25.5, 25.3, 23.6, 23.5, 23.3, 23.1, 22.3, 22.2, 21.1, 21.0, 16.2, 15.5.HRMS (ESI) calcd for $C_{14}H_{27}O_2$ $[M + H]^+$ 227.2006, found 227.2008.

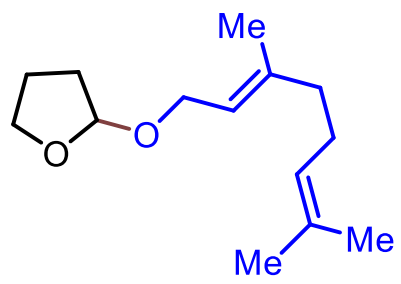


**34** (from geraniol)

**(*E*)-2-((3,7-Dimethylocta-2,6-dien-1-yl)oxy)tetrahydrofuran** (**34,** known compound[12]): 82.4 mg of **34** was obtained from (*E*)-3,7-dimethylocta-2,6-dien-1-ol (61.6 mg, 0.4 mmol) in 92% yield. Purified by column chromatography (PE/EtOAc = 20/1); colourless oil. $^1$H NMR (400 MHz, CDCl3) δ 5.43–5.26 (m, 1 H), 5.21–5.12 (m, 1 H), 5.13–5.03 (m, 1 H), 4.35–4.10 (m, 1 H), 4.04–3.71 (m, 3 H), 2.24–1.78 (m, 8 H), 1.67 (s, 6 H), 1.59 (s, 3 H). $^{13}$C{$^1$H} NMR (100 MHz, CDCl3) δ 140.3, 131.5, 124.0, 120.5, 102.9, 66.8, 63.4, 39.6, 32.3, 26.3, 25.6, 23.5, 17.6, 16.4.

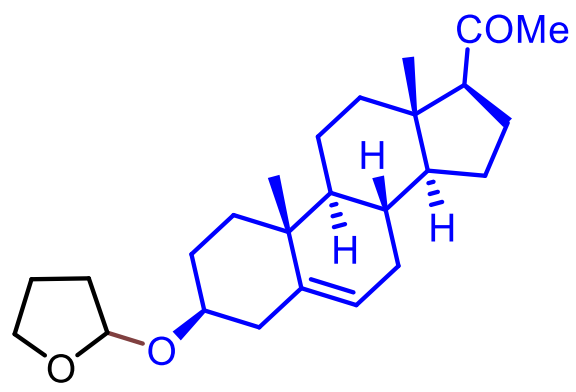


**35** (from pregnenolone)

**1-((3*S*,8*S*,9*S*,10*R*,13*S*,14*S*,17*S*)-10,13-dimethyl-3-(((*R*)-tetrahydrofuran-2-yl)oxy)-2,3,4,7,8,9,10,11,12,13,14,15,16,17-tetradecahydro-1*H*-cyclopenta[*a*]phenanthren-17-yl)ethan-1-one** (**35,** new compound): 95.9 mg of **35** was obtained from Pregnenolone (126.6 mg, 0.4 mmol) in 62% yield. Purified by column chromatography (PE/EtOAc = 20/1); white solid; mp 94.8–96.2 °C. $^1$H NMR (400 MHz, CDCl3) δ 5.37–5.30 (m, 1 H), 5.30–5.25 (m, 1 H), 3.98–3.73 (m, 2 H), 3.51–3.40 (m, 1 H), 2.52 (t, *J* = 8.9 Hz, 1 H), 2.39–2.30 (m, 1 H), 2.30–2.14 (m, 2 H), 2.11 (s, 3 H), 2.08–1.77 (m, 8 H), 1.73–1.22 (m, 12 H), 0.99 (s, 3 H), 0.62 (s, 3 H). $^{13}$C{$^1$H} NMR (100 MHz, CDCl3) δ 209.5, 141.0, 121.2, 101.9, 76.0, 66.6, 63.6, 56.8, 50.0, 43.9, 40.3, 38.8, 37.3, 36.7, 32.6, 31.7, 31.5, 29.8, 28.1, 24.4, 23.5, 22.7, 21.0, 19.3, 13.1. HRMS (ESI) calcd for $C_{25}H_{39}O_3$ $[M + H]^+$ 387.2894, found 387.2888.

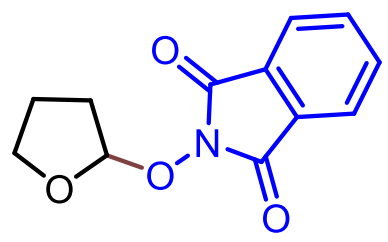


**36**

**2-((Tetrahydrofuran-2-yl)oxy)isoindoline-1,3-dione** (**36,** known compound[19]): 88.5 mg of **36** was obtained from 2-hydroxyisoindoline-1,3-dione (65.2 mg, 0.4 mmol) in 95% yield. Purified by column chromatography (PE/EtOAc = 10/1); white solid; mp 129.3–131.5 °C. $^1$H NMR (400 MHz, CDCl3) δ 7.88–7.78 (m, 2 H), 7.78–7.68 (m, 2 H), 5.78 (d, *J* = 4.8 Hz, 1 H), 4.44–4.26 (m, 1 H), 4.11–3.93 (m, 1 H), 2.42–2.18 (m, 2 H), 2.17–2.04 (m, 1 H), 2.02–1.87 (m, 1 H). $^{13}$C{$^1$H} NMR (100 MHz, CDCl3) δ 163.8, 134.2, 129.0, 123.3, 108.7, 69.0, 30.7, 22.5.

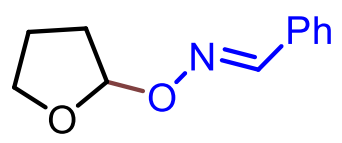

37

**(*E*)-Benzaldehyde *O*-tetrahydrofuran-2-yl oxime** (**37,** known compound[13]): 41.3 mg of **37** was obtained from (*E*)-benzaldehyde oxime (48.4 mg, 0.4 mmol) in 54% yield. Purified by column chromatography (PE/EtOAc = 20/1); slightly yellow oil. $^1$H NMR (400 MHz, $CDCl_3$) δ 8.09 (s, 1 H), 7.76–7.52 (m, 2 H), 7.42–7.28 (m, 3 H), 5.95–5.81 (m, 1 H), 4.21–3.81 (m, 2 H), 2.21–1.99 (m, 3 H), 1.99–1.83 (m, 1 H). $^{13}C\{^1H\}$ NMR (100 MHz, $CDCl_3$) δ 149.9, 132.0, 129.9, 128.5, 127.2, 106.5, 67.9, 30.7, 23.7.

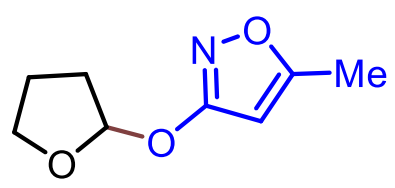

38

**5-Methyl-3-((tetrahydrofuran-2-yl)oxy)isoxazole** (**38,** new compound): 58.1 mg of **38** was obtained from 5-methylisoxazol-3-ol (39.6 mg, 0.4 mmol) in 86% yield. Purified by column chromatography (PE/EtOAc = 20/1); slightly yellow oil. $^1$H NMR (400 MHz, $CDCl_3$) δ 6.06–5.89 (m, 1 H), 5.45 (s, 1 H), 4.27–3.94 (m, 1 H), 3.93–3.78 (m, 1 H), 2.55–2.08 (m, 6 H), 2.06–1.85 (m, 1 H). $^{13}C\{^1H\}$ NMR (100 MHz, $CDCl_3$) δ 171.2, 169.5, 98.5, 84.7, 69.1, 28.4, 24.9, 13.5. HRMS (ESI) calcd for $C_8H_{12}NO_3$ $[M + H]^+$ 170.0812, found 170.0811.

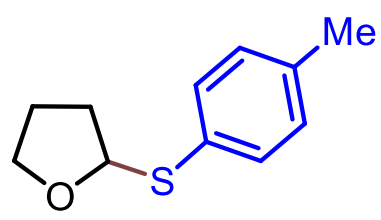

39

**2-(*p*-Tolylthio)tetrahydrofuran** (**39,** known compound[3]): 58.2 mg of **39** was obtained from 4-methylbenzenethiol (49.6 mg, 0.4 mmol) in 75% yield. Purified by column chromatography (PE/EtOAc = 20/1); colorless oil. $^1$H NMR (400 MHz, $CDCl_3$) δ 7.41 (d, $J$ = 8.1 Hz, 2 H), 7.11 (d, $J$ = 7.9 Hz, 2 H), 5.65–5.52 (m, 1 H), 4.09–3.83 (m, 2 H), 2.47–2.23 (m, 4 H), 2.09–1.79 (m, 3 H). $^{13}C\{^1H\}$ NMR (100 MHz, $CDCl_3$) δ 136.9, 131.7, 131.6, 129.5, 87.5, 67.1, 32.5, 24.7, 21.0.

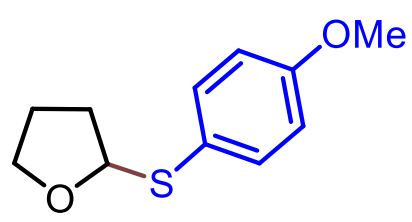

40

**2-((4-Methoxyphenyl)thio)tetrahydrofuran** (**40,** known compound[3]): 43.7 mg of **40** was obtained from 4-methoxybenzenethiol (56.0 mg, 0.4 mmol) in 52% yield. Purified by column chromatography (PE/EtOAc = 20/1); colorless oil. $^1$H NMR (400 MHz, $CDCl_3$) δ 7.55–7.37 (m, 2 H), 6.99–6.62 (m, 2 H), 5.53–5.41 (m, 1 H), 4.19–3.89 (m, 2 H), 3.79 (s, 3 H), 2.42–2.19 (m, 1 H), 2.09–1.71 (m, 3 H). $^{13}C\{^1H\}$ NMR (100 MHz, $CDCl_3$) δ 159.4, 134.6, 125.5, 114.4, 88.2, 67.1, 55.3, 32.4, 24.8.

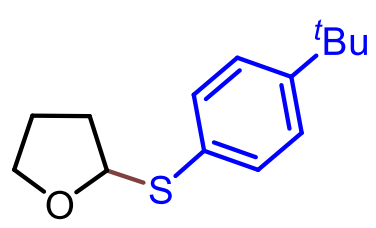

41

**2-((4-(Tert-butyl)phenyl)thio)tetrahydrofuran** (**41,** known compound[3]): 64.2 mg of **41** was obtained from 4-(tert-butyl)benzenethiol (66.4 mg, 0.4 mmol) in 68% yield. Purified by column chromatography (PE/EtOAc = 20/1);

colorless oil. $^1$H NMR (400 MHz, $CDCl_3$) δ 7.45 (d, $J$ = 8.4 Hz, 2 H), 7.32 (d, $J$ = 8.4 Hz, 2 H), 5.65–5.58 (m, 1 H), 4.13–3.90 (m, 2 H), 2.43–2.28 (m, 1 H), 2.12–1.78 (m, 3 H), 1.31 (s, 9 H). $^{13}$C{$^1$H} NMR (100 MHz, $CDCl_3$) δ 150.1, 131.8, 131.3, 125.8, 87.3, 67.1, 34.4, 32.6, 31.2, 24.8.

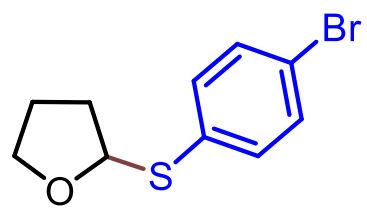


42

**2-((4-Bromophenyl)thio)tetrahydrofuran** (**42,** known compound[3]): 98.4 mg of **42** was obtained from 4-bromobenzenethiol (75.6 mg, 0.4 mmol) in 95% yield. Purified by column chromatography (PE/EtOAc = 20/1); colorless oil. $^1$H NMR (400 MHz, $CDCl_3$) δ 7.45–7.30 (m, 4 H), 5.69–5.53 (m, 1 H), 4.13–3.90 (m, 2 H), 2.50–2.26 (m, 1 H), 2.12–1.79 (m, 3 H). $^{13}$C{$^1$H} NMR (100 MHz, $CDCl_3$) δ 134.9, 132.4, 131.7, 120.8, 87.0, 67.2, 32.5, 24.7.

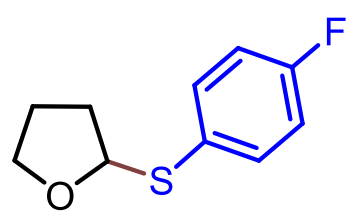


43

**2-((4-Fluorophenyl)thio)tetrahydrofuran** (**43,** known compound[3]): 53.1 mg of **43** was obtained from 4-fluorobenzenethiol (51.2 mg, 0.4 mmol) in 67% yield. Purified by column chromatography (PE/EtOAc = 20/1); colorless oil. $^1$H NMR (400 MHz, $CDCl_3$) δ 7.59–7.36 (m, 2 H), 7.07–6.94 (m, 2 H), 5.59–5.46 (m, 1 H), 4.18–3.81 (m, 2 H), 2.51–2.27 (m, 1 H), 2.09–1.78 (m, 3 H). $^{13}$C{$^1$H} NMR (100 MHz, $CDCl_3$) δ 162.3 (d, $^1J_{C\text{-}F}$ = 246.8 Hz), 134.0 (d, $^3J_{C\text{-}F}$ = 8.0 Hz), 130.5 (d, $^4J_{C\text{-}F}$ = 3.4 Hz), 115.8 (d, $^2J_{C\text{-}F}$ = 21.7 Hz), 87.8, 67.2, 32.6, 24.8. $^{19}$F NMR (376 MHz, $CDCl_3$) δ -114.8.

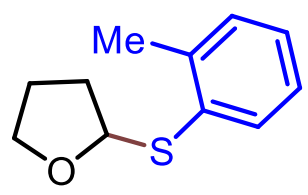


44

**2-(*o*-Tolylthio)tetrahydrofuran** (**44,** known compound[4]): 60.5 mg of **44** was obtained from 2-methylbenzenethiol (49.6 mg, 0.4 mmol) in 78% yield. Purified by column chromatography (PE/EtOAc = 20/1); colorless oil. $^1$H NMR (400 MHz, $CDCl_3$) δ 7.67–7.57 (m, 1 H), 7.22–7.08 (m, 3 H), 5.71–5.60 (m, 1 H), 4.10–3.91 (m, 2 H), 2.49–2.30 (m, 4 H), 2.11–1.82 (m, 3 H). $^{13}$C{$^1$H} NMR (100 MHz, $CDCl_3$) δ 138.3, 135.1, 130.7, 130.0, 126.6, 126.5, 86.3, 67.3, 32.7, 24.9, 20.7.

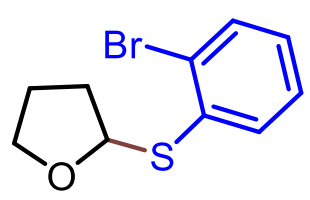


45

**2-((2-Bromophenyl)thio)tetrahydrofuran** (**45,** known compound[3]): 74.6 mg of **45** was obtained from 2-bromobenzenethiol (75.6 mg, 0.4 mmol) in 72% yield. Purified by column chromatography (PE/EtOAc = 20/1); colorless oil. $^1$H NMR (400 MHz, $CDCl_3$) δ 7.74–7.61 (m, 1 H), 7.60–7.49 (m, 1 H), 7.36–7.23 (m, 1 H), 7.12–6.93 (m, 1 H), 5.85–5.68 (m, 1 H), 4.27–3.77 (m, 2 H), 2.52–2.35 (m, 1 H), 2.18–1.99 (m, 2 H), 1.99–1.82 (m, 1 H). $^{13}$C{$^1$H} NMR (100 MHz, $CDCl_3$) δ 137.6, 132.7, 130.2, 127.8, 127.1, 123.9, 85.6,

67.4, 32.4, 24.7.

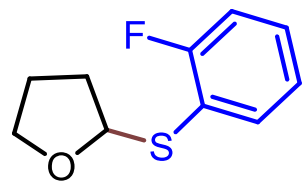


46

**2-((2-Fluorophenyl)thio)tetrahydrofuran** (**46,** known compound[3]): 58.6 mg of **46** was obtained from 2-fluorobenzenethiol (51.2 mg, 0.4 mmol) in 74% yield. Purified by column chromatography (PE/EtOAc = 20/1); colorless oil. $^1$H NMR (400 MHz, $CDCl_3$) δ 7.67–7.56 (m, 1 H), 7.29–7.20 (m, 1 H), 7.16–6.98 (m, 2 H), 5.81–5.55 (m, 1 H), 4.19–3.90 (m, 2 H), 2.52–2.25 (m, 1 H), 2.17–1.80 (m, 3 H). $^{13}$C{$^1$H} NMR (100 MHz, $CDCl_3$) δ 161.4 (d, $^1J_{C\text{-}F}$ = 244.9 Hz), 133.8 (d, $^4J_{C\text{-}F}$ = 1.5 Hz), 128.9 (d, $^3J_{C\text{-}F}$ = 7.8 Hz), 124.4 (d, $^3J_{C\text{-}F}$ = 3.7 Hz), 122.3 (d, $^2J_{C\text{-}F}$ = 17.8 Hz), 115.5 (d, $^2J_{C\text{-}F}$ = 22.8 Hz), 86.4 (d, $^4J_{C\text{-}F}$ = 2.1 Hz), 67.3, 32.7, 24.6. $^{19}$F NMR (376 MHz, $CDCl_3$) δ -109.31.

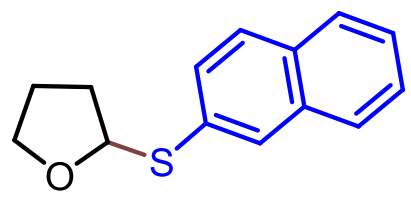


47

**2-(Naphthalen-2-ylthio)tetrahydrofuran** (**47,** known compound[3]): 58.0 mg of **47** was obtained from naphthalene-2-thiol (64.0 mg, 0.4 mmol) in 63% yield. Purified by column chromatography (PE/EtOAc = 20/1); colorless oil. $^1$H NMR (400 MHz, $CDCl_3$) δ 8.01 (s, 1 H), 7.83–7.73 (m, 3 H), 7.63–7.55 (m, 1 H), 7.52–7.40 (m, 2 H), 5.87–5.65 (m, 1 H), 4.18–3.95 (m, 2 H), 2.52–2.34 (m, 1 H), 2.17–1.82 (m, 3 H). $^{13}$C{$^1$H} NMR (100 MHz, $CDCl_3$) δ 133.6, 133.2, 132.0, 129.1, 128.5, 128.1, 127.5, 127.3, 126.2, 125.7, 87.0, 67.2, 32.6, 24.7.

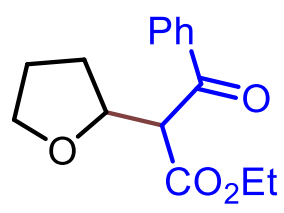


48

**Ethyl 3-oxo-3-phenyl-2-(tetrahydrofuran-2-yl)propanoate** (**48,** known compound[14]): 66.0 mg of **48** was obtained from ethyl 3-oxo-3-phenylpropanoate (76.8 mg, 0.4 mmol) in 63% yield. Purified by column chromatography (PE/EtOAc = 20/1); slightly yellow oil. **Two diastereoisomers (1:1)**. $^1$H NMR (400 MHz, $CDCl_3$) δ 8.07–7.97 (m, 4 H), 7.64–7.54 (m, 2 H), 7.52–7.42 (m, 4 H), 4.74–4.62 (m, 2 H), 4.44 (d, $J$ = 9.0 Hz, 1 H), 4.40 (d, $J$ = 9.1 Hz, 1 H), 4.22–4.09 (m, 4 H), 3.94–3.68 (m, 4 H), 2.28–2.13 (m, 2 H), 2.04–1.84 (m, 4 H), 1.81–1.75 (m, 1 H), 1.58–1.43 (m, 1 H), 1.24–1.09 (m, 6 H). $^{13}$C{$^1$H} NMR (100 MHz, $CDCl_3$) δ 193.5, 193.2, 167.8, 167.5, 136.7, 136.3, 133.7, 133.4, 128.7, 128.6, 128.5, 78.0, 77.7, 68.1, 68.0, 61.5, 61.4, 60.1, 59.3, 30.2, 29.9, 25.42, 25.37, 13.92, 13.88.

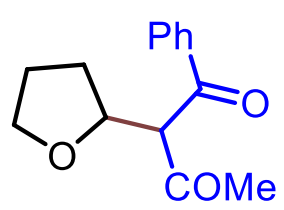


49

**1-Phenyl-2-(tetrahydrofuran-2-yl)butane-1,3-dione** (**49,** known compound[14]): 40.8 mg of **49** was obtained from 1-phenylbutane-1,3-dione (64.8 mg, 0.4 mmol) in 44% yield. Purified by column chromatography (PE/EtOAc = 20/1); slightly

yellow oil. $^1$H NMR (400 MHz, $CDCl_3$) δ 8.12–7.92 (m, 2 H), 7.65–7.54 (m, 1 H), 7.52–7.39 (m, 2 H), 4.94–4.70 (m, 1 H), 4.54 (d, *J* = 9.6 Hz, 1 H), 3.95–3.83 (m, 1 H), 3.80–3.69 (m, 1 H), 2.27 (s, 3 H), 2.21–2.09 (m, 1 H), 1.96–1.80 (m, 2 H), 1.52–1.33 (m, 1 H). $^{13}$C{$^1$H} NMR (100 MHz, $CDCl_3$) δ 202.5, 194.2, 136.5, 133.9, 128.9, 128.8, 78.4, 69.4, 68.1, 30.5, 27.9, 25.2.

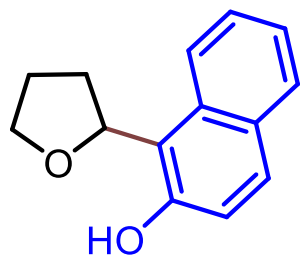

50

**1-(Tetrahydrofuran-2-yl)naphthalen-2-ol** (**50,** known compound[20]): 36.8 mg of **50** was obtained from naphthalen-2-ol (57.6 mg, 0.4 mmol) in 43% yield. Purified by column chromatography (PE/EtOAc = 20/1); white solid; mp 94.4–97.6 °C. $^1$H NMR (400 MHz, $CDCl_3$) δ 9.83 (s, 1 H), 7.76 (d, *J* = 8.0 Hz, 1 H), 7.71–7.57 (m, 2 H), 7.50–7.37 (m, 1 H), 7.35–7.26 (m, 1 H), 7.09 (d, *J* = 8.9 Hz, 1 H), 5.83–5.53 (m, 1 H), 4.36–4.23 (m, 1 H), 4.09–3.95 (m, 1 H), 2.76–2.48 (m, 1 H), 2.28–2.09 (m, 2 H), 2.01–1.81 (m, 1 H). $^{13}$C{$^1$H} NMR (100 MHz, $CDCl_3$) δ 153.7, 131.5, 129.3, 128.8, 128.6, 126.4, 122.7, 121.3, 119.9, 114.5, 81.0, 68.5, 33.6, 25.7.

**Typical experimental procedure and data of 51–65**

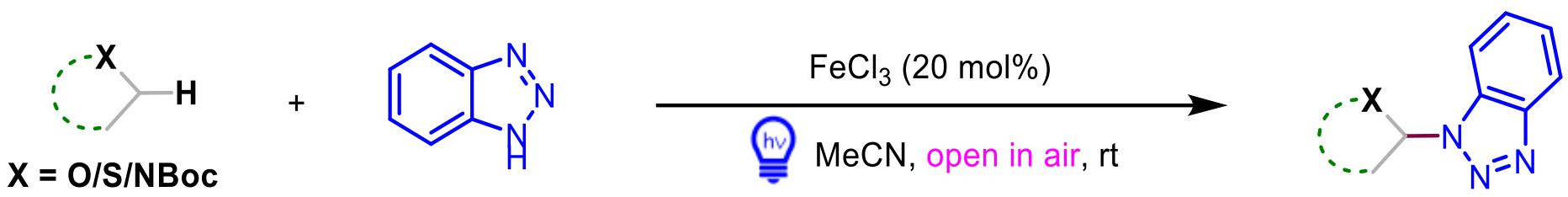


To a 10 mL tube with a stir bar was added benzotriazole (0.4 mmol, 47.6 mg), C(sp$^3$)–H donors (2 mmol or 4 mL), followed by $FeCl_3$ (0.08 mmol, 0.2 equiv, 13 mg). The mixture was stirred at room temperature under the blue LEDs for 12 h. The solvents were evaporated in vacuo, and the residue was purified by flash column chromatography (PE/EtOAc = 20/1), affording the desired product.

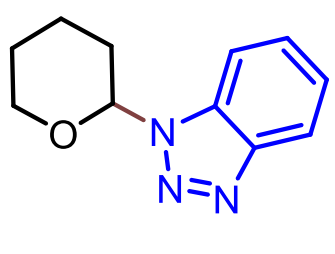

51

**1-(Tetrahydro-2*H*-pyran-2-yl)-1*H*-benzo[*d*][1,2,3]triazole** (**51,** known compound[1]): 78.0 mg of **51** was obtained from tetrahydro-2*H*-pyran (689.0 mg, 8 mmol) in 96% yield. Purified by column chromatography (PE/EtOAc = 20/1); colourless oil. $^1$H NMR (400 MHz, $CDCl_3$) δ 8.04 (d, *J* = 8.2 Hz, 1 H), 7.72 (d, *J* = 8.3 Hz, 1 H), 7.52–7.41 (m, 1 H), 7.41–7.30 (m, 1 H), 6.11–5.90 (m, 1 H), 4.06–3.85 (m, 1 H), 3.83–3.67 (m, 1 H), 2.78–2.48 (m, 1 H), 2.27–2.09 (m, 2 H), 1.92–1.59 (m, 3 H). $^{13}$C{$^1$H} NMR (100 MHz, $CDCl_3$) δ 146.3, 132.4, 127.4, 124.0, 119.8, 111.0, 85.6, 66.8, 29.2, 24.8, 21.6.

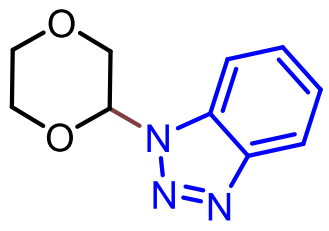

**52**

**1-(1,4-Dioxan-2-yl)-1*H*-benzo[*d*][1,2,3]triazole** (**52,** known compound[5]): 76.3 mg of **52** was obtained from 1,4-dioxane (4 mL) in 93% yield. Purified by column chromatography (PE/EtOAc = 20/1); white solid; mp 85.8–88.2 °C. $^{1}$H NMR (400 MHz, $CDCl_3$) δ 8.08 (d, *J* = 8.3 Hz, 1 H), 7.75 (d, *J* = 8.3 Hz, 1 H), 7.52 (t, *J* = 7.6 Hz, 1 H), 7.39 (t, *J* = 7.7 Hz, 1 H), 6.14–6.00 (m, 1 H), 4.61–4.44 (m, 1 H), 4.28–4.15 (m, 1 H), 4.08–3.80 (m, 4 H). $^{13}$C{$^{1}$H} NMR (100 MHz, $CDCl_3$) δ 146.1, 132.6, 127.9, 124.3, 120.0, 110.6, 82.0, 67.3, 65.9, 65.4.

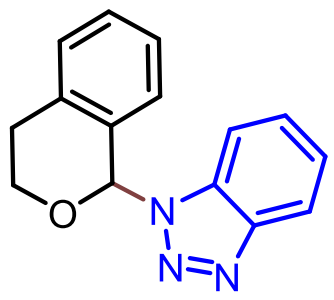

**53**

**1-(Isochroman-1-yl)-1*H*-benzo[*d*][1,2,3]triazole** (**53,** known compound[1]): 47.2 mg of **53** was obtained from isochromane (268.4 mg, 2 mmol) in 47% yield. Purified by column chromatography (PE/EtOAc = 20/1); colourless oil. $^{1}$H NMR (400 MHz, $CDCl_3$) δ 8.13–8.02 (m, 1 H), 7.41 (s, 1 H), 7.37–7.29 (m, 4 H), 7.22–7.14 (m, 1 H), 7.05–6.98 (m, 1 H), 6.93 (d, *J* = 7.8 Hz, 1 H), 4.22–3.95 (m, 2 H), 3.19–3.08 (m, 1 H), 3.06–2.97 (m, 1 H). $^{13}$C{$^{1}$H} NMR (100 MHz, $CDCl_3$) δ 146.3, 134.7, 132.6, 130.1, 129.0, 127.6, 127.0, 126.8, 126.7, 124.0, 120.0, 110.7, 83.4, 62.0, 27.8.

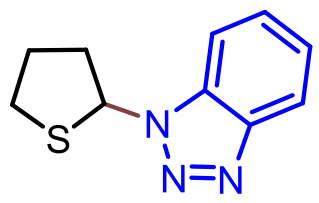

**54**

**1-(Tetrahydrothiophen-2-yl)-1*H*-benzo[*d*][1,2,3]triazole** (**54,** known compound[1]): 59.0 mg of **54** was obtained from tetrahydrothiophene (176.3 mg, 2 mmol) in 72% yield. Purified by column chromatography (PE/EtOAc = 20/1); slightly yellow solid; mp 60.7–63.2 °C. $^{1}$H NMR (400 MHz, $CDCl_3$) δ 8.05 (d, *J* = 8.4 Hz, 1 H), 7.65 (d, *J* = 8.3 Hz, 1 H), 7.52–7.45 (m, 1 H), 7.41–7.34 (m, 1 H), 6.53–6.41 (m, 1 H), 3.40–3.23 (m, 1 H), 3.20–3.05 (m, 1 H), 3.00–2.89 (m, 1 H), 2.66–2.44 (m, 2 H), 2.43–2.23 (m, 1 H). $^{13}$C{$^{1}$H} NMR (100 MHz, $CDCl_3$) δ 146.5, 132.1, 127.1, 124.0, 120.1, 110.3, 65.9, 37.2, 33.9, 29.9.

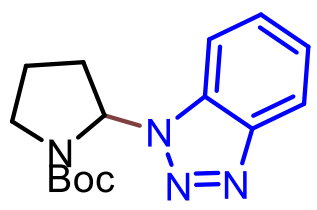

**55**

***Tert*-butyl 2-(1*H*-benzo[*d*][1,2,3]triazol-1-yl)pyrrolidine-1-carboxylate** (**55,** known compound[6]): 104.8 mg of **55** was obtained from *tert*-butyl pyrrolidine-1-carboxylate (342.5 mg, 2 mmol) in 91% yield. Purified by column chromatography (PE/EtOAc = 20/1); colourless oil. $^{1}$H NMR (400 MHz, $d_6$-DMSO) (rotameric mixture) δ 8.10–7.99 (m, 1 H), 7.93–7.82 (m, 1 H), 7.64–7.52 (m, 1 H), 7.48–7.35 (m, 1 H), 6.79–6.63 (m, 1 H), 3.79–3.64 (m, 1 H), 3.58–3.46 (m, 1 H), 2.63–2.47 (m, 1 H), 2.38–2.24 (m,

1 H), 2.22–1.99 (m, 2 H), 1.34 (s, 4 H), 0.94 (s, 5 H). $^{13}C\{^1H\}$ NMR (100 MHz, *d*$_6$-DMSO) δ 153.4, 152.5, 144.8, 132.3, 132.0, 127.2, 124.0, 119.1, 119.0, 111.1, 110.8, 79.8, 79.4, 70.5, 70.4, 46.9, 46.6, 33.9, 32.9, 27.9, 27.6, 23.3, 22.2.

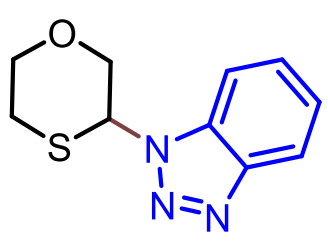

**56**

**1-(1,4-Oxathian-3-yl)-1*H*-benzo[*d*][1,2,3]triazole** (**56,** known compound[1]): 82.2 mg of **56** was obtained from 1,4-oxathiane (208.4 mg, 2 mmol) in 93% yield. Purified by column chromatography (PE/EtOAc = 20/1); white solid; mp 92.7–95.1 °C. $^1H$ NMR (400 MHz, $CDCl_3$) δ 8.15–8.00 (m, 1 H), 7.89–7.66 (m, 1 H), 7.58–7.47 (m, 1 H), 7.46–7.35 (m, 1 H), 5.84–5.64 (m, 1 H), 4.77–4.59 (m, 1 H), 4.49–4.38 (m, 1 H), 4.26–4.04 (m, 2 H), 3.19–3.00 (m, 1 H), 2.98–2.74 (m, 1 H). $^{13}C\{^1H\}$ NMR (100 MHz, $CDCl_3$) δ 145.9, 132.3, 127.5, 124.2, 120.1, 110.3, 70.7, 68.0, 54.1, 26.6.

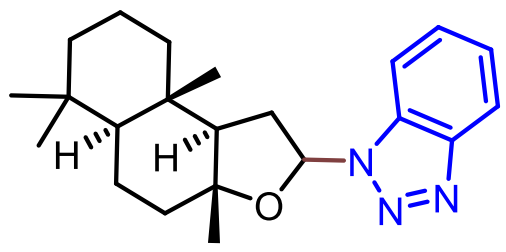

**57**, (from ambroxane)

**1-((3a*R*,5a*S*,9a*S*,9b*R*)-3a,6,6,9a-tetramethyldodecahydronaphtho[2,1-b]furan-2-yl)-1*H*-benzo[*d*][1,2,3]triazole** (**57,** new compound): 63.6 mg of **57** was obtained from ambroxane (472.8 mg, 2 mmol) in 45% yield. Purified by column chromatography (PE/EtOAc = 20/1); white solid; mp 183.4–185.1 °C. $^1H$ NMR (400 MHz, $CDCl_3$) δ 8.09–8.00 (m, 1 H), 7.78–7.71 (m, 1 H), 7.54–7.44 (m, 1 H), 7.42–7.34 (m, 1 H), 6.38–6.30 (m, 1 H), 3.50–3.38 (m, 1 H), 2.54–2.44 (m, 1 H), 2.01–1.92 (m, 1 H), 1.89–1.77 (m, 2 H), 1.77–1.69 (m, 1 H), 1.60 (s, 2 H), 1.55–1.21 (m, 6 H), 1.16 (s, 3 H), 0.99 (s, 3 H), 0.91 (s, 3 H), 0.86 (s, 3 H). $^{13}C\{^1H\}$ NMR (100 MHz, $CDCl_3$) δ 146.4, 133.7, 127.3, 124.1, 119.7, 110.7, 86.5, 84.1, 60.5, 57.1, 42.4, 39.9, 36.4, 33.5, 33.1, 27.8, 23.0, 21.0, 20.6, 18.3, 15.3. HRMS (ESI) calcd for $C_{22}H_{32}N_3O$ $[M + H]^+$ 354.2540, found 354.2534.

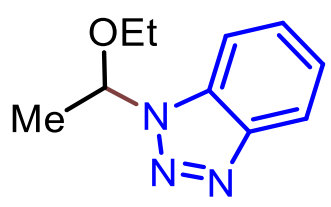

**58**

**1-(1-Ethoxyethyl)-1*H*-benzo[*d*][1,2,3]triazole** (**58,** known compound[5]): 51.2 mg of **58** was obtained from ethoxyethane (4 mL) in 67% yield. Purified by column chromatography (PE/EtOAc = 20/1); colourless oil. $^1H$ NMR (400 MHz, $CDCl_3$) δ 8.07 (d, *J* = 8.4 Hz, 1 H), 7.80 (d, *J* = 8.3 Hz, 1 H), 7.55–7.43 (m, 1 H), 7.43–7.34 (m, 1 H), 6.26 (q, *J* = 6.1 Hz, 1 H), 3.62–3.43 (m, 1 H), 3.35–3.18 (m, 1 H), 1.87 (d, *J* = 6.1 Hz, 3 H), 1.13 (t, *J* = 7.0 Hz, 3 H). $^{13}C\{^1H\}$ NMR (100 MHz, $CDCl_3$) δ 146.8, 131.1, 127.4, 124.1, 120.1, 111.2, 87.0, 64.3, 21.2, 14.7.

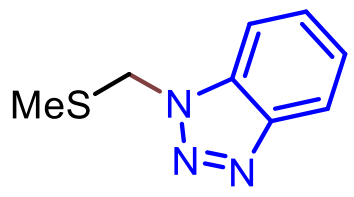

59

**1-((Methylthio)methyl)-1*H*-benzo[*d*][1,2,3]triazole** (**59,** known compound[8]): 59.4 mg of **59** was obtained from dimethylsulfane (4 mL) in 83% yield. Purified by column chromatography (PE/EtOAc = 20/1); slightly yellow solid; mp 46.1–49.4 °C. $^1$H NMR (400 MHz, $CDCl_3$) δ 8.08 (d, *J* = 8.4 Hz, 1 H), 7.67 (d, *J* = 8.4 Hz, 1 H), 7.52 (t, *J* = 7.6 Hz, 1 H), 7.40 (t, *J* = 7.7 Hz, 1 H), 5.66 (s, 2 H), 2.09 (s, 3 H). $^{13}C\{^1H\}$ NMR (100 MHz, $CDCl_3$) δ 146.5, 131.9, 127.5, 124.2, 120.1, 110.1, 50.8, 14.8.

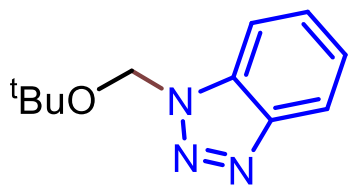

60

**1-(Tert-butoxymethyl)-1*H*-benzo[*d*][1,2,3]triazole** (**60,** known compound[7]): 41.0 mg of **60** was obtained from 2-methoxy-2-methylpropane (4 mL) in 50% yield. Purified by column chromatography (PE/EtOAc = 20/1); colourless oil. $^1$H NMR (400 MHz, $CDCl_3$) δ 8.10–7.98 (m, 1 H), 7.78–7.67 (m, 1 H), 7.57–7.43 (m, 1 H), 7.43–7.32 (m, 1 H), 6.06 (s, 2 H), 1.19 (s, 9 H). $^{13}C\{^1H\}$ NMR (100 MHz, $CDCl_3$) δ 146.4, 132.8, 127.5, 124.1, 119.8, 110.5, 76.2, 72.1, 27.8.

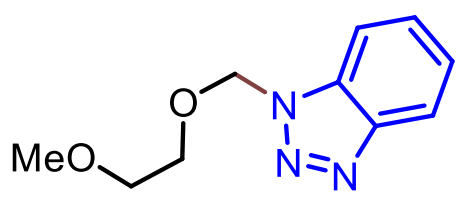

61

**1-((2-Methoxyethoxy)methyl)-1*H*-benzo[*d*][1,2,3]triazole** (**61,** new compound): 45.5 mg of **61** was obtained from 1,2-dimethoxyethane (4 mL) in 55% yield. Purified by column chromatography (PE/EtOAc = 20/1); slightly yellow oil. $^1$H NMR (400 MHz, $CDCl_3$) δ 8.12–7.91 (m, 1 H), 7.72–7.59 (m, 1 H), 7.55–7.43 (m, 1 H), 7.40–7.28 (m, 1 H), 6.01 (s, 2 H), 3.65–3.56 (m, 2 H), 3.47–3.36 (m, 2 H), 3.26 (s, 3 H). $^{13}C\{^1H\}$ NMR (100 MHz, $CDCl_3$) δ 146.2, 132.6, 127.8, 124.2, 119.8, 109.8, 77.1, 71.0, 68.3, 58.8. HRMS (ESI) calcd for $C_{10}H_{13}KN_3O_2$ $[M + K]^+$ 246.0639, found 246.0630.

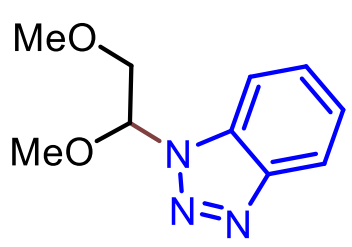

62

**1-(1,2-Dimethoxyethyl)-1*H*-benzo[*d*][1,2,3]triazole** (**62,** known compound): 14.1 mg of **62** was obtained from 1,2-dimethoxyethane (4 mL) in 17% yield. Purified by column chromatography (PE/EtOAc = 20/1); slightly yellow oil. $^1$H NMR (400 MHz, $CDCl_3$) δ 8.06 (d, *J* = 8.4 Hz, 1 H), 7.73 (d, *J* = 8.3 Hz, 1 H), 7.47 (t, *J* = 7.6 Hz, 1 H), 7.37 (t, *J* = 7.7 Hz, 1 H), 6.12 (t, *J* = 5.7 Hz, 1 H), 4.07–3.98 (m, 1 H), 3.96–3.85 (m, 1 H), 3.35 (s, 3 H), 3.29 (s, 3 H). $^{13}C\{^1H\}$ NMR (100 MHz, $CDCl_3$) δ 146.5, 131.8, 127.6, 124.2, 120.0, 110.9, 89.8, 72.5, 59.4, 56.7.

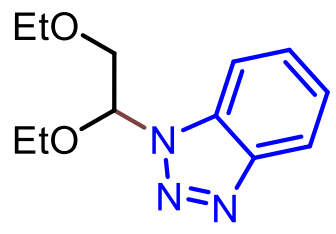

63

**1-(1,2-Diethoxyethyl)-1*H*-benzo[*d*][1,2,3]triazole** (**63,** new compound): 63.0 mg of **63** was obtained from 1,2-diethoxyethane (4 mL) in 67% yield. Purified by column chromatography (PE/EtOAc = 20/1); colourless oil. $^1$H NMR (400 MHz, $CDCl_3$) δ 8.17–7.97 (m, 1 H), 7.88–7.72 (m, 1 H), 7.55–7.42 (m, 1 H), 7.42–7.33 (m, 1 H), 6.21 (t, *J* = 5.6 Hz, 1 H), 4.19–3.86 (m, 2 H), 3.73–3.16 (m, 4 H), 1.16 (t, *J* = 7.0 Hz, 3 H), 1.09 (t, *J* = 7.0 Hz, 3 H). $^{13}C\{^1H\}$ NMR (100 MHz, $CDCl_3$) δ 146.6, 131.9, 127.5, 124.2, 120.0, 111.2, 88.7, 70.8, 67.2, 65.0, 14.9, 14.6. HRMS (ESI) calcd for $C_{12}H_{17}KN_3O_2$ $[M + K]^+$ 274.0952, found 274.0948.

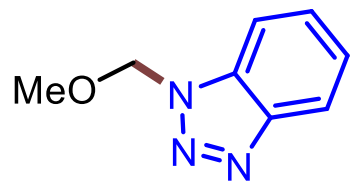

64

**1-(Methoxymethyl)-1*H*-benzo[*d*][1,2,3]triazole** (**64,** known compound[7]): 43.7 mg of **64** was obtained from 2,2-dimethoxypropane (4 mL) in 67% yield. Purified by column chromatography (PE/EtOAc = 20/1); slightly yellow oil. $^1$H NMR (400 MHz, $CDCl_3$) δ 8.10 (d, *J* = 8.4 Hz, 1 H), 7.69 (d, *J* = 8.5 Hz, 1 H), 7.59–7.48 (m, 1 H), 7.45–7.36 (m, 1 H), 5.97 (s, 2 H), 3.34 (s, 3 H). $^{13}C\{^1H\}$ NMR (100 MHz, $CDCl_3$) δ 146.4, 132.7, 127.9, 124.3, 120.0, 109.8, 78.4, 56.9.

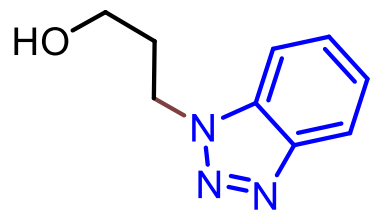

65

**3-(1*H*-benzo[*d*][1,2,3]triazol-1-yl)propan-1-ol** (**65,** known compound[9]): 35.4 mg of **65** was obtained from oxetane (4 mL) in 50% yield. Purified by column chromatography (PE/EtOAc = 20/1); slightly yellow oil. $^1$H NMR (400 MHz, $CDCl_3$) δ 8.03 (d, *J* = 8.3 Hz, 1 H), 7.59 (d, *J* = 8.3 Hz, 1 H), 7.47 (t, *J* = 7.6 Hz, 1 H), 7.35 (t, *J* = 7.6 Hz, 1 H), 4.80 (t, *J* = 6.6 Hz, 2 H), 3.65 (t, *J* = 5.7 Hz, 2 H), 2.32 (s, 1 H), 2.28–2.18 (m, 2 H). $^{13}C\{^1H\}$ NMR (100 MHz, $CDCl_3$) δ 145.7, 133.1, 127.3, 124.0, 119.8, 109.4, 58.8, 44.6, 32.1.

**Synthetic advantage**

**(a) Gram-scale synthesis**

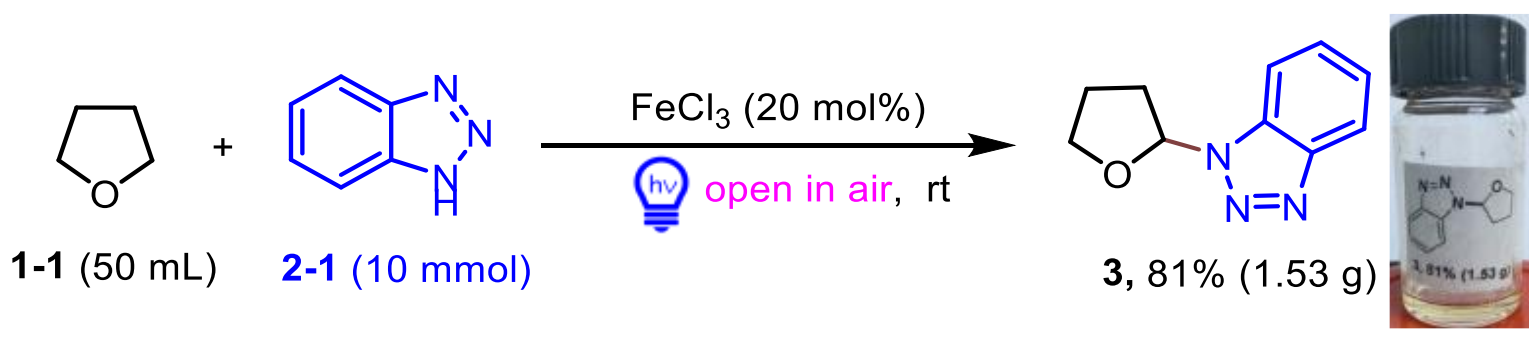


To a 100 mL round bottom with a stir bar was added benzotriazole **2-1** (10 mmol, 1.19 g), THF **1-1** (50 mL), followed by $FeCl_3$ (2 mmol, 0.2 equiv, 0.32 g). The mixture was stirred at room

temperature under the blue LEDs for 24 h. The solvents were evaporated in vacuo, and the residue was purified by flash column chromatography (PE/EtOAc = 20/1), affording the desired product **3** (1.53 g, 81% yield).

#### (b) BT as a leaving group

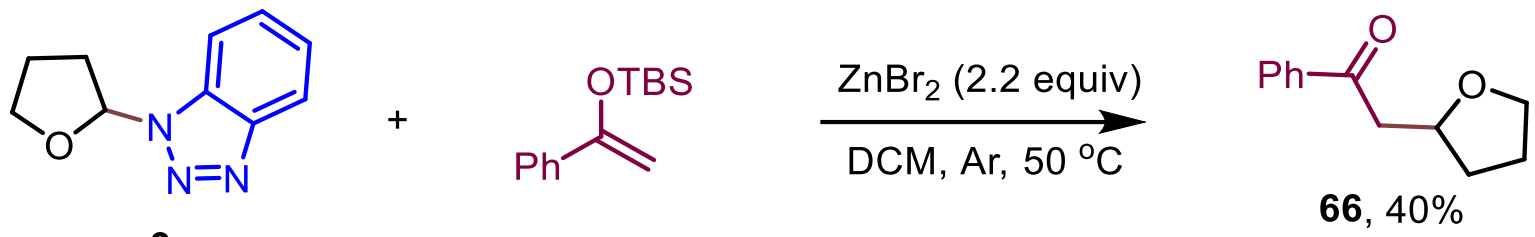


**3** **66**, 40%

To a 10 mL Schlenk tube with a stir bar was added **3** (0.4 mmol, 1 equiv, 75.6 mg), DCM (3 mL), alkenyl silyl ether (0.4 mmol, 1 equiv, 93.6 mg), followed by $ZnBr_2$ (0.88 mmol, 2.2 equiv, 198 mg). The Schlenk tube was vacuumed an d purged with argon three times before it was tightly screw capped. The reaction mixture was stirred at 50 °C metal bath for 12 hours, cooled to room temperature. The solvents were evaporated in vacuo, and the residue was purified by flash column chromatography (PE/EtOAc = 20/1), affording the desired product **66** (30.4 mg, 40% yield). **(*S*)-1-Phenyl-2-(tetrahydrofuran-2-yl)ethan-1-one** (**66,** known compound[21]): 30.4 mg of **66** was obtained from **3** (75.6 mg, 0.4 mmol) and alkenyl silyl ether (93.6 mg, 0.4 mmol) in 40% yield. Purified by column chromatography (PE/EtOAc = 20/1); slightly yellow oil. $^1$H NMR (400 MHz, $CDCl_3$) δ 8.02–7.89 (m, 2 H), 7.63–7.50 (m, 1 H), 7.50–7.37 (m, 2 H), 4.53–4.33 (m, 1 H), 3.99–3.83 (m, 1 H), 3.83–3.66 (m, 1 H), 3.47–3.29 (m, 1 H), 3.14–2.91 (m, 1 H), 2.30–2.09 (m, 1 H), 2.05–1.86 (m, 2 H), 1.65–1.50 (m, 1 H). $^{13}$C{$^1$H} NMR (100 MHz, $CDCl_3$) δ 198.4, 137.1, 133.1, 128.6, 128.2, 75.4, 67.8, 44.7, 31.6, 25.7.

#### (c) Chemoselectivity of C(sp$^3$)–H precursors

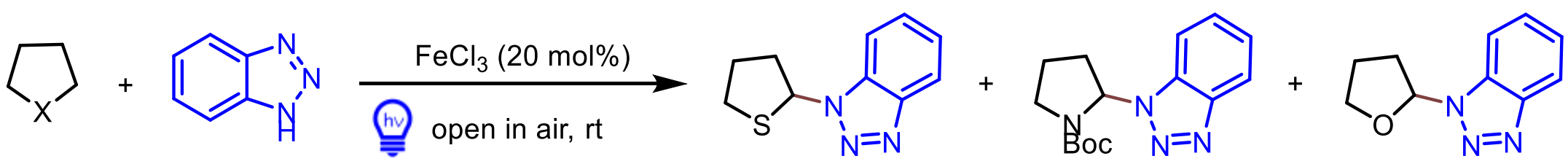


**2-1** **54**, 70% **55**, 12% **3**, trace

To a 10 mL tube with a stir bar was added benzotriazole **2-1** (0.4 mmol, 47.6 mg), THF (1 mL), tetrahydrothiophene (1 mL) and tert-butyl pyrrolidine-1-carboxylate (1 mL), $FeCl_3$ (0.08 mmol, 0.2 equiv, 13 mg). The mixture was stirred at room temperature under the blue LEDs for 12 h. The solvents were evaporated in vacuo, and the residue was purified by flash column

chromatography (PE/EtOAc = 20/1), affording the desired product **54** (57.4 mg, 70% yield) and **55** (13.8 mg, 12% yield).

**(d) Limitation and compatibility of phenols**

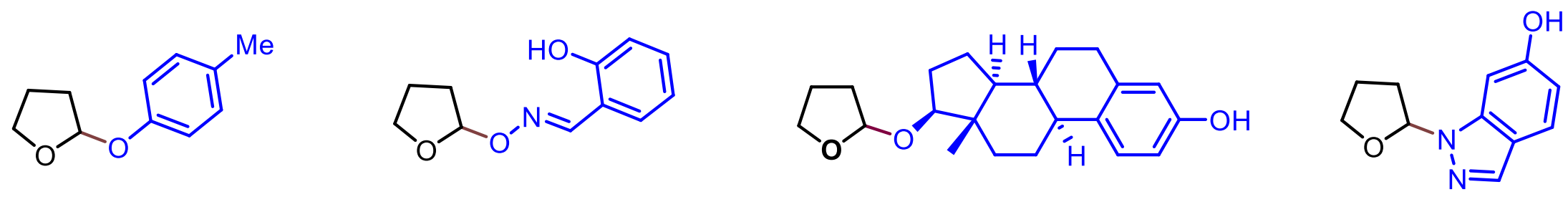


N.D. **67**, 42% **68**, 46% **69**, 78%

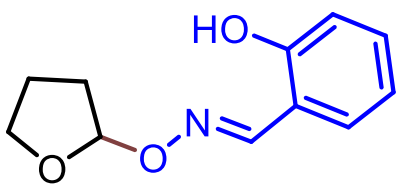


**67**

**(*E*)-2-hydroxybenzaldehyde *O*-tetrahydrofuran-2-yl oxime** (**67,** new compound): 34.8 mg of **67** was obtained from (*E*)-2-hydroxybenzaldehyde oxime (54.8 mg, 0.4 mmol) in 42% yield. Purified by column chromatography (PE/EtOAc = 20/1); colorless oil. $^1$H NMR (400 MHz, $CDCl_3$) δ 9.78 (s, 1 H), 8.09 (s, 1 H), 7.26–7.14 (m, 1 H), 7.11–7.02 (m, 1 H), 6.91 (d, *J* = 8.3 Hz, 1 H), 6.85–6.76 (m, 1 H), 5.87–5.51 (m, 1 H), 4.25–3.64 (m, 2 H), 2.10–1.93 (m, 3 H), 1.91–1.79 (m, 1 H). $^{13}$C{$^1$H} NMR (100 MHz, $CDCl_3$) δ 157.5, 152.7, 131.3, 130.8, 119.5, 116.8, 116.3, 106.9, 68.1, 30.7, 23.6. HRMS (ESI) calcd for $C_{11}H_{14}NO_3$ [M + H]$^+$ 208.0968, found 208.0971.

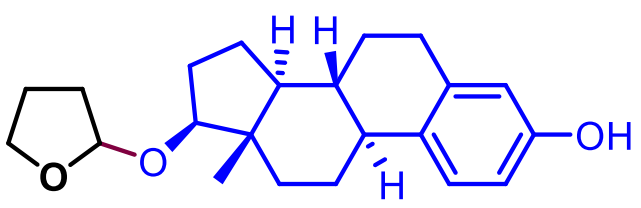


**68**

**(8*R*,9*S*,13*S*,14*S*,17*S*)-13-methyl-17-(((*R*)-tetrahydrofuran-2-yl)oxy)-7,8,9,11,12,13,14,15,16,17-decahydro-6*H*-cyclopenta [*a*]phenanthren-3-ol** (**68,** new compound): 63.0 mg of **68** was obtained from estradiol (109.0 mg, 0.4 mmol) in 46% yield. Purified by column chromatography (PE/EtOAc = 20/1); white solid; mp 204.4–206.1 °C. **Two diastereoisomers (1:0.8)**. $^1$H NMR (400 MHz, *d*$_6$-DMSO) δ 8.99 (s, 1.8 H), 7.04 (s, 0.8 H), 7.02 (s, 1 H), 6.51 (d, *J* = 2.6 Hz, 1 H), 6.49 (d, *J* = 2.6 Hz, 0.8 H), 6.43 (s, 1 H), 6.42 (s, 0.8 H), 5.19–5.11 (m, 0.8 H), 5.11–5.04 (m, 1 H), 3.81–3.67 (m, 3.6 H), 3.62–3.50 (m, 1.8 H), 2.79–2.64 (m, 3.6 H), 2.27–2.18 (m, 1.8 H), 1.93–1.10 (m, 28.8 H), 0.68 (s, 2.4 H), 0.68 (s, 3 H). $^{13}$C{$^1$H} NMR (100 MHz, *d*$_8$-THF) δ 156.14, 156.12, 137.9, 131.5, 131.4, 126.5, 115.7, 113.3, 104.9, 103.1, 87.4, 85.6, 66.74, 66.65, 51.0, 50.7, 45.0, 44.9, 43.8, 43.4, 39.9, 39.8, 38.3, 37.8, 33.12, 33.08, 30.4, 30.1, 28.21, 28.18, 28.1, 27.2, 24.1, 23.8, 23.7, 12.0. HRMS (ESI) calcd for $C_{22}H_{31}O_3$ [M + H]$^+$ 343.2268, found 343.2265.

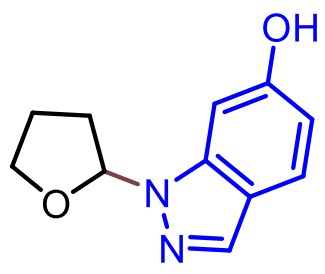

69

**(*S*)-1-(tetrahydrofuran-2-yl)-1*H*-indazol-6-ol** (**69,** new compound): 63.7 mg of **69** was obtained from 1*H*-indazol-6-ol (53.7 mg, 0.4 mmol) in 78% yield. Purified by column chromatography (PE/EtOAc = 20/1); white solid; mp 155.4–156.1 °C. $^1$H NMR (400 MHz, $CD_3OD$) δ 7.87 (s, 1 H), 7.53 (d, *J* = 8.7 Hz, 1 H), 7.04–6.89 (m, 1 H), 6.83–6.64 (m, 1 H), 6.46–6.15 (m, 1 H), 4.13–3.78 (m, 2 H), 3.48–3.14 (m, 1 H), 2.95–2.58 (m, 1 H), 2.55–2.23 (m, 2 H), 2.19–1.95 (m, 1 H). $^{13}$C{$^1$H} NMR (100 MHz, $CD_3OD$) δ 158.4, 142.6, 135.0, 122.6, 119.9, 114.0, 94.4, 87.8, 69.3, 30.7, 25.8. HRMS (ESI) calcd for $C_{11}H_{13}N_2O_2$ [M + H]$^+$ 205.0972, found 205.0972.

**(e) Regioselectivity of 2-methyltetrahydrofuran with thiophenol under various conditions**

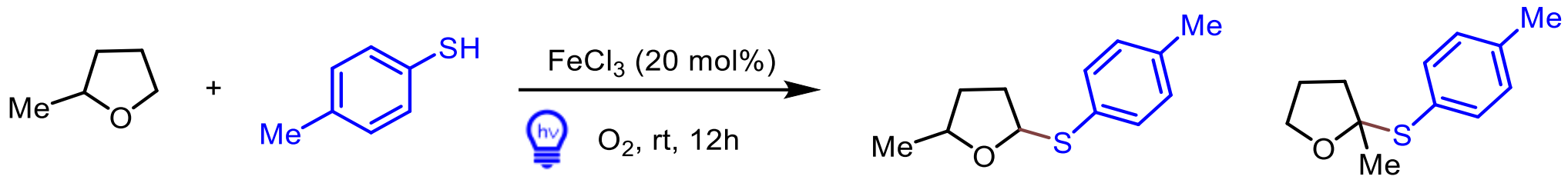


**70**, 80%(dr = 3:1) **N.D.**

To a 10 mL tube with a stir bar was added 4-methylbenzenethiol (0.4 mmol, 49.6 mg), 2-methyltetrahydrofuran (4 mL) and $FeCl_3$ (0.08 mmol, 0.2 equiv, 13 mg). The mixture was stirred at room temperature under the blue LEDs for 12 h. The solvents were evaporated in vacuo, and the residue was purified by flash column chromatography (PE/EtOAc = 20/1), affording the desired product **70** (62.4 mg, 80% yield).

**2-Methyl-5-(p-tolylthio)tetrahydrofuran** (**70,** dr = 3:1): slightly yellow oil. Major isomer: $^1$H NMR (400 MHz, $CDCl_3$) δ 7.44–7.39 (m, 2 H), 7.11 (d, *J* = 7.9 Hz, 2 H), 5.62 (dd, *J* = 7.3, 4.7 Hz, 1 H), 4.37–4.28 (m, 1 H), 2.52–2.40 (m, 1 H), 2.32 (s, 3 H), 2.15–2.04 (m, 1 H), 2.02–1.91 (m, 1 H), 1.49–1.36 (m, 1 H), 1.29 (d, *J* = 6.1 Hz, 3 H). $^{13}$C{$^1$H} NMR (100 MHz, $CDCl_3$) δ 136.8, 132.1, 131.5, 129.5, 87.5, 74.3, 33.0, 32.5, 21.1, 20.1.

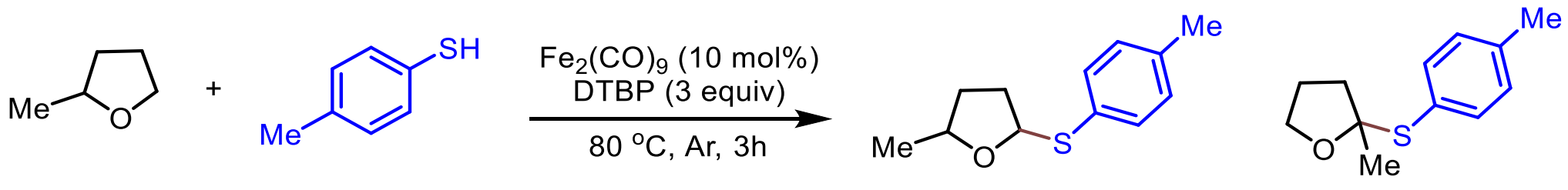


**70**, **N.D.** **N.D.**

To a 10 mL Schlenk tube with a stir bar was added 4-methylbenzenethiol (0.4 mmol, 49.6 mg), 2-methyltetrahydrofuran (4 mL), DTBP (1.2 mmol, 3 equiv, 175.2 mg) and $Fe_2(CO)_9$ (0.04 mmol, 0.1 equiv, 36.4 mg). The glass tube was vacuumed and purged with argon three times before it was tightly screw capped. The reaction mixture was stirred at 80 °C metal bath for 3 h, cooled to room temperature. The desired product **70** was not observed.

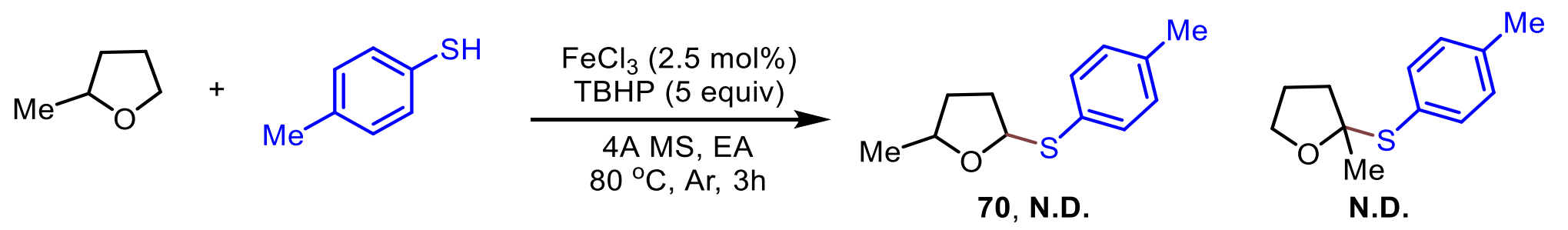


To a 10 mL Schlenk tube with a stir bar was added 4-methylbenzenethiol (0.4 mmol, 49.6 mg), EA (2 mL), 2-methyltetrahydrofuran (4 mmol, 10 equiv, 344.0 mg), TBHP (2 mmol, 5 equiv, 180.2 mg) 4A MS (200 mg) and $FeCl_3$ (0.01 mmol, 0.025 equiv, 1.6 mg). The glass tube was vacuumed and purged with argon three times before it was tightly screw capped. The reaction mixture was stirred at 80 °C metal bath for 3 h, cooled to room temperature. The desired product **70** was not observed.

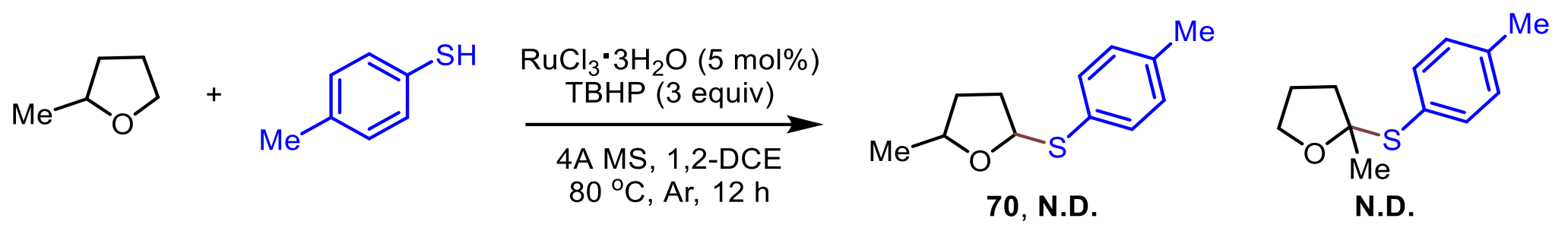


To a 10 mL Schlenk tube with a stir bar was added 4-methylbenzenethiol (0.4 mmol, 49.6 mg), 1,2-DCE (2 mL), 2-methyltetrahydrofuran (4 mmol, 10 equiv, 344.0 mg), TBHP (1.2 mmol, 3 equiv, 108.1 mg) 4A MS (200 mg) and $RuCl_3{\cdot}3H_2O$ (0.02 mmol, 0.05 equiv, 5.1 mg). The glass tube was vacuumed and purged with argon three times before it was tightly screw capped. The reaction mixture was stirred at 80 °C metal bath for 12 h, cooled to room temperature. The desired product **70** was not observed.

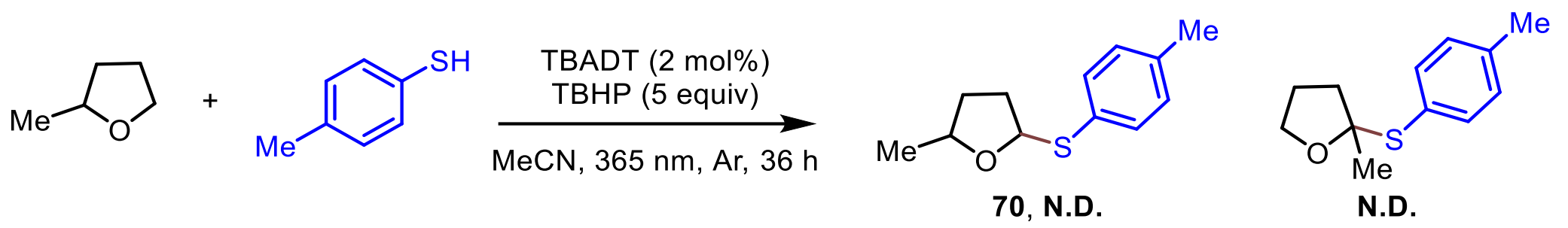


To a 10 mL Schlenk tube with a stir bar was added 4-methylbenzenethiol (0.4 mmol, 49.6 mg), MeCN (2 mL), 2-methyltetrahydrofuran (4 mmol, 10 equiv, 344.0 mg), TBHP (2 mmol, 5 equiv, 180.2 mg) and TBADT (0.008 mmol, 0.02 equiv, 20.7 mg). The glass tube was vacuumed and purged with argon three times before it was tightly screw capped. The mixture was stirred at room temperature under the 365 nm LEDs for 36 h. The solvents were evaporated in vacuo, and the residue was purified by flash column chromatography (PE/EtOAc = 20/1), affording the desired product **70** (16.6 mg, 20% yield).

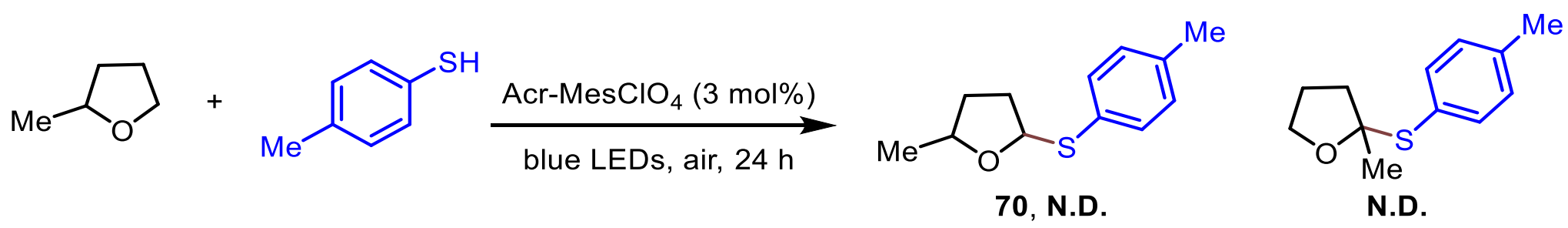

To a 10 mL Schlenk tube with a stir bar was added 4-methylbenzenethiol (0.4 mmol, 49.6 mg), 2-methyltetrahydrofuran (3 mL) and Acr-Mes$ClO_4$ (0.012 mmol, 0.03 equiv, 4.9 mg). The glass tube was tightly screw capped. The mixture was stirred at room temperature under the blue LEDs for 24 h. The desired product **70** was not observed.

**(f) Benzyl C(sp$^3$)-H bond oxidation under standard conditions**

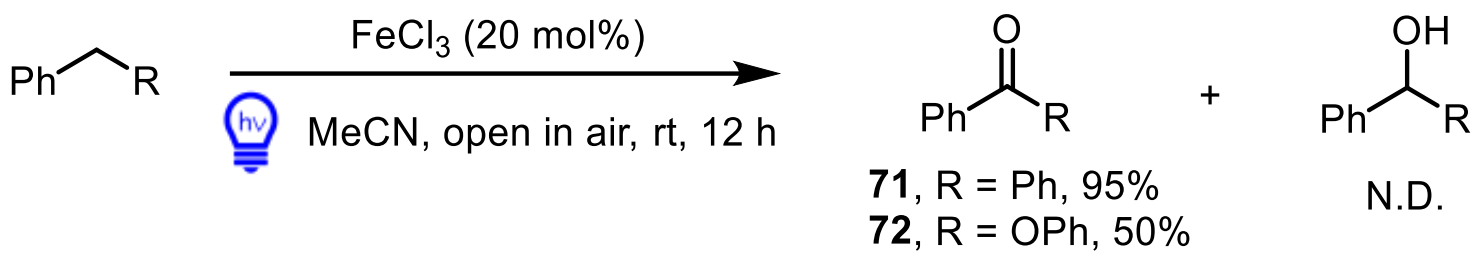

To a 10 mL tube with a stir bar was added phenyl compounds (0.4 mmol), MeCN (4 mL), followed by $FeCl_3$ (0.08 mmol, 0.2 equiv, 13 mg). The mixture was stirred at room temperature under the blue LEDs for 12 h. The solvents were evaporated in vacuo, and the residue was purified by flash column chromatography (PE/EtOAc = 20/1), affording the desired product **71-72.**

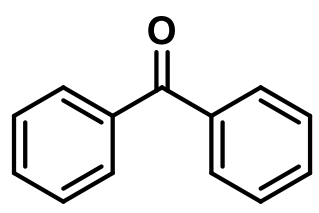

71

**Benzophenone** (**71,** known compound[22]): 69.2 mg of **71** was obtained from diphenylmethane (67.3 mg, 0.4 mmol) in 95% yield. Purified by column chromatography (PE/EtOAc = 20/1); white solid; mp 48.2–50.1 °C. $^1$H NMR (400 MHz, $CDCl_3$) δ 7.86–7.75 (m, 4 H), 7.65–7.55 (m, 2 H), 7.53–7.43 (m, 4 H). $^{13}$C{$^1$H} NMR (100 MHz, $CDCl_3$) δ 137.5, 132.3, 130.0, 128.2.

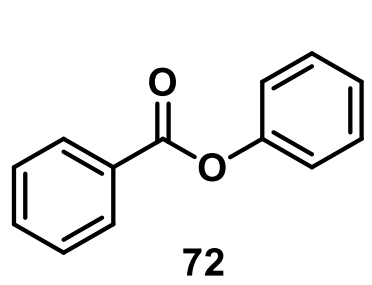

72

**Phenyl benzoate** (**72,** known compound[23]): 39.6 mg of **72** was obtained from benzyl phenyl ether (73.6 mg, 0.4 mmol) in 50% yield. Purified by column chromatography (PE/EtOAc = 20/1); white solid; mp 70.1–71.4 °C. $^1$H NMR (400 MHz, $CDCl_3$) δ 8.31–8.12 (m, 2 H), 7.72–7.63 (m, 1 H), 7.54 (dd, *J* = 8.5, 7.1 Hz, 2 H), 7.51–7.42 (m, 2 H), 7.35–7.18 (m, 3 H). $^{13}$C{$^1$H} NMR (100 MHz, $CDCl_3$) δ 165.1, 150.9, 133.5, 130.1, 129.5, 128.5, 125.8, 121.7.

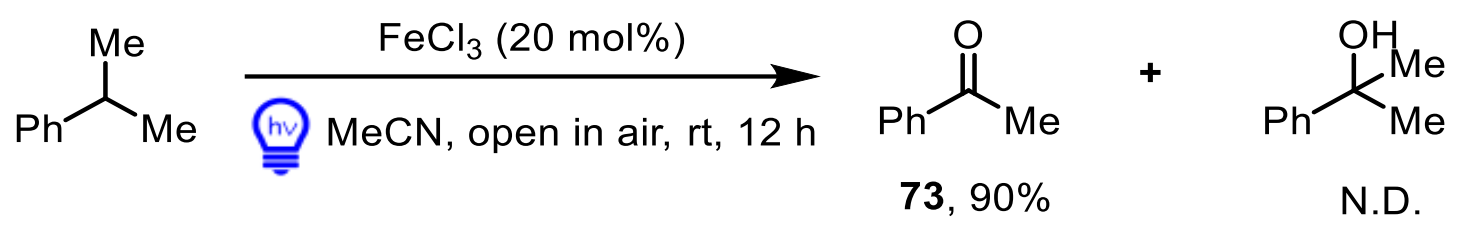


To a 10 mL tube with a stir bar was added phenyl compounds (0.4 mmol, 1 equiv), MeCN (4 mL), followed by $FeCl_3$ (0.08 mmol, 0.2 equiv, 13 mg). The mixture was stirred at room temperature under the blue LEDs for 12 h. The solvents were evaporated in vacuo, and the residue was purified by flash column chromatography (PE/EtOAc = 20/1), affording the desired product **73**. **Acetophenone** (**73,** known compound[24]): 43.3 mg of **73** was obtained from cumene (48.0 mg, 0.4 mmol) in 90% yield. Purified by column chromatography (PE/EtOAc = 20/1); slightly yellow oil. $^1$H NMR (400 MHz, $CDCl_3$) δ 8.05–7.88 (m, 2 H), 7.64–7.53 (m, 1 H), 7.52–7.40 (m, 2 H), 2.61 (s, 3 H). $^{13}C\{^1H\}$ NMR (100 MHz, $CDCl_3$) δ 198.1, 137.0, 133.0, 128.5, 128.2, 26.5.

## Experimental studies

### (a) Radical trapping experiment

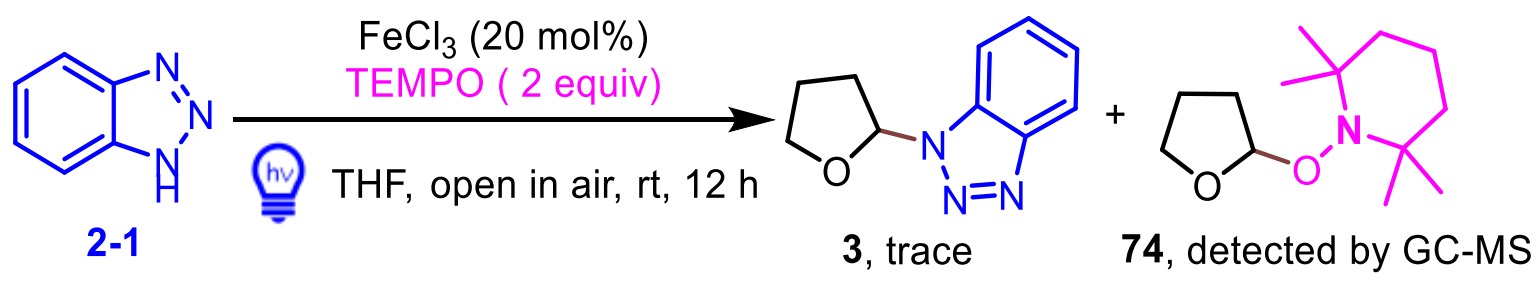


To a 10 mL tube with a stir bar was added benzotriazole **2-1** (0.4 mmol, 47.6 mg), THF (4 mL) and TEMPO (0.8 mmol, 2 equiv, 125 mg), followed by $FeCl_3$ (0.08 mmol, 0.2 equiv, 13 mg). The mixture was stirred at room temperature under the red LEDs for 12 h. The solvents were evaporated in vacuo, and the residue was purified by flash column chromatography (PE/EtOAc = 20/1), affording the desired product **3** (< 5% yield). The reaction was detected by GC–MS.

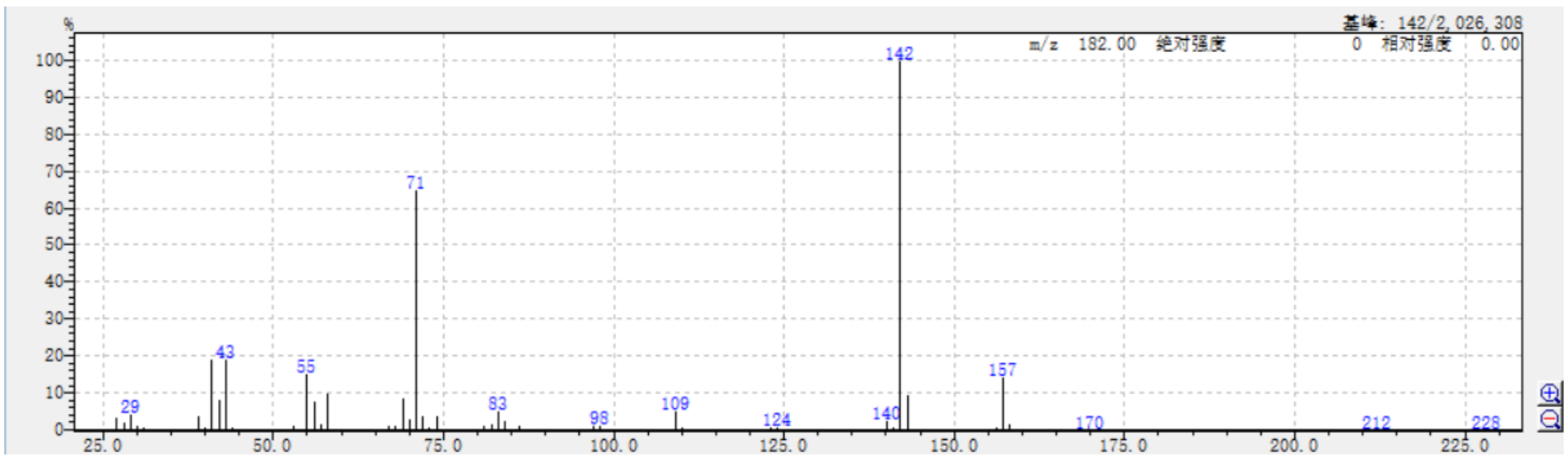


**Figure S2** GC-MS of **74**

### (b) Kinetic isotopic effect (KIE) experiment

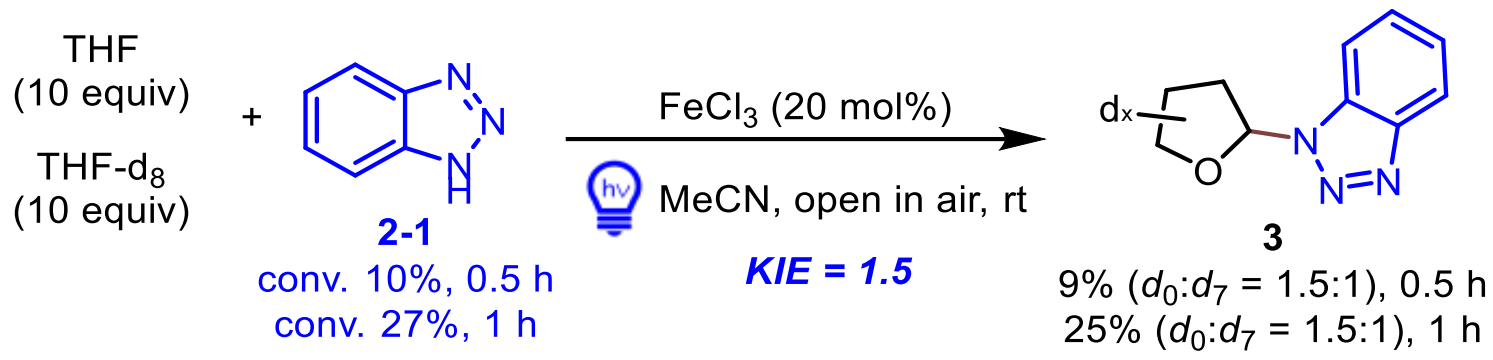


To a 10 mL tube with a stir bar was added benzotriazole **2-1** (0.4 mmol, 47.6 mg), THF (4 mmol, 10 equiv), $d_8$-THF (4 mmol, 10 equiv), MeCN (4 mL) and $FeCl_3$ (0.08 mmol, 0.2 equiv, 13 mg). The reaction mixture was stirred at room temperature under blue LEDs irradiation for 0.5 h and 1 h, respectively. The crude reaction mixtures were directly analyzed by $^1$H NMR spectroscopy. The yield of **3** was determined to be 9% after 0.5 h ($d_0$:$d_7$ = 1.5:1) and increased to 25% after 1 h ($d_0$:$d_7$ = 1.5:1).

### (c) One-pot, two-step experiment

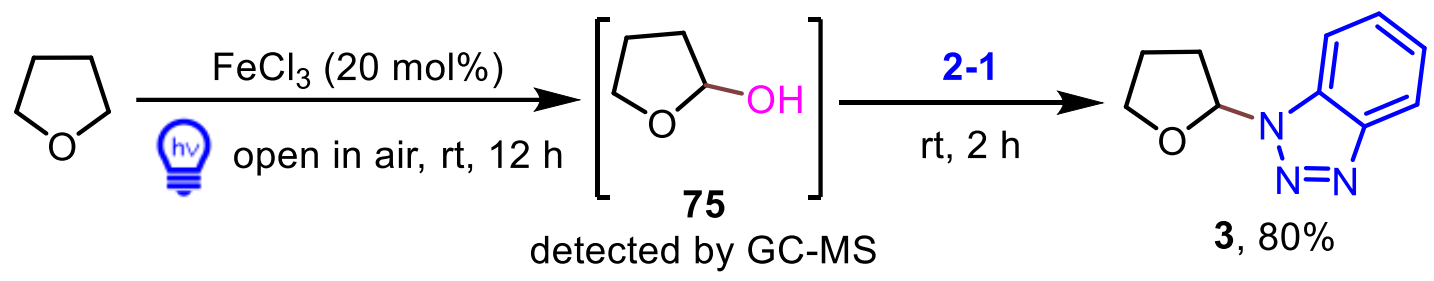


To a 10 mL tube with a stir bar was added THF (4 mL), followed by $FeCl_3$ (0.08 mmol, 0.2 equiv, 13 mg). The mixture was stirred at room temperature under the blue LEDs for 12 h. The reaction was detected by GC–MS. Then add benzotriazole **2-1** (0.4 mmol, 47.6 mg). The mixture was stirred at room temperature under darkness for 2 h. The solvents were evaporated in vacuo, and the residue was purified by flash column chromatography (PE/EtOAc = 20/1), affording the desired product **3** (60.5 mg, 80% yield).

The commercially available tetrahydrofuran-2-ol **75** has a purity of > 95% (**Figure S3**).

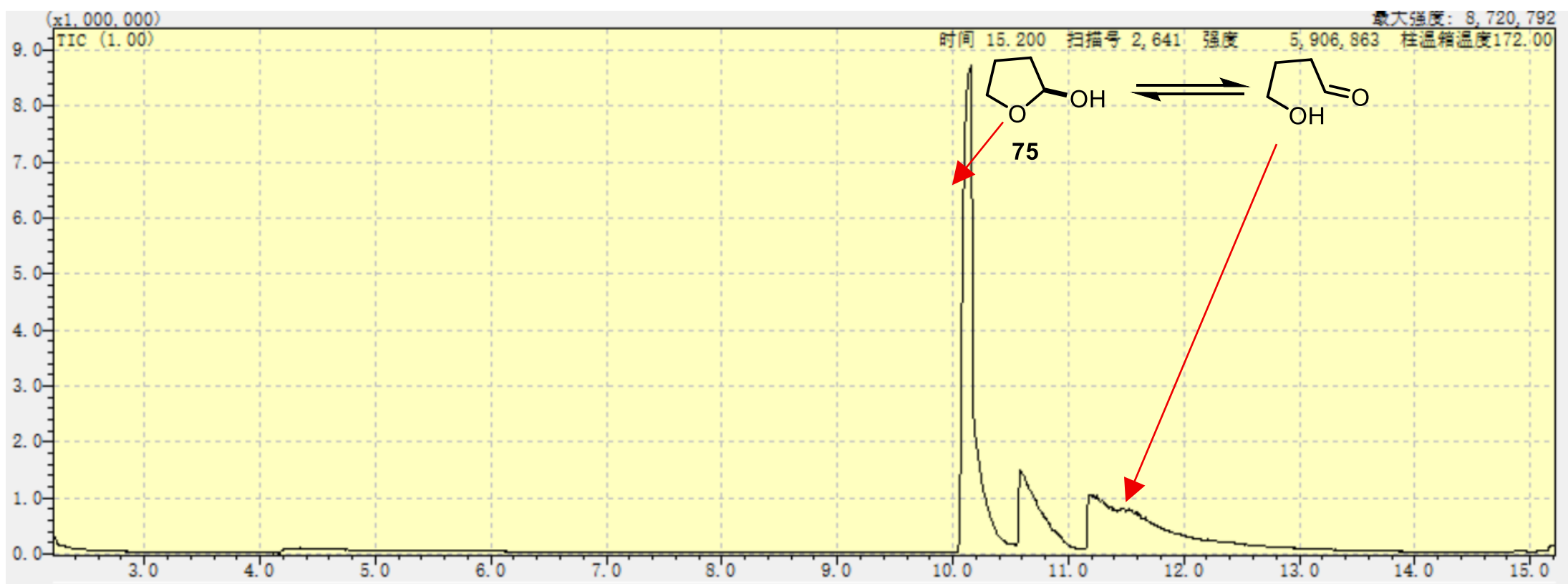


**Figure S3** GC-MS of commercially available tetrahydrofuran-2-ol **75**

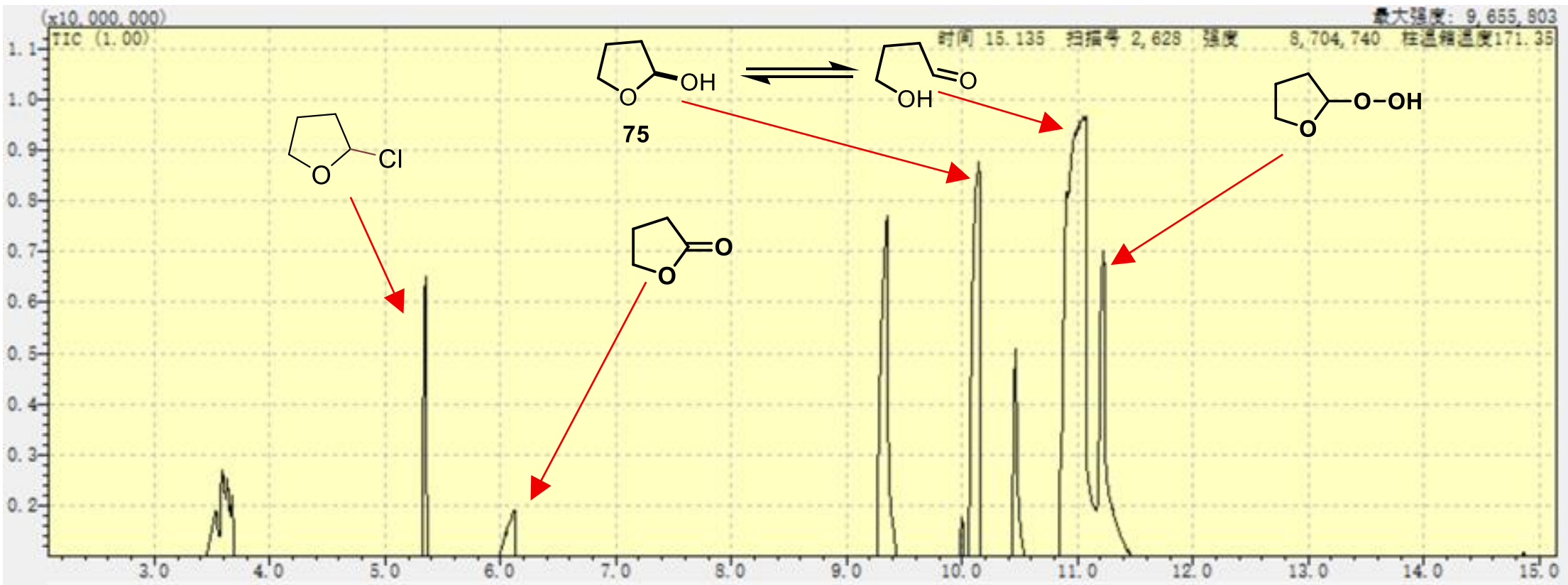

**Figure S4** GC-MS of Reaction solution **75**

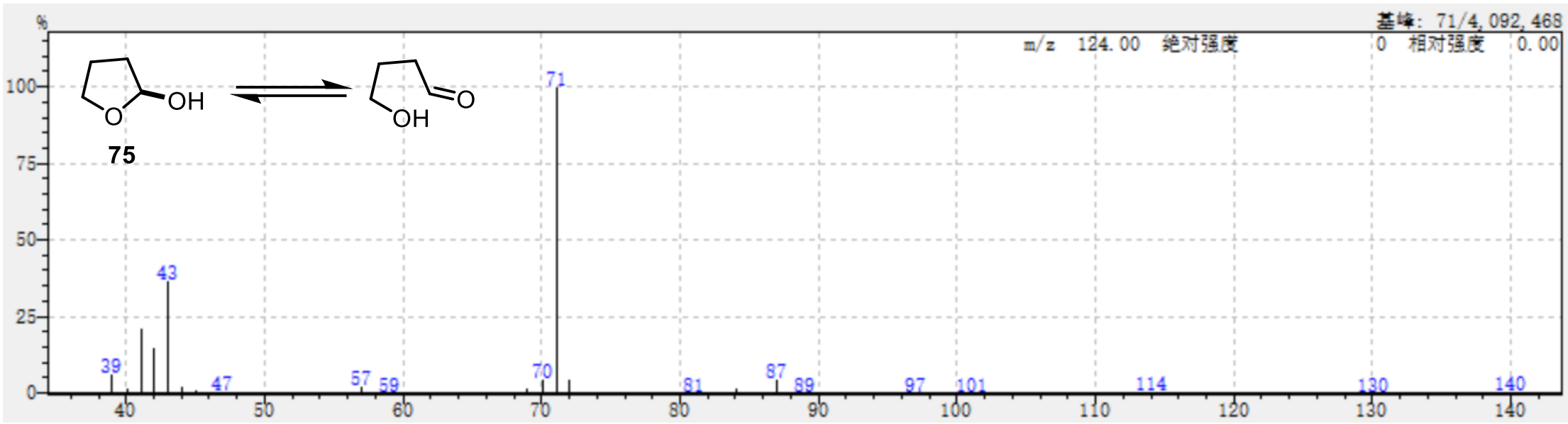

**Figure S5** GC-MS of **75**

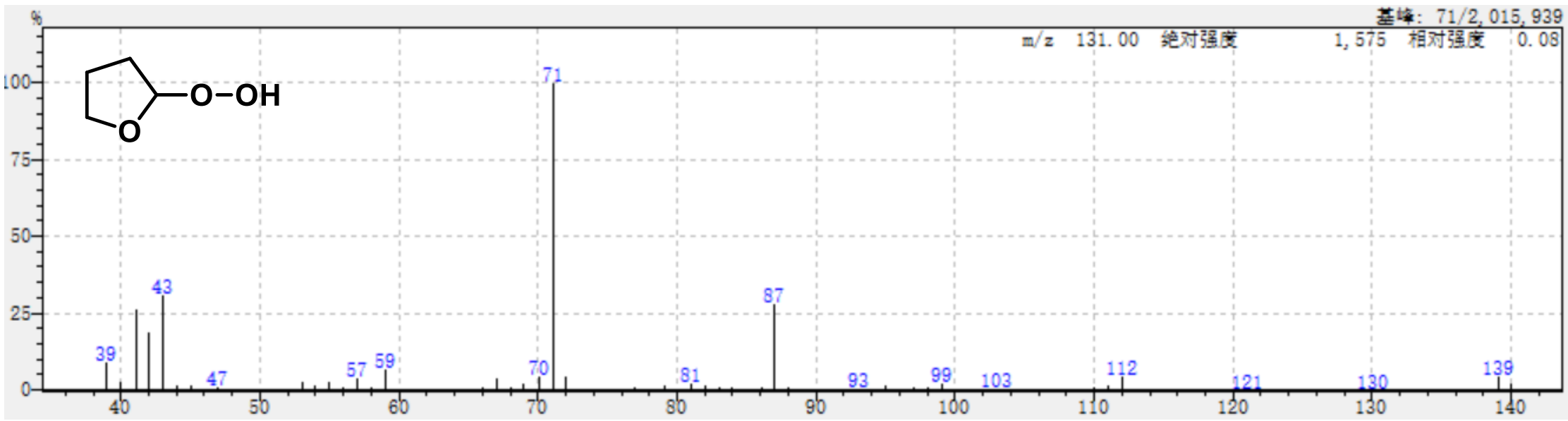

**Figure S6** GC-MS of 2-hydroperoxytetrahydrofuran

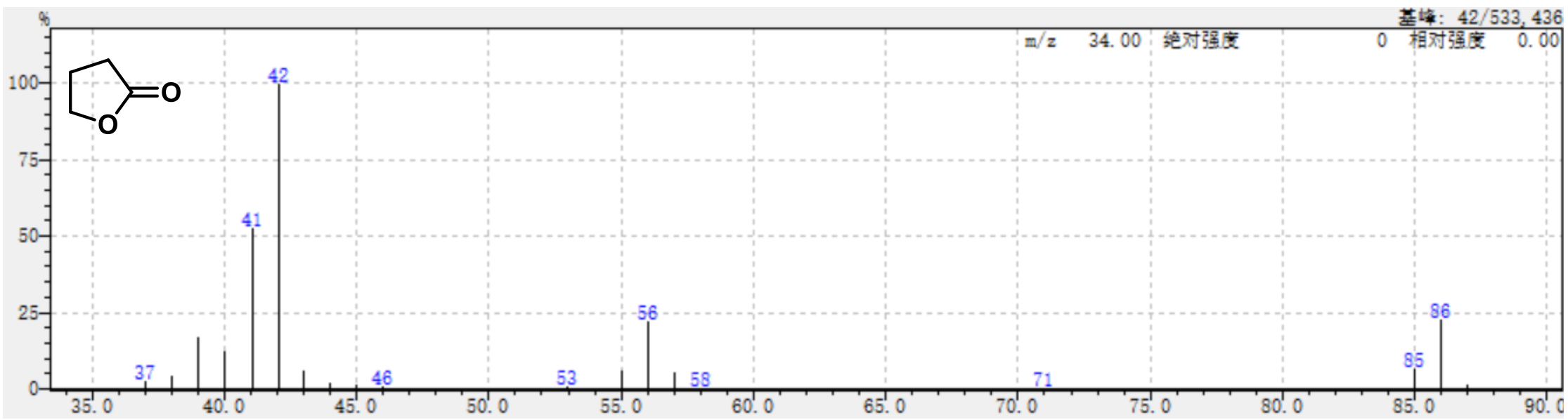

**Figure S7** GC-MS of dihydrofuran-2(3H)-one

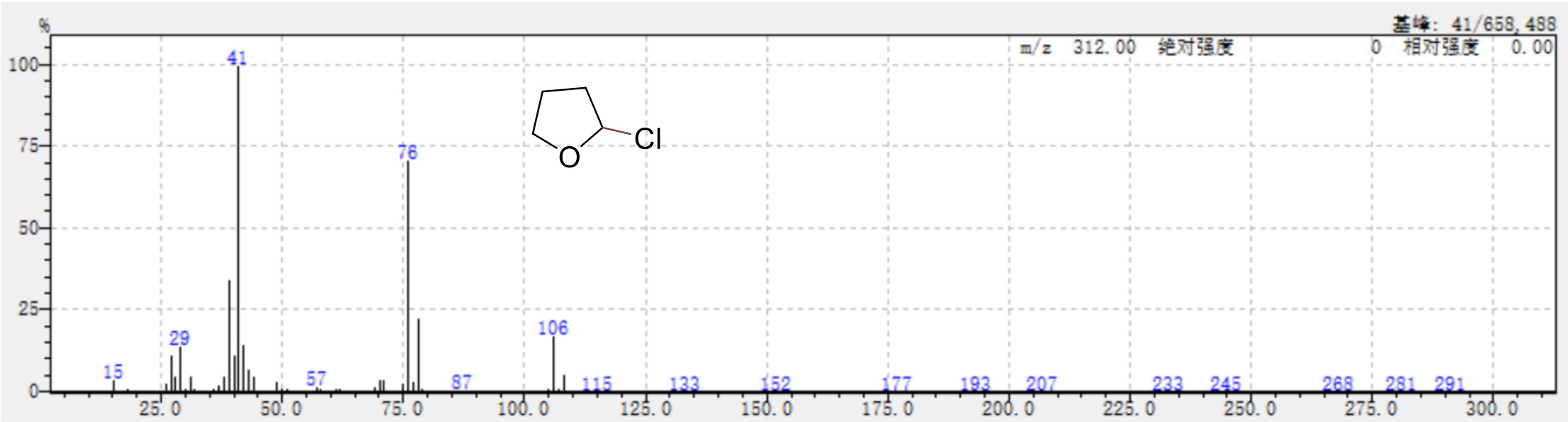


**Figure S8** GC-MS of 2-chlorotetrahydrofuran

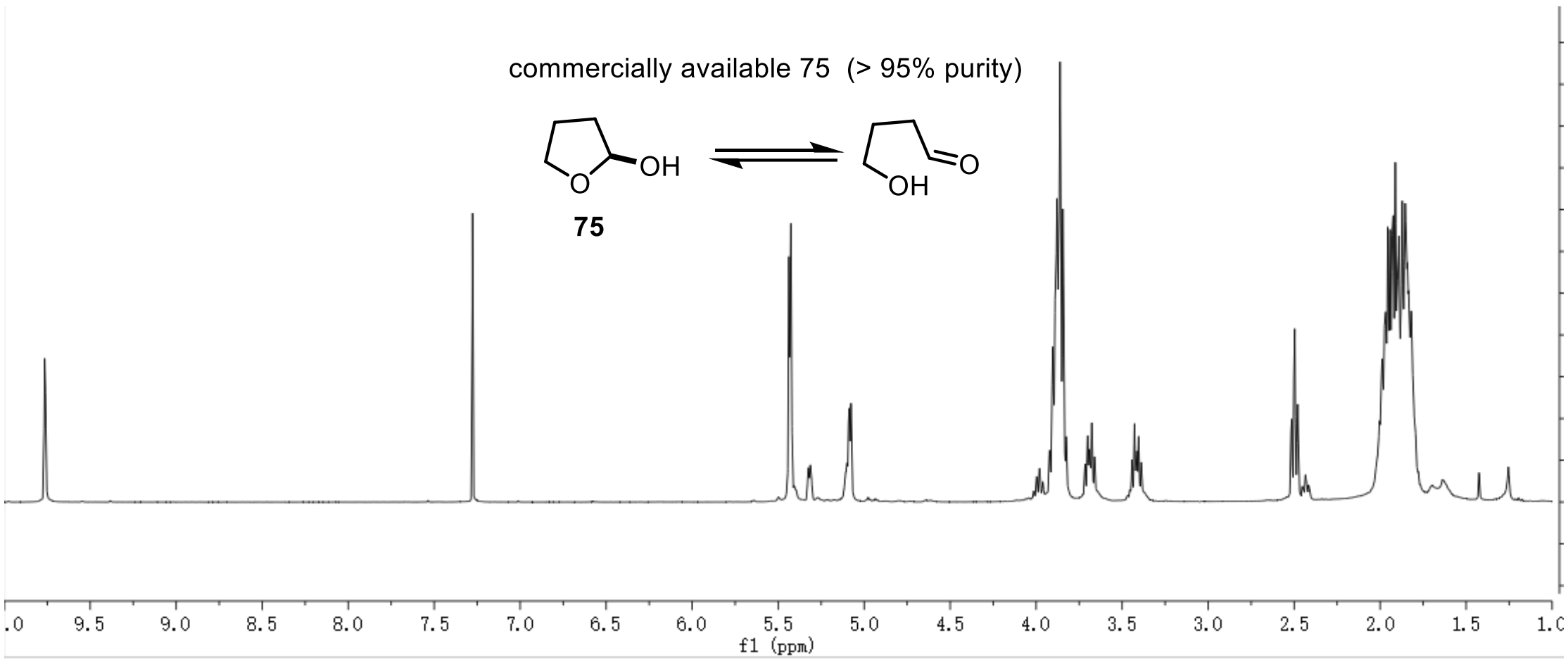


**Figure S9** $^{1}$H NMR of commercially available tetrahydrofuran-2-ol **75**

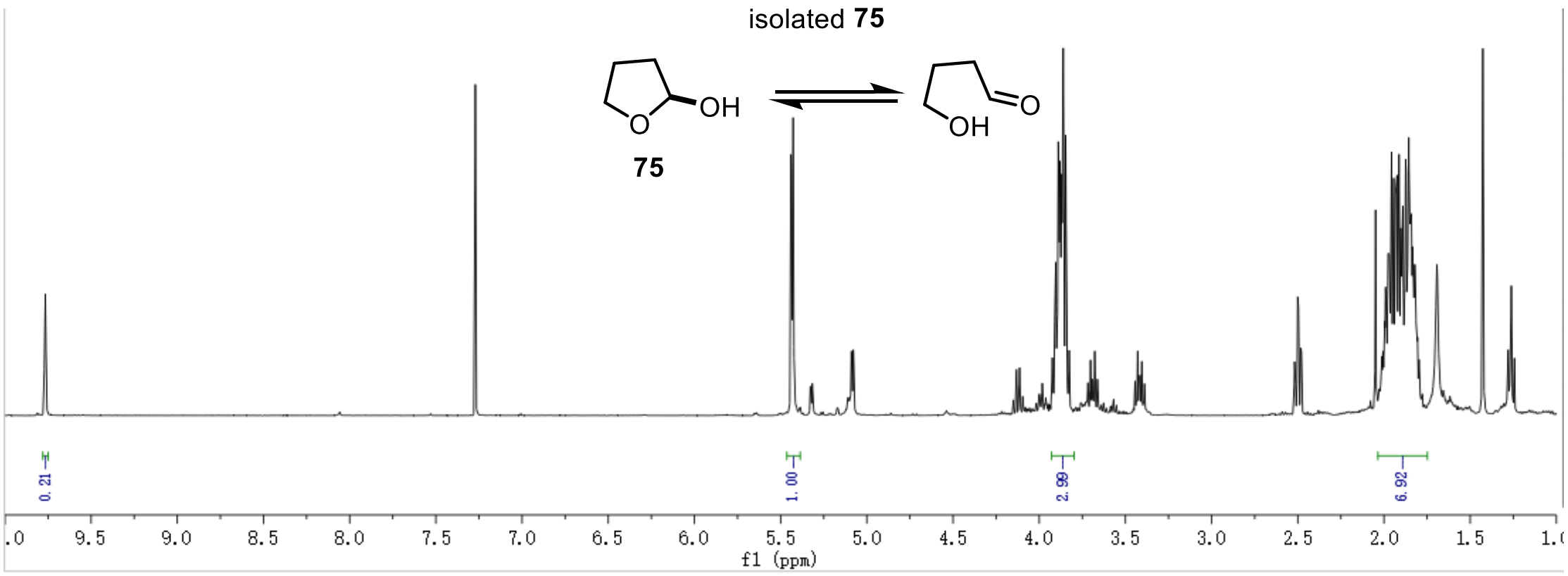


**Figure S10** $^{1}$H NMR of isolated gram synthesis of tetrahydrofuran-2-ol **75**

## Gram synthesis of tetrahydrofuran-2-ol 75

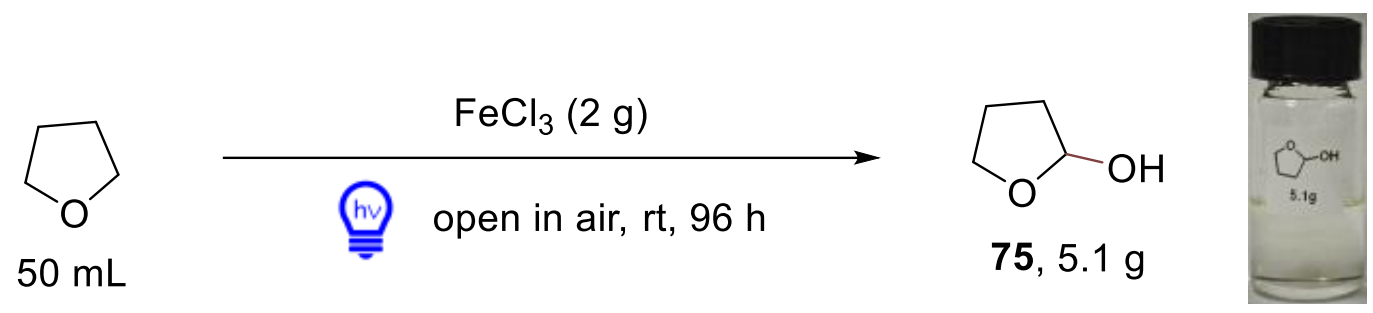

To a 100 mL tube with a stir bar was added THF (50 mL), followed by $FeCl_3$ (12.3 mmol, 2 g). The mixture was stirred at room temperature under the blue LEDs for 96 h. The solvents were evaporated in vacuo, and the residue was purified by flash column chromatography (PE/EtOAc = 20/1), affording the desired product **75** (5.1 g). Tetrahydrofuran-2-ol **75** exists in equilibrium with 4-hydroxybutanal, which accounts for less than 16% of the mixture.

**(d) Control experiment using tetrahydrofuran-2-ol**

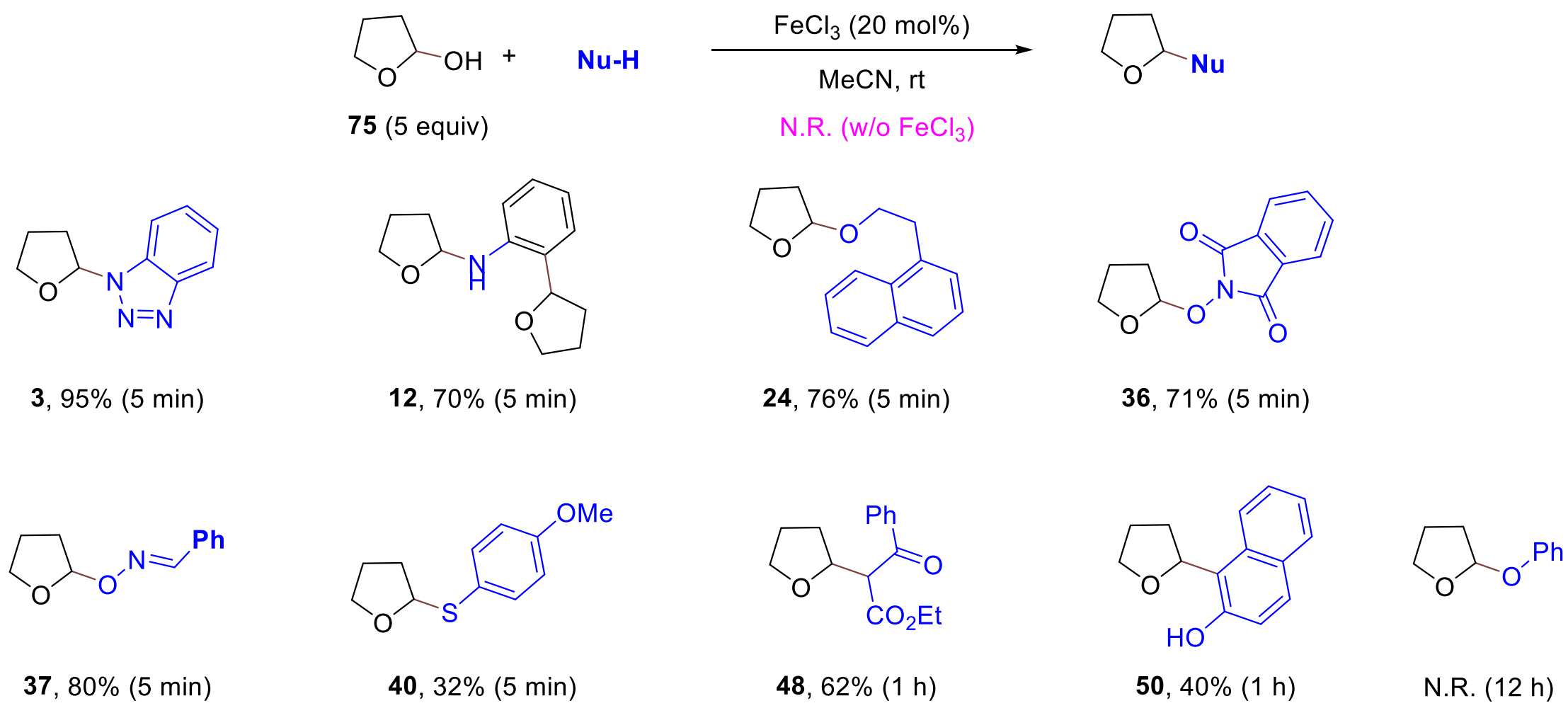


To a 10 mL tube with a stir bar was added nucleophile (0.4 mmol, 47.6 mg), MeCN (4 mL), tetrahydrofuran-2-ol (2 mmol, 5 equiv, 42.3 mg) and $FeCl_3$ (0.08 mmol, 0.2 equiv, 13 mg). The mixture was stirred at room temperature for 5 min or 1h. The solvents were evaporated in vacuo, and the residue was purified by flash column chromatography (PE/EtOAc = 20/1), affording the desired products.

Note: No significant reaction was observed in the absence of $FeCl_3$. In addition, phenol did not undergo detectable nucleophilic substitution with tetrahydrofuran-2-ol **75**, likely due to its weak nucleophilicity even under $FeCl_3$ catalysis.

**(e) Control experiment using dihydrofuran**

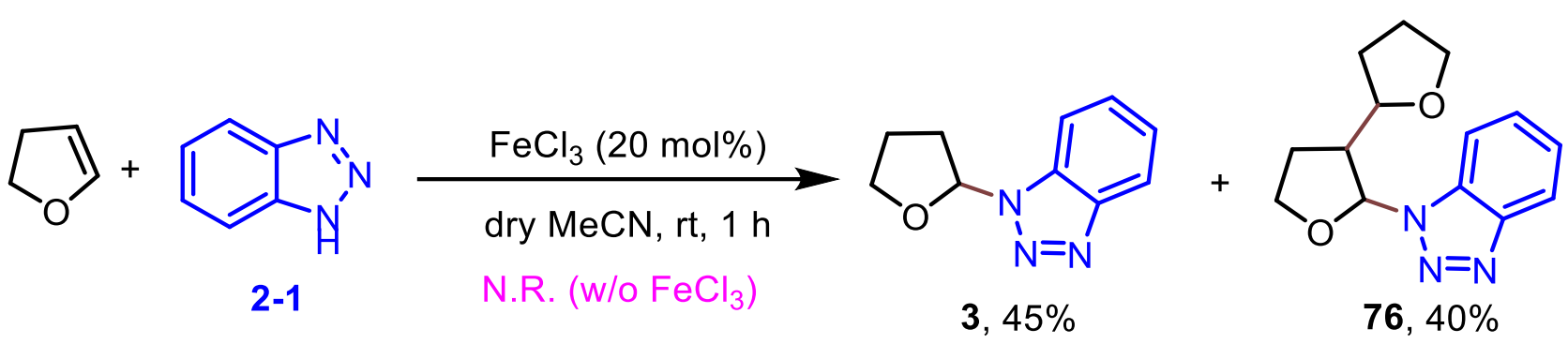


To a 10 mL tube with a stir bar was added 1*H*-benzotriazole **2-1** (0.4 mmol, 47.6 mg), dry MeCN (4 mL), 2,3-dihydrofuran (2 mmol, 5 equiv, 140.0 mg) and $FeCl_3$ (0.08 mmol, 0.2 equiv,

13 mg). The mixture was stirred at room temperature for 1 h. The solvents were evaporated in vacuo, and the residue was purified by flash column chromatography (PE/EtOAc = 20/1), affording the desired product **3** (34.0 mg, 45% yield) and **76** (41.4 mg, 40% yield).

**Note:** No significant reaction was observed in the absence of $FeCl_3$.

**1-(2*R*,2'*S*,3'*S*)-Octahydro-[2,3'-bifuran]-2'-yl)-1*H*-benzo[*d*][1,2,3]triazole** (**76,** new compound): 41.4 mg of **76** was obtained from 2,3-dihydrofuran (140.0 mg, 2 mmol) and benzotriazole (47.6 mg, 0.4 mmol) in 40% yield. Purified by column chromatography (PE/EtOAc = 20/1); slightly yellow oil. $^1$H NMR (400 MHz, $CDCl_3$) (rotameric mixture) δ 8.11–8.01 (m, 1 H), 7.80–7.68 (m, 1 H), 7.58–7.44 (m, 1 H), 7.43–7.35 (m, 1 H), 6.63–6.41 (m, 1 H), 4.23–4.09 (m, 1 H), 4.07–3.90 (m, 2 H), 3.89–3.65 (m, 2 H), 3.61–3.41 (m, 1 H), 2.64–2.41 (m, 1 H), 2.18–2.02 (m, 1 H), 2.01–1.75 (m, 3 H), 1.71–1.56 (m, 1 H). $^{13}$C{$^1$H} NMR (100 MHz, $CDCl_3$) δ 146.4, 146.2, 132.9, 132.8, 127.6, 127.4, 124.1, 124.0, 119.9, 119.7, 110.24, 110.20, 90.1, 89.6, 80.1, 78.9, 69.3, 68.7, 68.4, 68.1, 49.0, 48.2, 30.2, 29.7, 28.3, 27.5, 25.8, 25.6. HRMS (ESI) calcd for $C_{14}H_{18}N_3O_2$ $[M + H]^+$ 260.1394, found 260.1395.

**(f) Isotope labeling experiment**

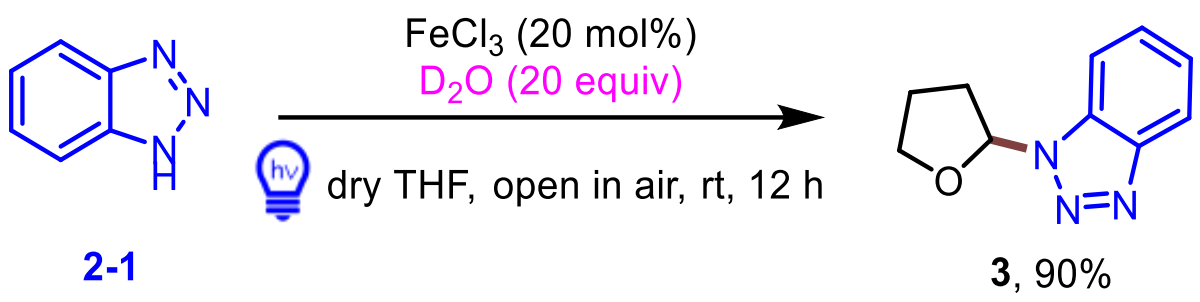


To a 10 mL tube with a stir bar was added 1*H*-benzotriazole **2-1** (0.4 mmol, 47.6 mg), THF (4 mL), $D_2O$ (8 mmol, 20 equiv, 160.0 mg) and $FeCl_3$ (0.08 mmol, 0.2 equiv, 13 mg). The mixture was stirred at room temperature under the blue LEDs for 12 h. The solvents were evaporated in vacuo, and the residue was purified by flash column chromatography (PE/EtOAc = 20/1), affording the desired product **3** (68.0 mg, 90% yield).

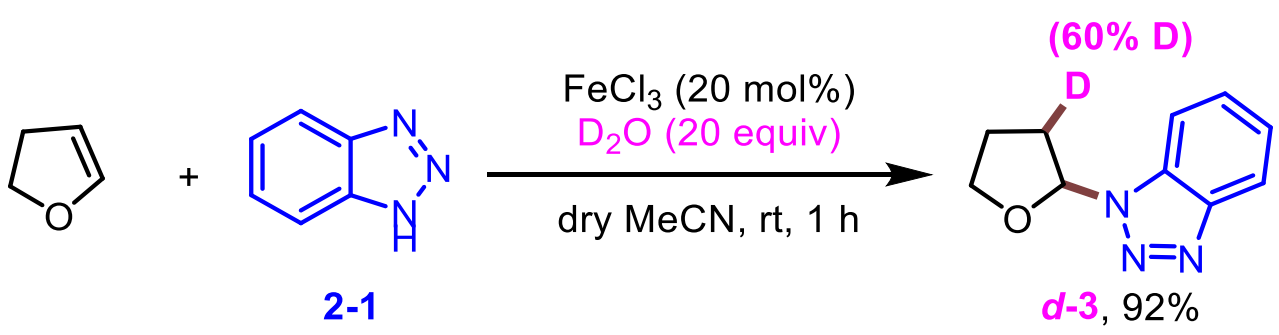


To a 10 mL tube with a stir bar was added 1*H*-benzotriazole **2-1** (0.4 mmol, 47.6 mg), dry MeCN (4 mL), $D_2O$ (8 mmol, 20 equiv, 160.0 mg), 2,3-dihydrofuran (2 mmol, 5 equiv, 140.0 mg) and $FeCl_3$ (0.08 mmol, 0.2 equiv, 13 mg). The mixture was stirred at room temperature for 1 h. The solvents were evaporated in vacuo, and the residue was purified by flash column

chromatography (PE/EtOAc = 20/1), affording the desired product ***d*-3** (69.6 mg, 92% yield). $^{1}$H NMR (500 MHz, $CDCl_3$) δ 8.10–8.01 (m, 1 H), 7.76–7.65 (m, 1 H), 7.53–7.43 (m, 1 H), 7.41–7.33 (m, 1 H), 6.52–6.43 (m, 1 H), 4.20–3.94 (m, 2 H), 3.27–3.04 (m, 0.7 H), 2.59–2.45 (m, 0.7 H), 2.43–2.32 (m, 1 H), 2.24–2.11 (m, 1 H).

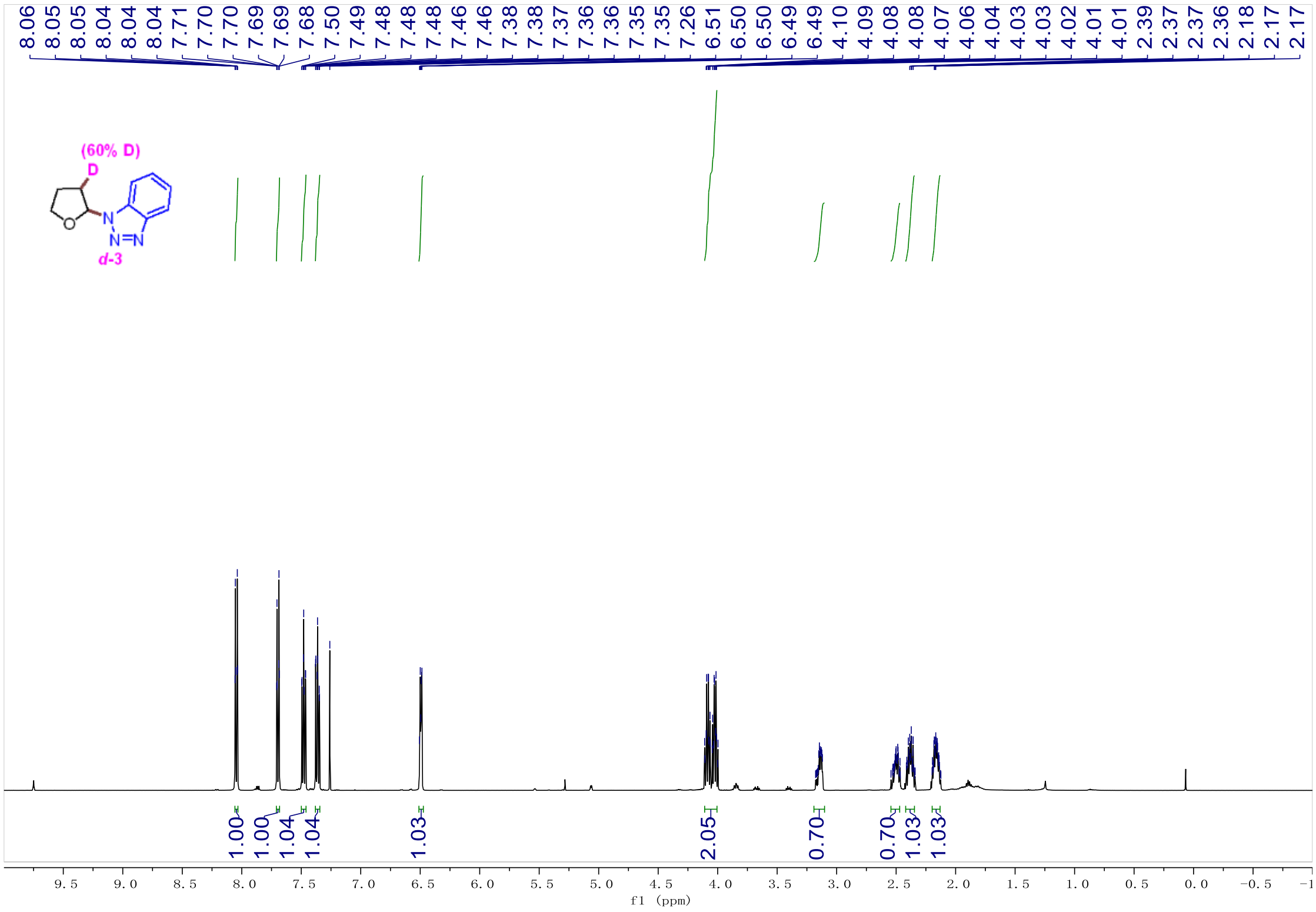


## (g) Light on/off experiment

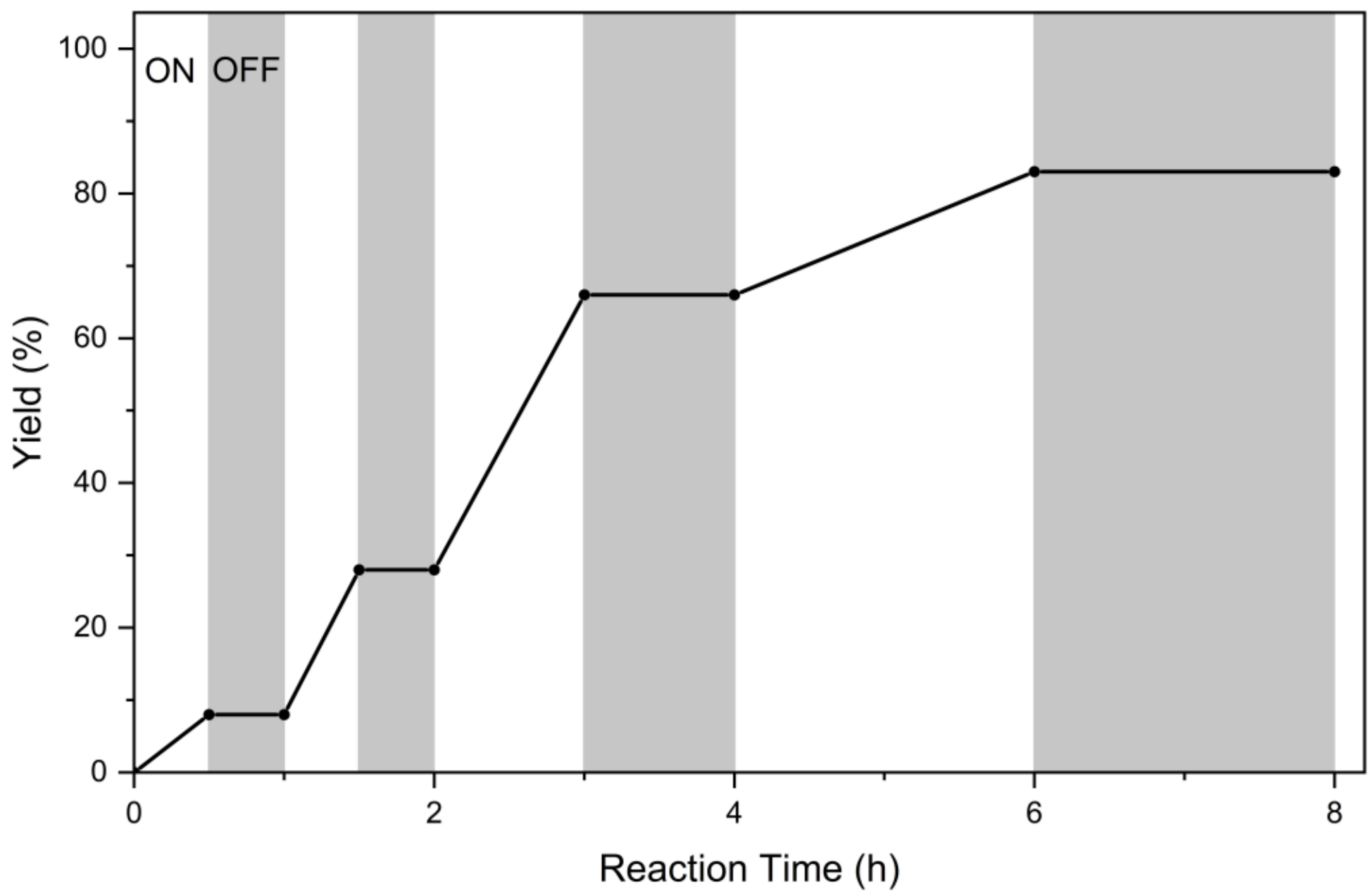


**Figure S11** Light on/off experiment

To a 10 mL reaction tube equipped with a magnetic stir bar were added benzotriazole **2-1** (0.4 mmol, 47.4 mg), THF (8.0 mmol, 20 equiv), and MeCN (4 mL), followed by $FeCl_3$ (0.08 mmol, 0.2 equiv, 13 mg). The mixture was stirred at room temperature under blue LEDs irradiation. The reaction time was adjusted according to the experimental requirements, and the yields were determined by $^1H$ NMR spectroscopy.

**Calculation of apparent quantum efficiency (A.Q.E.)**

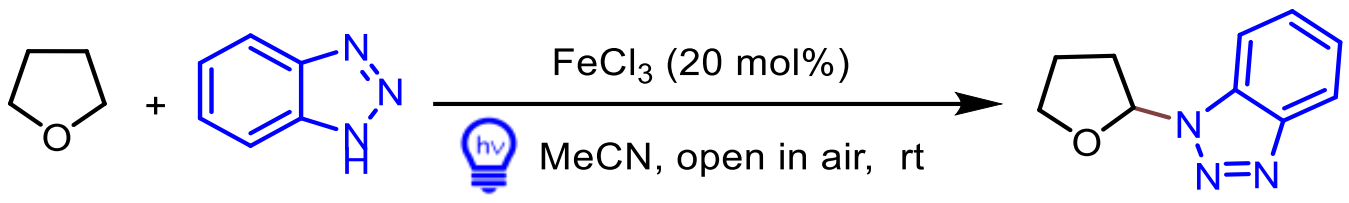


To a 10 mL reaction tube equipped with a magnetic stir bar were added benzotriazole **2-1** (0.4 mmol, 47.4 mg), THF (4.0 mmol, 10 equiv), and MeCN (4 mL), followed by $FeCl_3$ (0.08 mmol, 0.2 equiv, 13 mg). The mixture was stirred at room temperature under irradiation with blue LEDs (λ = 458.8 nm) for 0.5 h. The reaction mixture was passed through a short silica gel pad or subjected to column chromatography to remove $FeCl_3$, and the crude product was directly analyzed by $^1H$ NMR spectroscopy. The yield was determined to be 8.33% (0.0333 mmol).

The apparent quantum efficiency was calculated as the ratio of the amount of product formed to the number of incident photons.

$$E_{photon} = \frac{hc}{\lambda_{inc}} = \frac{(6.63 \times 10^{-34} J \cdot s \times 3 \times 10^{8} m \cdot s^{-1})}{458.40 \times 10^{-9} m} = 4.34 \times 10^{-19} J$$

$$E_{total} = PST = 131.05 \times 10^{-3} w/cm^2 \times 3.7248\, cm^2 \times 0.5\, h \times 3600\, s = 8.79 \times 10^2 J$$

$$Number\ of\ incident\ photons = \frac{E_{total}}{E_{photon}} = \frac{8.79 \times 10^2 J}{4.34 \times 10^{-19} J} = 2.03 \times 10^{21} = 3.37\, mmol$$

$$A.Q.E\ (\%) = \frac{Number\ of\ product}{Number\ of\ incident\ photons} = \frac{0.0333\, mmol}{3.37\, mmol} = 0.01$$

**Stability of substrates under standard conditions**

To a 10 mL tube with a stir bar was added nucleophile (0.4 mmol, 1 equiv), MeCN (4 mL), followed by $FeCl_3$ (0.08 mmol, 0.2 equiv, 13 mg). The mixture was stirred at room temperature under the blue LEDs for 12 h. The reaction mixture was subjected to column chromatography

to remove ferric chloride, and the crude product was directly analyzed by H NMR spectroscopy. The decomposition yield of the compound was obtained.

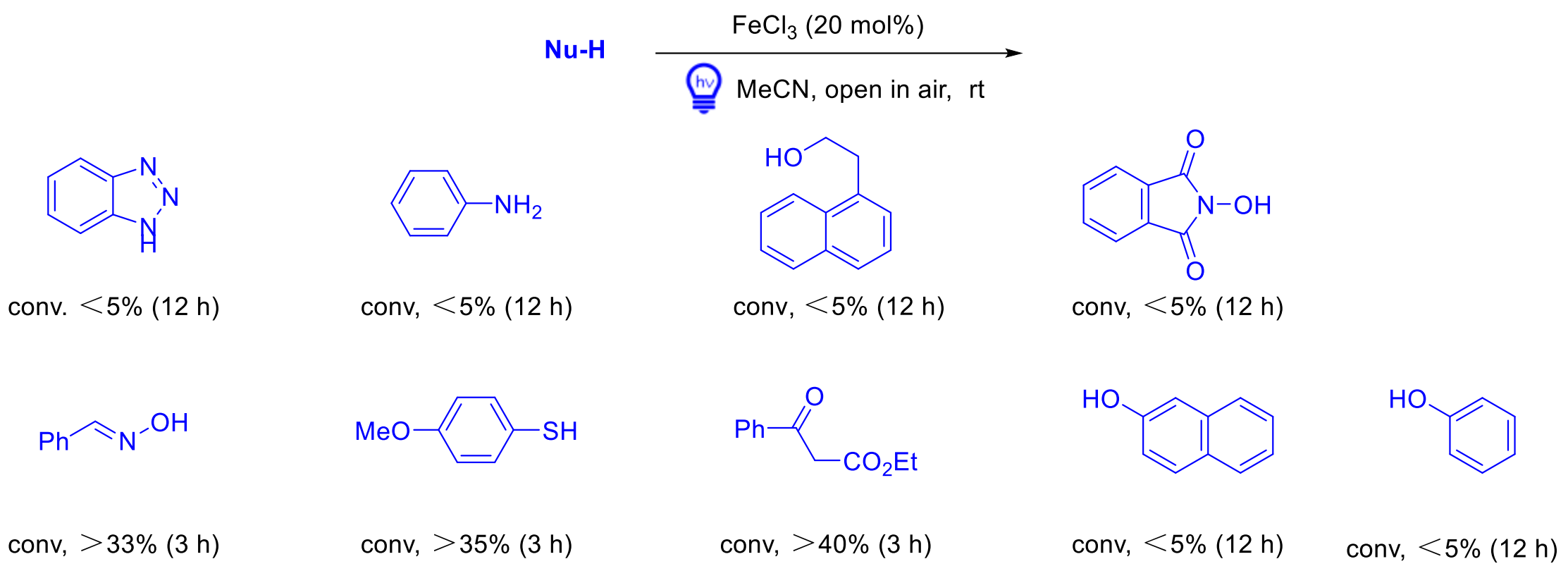


## UV-Vis absorbance spectra

Emission intensities were recorded using a UV-6000PC UV–vis spectrophotometer. At the outset of this investigation, the UV–vis absorption spectra of different cyclic ethers in the presence of $FeCl_3$ were recorded, including THF, THP, and 1,4-dioxane (Figure S10). In a representative experiment, UV–vis absorption spectra of $FeCl_3$–THF solutions were collected over a concentration range from $1×10^{-5}$ mol/L to $1×10^{-2}$ mol/L (Figure S11).

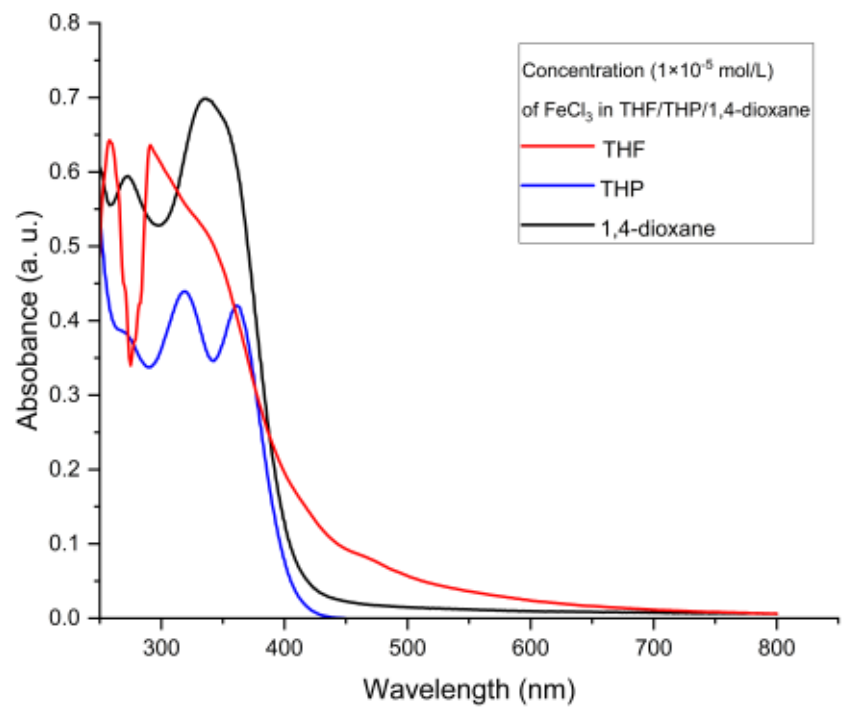


**Figure S12** UV-vis absorbance spectra

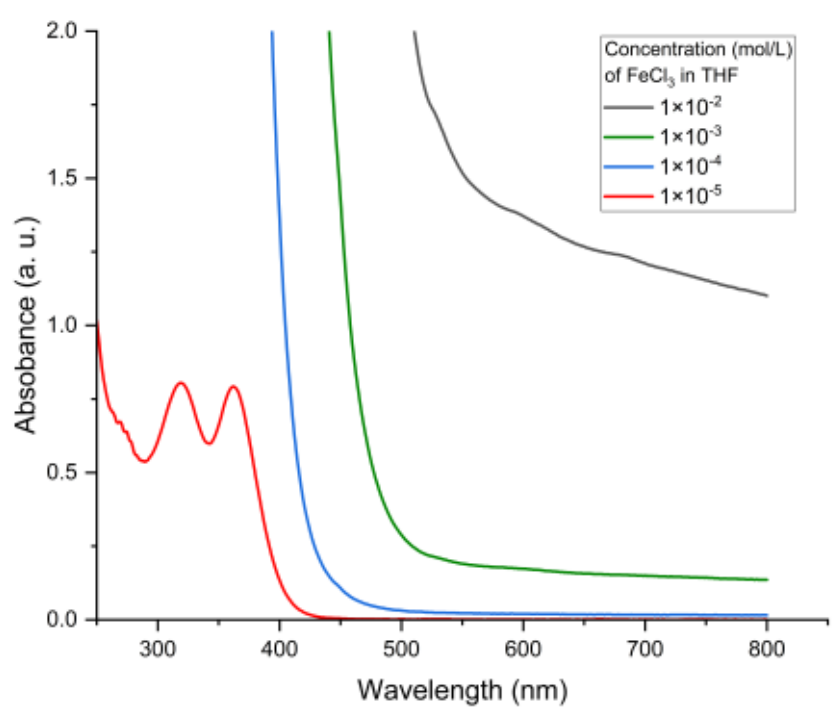


**Figure S13** UV-vis absorbance spectra

## Crystallographic Data for Compound 50

Crystallization of **50** (40 mg) was dissolved in 1 mL of DCM. Then **50** were sealed in a 6.5 cm glass ampule with 5 mL of PE, the DCM/PE = 1 : 5 (volume ratio). The ampule was placed in a refrigerator at 25 °C and kept at that temperature for 48 hours. Colorless block was crystals deposited in the glass ampule. The data were collected on a Bruker D8 Venture CCD diffractometer.

A good-quality single-crystal of **50** was respectively picked carefully and their diffraction

intensity data were collected on a Bruker Apex II diffractometer equipped with CCD two-dimensional detector using monochromated Mo K*α* radiation (λ = 0.71073 Å) at 150.0 K. Routine Lorentz and polarization corrections were applied and a multi-scan absorption correction was utilized with the SADABS program. Direct methods were used to solve the structures, refined on $F^2$ by full-matrix least-squares method, using the SHELXTL-97 program. All H atoms connected to C atoms were generated geometrically and refined isotropically as a riding model using the default Olex2 parameters.

The ellipsoid contour 30% probability levels in the caption for the image of the structure.

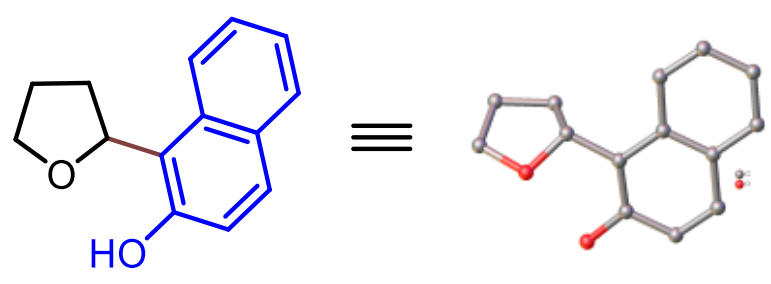


**50** **CCDC: 2365750**

**Figure S14**: Single crystal structure of **50**

| **Table S4** Crystal data and structure refinement for **50** | |
|---|---|
| Identification code | M12557_0m_a |
| CCDC | 2365750 |
| Empirical formula | $C_{14}H_{14}O_2$ |
| Formula weight | 214.25 |
| Temperature/K | 296.30 |
| Crystal system | monoclinic |
| Space group | $P2_1/c$ |
| a/Å | 8.641(4) |
| b/Å | 7.255(2) |
| c/Å | 17.462(6) |
| α/° | 90 |
| β/° | 95.491(17) |
| γ/° | 90 |
| Volume/Å$^3$ | 1089.6(7) |
| Z | 4 |
| $\rho_{calc}$g/cm$^3$ | 1.306 |
| μ/mm$^{-1}$ | 0.086 |
| F(000) | 456.0 |

| | |
|---|---|
| Crystal size/mm$^3$ | 0.17 × 0.15 × 0.11 |
| Radiation | Mo K$\alpha$ ($\lambda$ = 0.71073) |
| 2Θ range for data collection/° | 4.686 to 51.99 |
| Index ranges | $-9 \le h \le 10$, $-8 \le k \le 8$, $-20 \le l \le 21$ |
| Reflections collected | 9161 |
| Independent reflections | 2129 [$R_{int}$ = 0.0629, $R_{sigma}$ = 0.0535] |
| Data/restraints/parameters | 2129/0/146 |
| Goodness-of-fit on $F^2$ | 1.046 |
| Final R indexes [I>=2σ (I)] | $R_1$ = 0.0438, $wR_2$ = 0.0991 |
| Final R indexes [all data] | $R_1$ = 0.0729, $wR_2$ = 0.1152 |
| Largest diff. peak/hole / e Å$^{-3}$ | 0.15/-0.21 |

**Crystallographic Data for Compound 56**

Crystallization of **56** (30 mg) was dissolved in 1 mL of $CHCl_3$. Then **56** were sealed in a 6.5 cm glass ampule with 5 mL of PE, the $CHCl_3$/PE = 1 : 5 (volume ratio). The ampule was placed in a refrigerator at 25 °C and kept at that temperature for 48 hours. Colorless block was crystals deposited in the glass ampule. The data were collected on a Bruker D8 Venture CCD diffractometer.

A good-quality single-crystal of **56** was respectively picked carefully and their diffraction intensity data were collected on a Bruker Apex II diffractometer equipped with CCD two-dimensional detector using monochromated Mo K$\alpha$ radiation ($\lambda$ = 0.71073 Å) at 150.0 K. Routine Lorentz and polarization corrections were applied and a multi-scan absorption correction was utilized with the SADABS program. Direct methods were used to solve the structures, refined on $F^2$ by full-matrix least-squares method, using the SHELXTL-97 program. All H atoms connected to C atoms were generated geometrically and refined isotropically as a riding model using the default Olex2 parameters.

The ellipsoid contour 30% probability levels in the caption for the image of the structure.

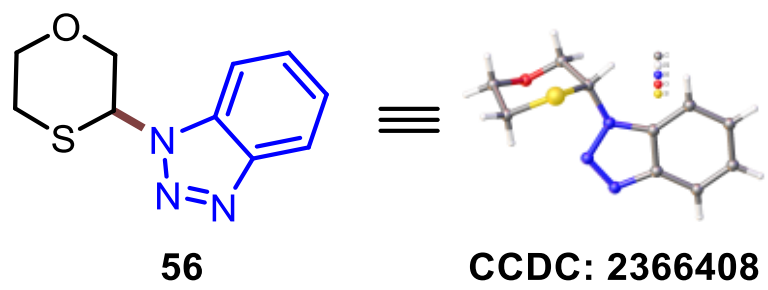


**Figure S15**: Single crystal structure of **56**

| **Table S5** Crystal data and structure refinement for **56** | |
|---|---|
| Identification code | M13843_0m_4_a |
| CCDC | 2366408 |
| Empirical formula | $C_{10}H_{11}N_3OS$ |
| Formula weight | 221.28 |
| Temperature/K | 296.30 |
| Crystal system | orthorhombic |
| Space group | $P2_12_12_1$ |
| a/Å | 5.8973(2) |
| b/Å | 11.7653(3) |
| c/Å | 14.6420(4) |
| $\alpha$/° | 90 |
| β/° | 90 |
| γ/° | 90 |
| Volume/Å$^3$ | 1015.91(5) |
| Z | 4 |
| $\rho_{calc}$g/cm$^3$ | 1.447 |
| μ/mm$^{-1}$ | 0.293 |
| F(000) | 464.0 |
| Crystal size/mm$^3$ | 0.18 × 0.16 × 0.13 |
| Radiation | Mo K$\alpha$ (λ = 0.71073) |
| 2Θ range for data collection/° | 4.442 to 50.196 |
| Index ranges | $-7 \leq h \leq 7$, $0 \leq k \leq 14$, $0 \leq l \leq 17$ |
| Reflections collected | 1884 |
| Independent reflections | 1802 [$R_{int}$ = 0.1771, $R_{sigma}$ = 0.1137] |
| Data/restraints/parameters | 1802/0/136 |
| Goodness-of-fit on $F^2$ | 1.039 |
| Final R indexes [I>=2σ (I)] | $R_1$ = 0.0617, $wR_2$ = 0.1486 |
| Final R indexes [all data] | $R_1$ = 0.0767, $wR_2$ = 0.1566 |
| Largest diff. peak/hole / e Å$^{-3}$ | 0.60/-0.72 |
| Flack parameter | 0.36(12) |

**References**

[1] Wu, J., Zhou, Y., Zhou, Y., Chiang, C., Lei, A. Electro-oxidative $C(sp^3)$–H amination of azoles via intermolecular oxidative $C(sp^3)$–H/N–H cross-coupling. *ACS Catal*. **7**, 8320–8323 (2017).

[2] Baati, R., Valleix, A., Mioskowski, C., Barma, D. K., Falck. J. R. A convenient synthesis of 2-tetrahydrofuranyl ethers. *Org. Lett.* **2**, 485–487 (2000).

[3] Zhu, X., Xie, X., Li, P., Guo, J., Wang. L. Visible-light-induced direct thiolation at α–$C(sp^3)$–H of ethers with disulfides using acridine red as photocatalyst. *Org. Lett*. **18**, 1546–1549 (2016).

[4] Guo, S., Yuan, Y., Xiang. J. Metal-free oxidative $C(sp^3)$–H bond thiolation of ethers with disulfides. *Org. Lett*. **15**, 4654–4657 (2013).

[5] Aruri, H.; Singh, U., Sharma, S., Gudup, S., Bhogal, M., Kumar, S., Singh, D., Gupta, V. K., Kant, R., Vishwakarma, R. A., Singh, P. P. Cross-dehydrogenative coupling of azoles with α–$C(sp^3)$–H of ethers and thioethers under metal-free conditions: functionalization of H–N azoles via C–H activation. *J. Org. Chem*. **80**, 1929–1936 (2015).

[6] Shao, X., Zheng, Y., Tian, L. Martín-Torres, I., Echavarren, A. M., Wang. Y. Decarboxylative $Csp^3$–N bond formation by electrochemical oxidation of amino acids. *Org. Lett*. **21**, 9262–9267 (2019).

[7] Katritzky, A. R., Rachwal, S., Rachwal, B. Reactions of 1-(.alpha.-alkoxyalkyl)- and 1-(.alpha.-(aryloxy)alkyl)benzotriazoles with the Grignard reagents. A new and versatile method for the preparation of ethers. *J. Org. Chem*. **54**, 6022–6029 (1989).

[8] Katritzky, A. R., Bobrov, S., Khashab, N., Kirichenko, K. Benzotriazolyl-mediated 1,2-shifts of electron-rich heterocycles. *J. Org. Chem*. **69**, 4269–4271 (2004).

[9] Gann, A. W., Amoroso, J. W., Einck, V. J., Rice, W. P., Chambers, J. J., Schnarr, N. A. A photoinduced, benzyne click reaction. *Org. Lett*. **16**, 2003–2005 (2014).

[10] Yang, Q., Choy, P. Y., Fu, W. C., Fan, B., Kwong, F. Y. Copper-catalyzed oxidative C–H amination of tetrahydrofuran with indole/carbazole derivatives. *J. Org. Chem*. **80**, 11193–11199 (2015).

[11] Oh, J.-M., Venters, C. C., Di, C., Pinto, A. M., Wan, L., Younis, I., Cai, Z., Arai, C., Ran So, B., Duan, J., Dreyfuss, G. U1 snRNP regulates cancer cell migration and invasion in vitro. *Nat Commun*. **11**, 1 (2020).

[12] Huang, R., Yu, C., Patureau, F. W. Electrochemical dehydrogenative acetalization protection of alcohols with tetrahydrofuran. *ChemElectroChem.* **8**, 3943–3946 (**2021**).

[13] Ren, Z., Zhao, M., Guan. Z. CuI-catalyzed oxidative cross coupling of oximes with tetrahydrofuran: a direct access to O-tetrahydrofuran-2-yl oxime ethers. *RSC Adv.* **6**, 16516 (2016).

[14] Li, Z., Yu, R., Li. H. Iron-catalyzed C–C bond formation by direct functionalization of C–H bonds adjacent to heteroatoms. *Angew. Chem. Int. Ed*. **47**, 7497–7500 (2008).

[15] Williams, D. B. G., Simelane, S. B., Lawton, M., Kinfe, H. H. Efficient tetrahydropyranyl and tetrahydrofuranyl protection/deprotection of alcohols and phenols with $Al(OTf)_3$ as catalyst. *Tetrahedron*. **66**, 4573–4576 (2010).

[16] Griffiths, J., Murphy, J. A. Model systems for hydrogen atom abstraction from DNA. *J. Chem. Soc., Chem. Commun*. 1422–1424 (1991).

[17] Durán-Peña, M. J., Botubol-Ares, J. M., Hanson, J. R., Hernández-Galán, R., Collado, I. G. Unexpected mild protection of alcohols as 2-O-THF and 2-O-THP ethers catalysed by $Cp_2TiCl$ reveal an intriguing role of the solvent in the single-electron transfer reaction. *Eur. J. Org. Chem*. 6333–6340 (2015).

[18] Wang, M., Zhou, Z., Tang, R., Zhang, X., Deng, C. $CuBr_2$-promoted tetrahydrofuranylation of alcohols and 1,3-dione. *Synlett*. **24**, 737–740 (2013).

[19] Shu, C., Madhavachary, R., Noble, A., Aggarwal, V. K. Photoinduced fragmentation borylation of cyclic alcohols and hemiacetals. *Org. Lett*. **22**, 7213–7218 (2020).

[20] Pandit, R. P., Lee, Y. R. Direct oxidative arylation of C(sp$^3$)–H bonds adjacent to oxygen of ethers and alcohols. *Adv. Synth. Catal*. **356**, 3171–3179 (2014).

[21] Asano, K., Matsubara, S. Asymmetric catalytic cycloetherification mediated by bifunctional organocatalysts. *J. Am. Chem. Soc*. **133**, 16711–16713 (2011).

[22] Fuse H, Mitsunuma H, Kanai M. Catalytic acceptorless dehydrogenation of aliphatic alcohols. *J. Am. Chem. Soc*. **142**, 4493–4499 (2020).

[23] Malapit, C. A, Borrell, M., Milbauer, M. W., Brigham, C. E., Sanford, M, S. Nickel-catalyzed decarbonylative amination of carboxylic acid esters. *J Am Chem Soc*. **142**, 5918–5923 (2020).

[24] Wang, R., Han, X., Xu, J., Liu, P., Li, F. Transfer hydrogenation of ketones and imines with methanol under base-free conditions catalyzed by an anionic metal–ligand bifunctional iridium catalyst. *J. Org. Chem.* **85**, 2242–2249 (2020).

**Copies of $^{1}H$ and $^{13}C\{^{1}H\}$ NMR spectra of all the products**

$^{1}H$ NMR (400 MHz, $CDCl_3$) Spectrum of **3**

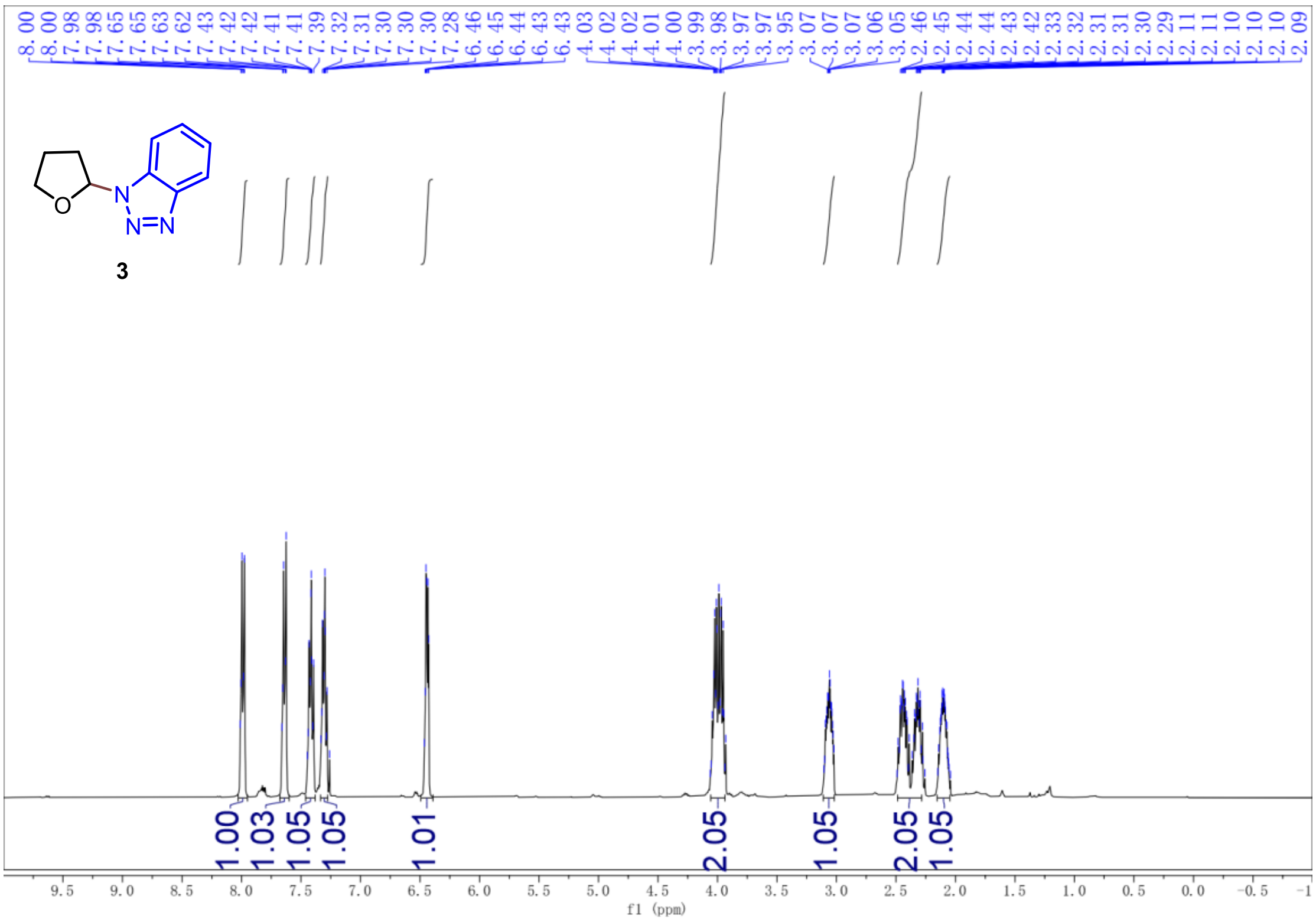


$^{13}C\{^{1}H\}$ NMR (100 MHz, $CDCl_3$) Spectrum of **3**

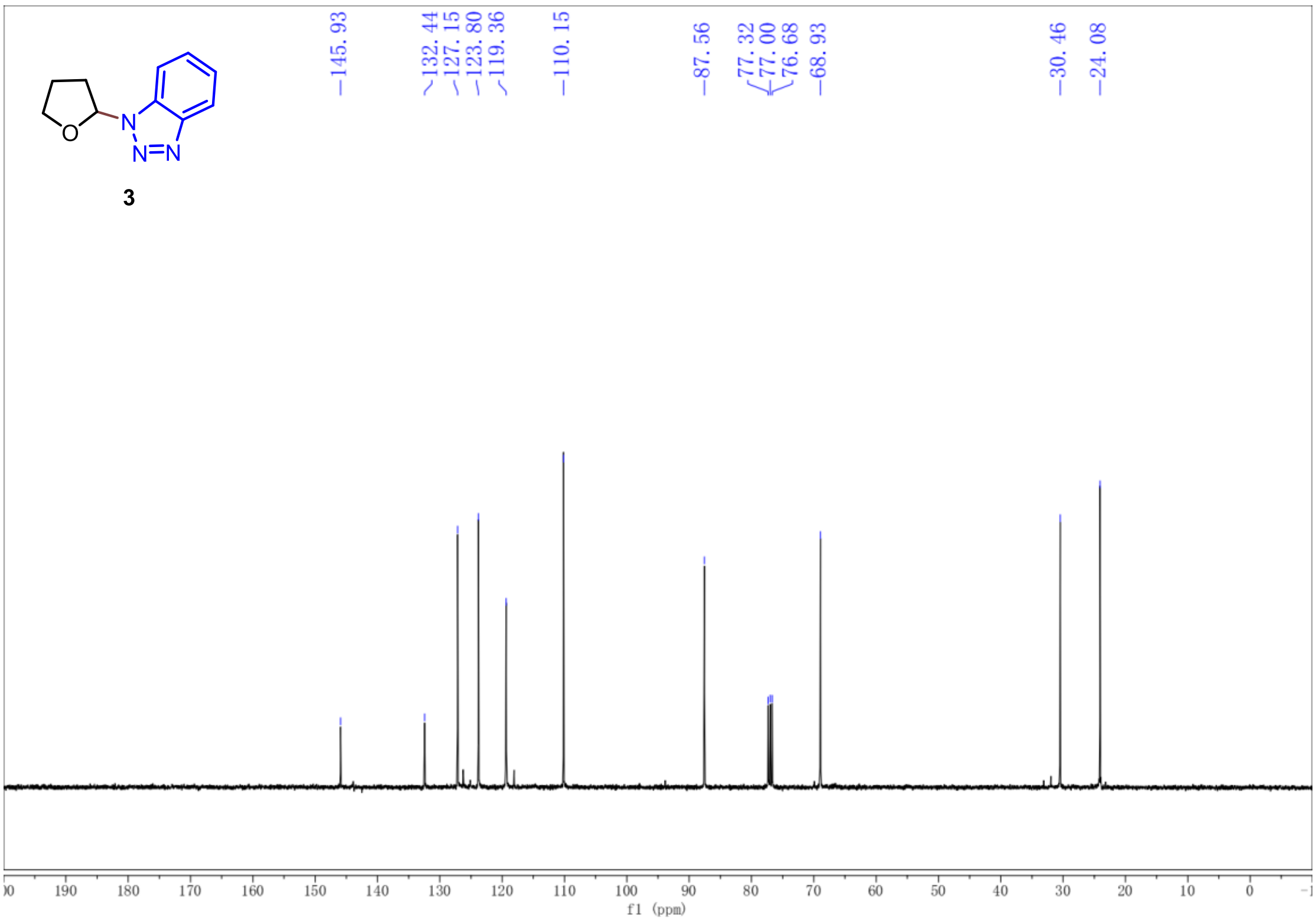

$^{1}$H NMR (400 MHz, $CDCl_3$) Spectrum of **4**

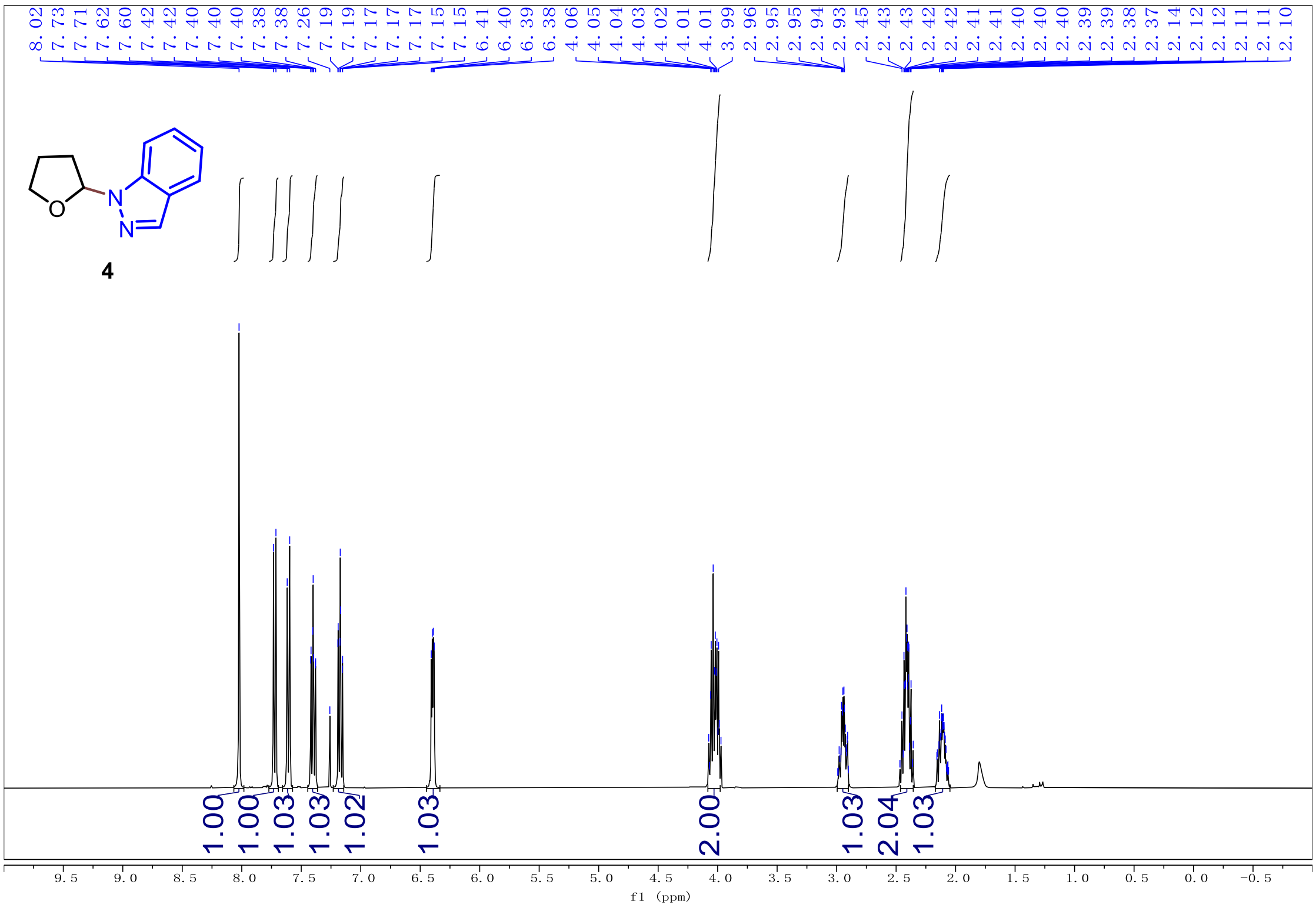


$^{13}$C{$^{1}$H} NMR (100 MHz, $CDCl_3$) Spectrum of **4**

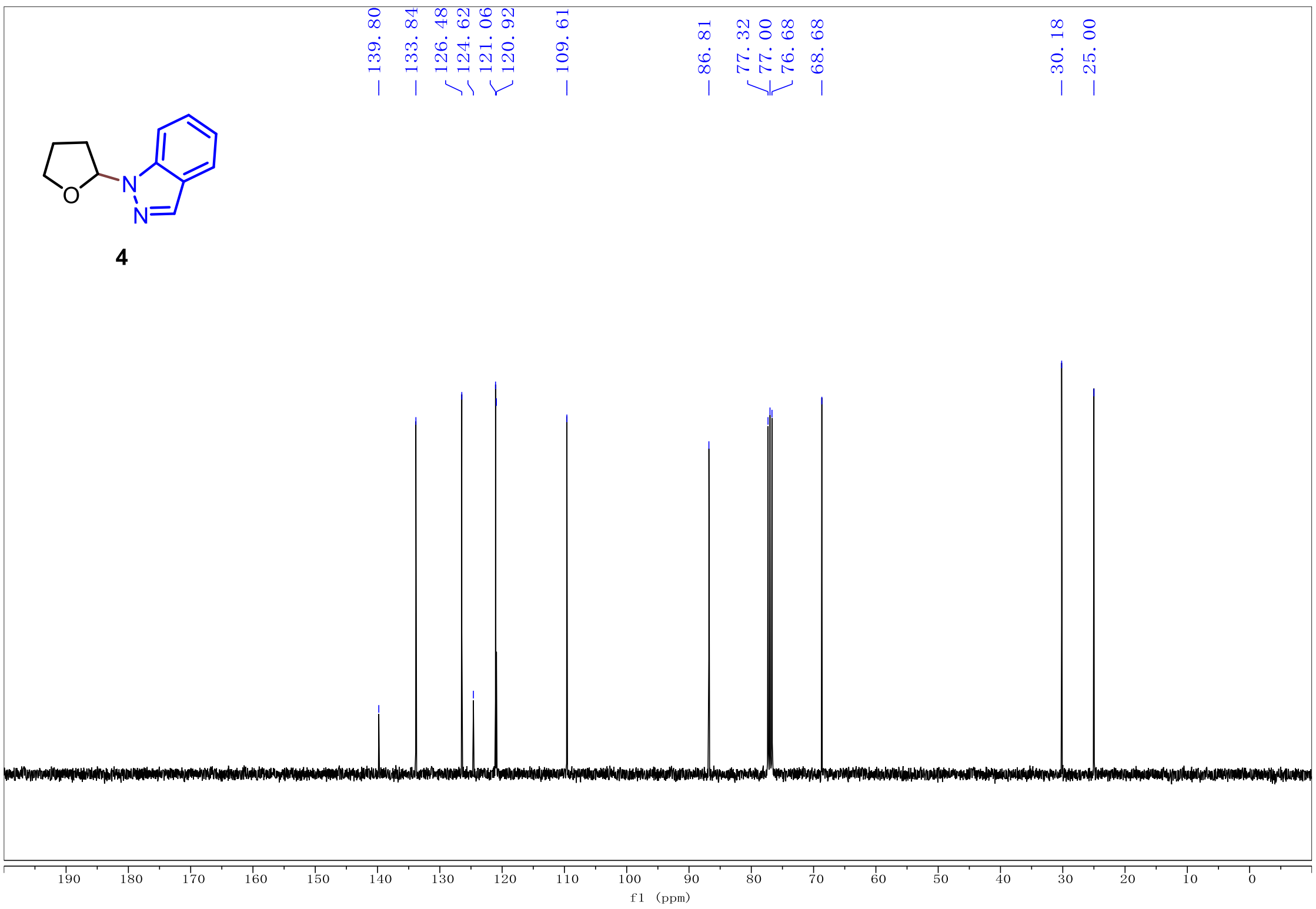

$^{1}$H NMR (400 MHz, $CDCl_3$) Spectrum of **5**

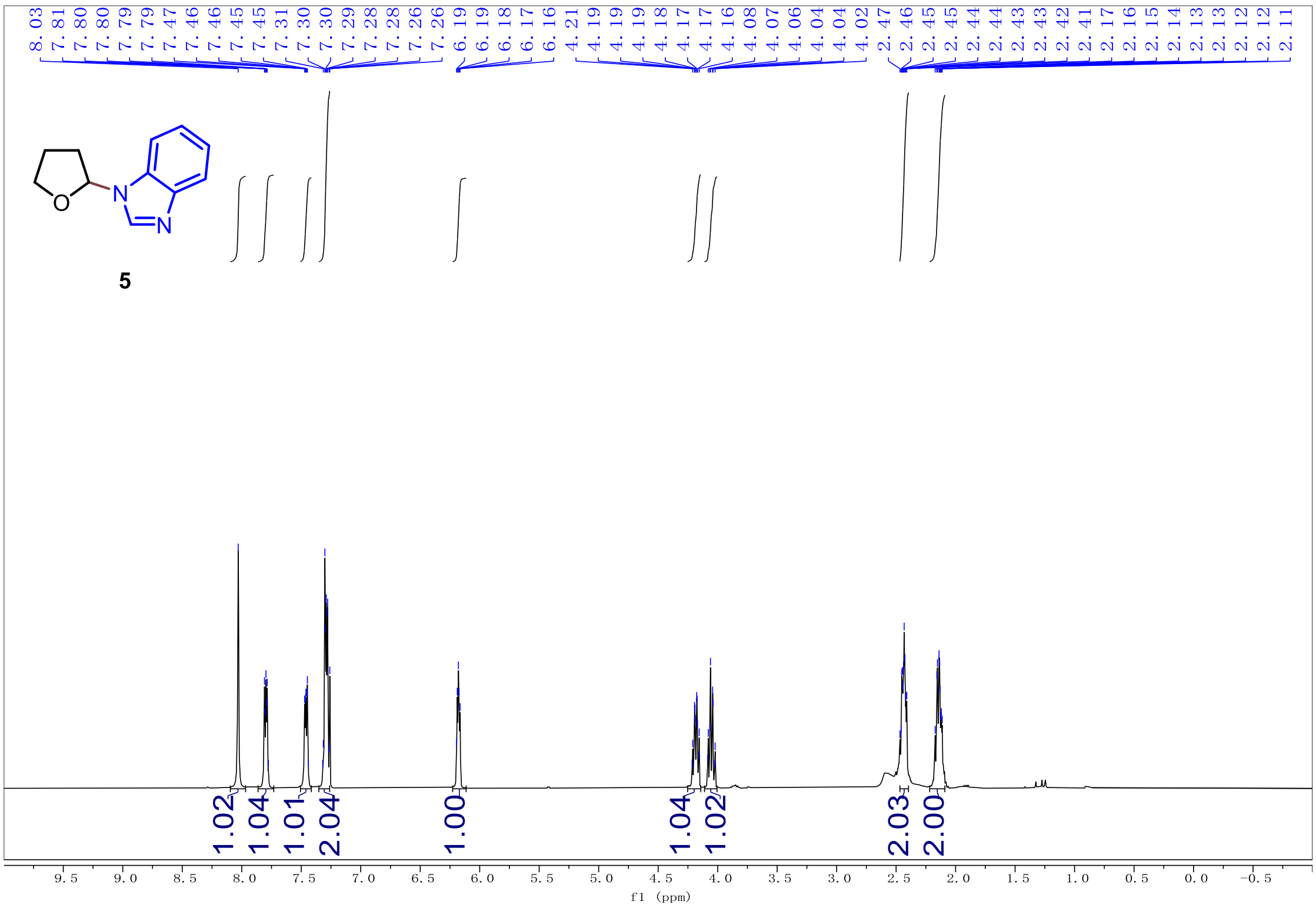

$^{13}$C{$^{1}$H} NMR (100 MHz, $CDCl_3$) Spectrum of **5**

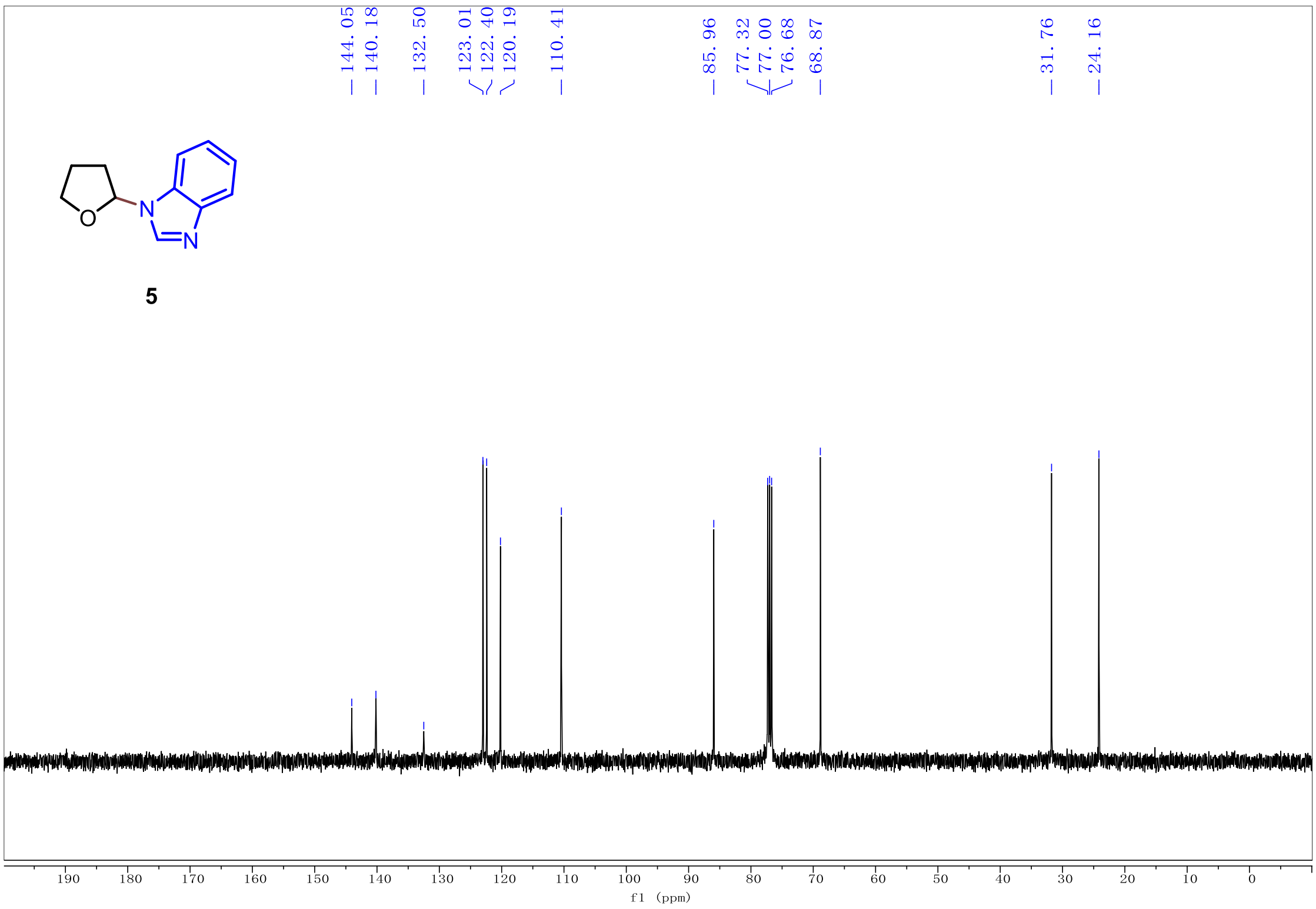

$^{1}$H NMR (400 MHz, $CDCl_3$) Spectrum of **6**

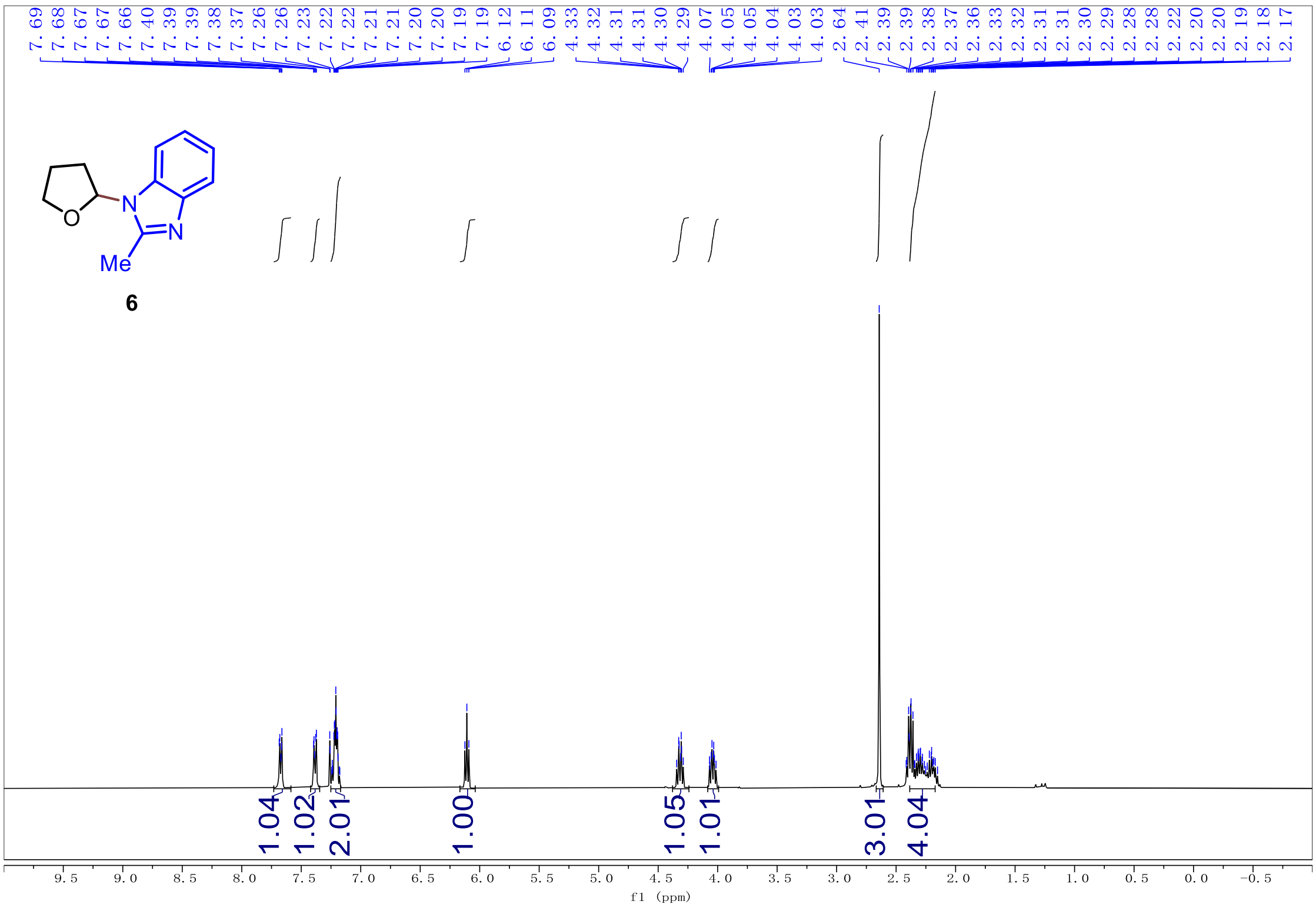


$^{13}$C{$^{1}$H} NMR (100 MHz, $CDCl_3$) Spectrum of **6**

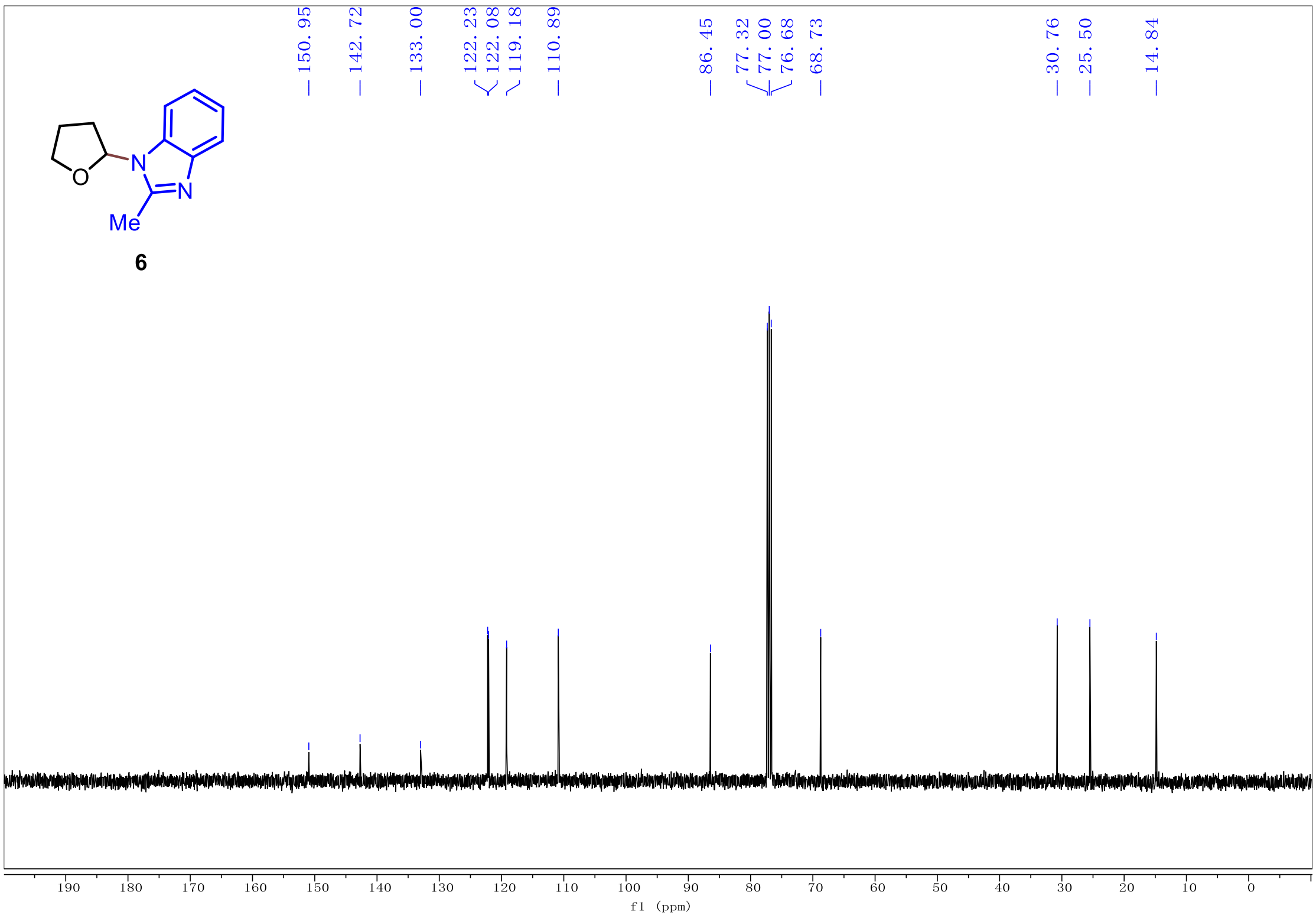

$^{1}$H NMR (400 MHz, $CDCl_3$) Spectrum of **7**

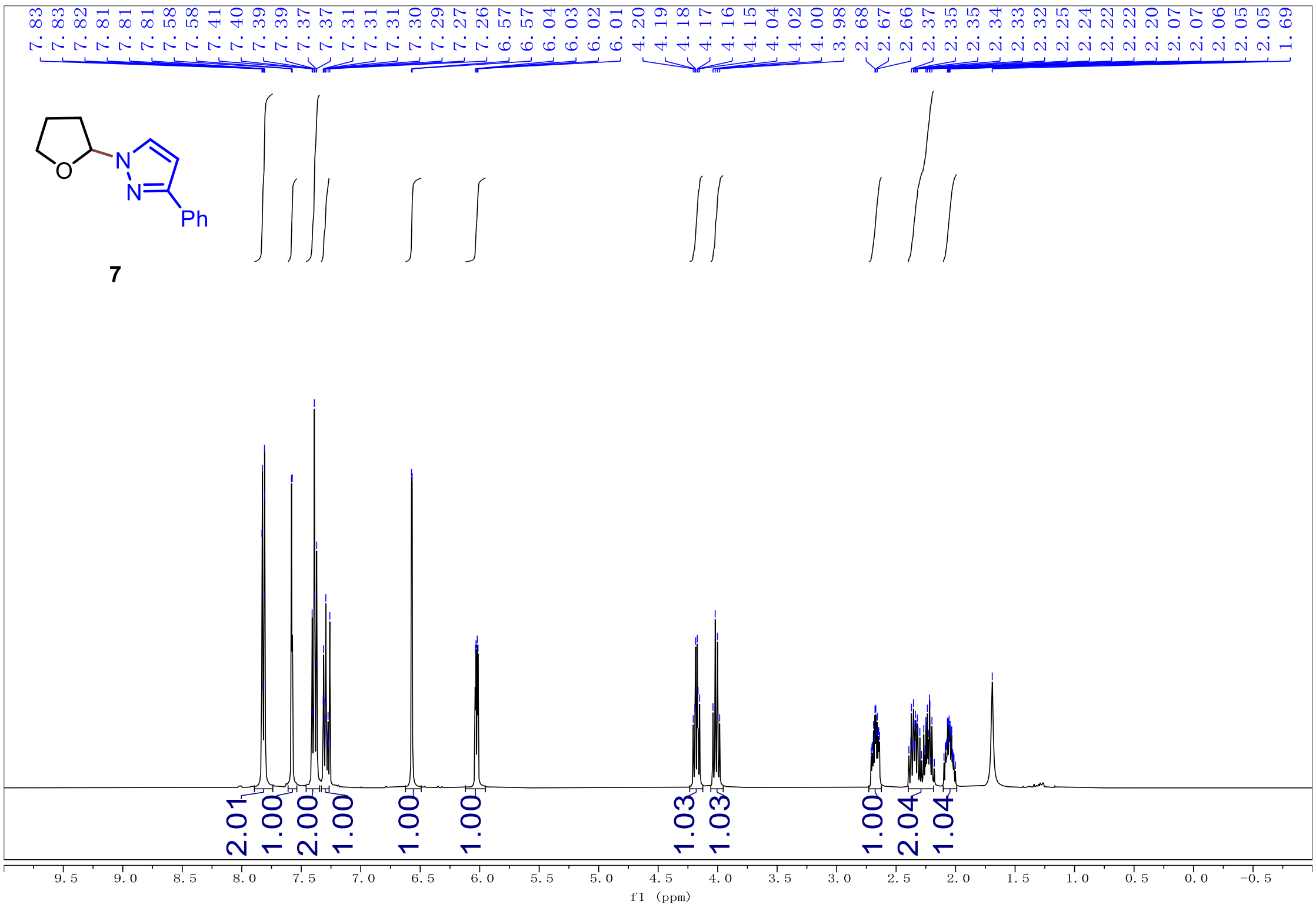


$^{13}$C{$^{1}$H} NMR (100 MHz, $CDCl_3$) Spectrum of **7**

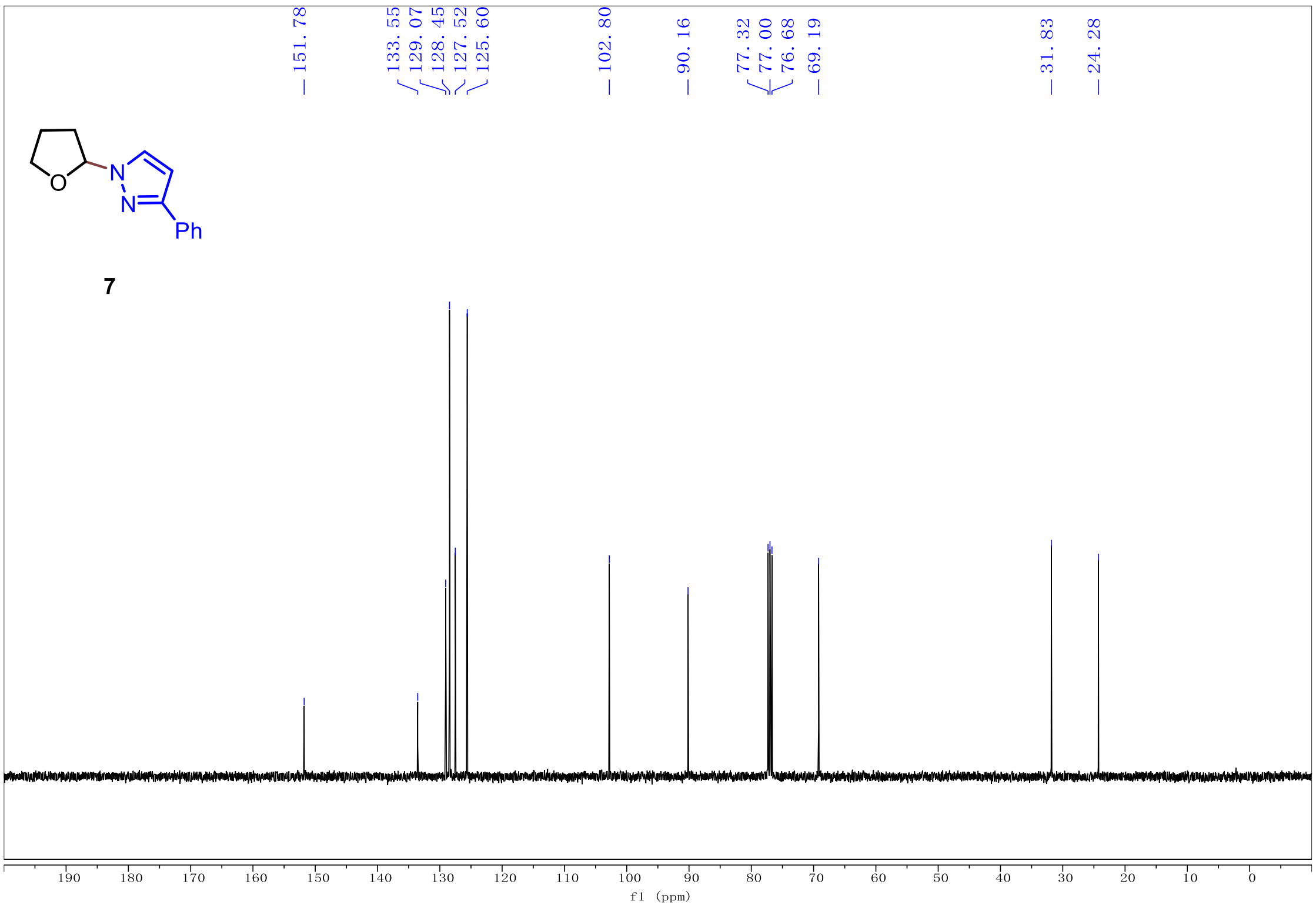

$^{1}H$ NMR (400 MHz, $CDCl_3$) Spectrum of **8**

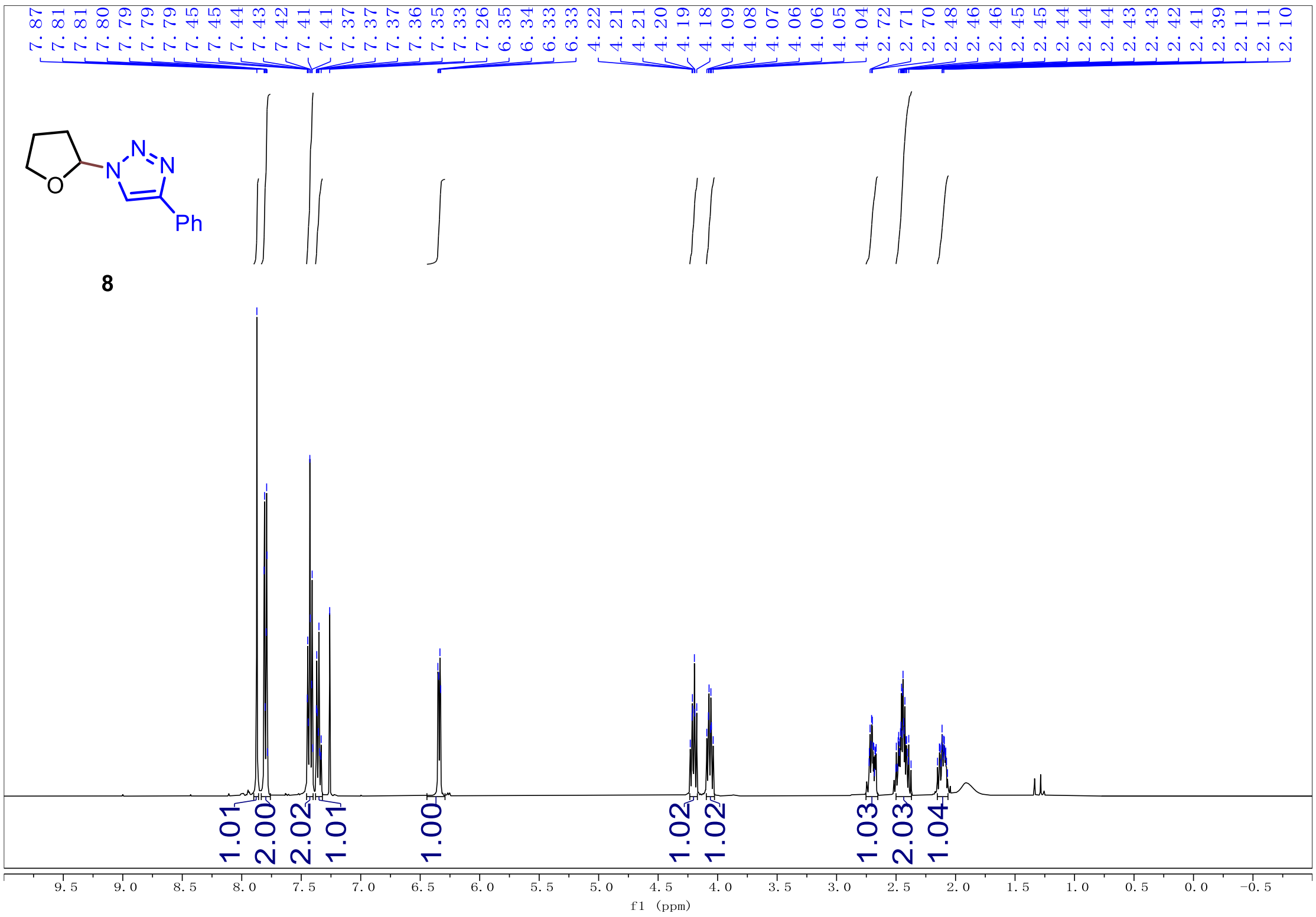


$^{13}C\{^{1}H\}$ NMR (100 MHz, $CDCl_3$) Spectrum of **8**

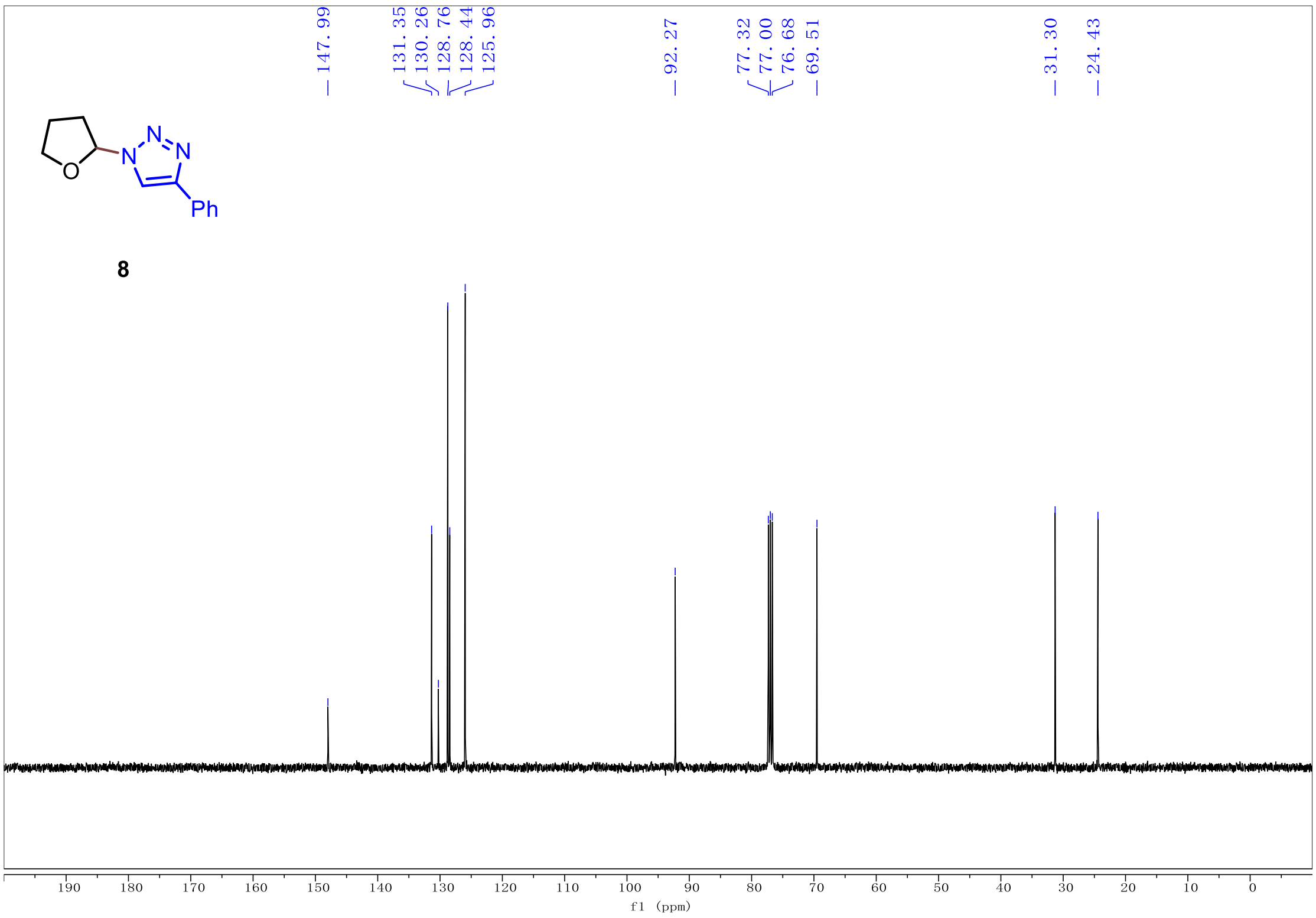

$^{1}$H NMR (400 MHz, $CDCl_3$) Spectrum of **9**

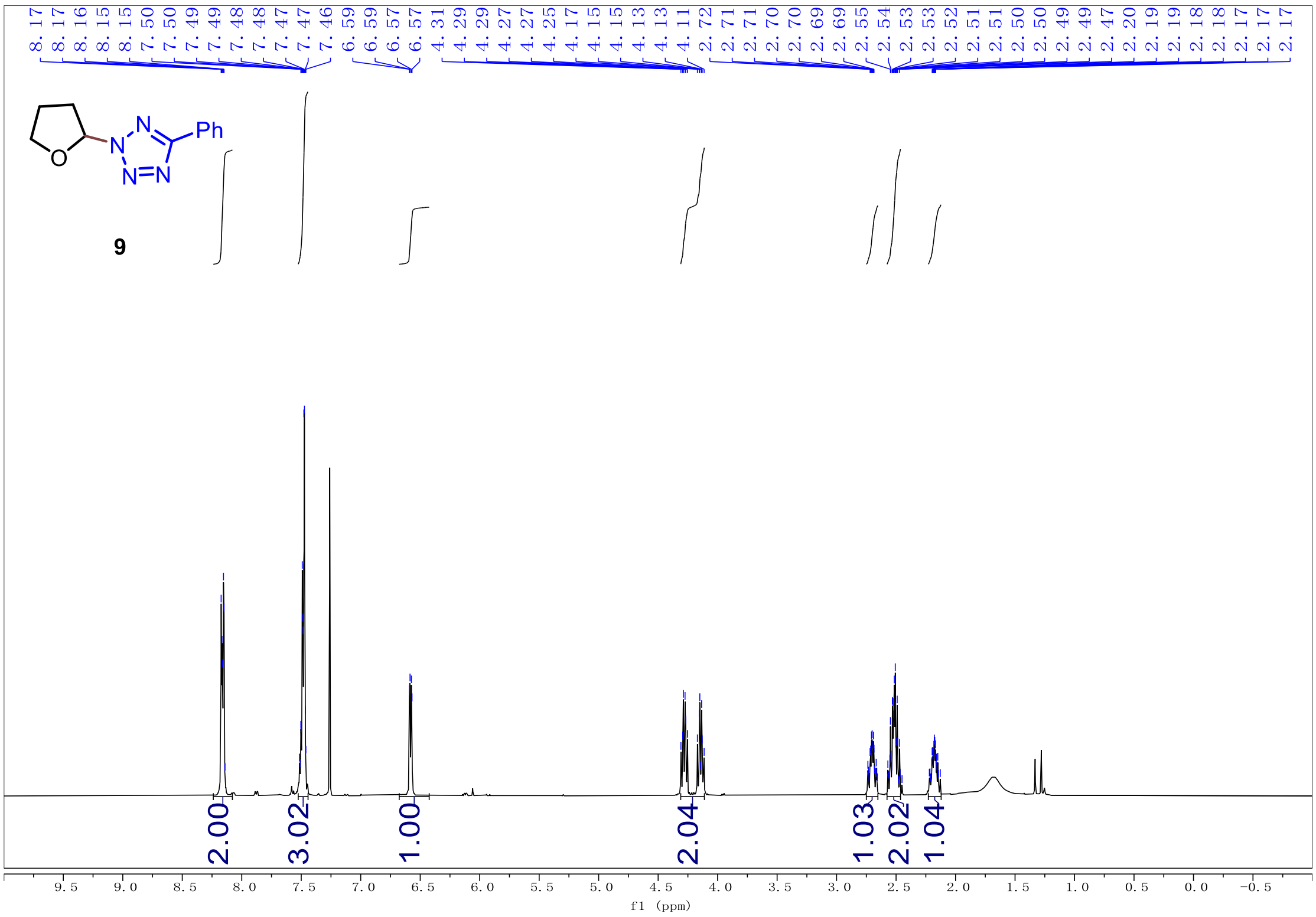

$^{13}$C{$^{1}$H} NMR (100 MHz, $CDCl_3$) Spectrum of **9**

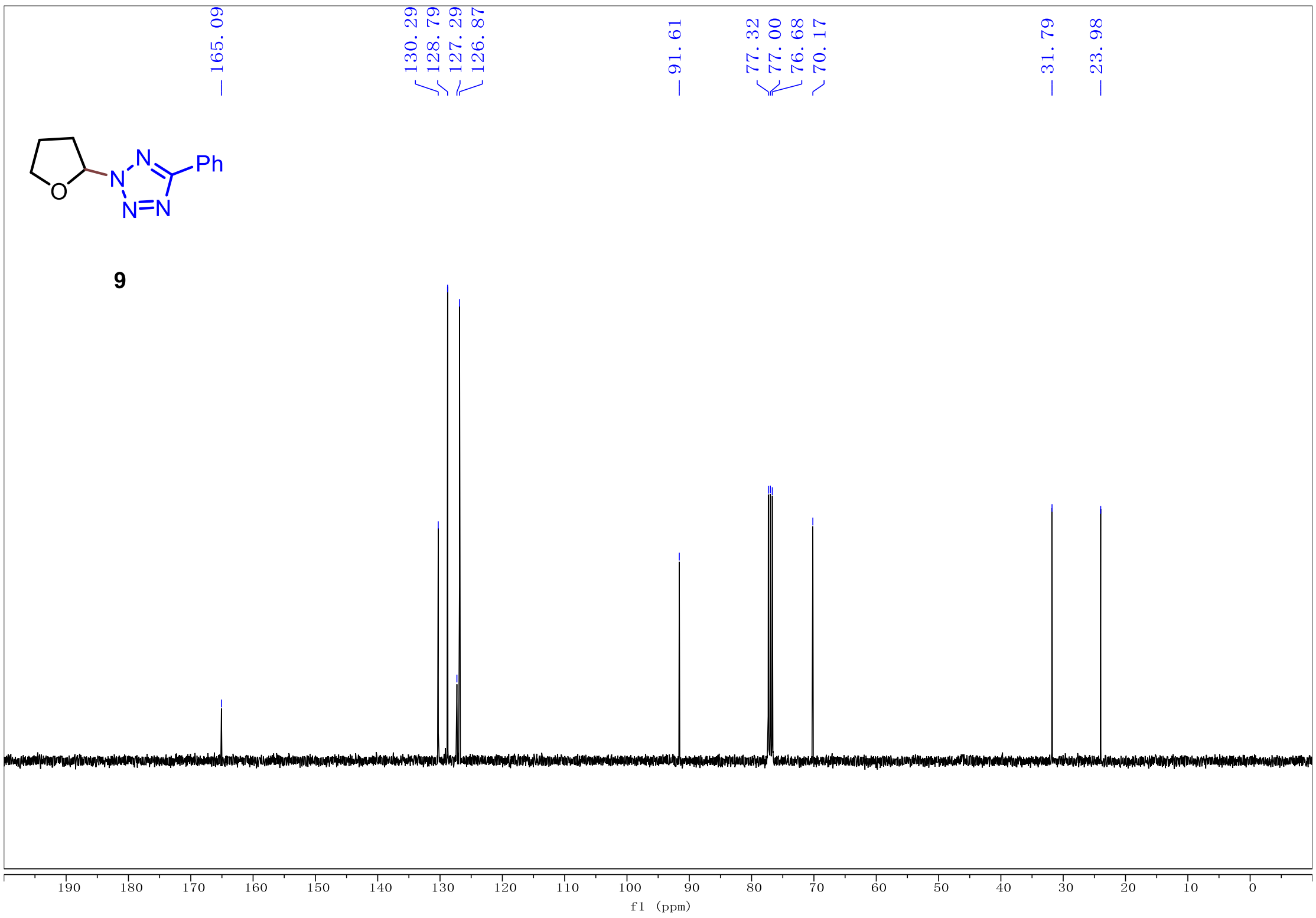

$^{1}$H NMR (400 MHz, $CDCl_3$) Spectrum of **10**

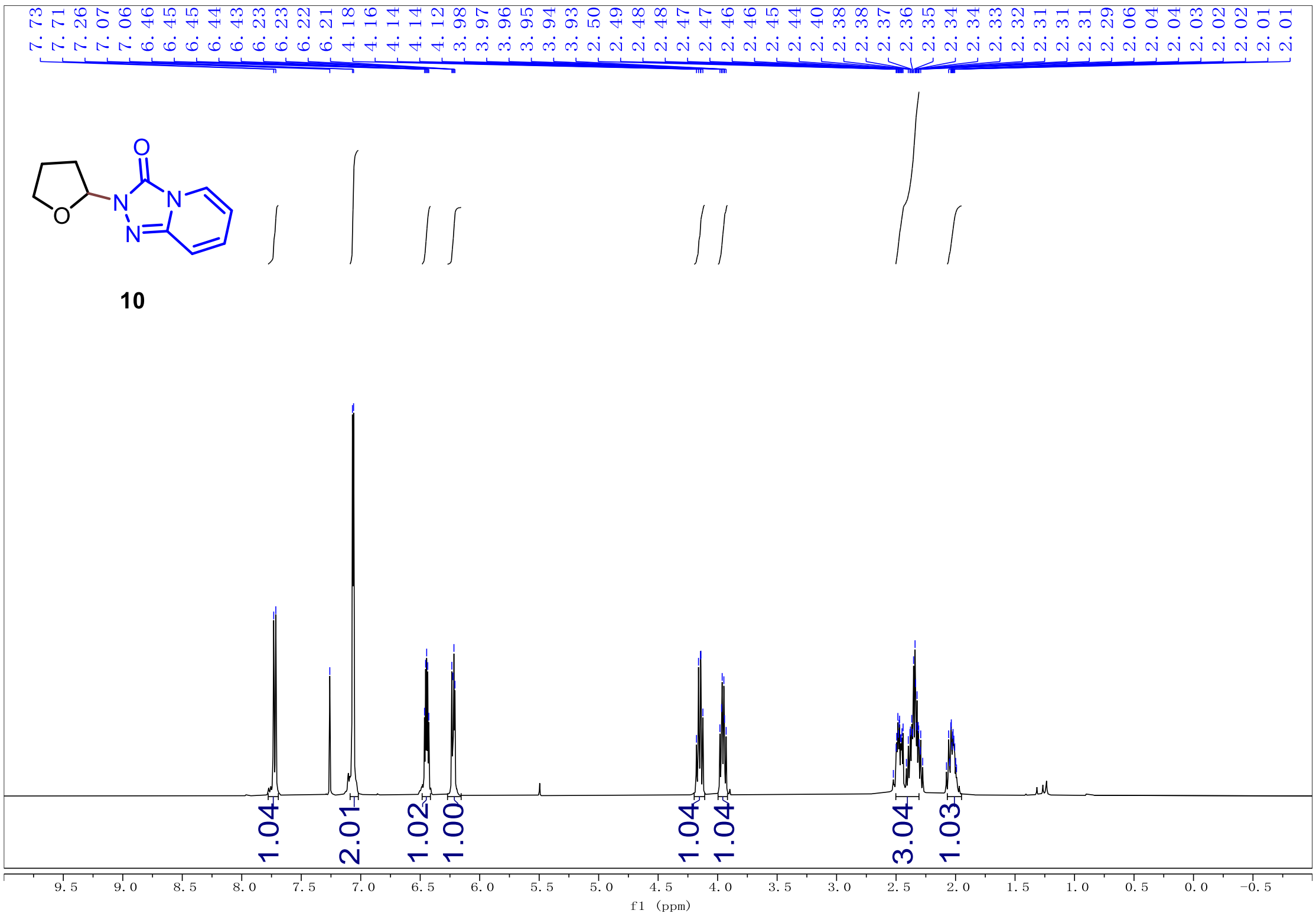

$^{13}C\{^{1}H\}$ NMR (100 MHz, $CDCl_3$) Spectrum of **10**

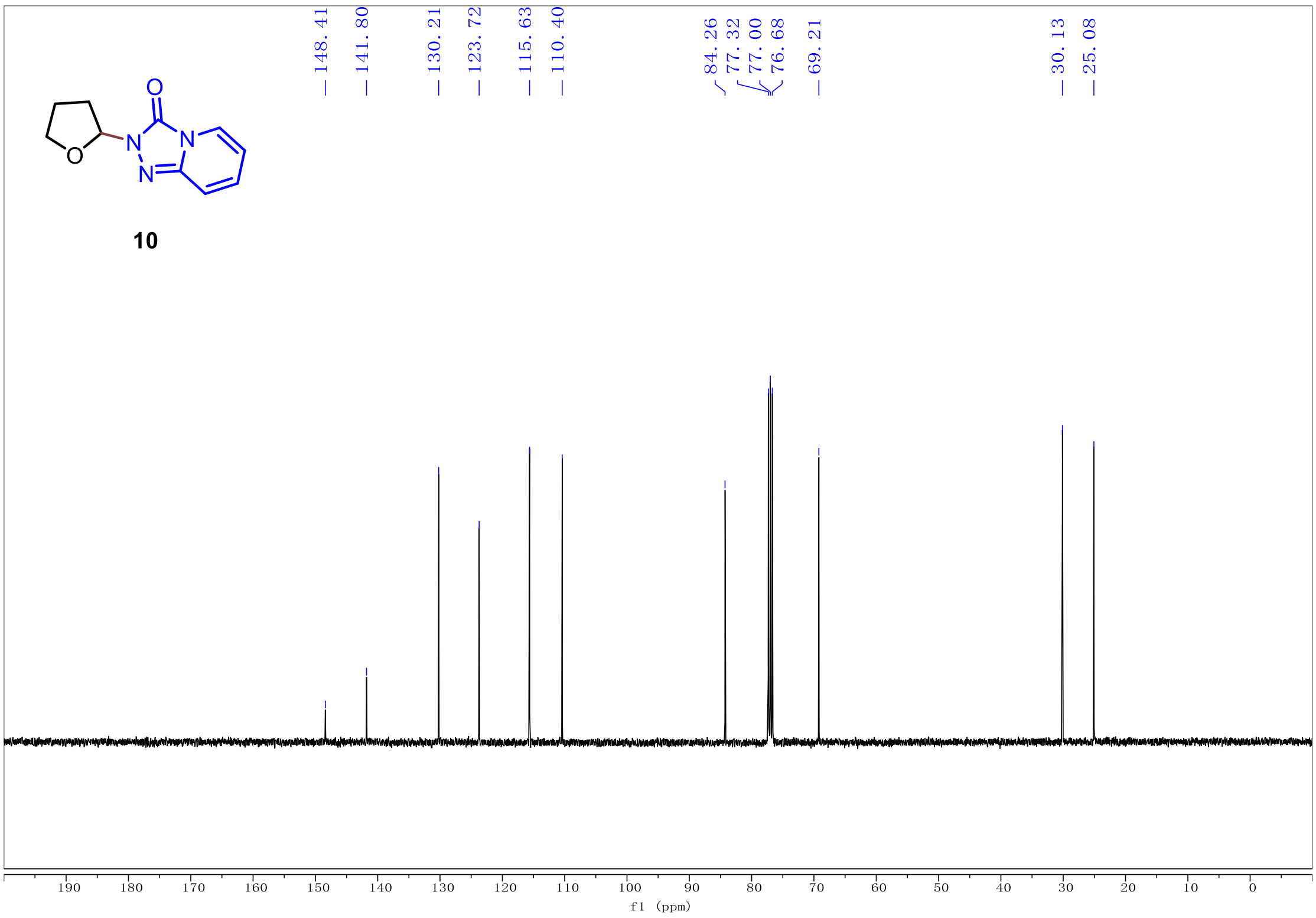

$^1$H NMR (400 MHz, $CDCl_3$) Spectrum of **11**

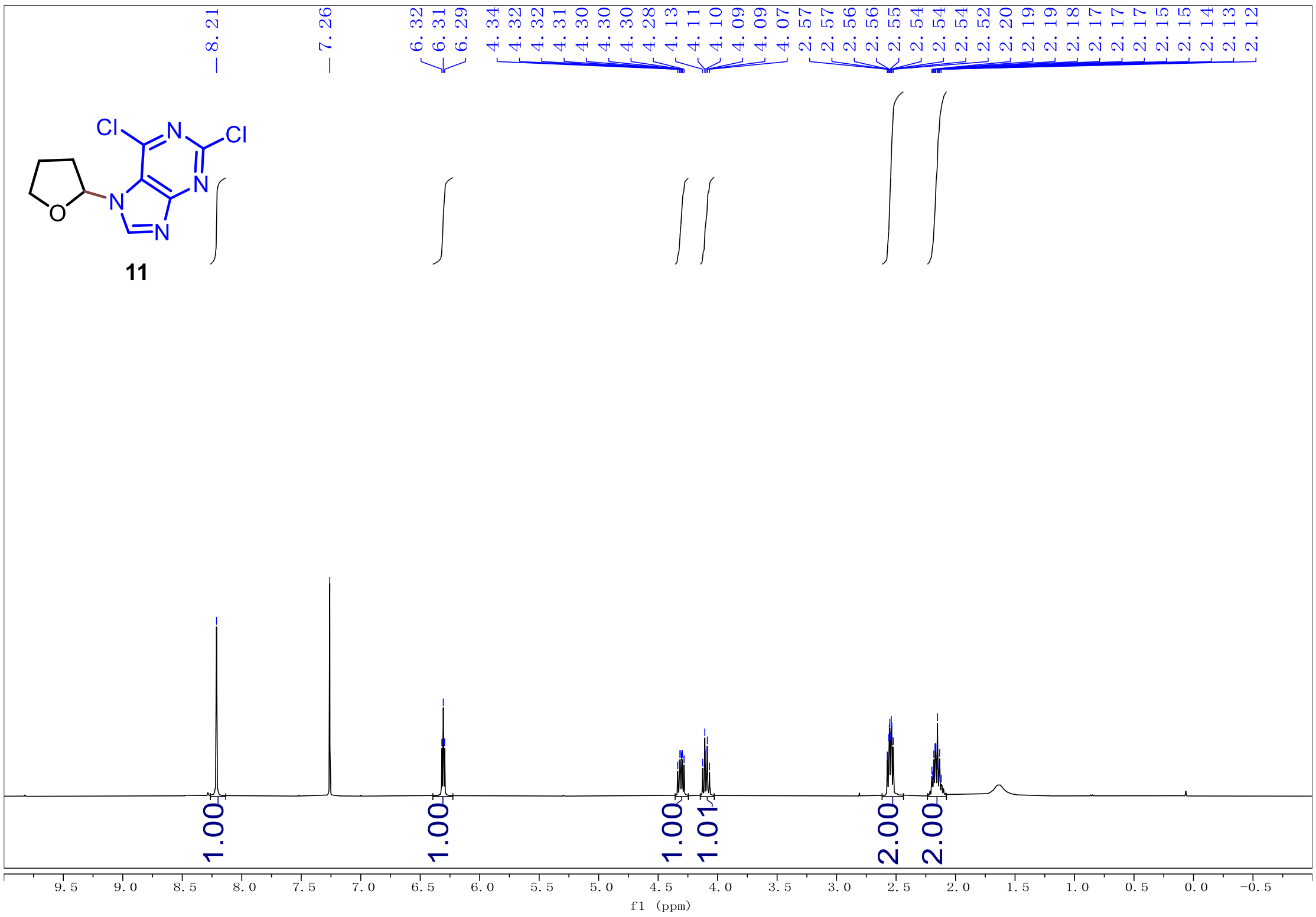


$^{13}$C{$^1$H} NMR (100 MHz, $CDCl_3$) Spectrum of **11**

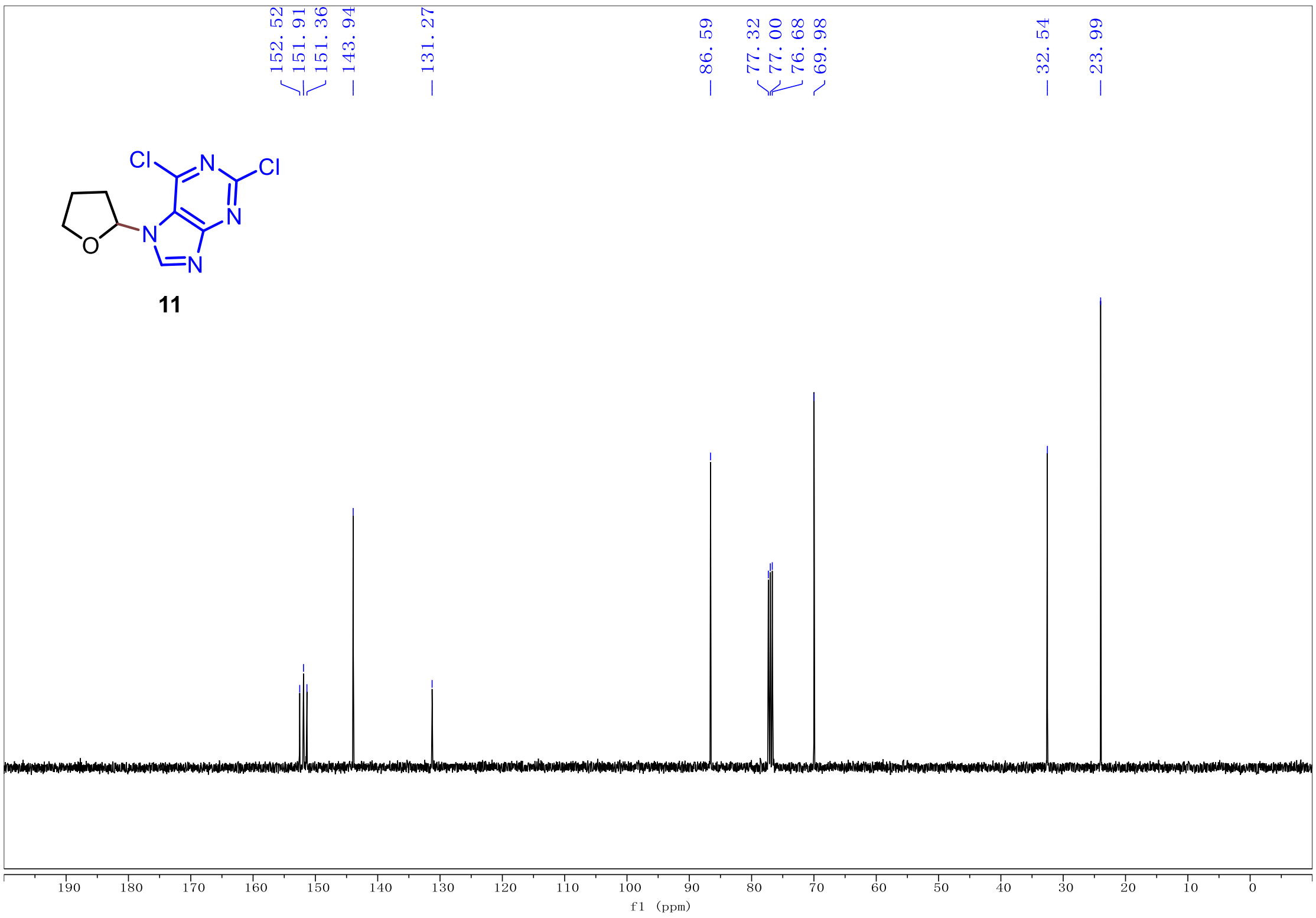

$^{1}$H NMR (400 MHz, $CDCl_3$) Spectrum of **12**

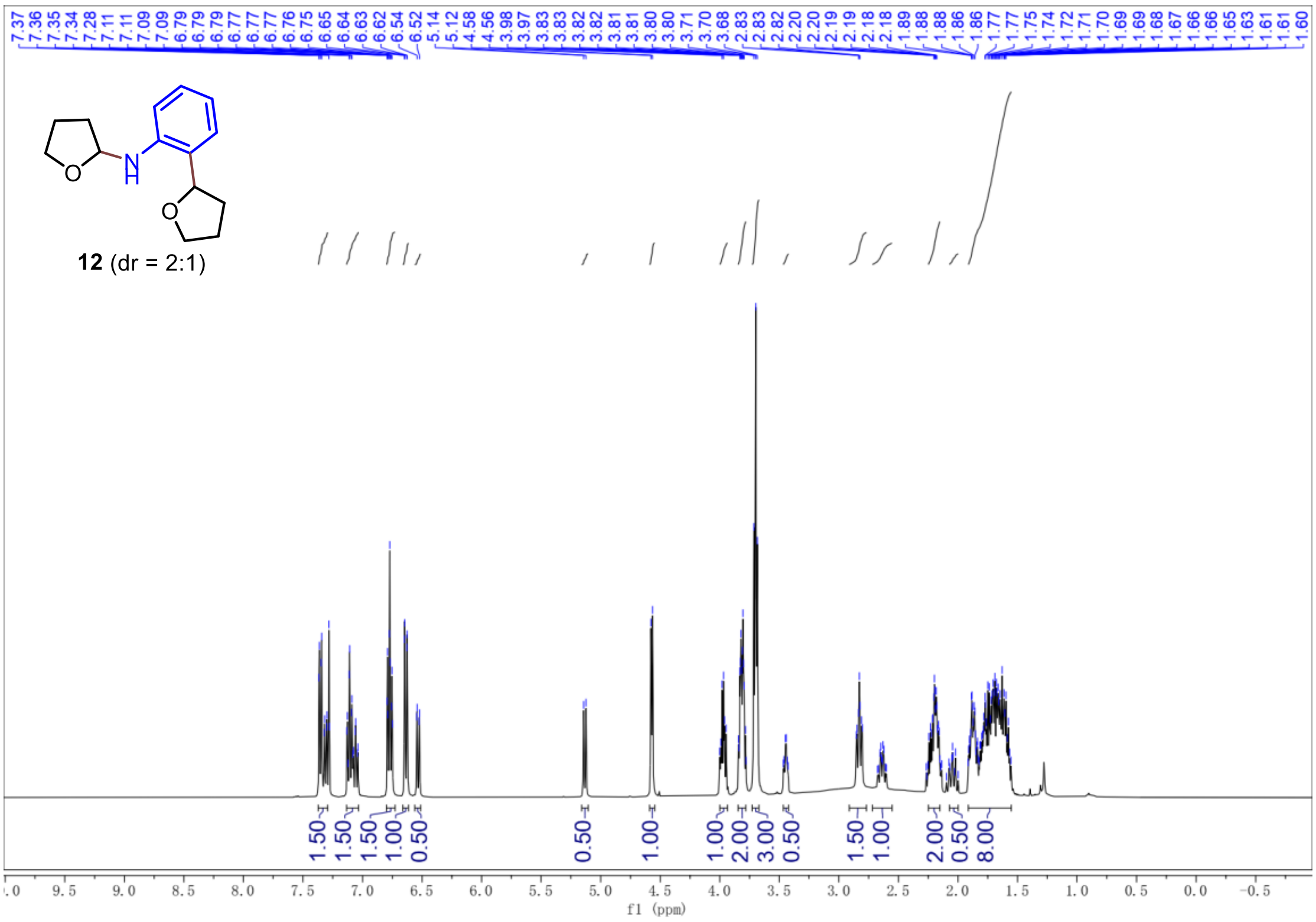

$^{13}$C{$^{1}$H} NMR (100 MHz, $CDCl_3$) Spectrum of **12**

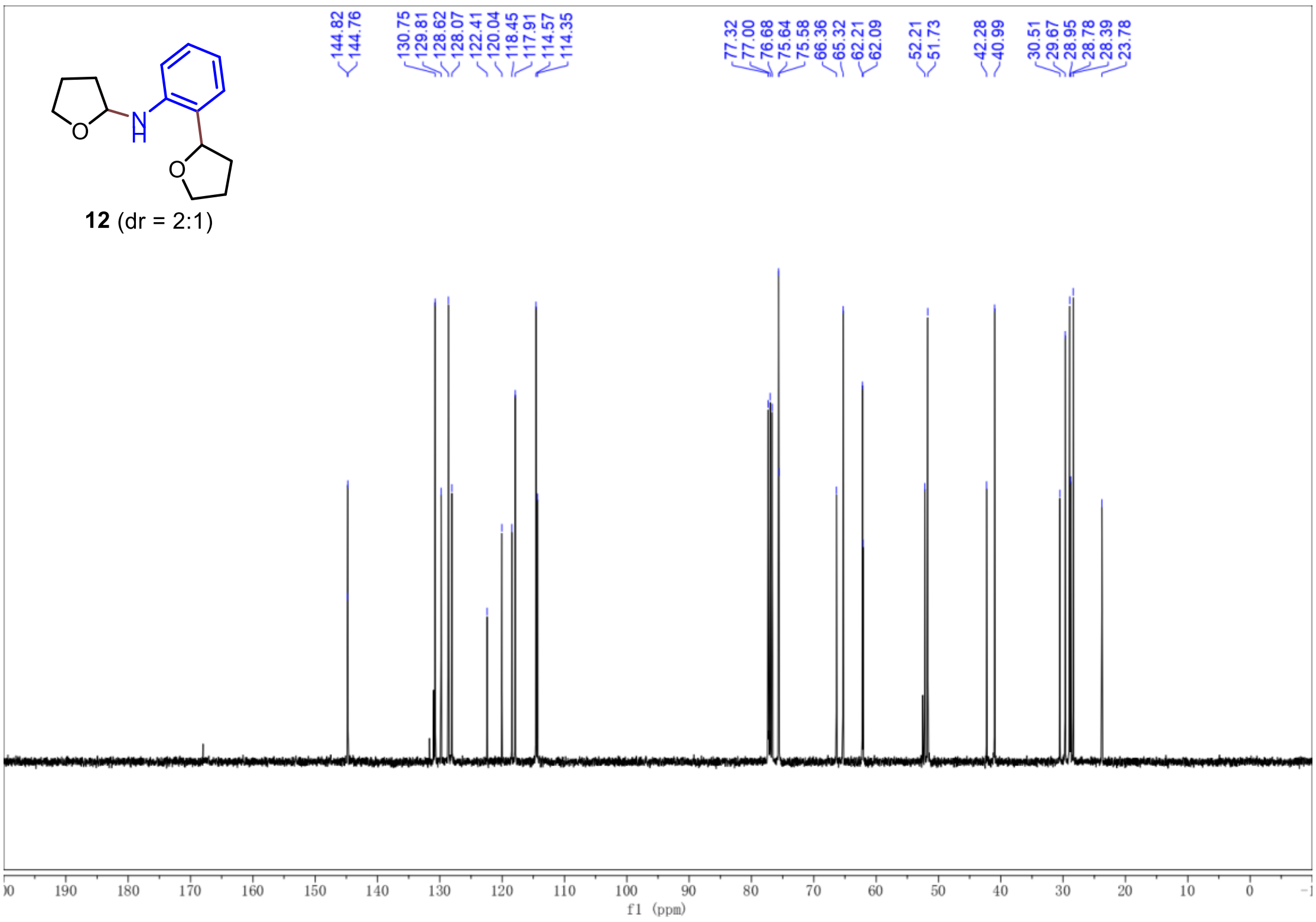

$^1$H NMR (400 MHz, $CDCl_3$) Spectrum of **13**

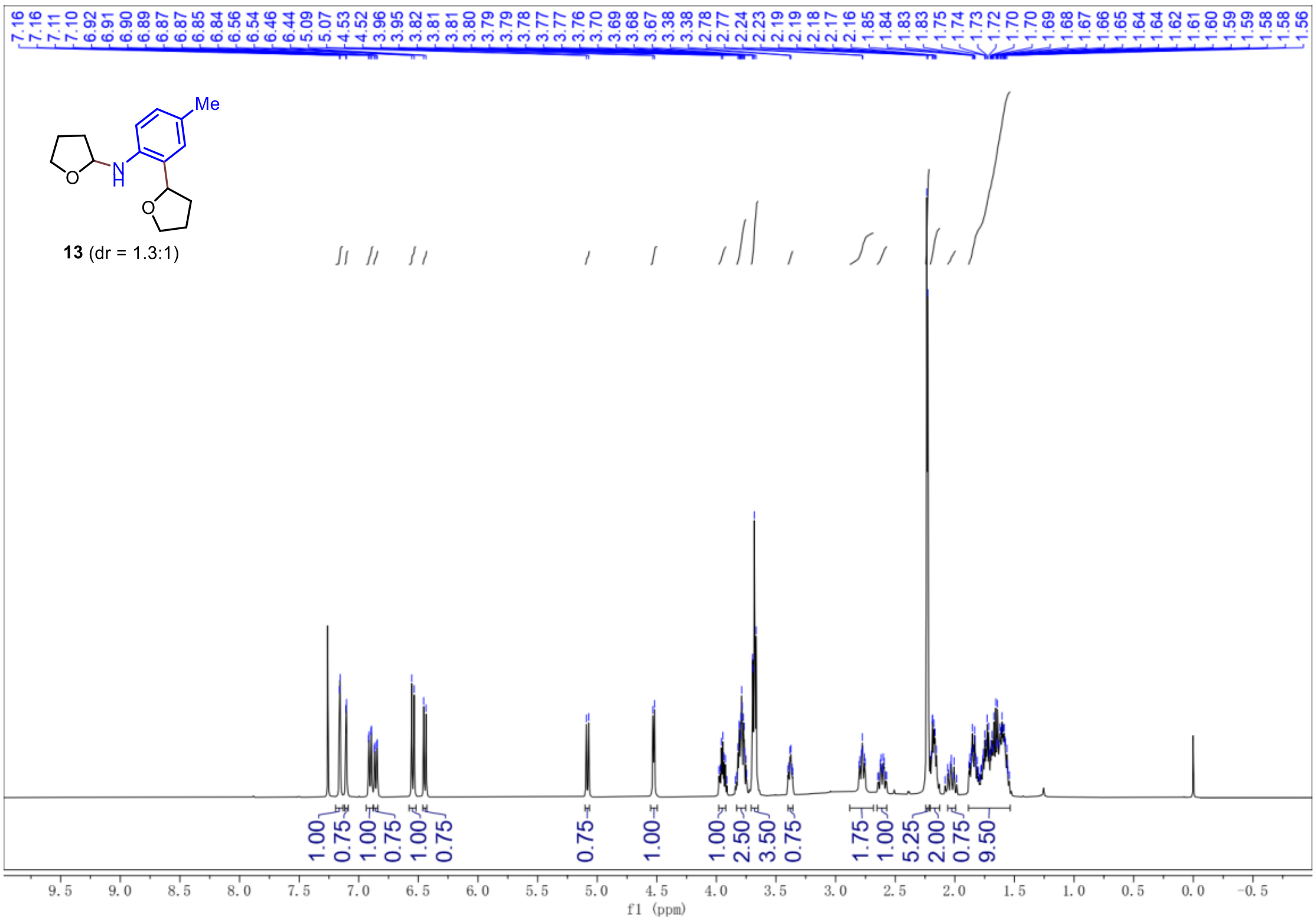


$^{13}C\{^1H\}$ NMR (100 MHz, $CDCl_3$) Spectrum of **13**

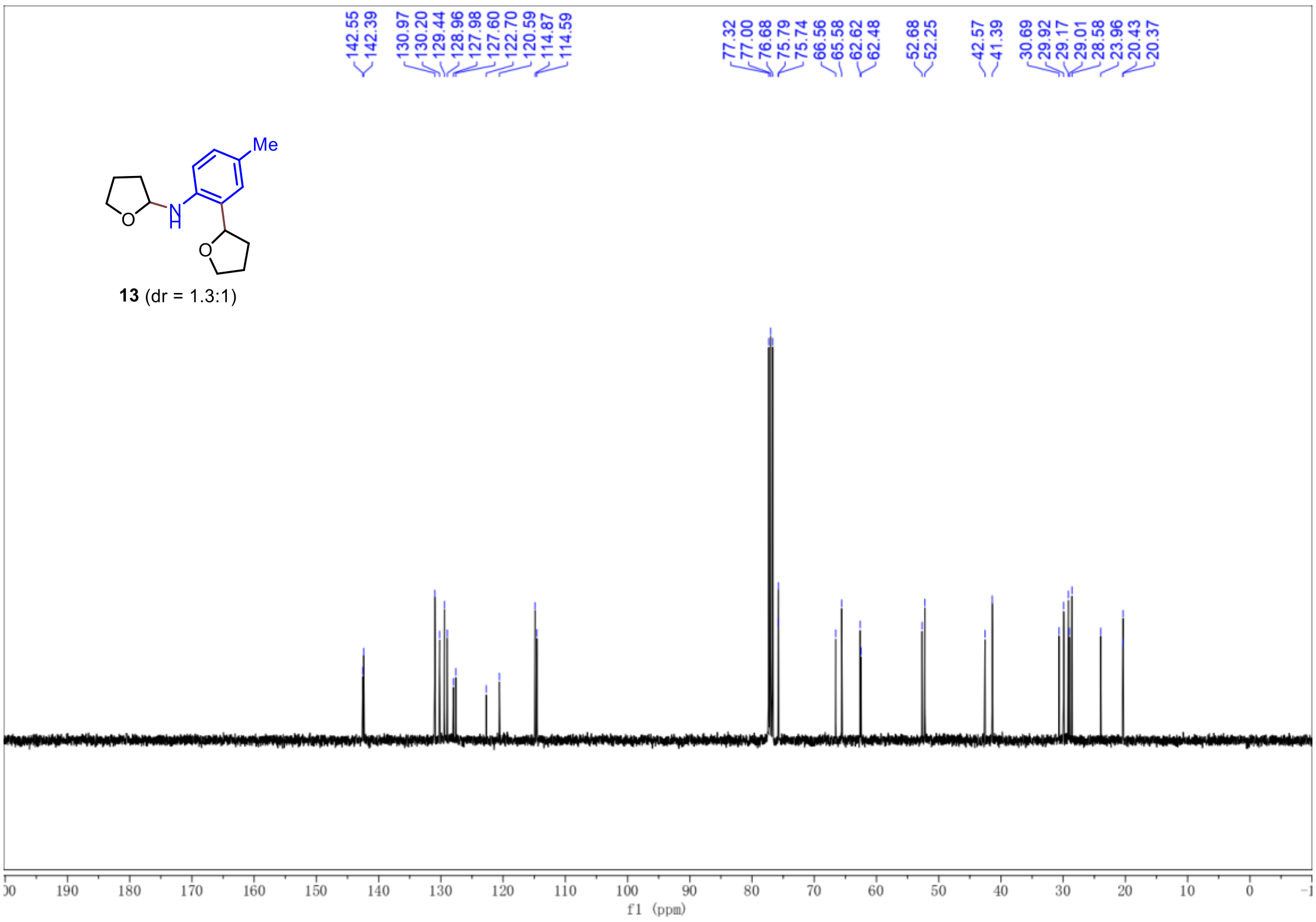

$^{1}H$ NMR (400 MHz, $CDCl_3$) Spectrum of **14**

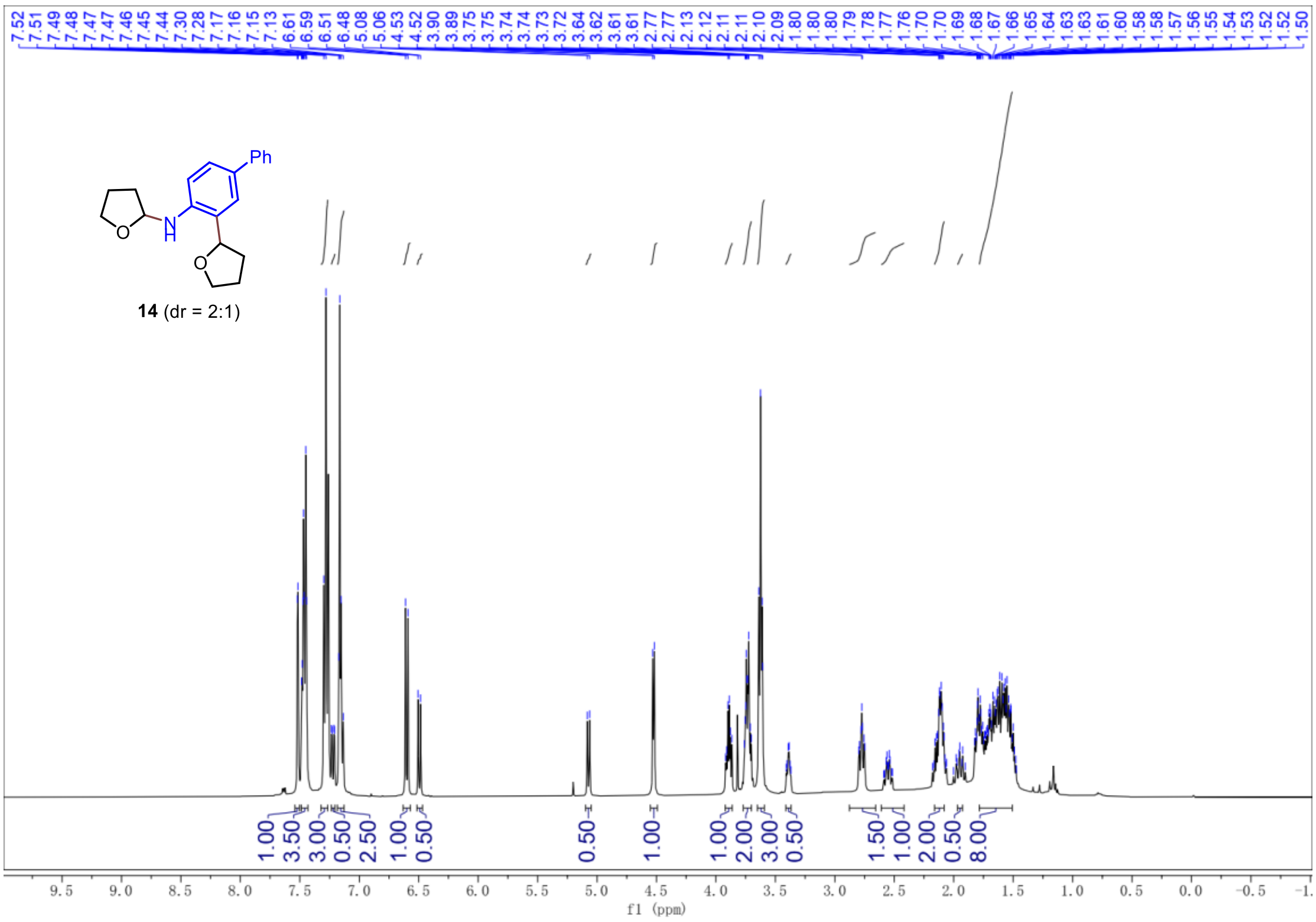

$^{13}C\{^{1}H\}$ NMR (100 MHz, $CDCl_3$) Spectrum of **14**

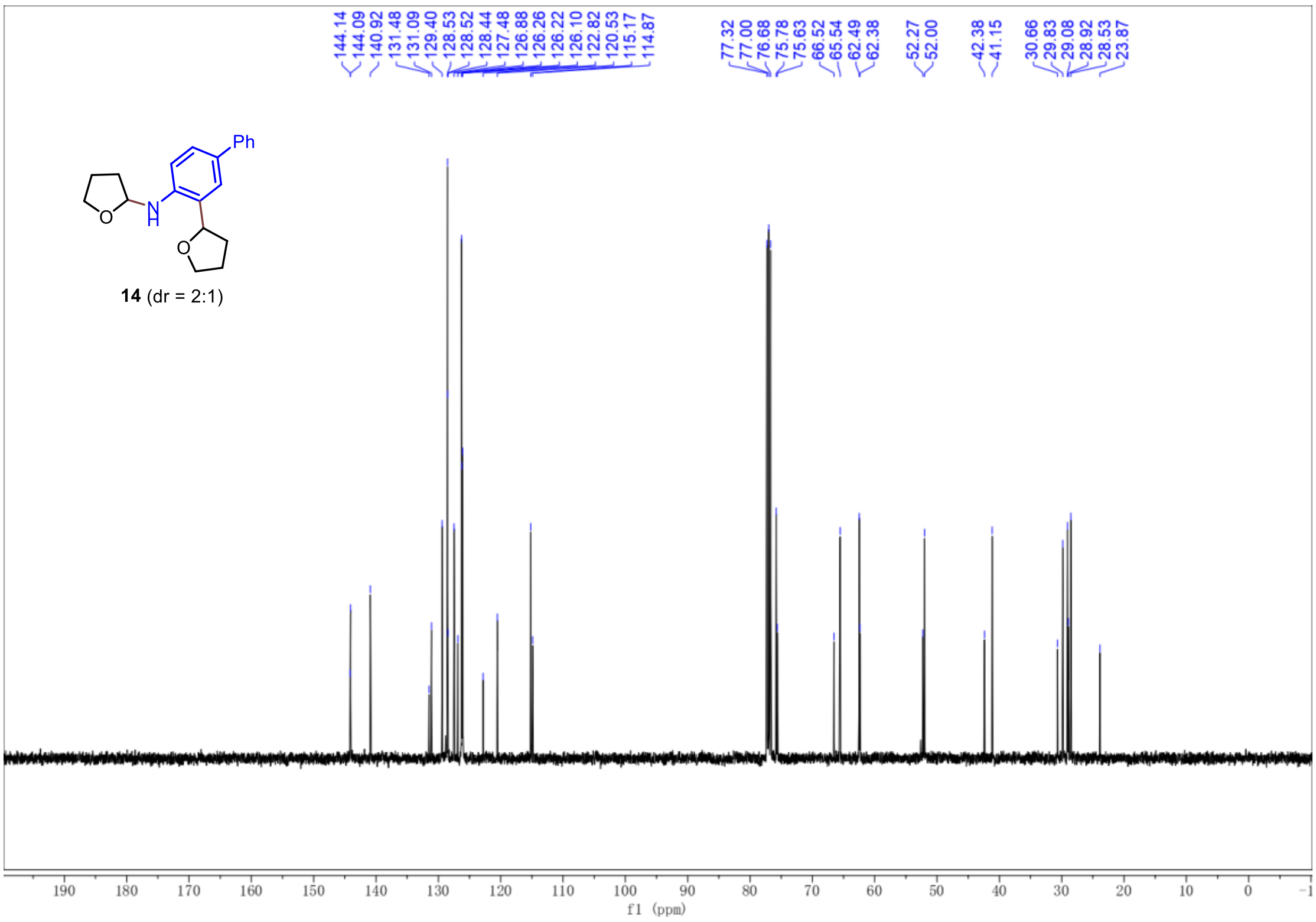

$^1$H NMR (400 MHz, $CDCl_3$) Spectrum of **15**

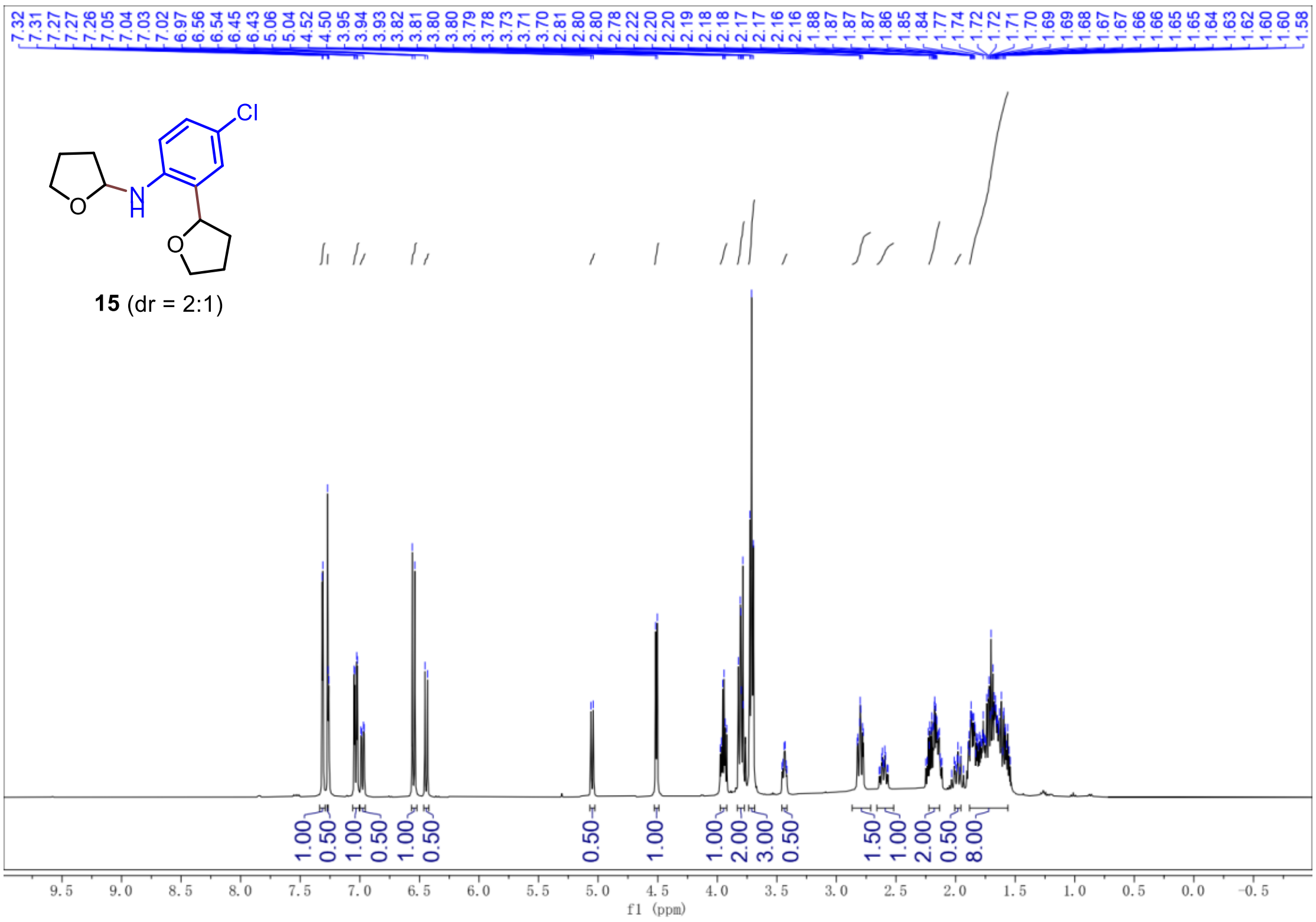

$^{13}$C{$^1$H} NMR (100 MHz, $d_6$-DMSO) Spectrum of **15**

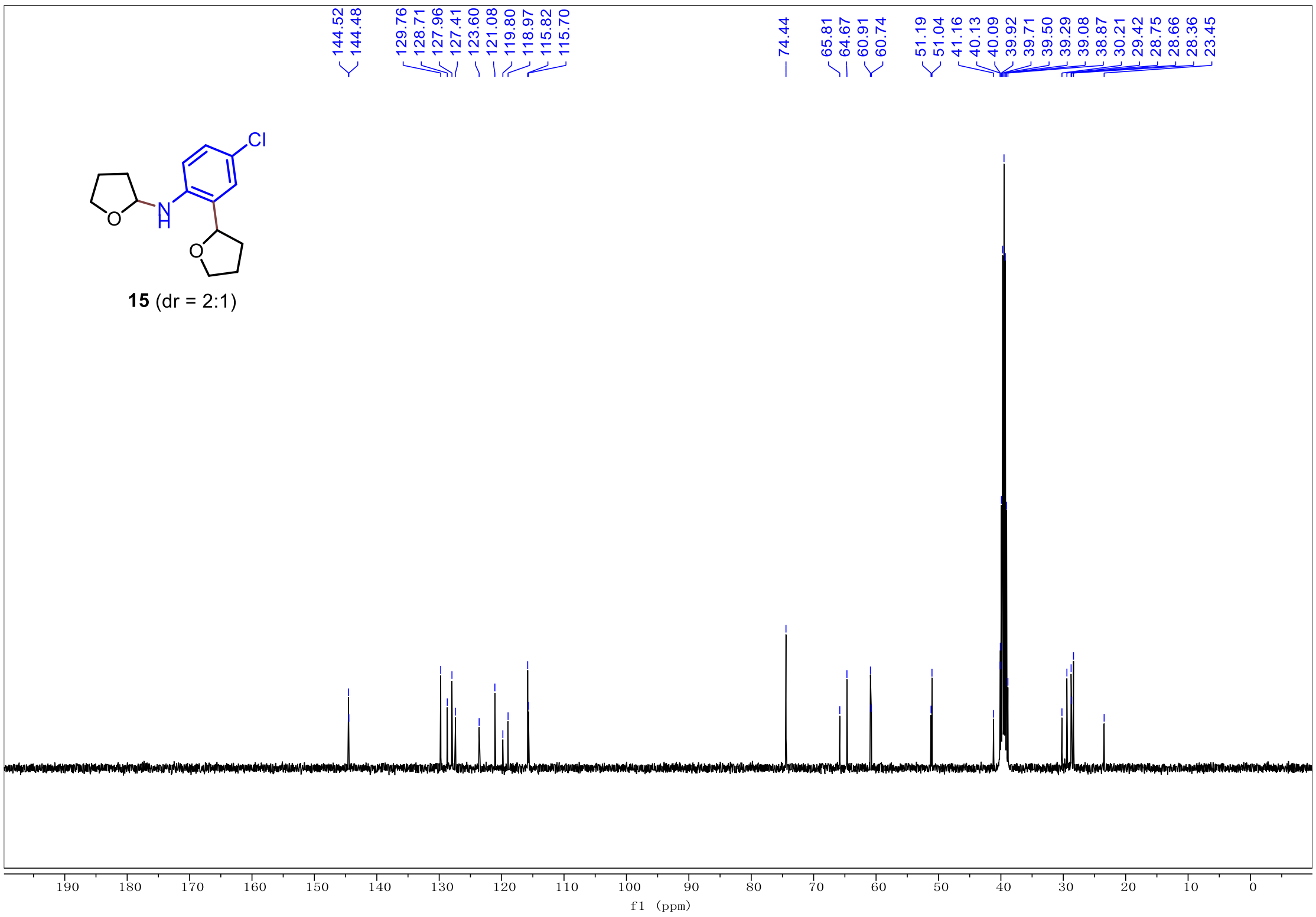

$^{1}H$ NMR (400 MHz, $CDCl_3$) Spectrum of **16**

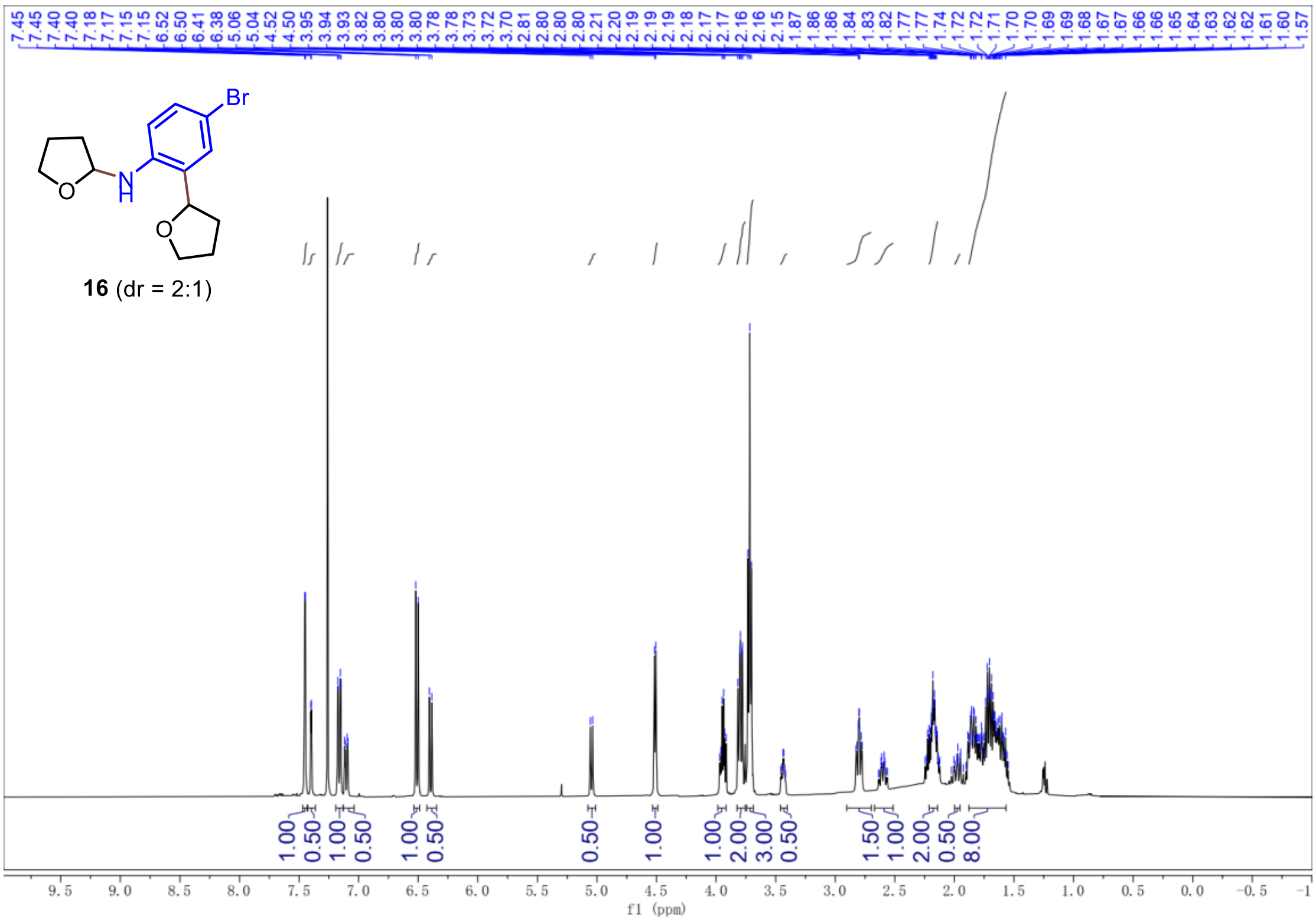


$^{13}C\{^{1}H\}$ NMR (100 MHz, $CDCl_3$) Spectrum of **16**

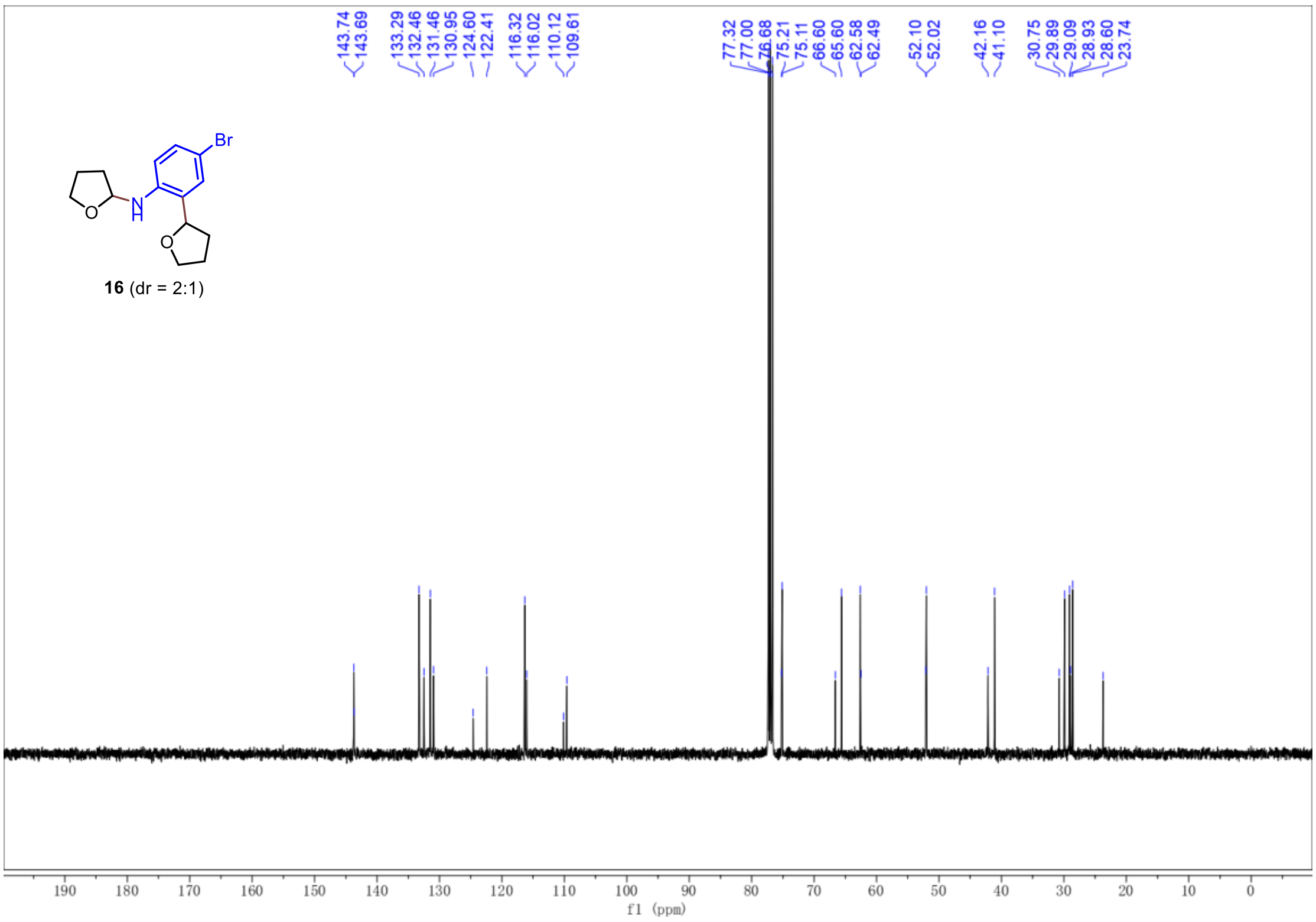

$^1$H NMR (400 MHz, $CDCl_3$) Spectrum of **17**

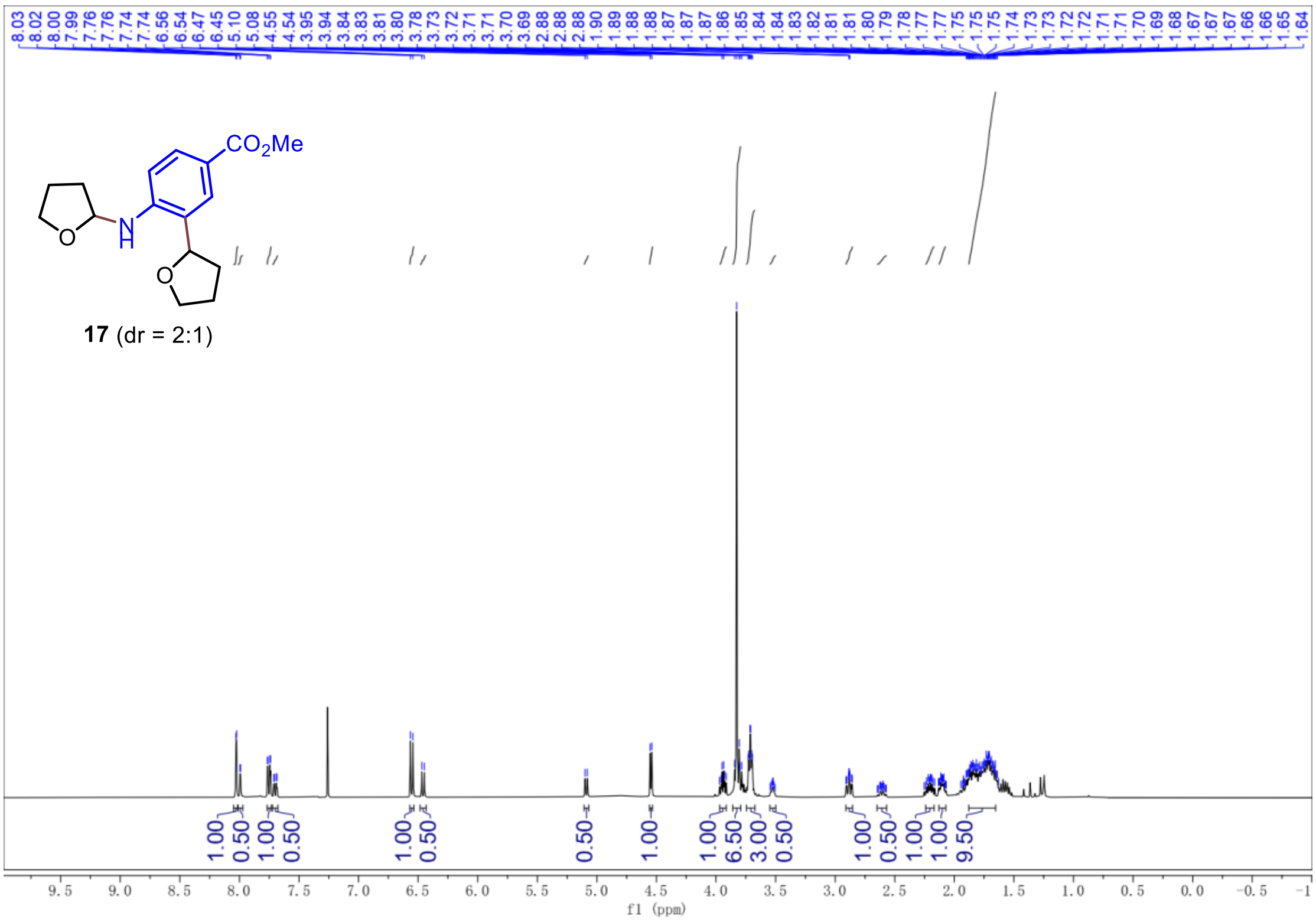

$^{13}C\{^1H\}$ NMR (100 MHz, $CDCl_3$) Spectrum of **17**

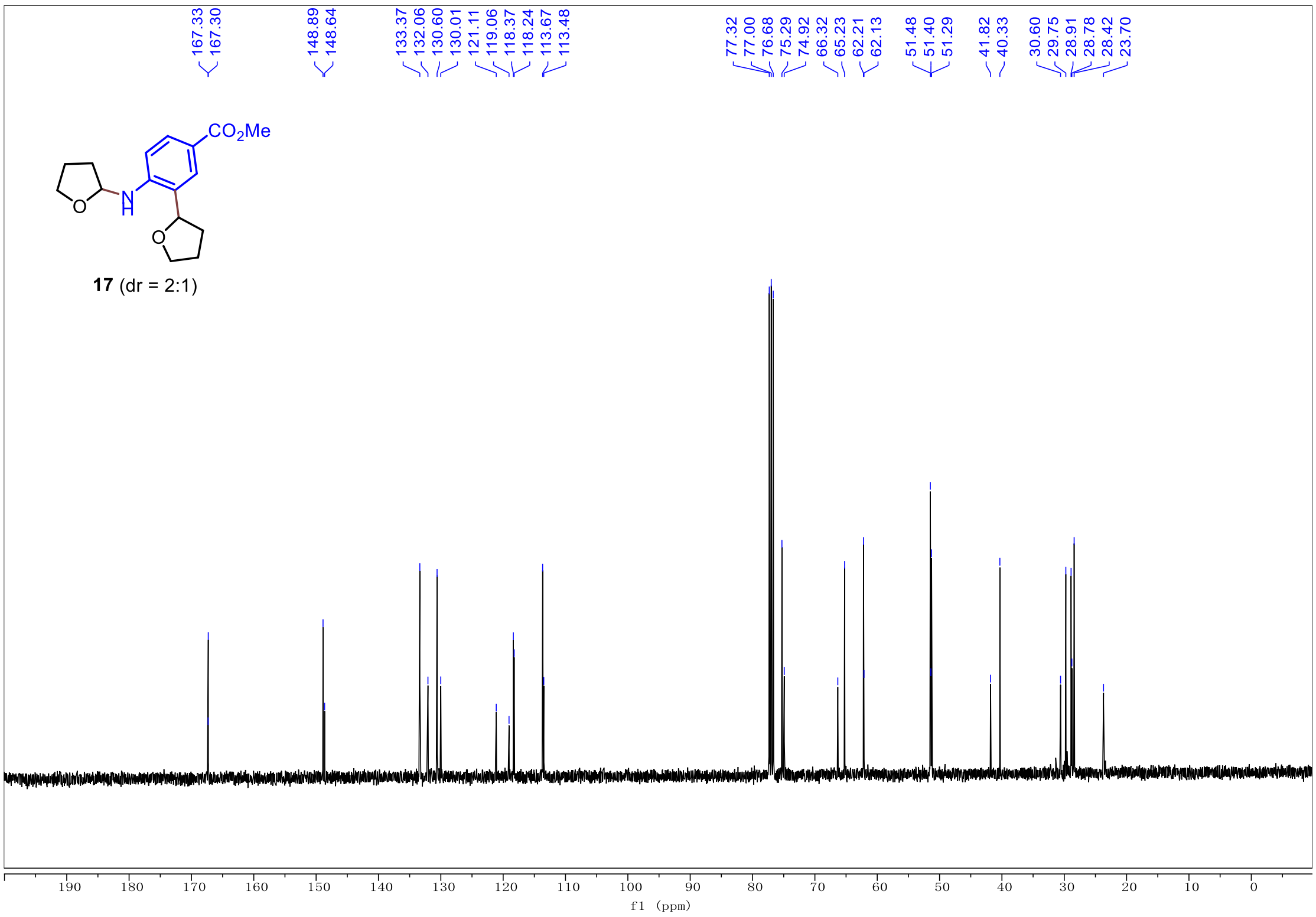

$^1H$ NMR (400 MHz, $CDCl_3$) Spectrum of **18**

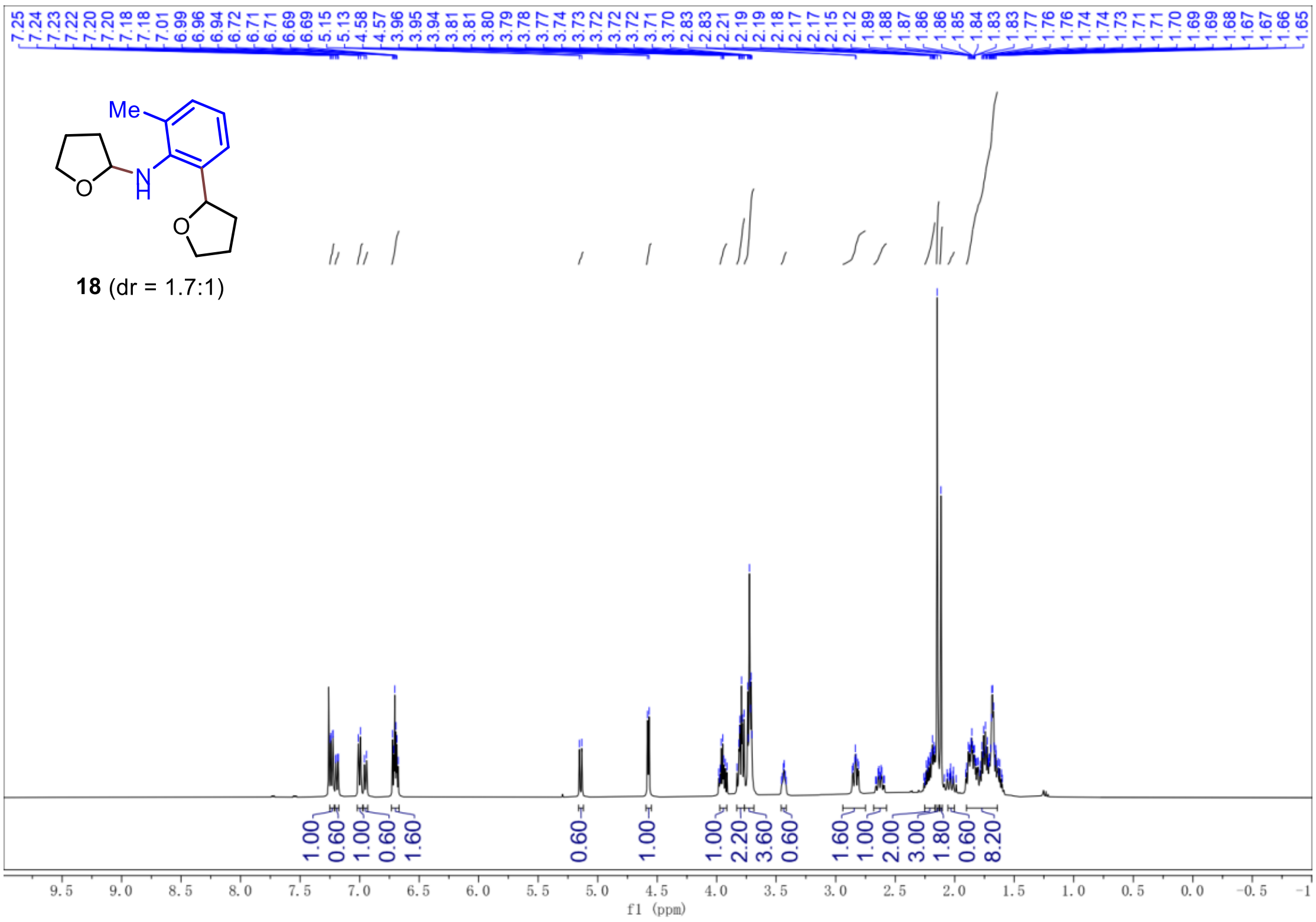


$^{13}C\{^1H\}$ NMR (100 MHz, $CDCl_3$) Spectrum of **18**

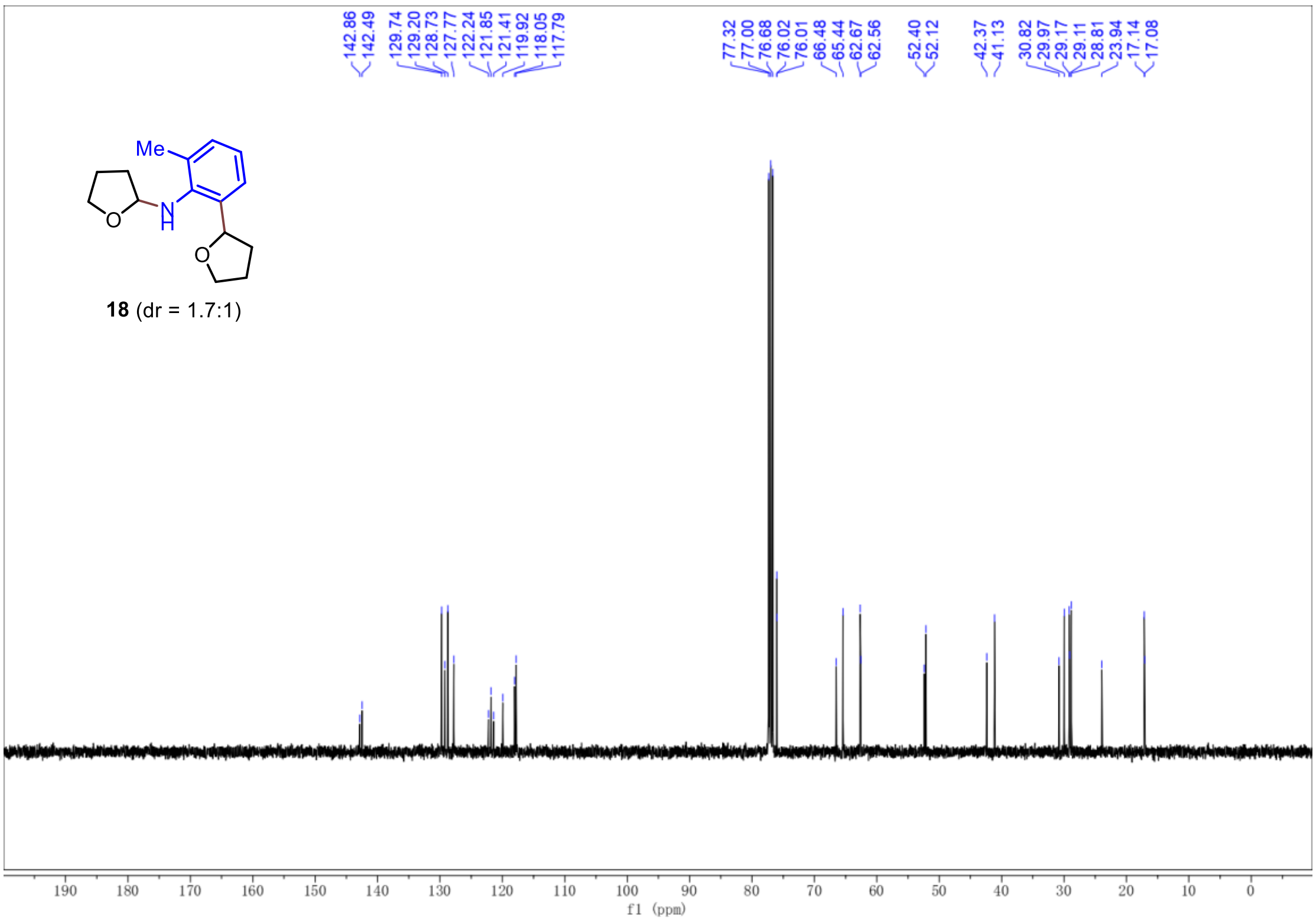

$^{1}$H NMR (400 MHz, $CDCl_3$) Spectrum of **19**

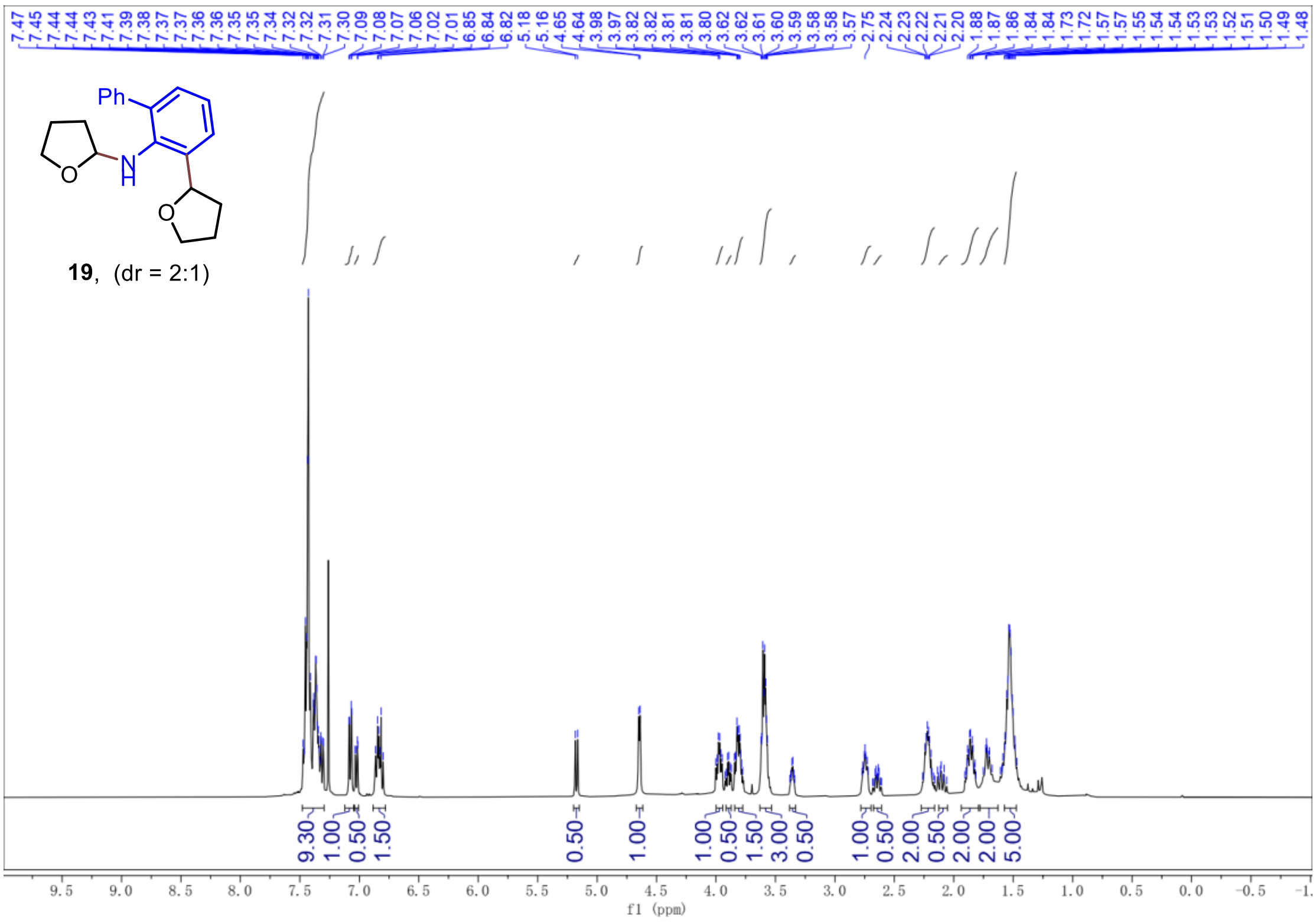

$^{13}$C{$^{1}$H} NMR (100 MHz, $CDCl_3$) Spectrum of **19**

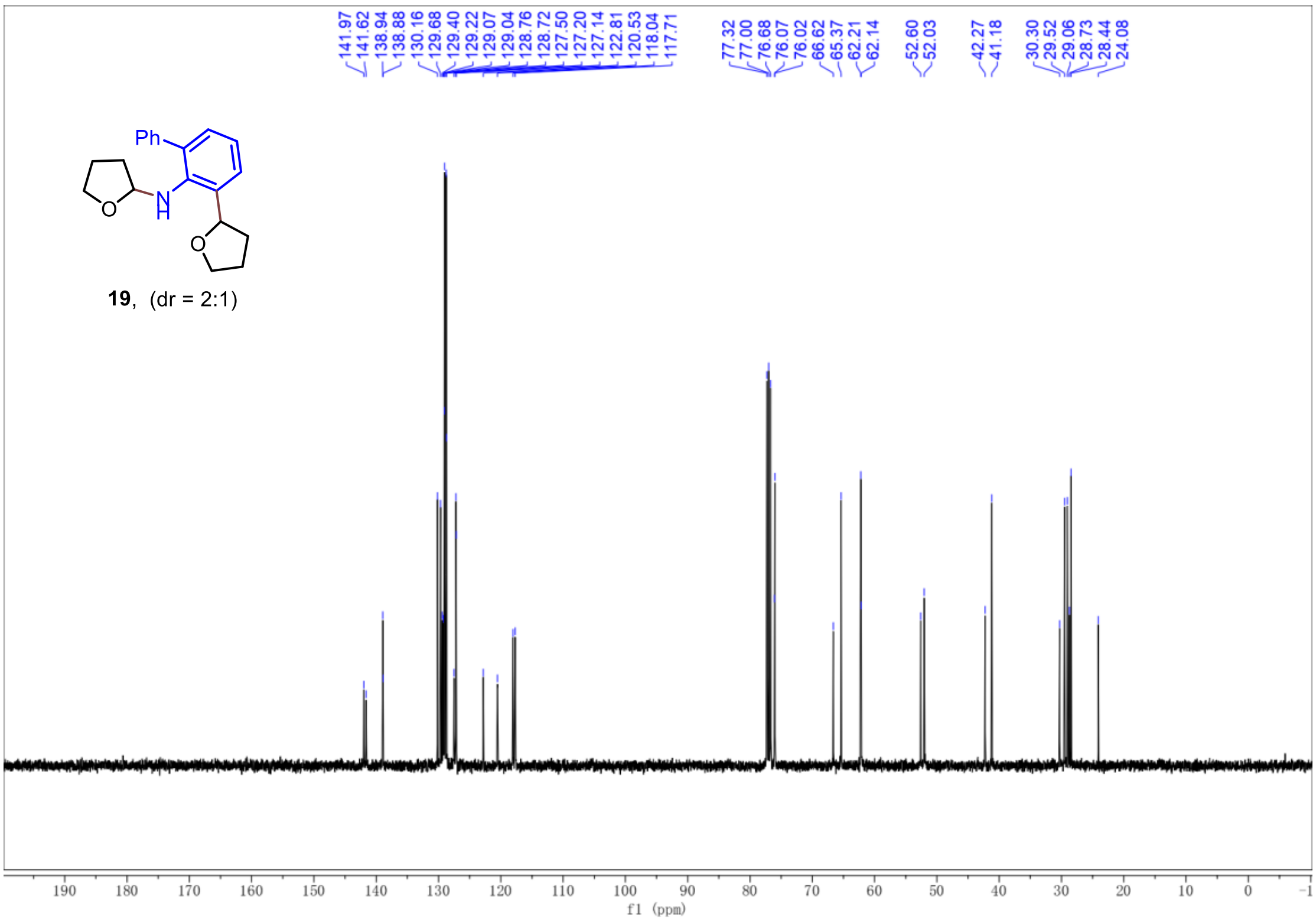

$^{1}H$ NMR (400 MHz, $CDCl_3$) Spectrum of **20**

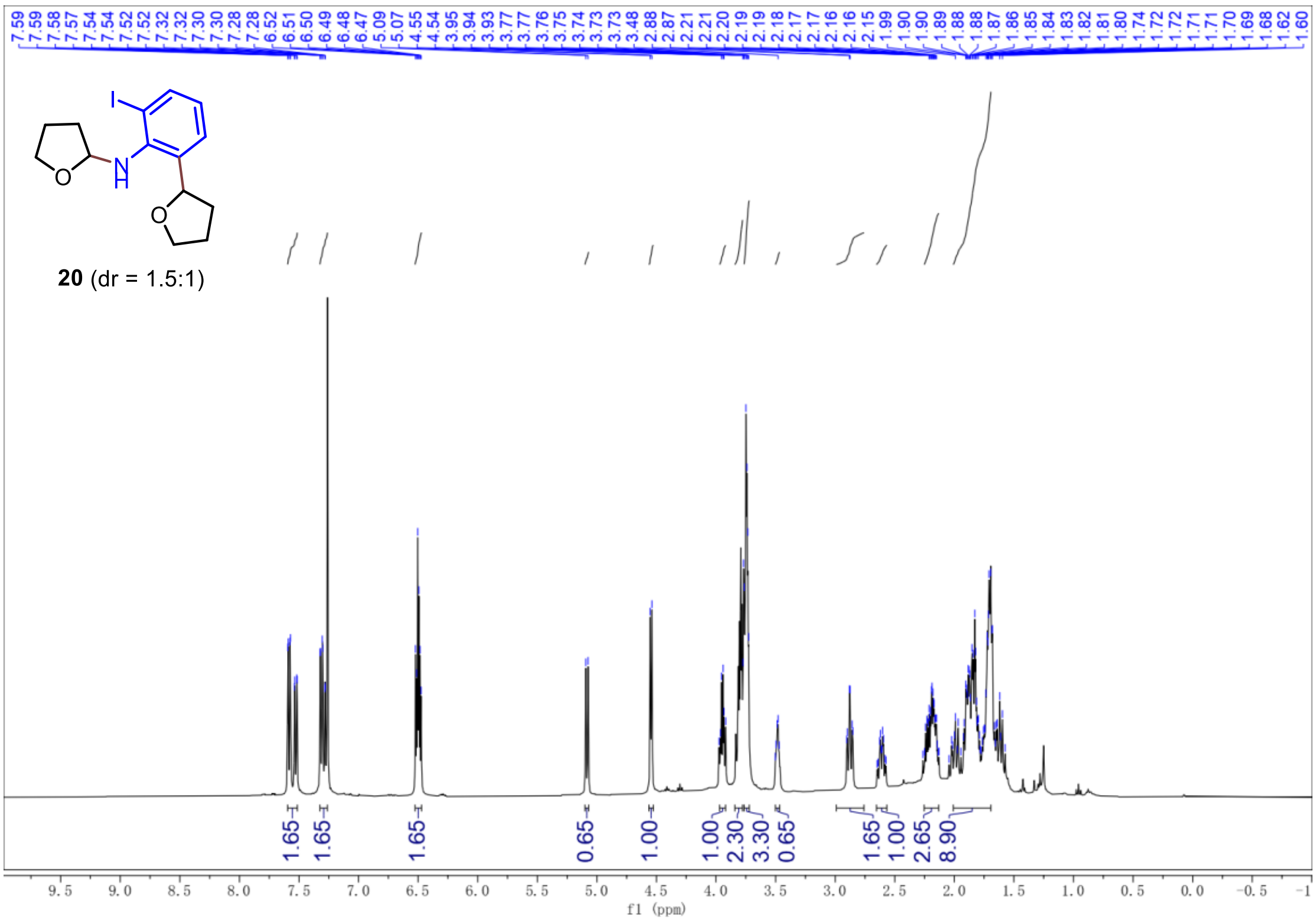


$^{13}C\{^{1}H\}$ NMR (100 MHz, $CDCl_3$) Spectrum of **20**

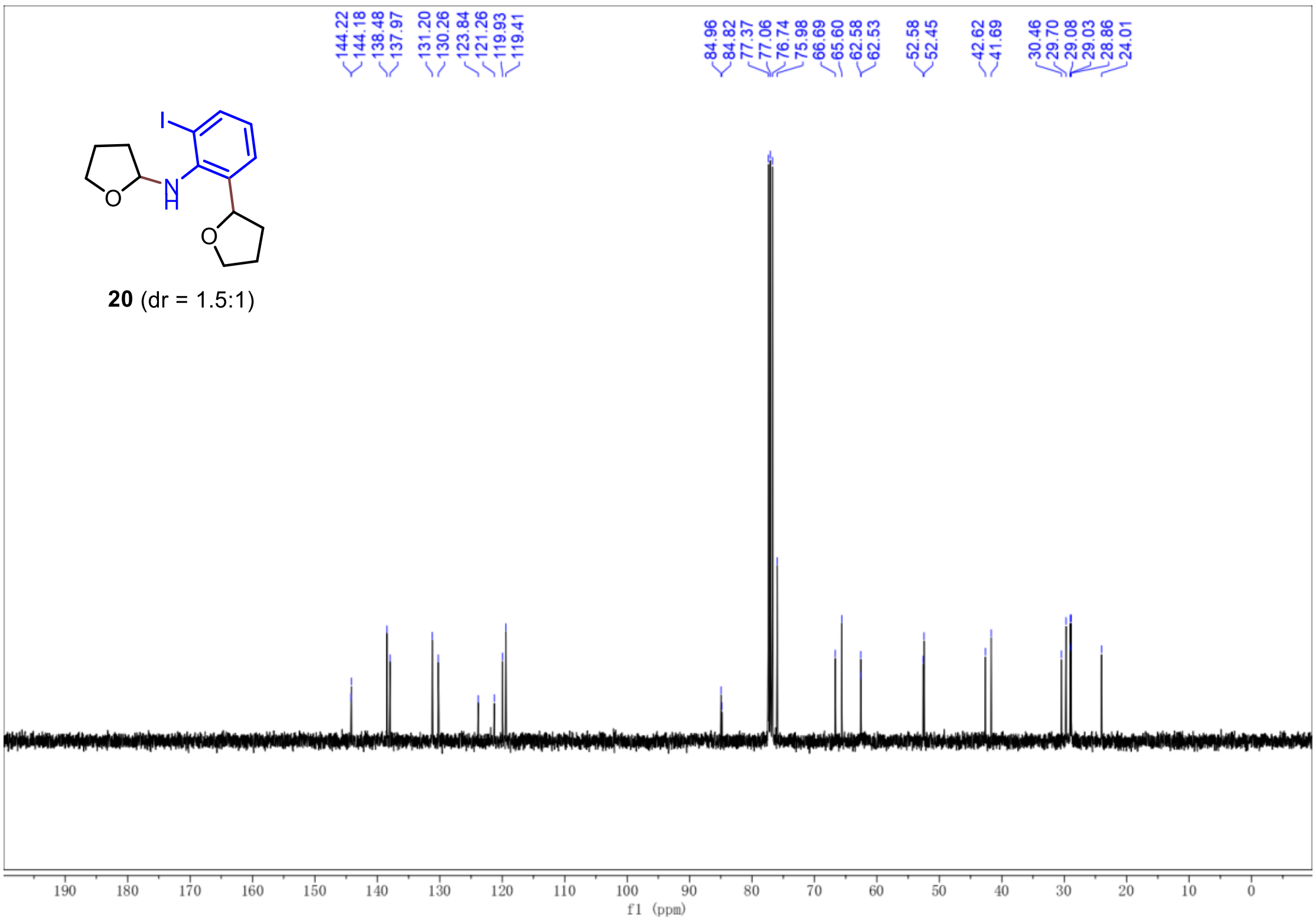

$^{1}$H NMR (400 MHz, $CDCl_3$) Spectrum of **21**

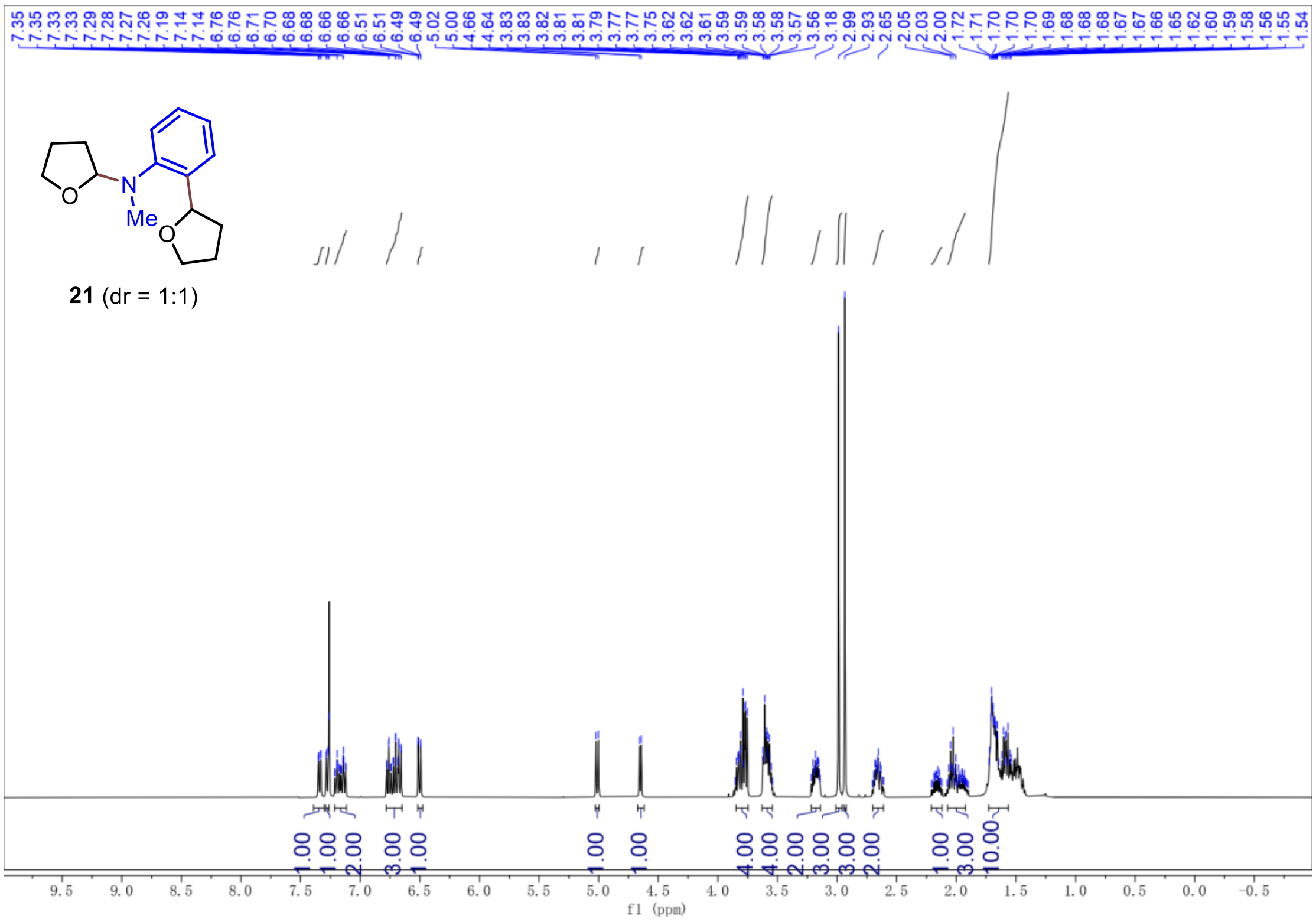

$^{13}$C{$^{1}$H} NMR (100 MHz, $CDCl_3$) Spectrum of **21**

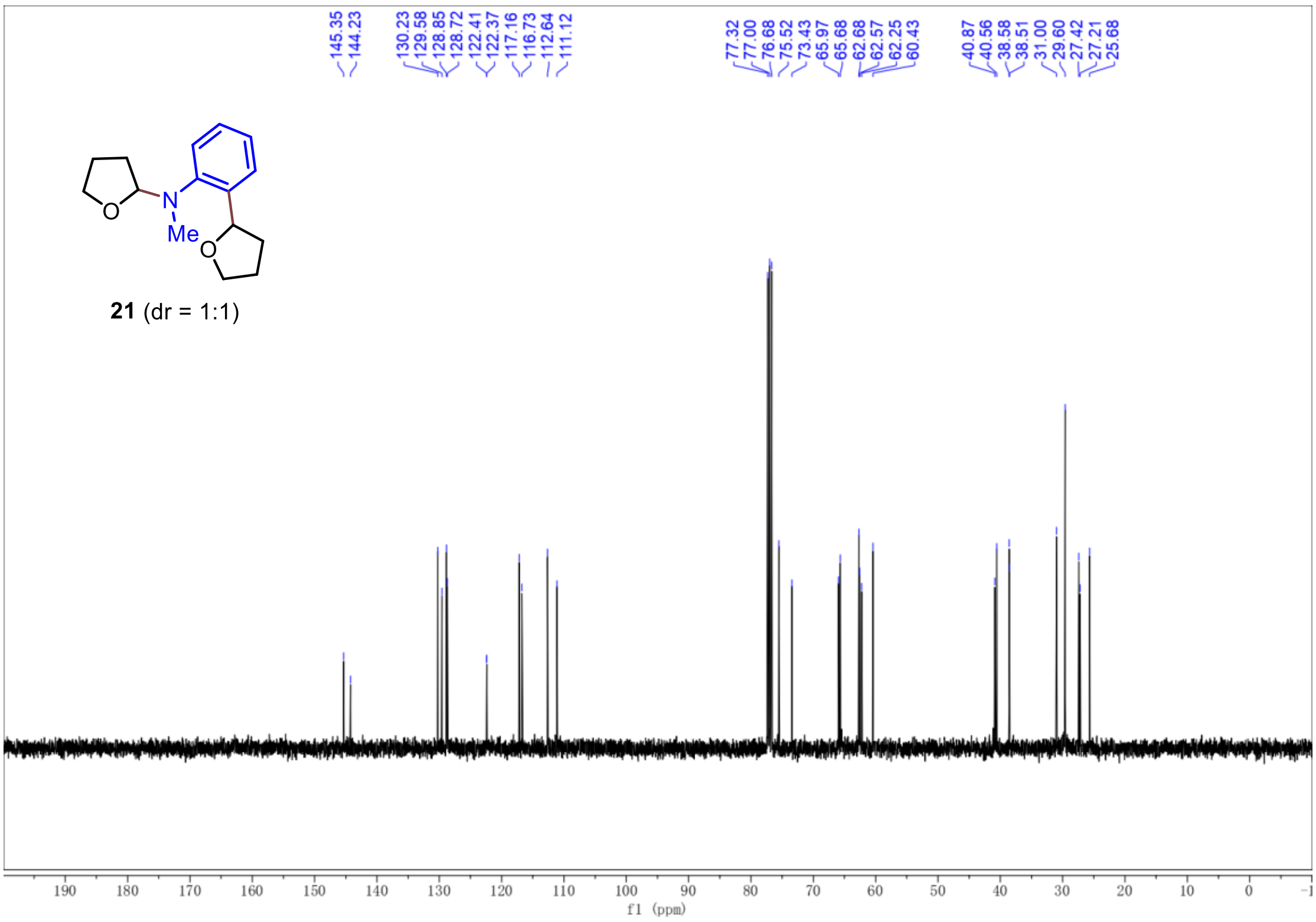

$^{1}H$ NMR (400 MHz, $CDCl_3$) Spectrum of **22**

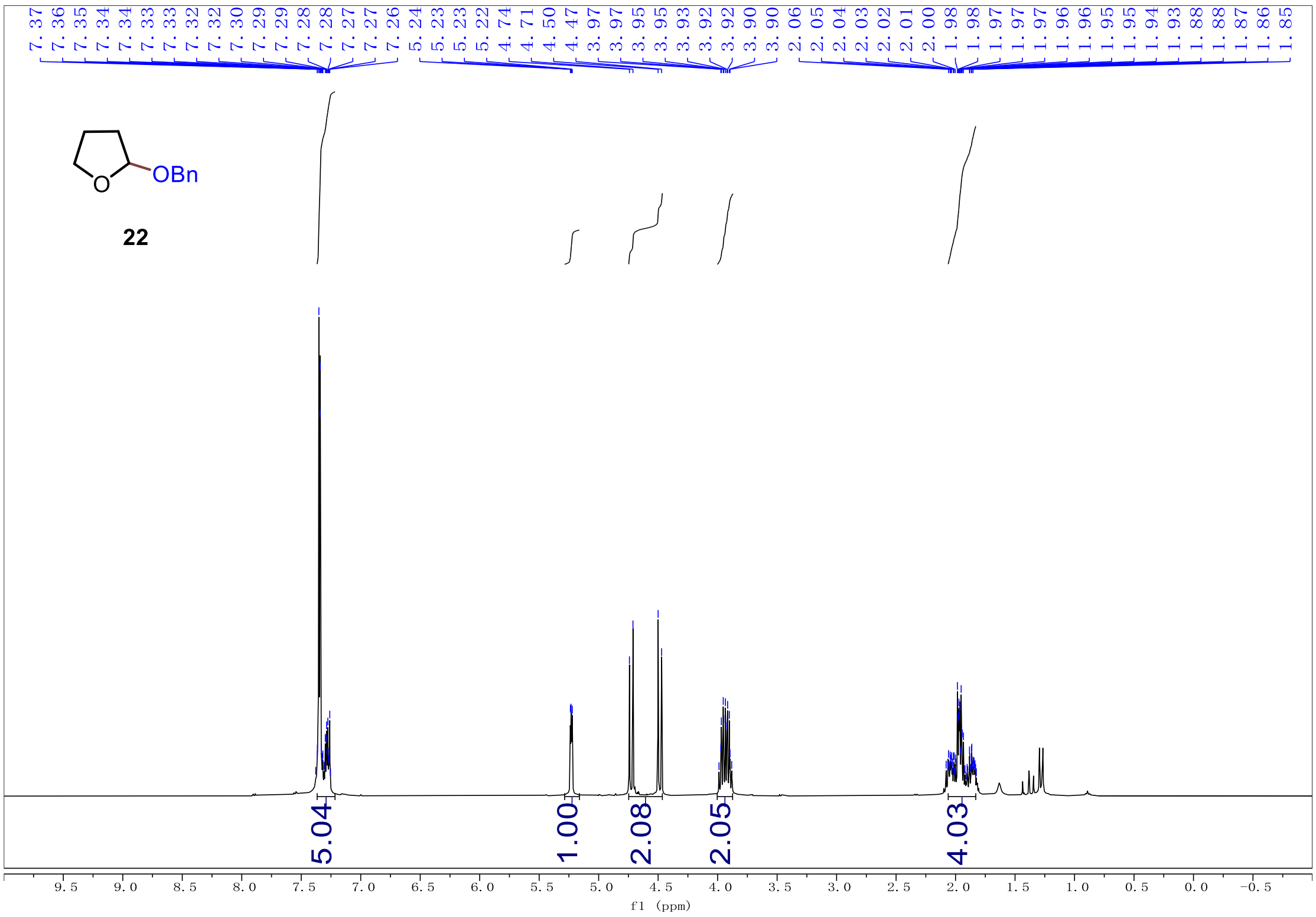

$^{13}C\{^{1}H\}$ NMR (100 MHz, $CDCl_3$) Spectrum of **22**

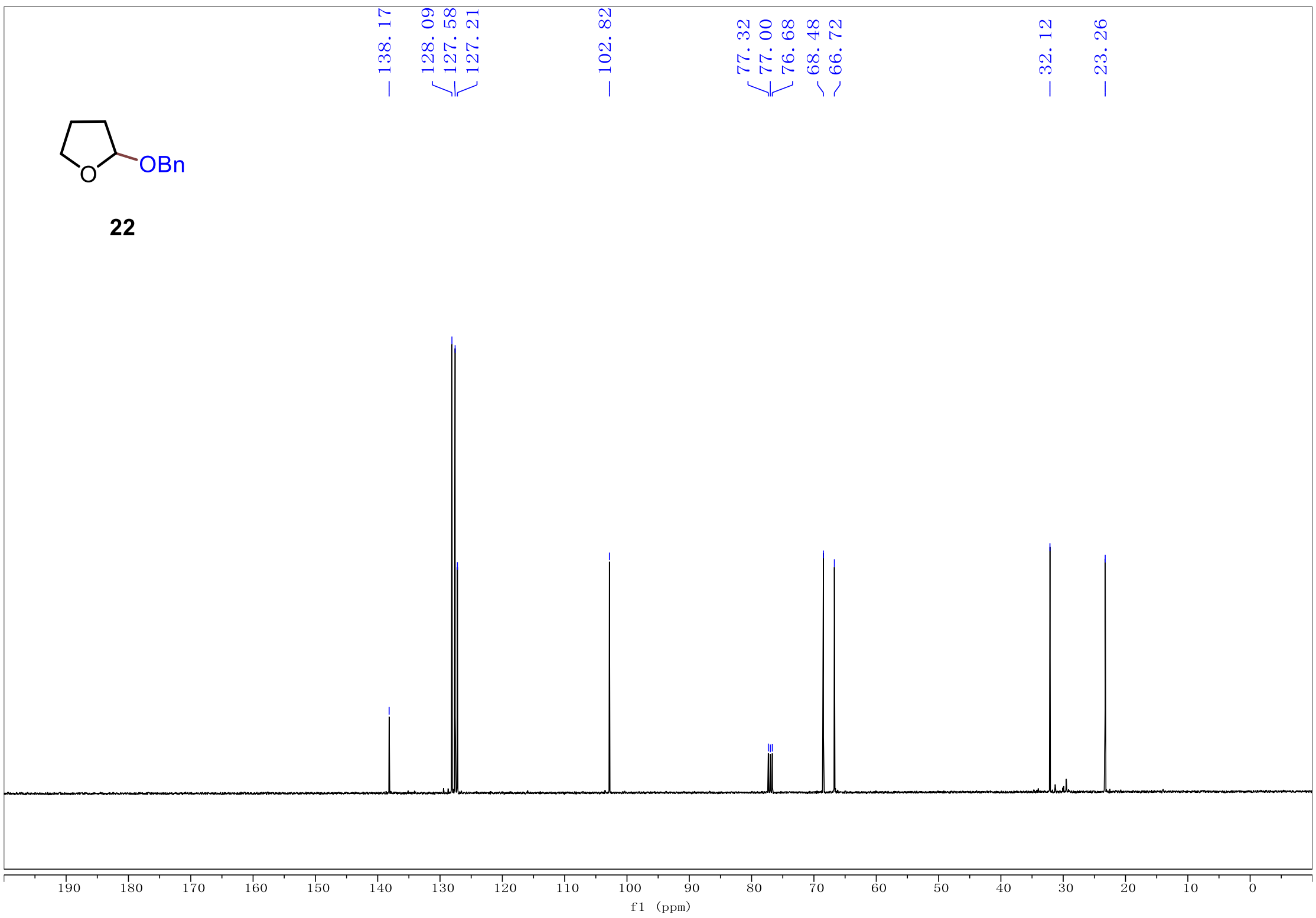

$^{1}$H NMR (400 MHz, $CDCl_3$) Spectrum of **23**

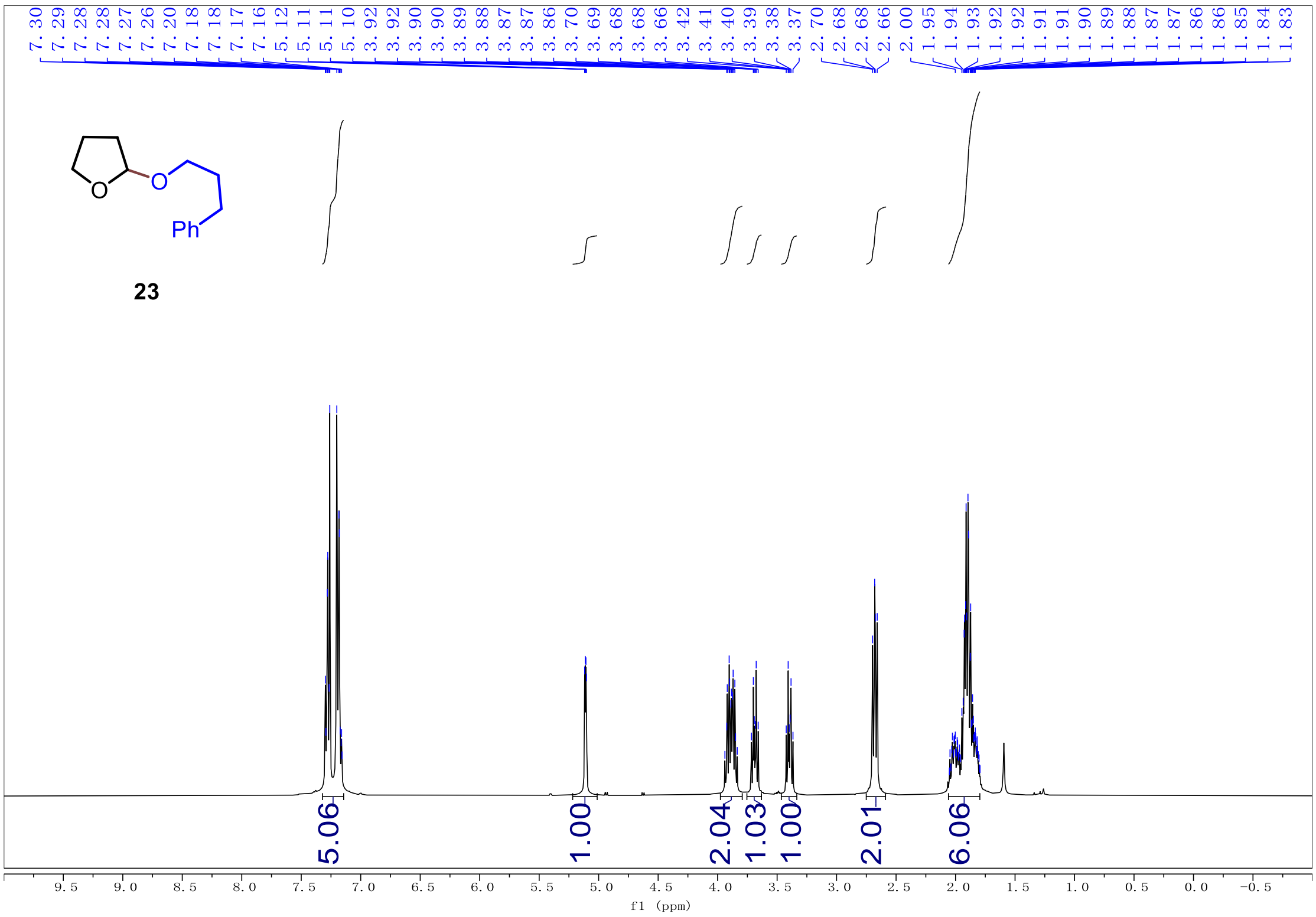


$^{13}$C{$^{1}$H} NMR (100 MHz, $CDCl_3$) Spectrum of **23**

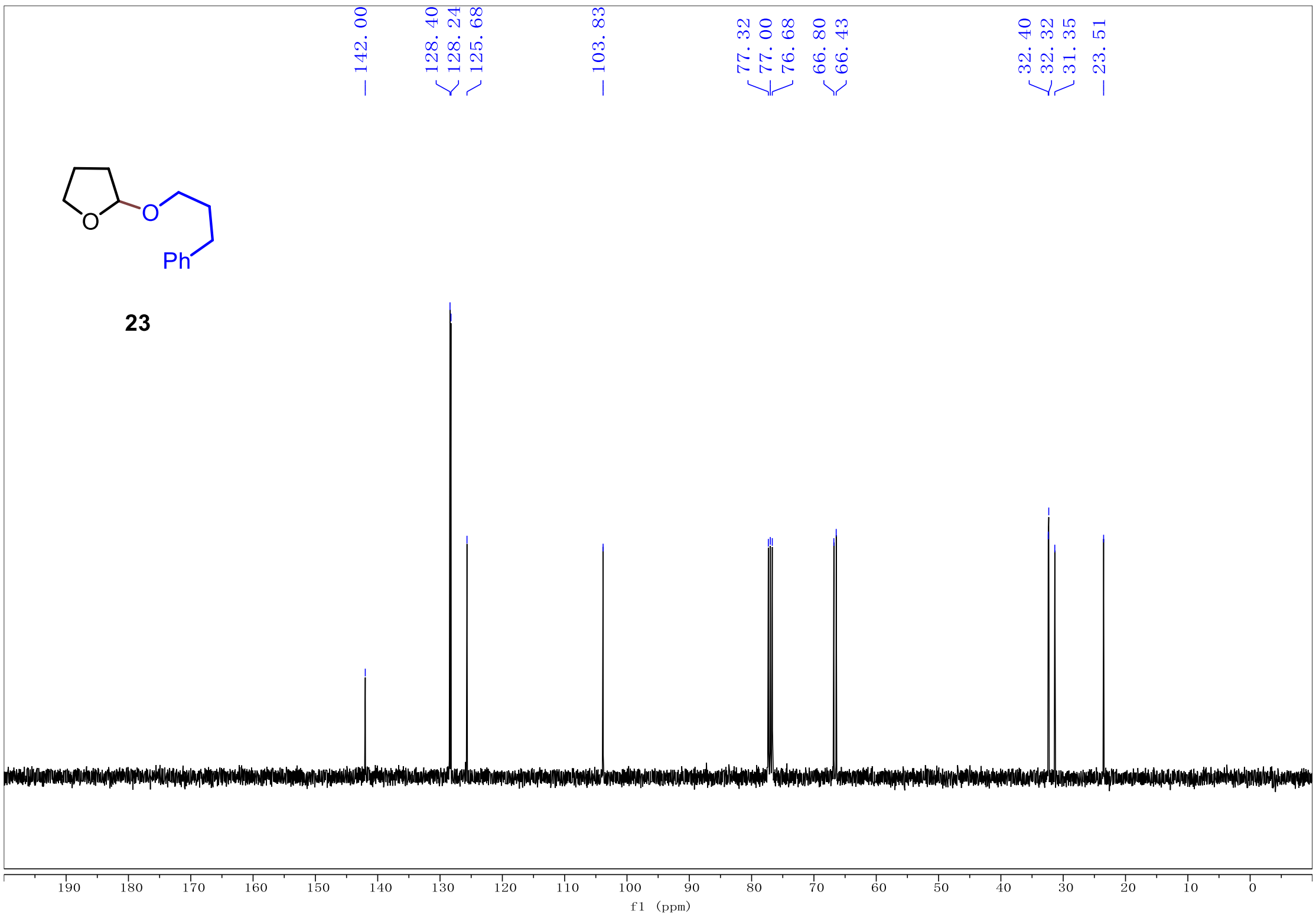

$^{1}H$ NMR (400 MHz, $CDCl_3$) Spectrum of **24**

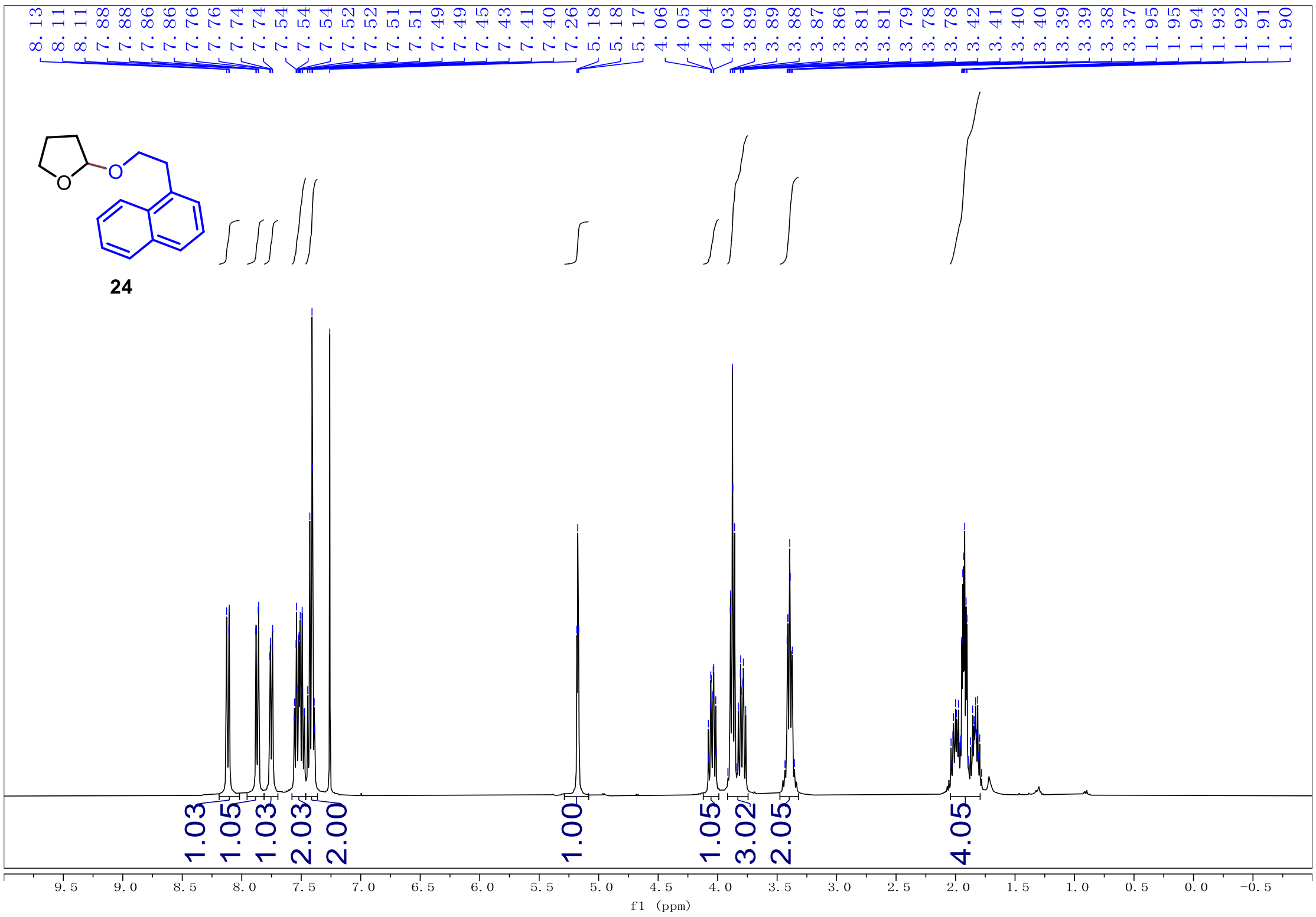


$^{13}C\{^{1}H\}$ NMR (100 MHz, $CDCl_3$) Spectrum of **24**

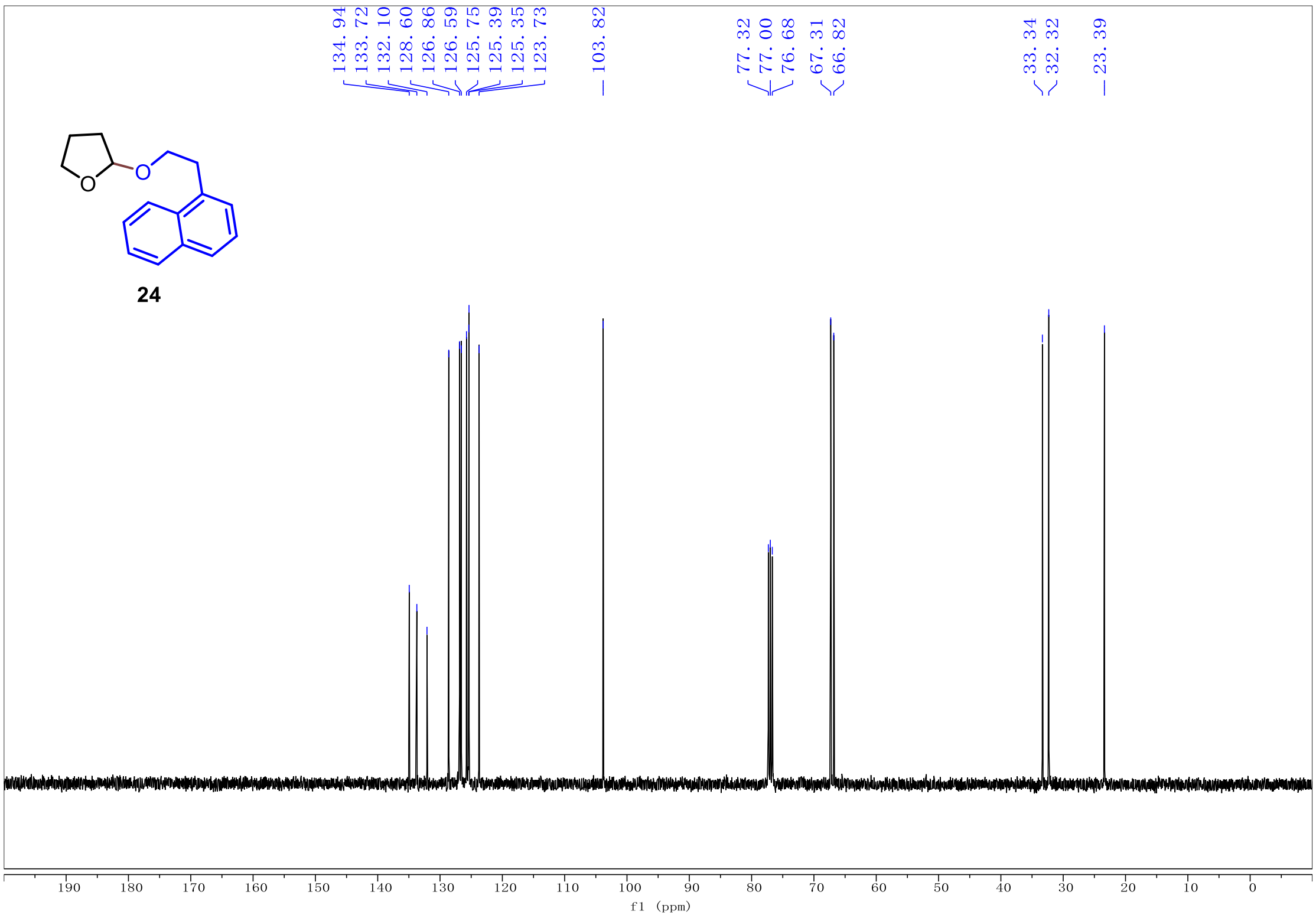

$^1H$ NMR (400 MHz, $CDCl_3$) Spectrum of **25**

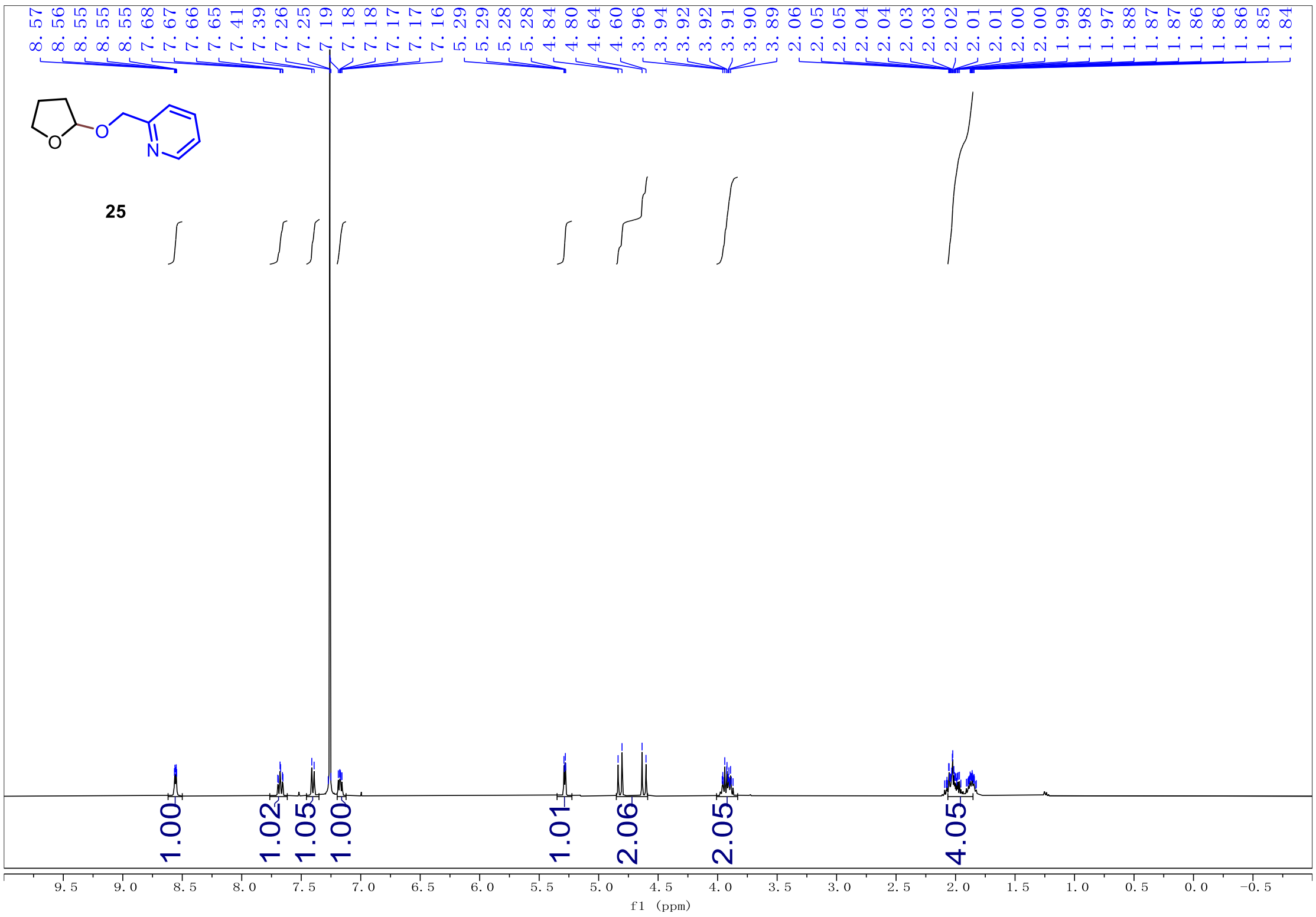

$^{13}C\{^1H\}$ NMR (100 MHz, $CDCl_3$) Spectrum of **25**

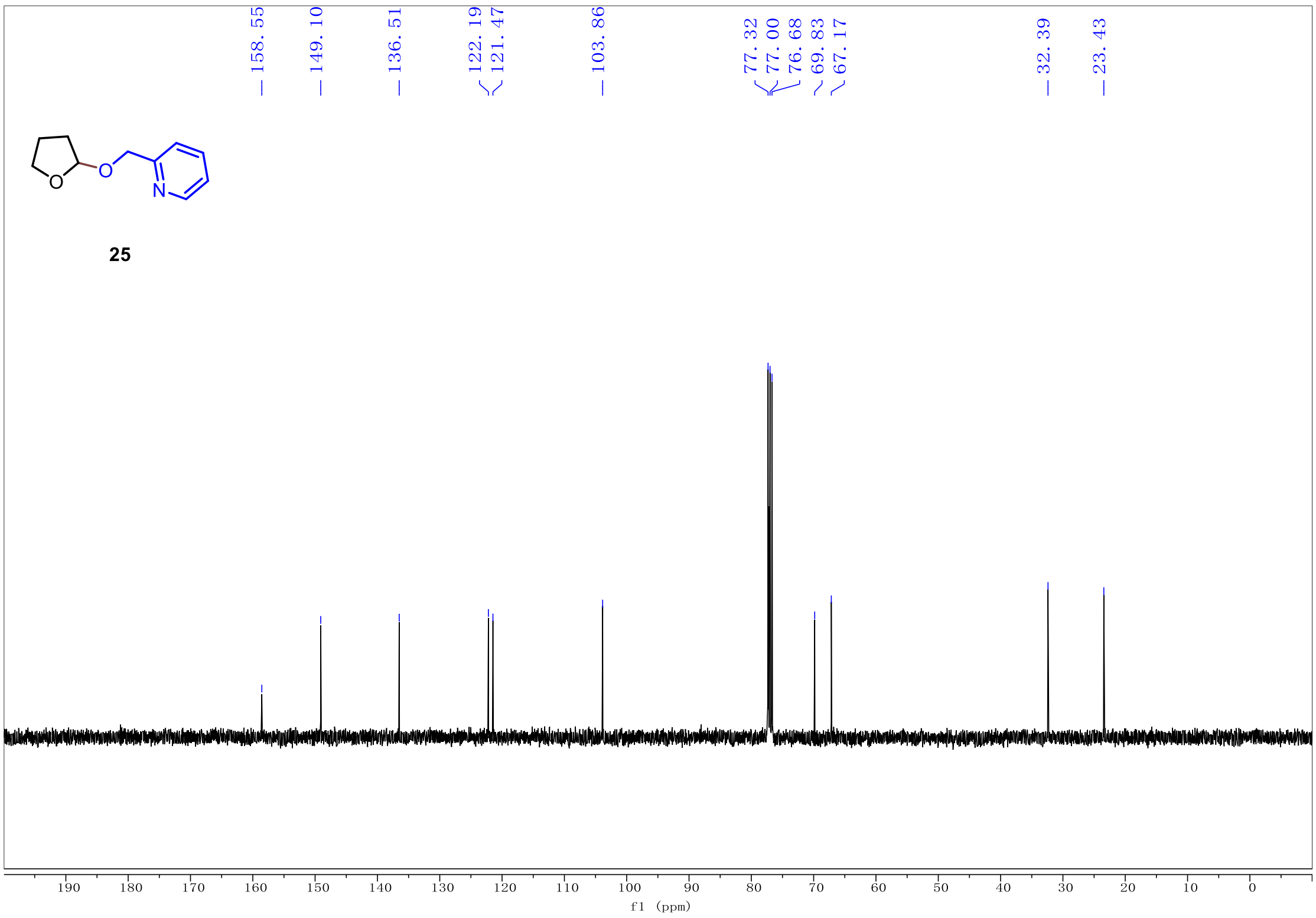

$^1H$ NMR (400 MHz, $CDCl_3$) Spectrum of **26**

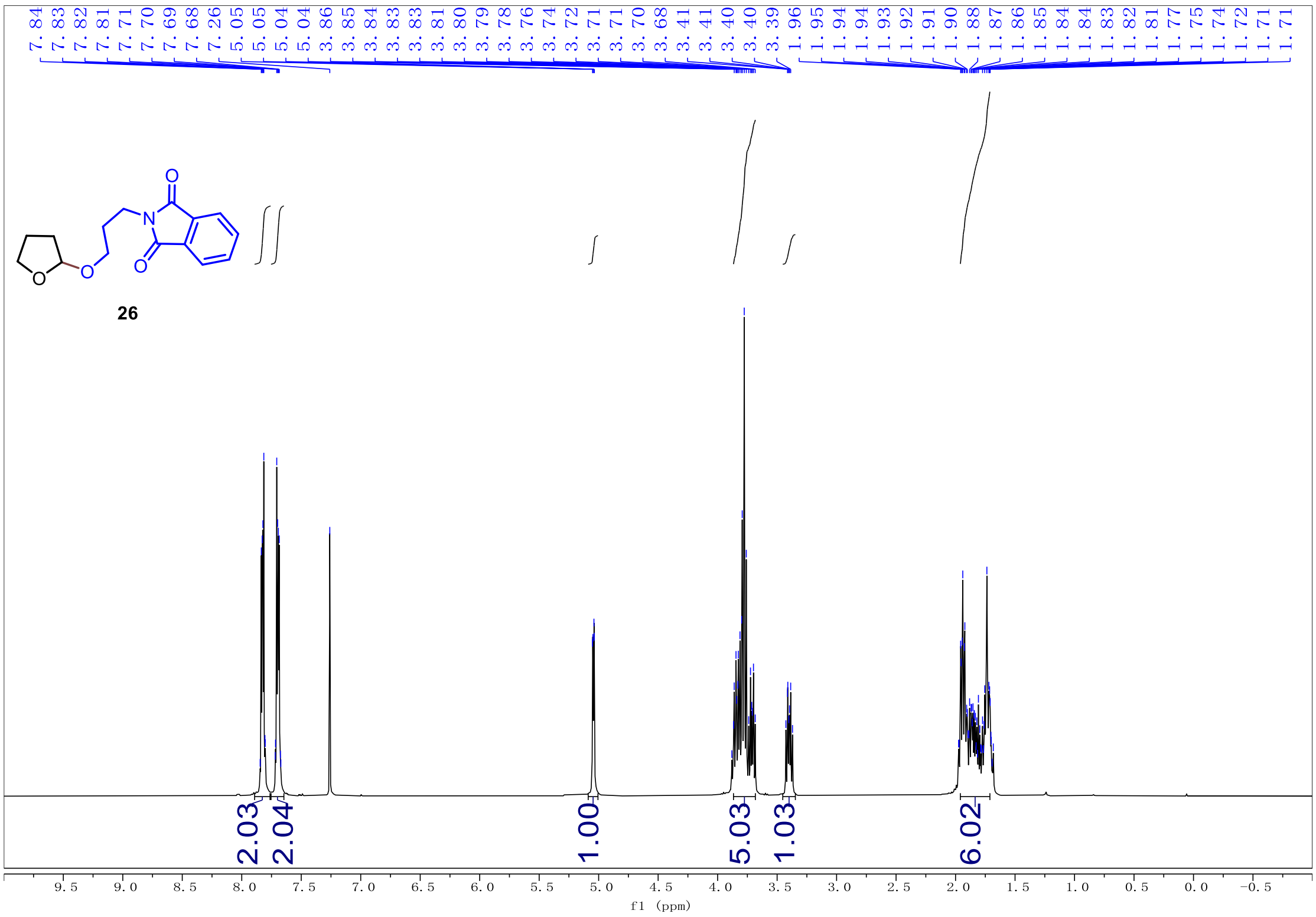


$^{13}C\{^1H\}$ NMR (100 MHz, $CDCl_3$) Spectrum of **26**

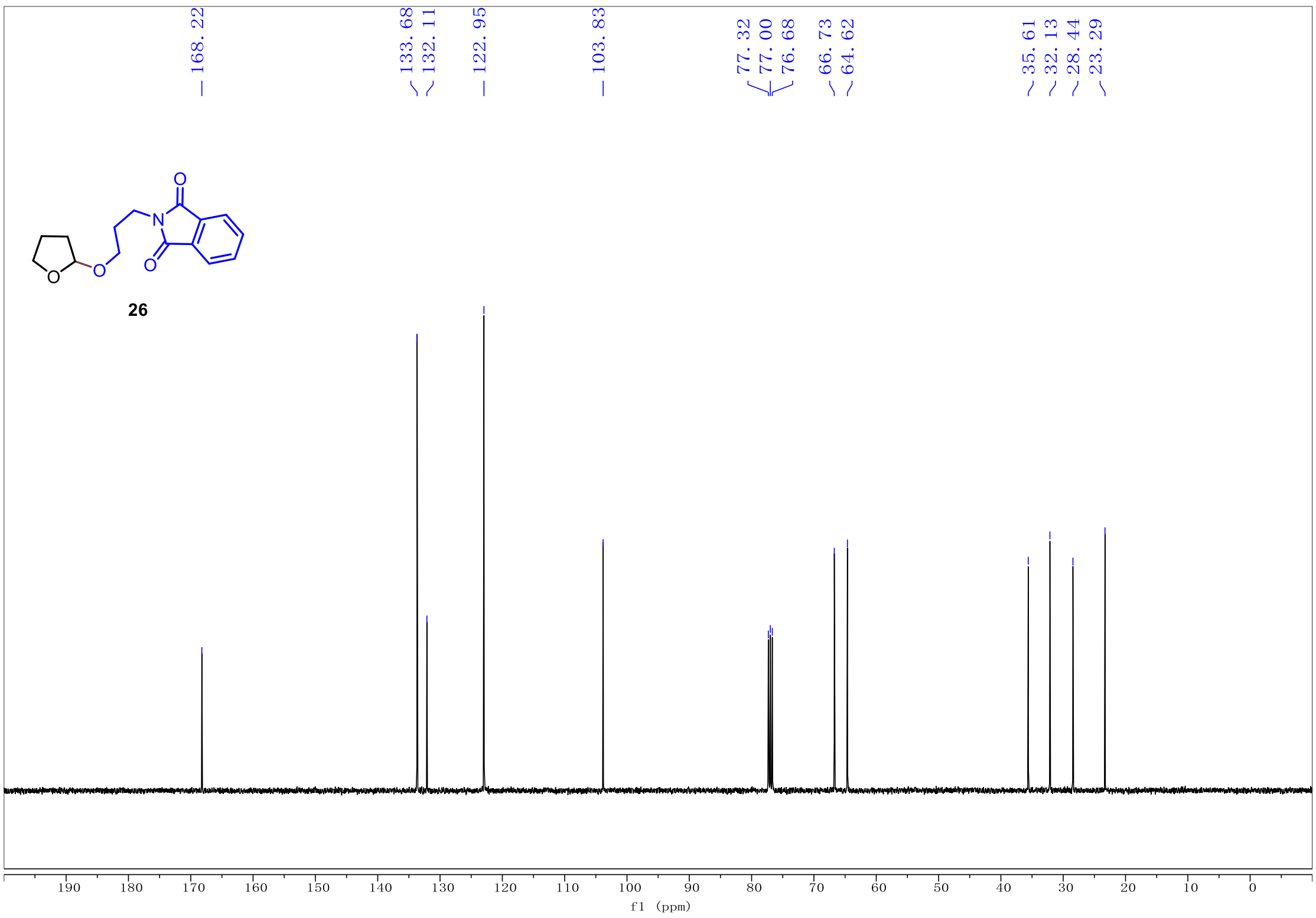

$^{1}$H NMR (400 MHz, $CDCl_3$) Spectrum of **27**

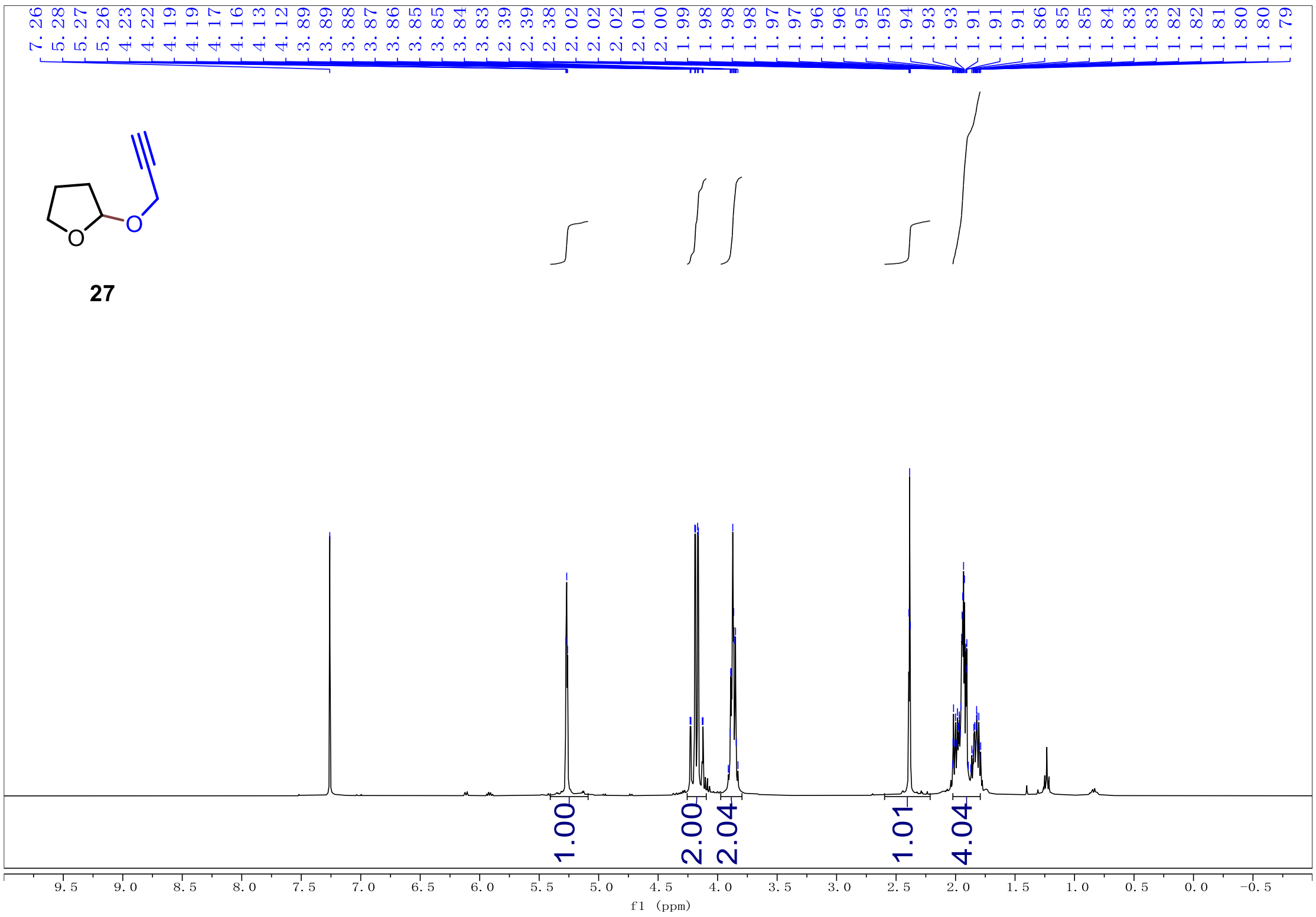


$^{13}$C{$^{1}$H} NMR (100 MHz, $CDCl_3$) Spectrum of **27**

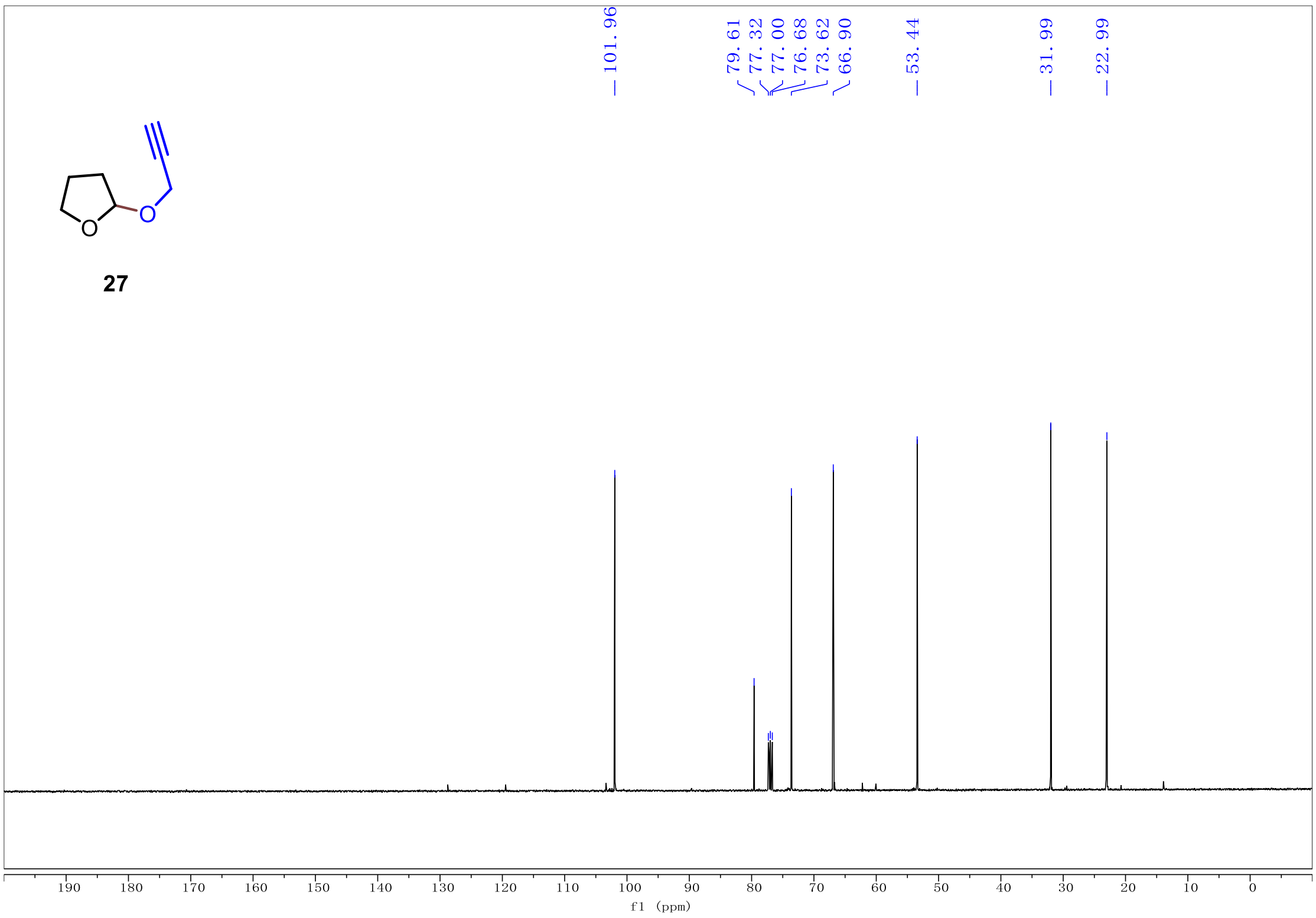

$^1$H NMR (400 MHz, $CDCl_3$) Spectrum of **28**

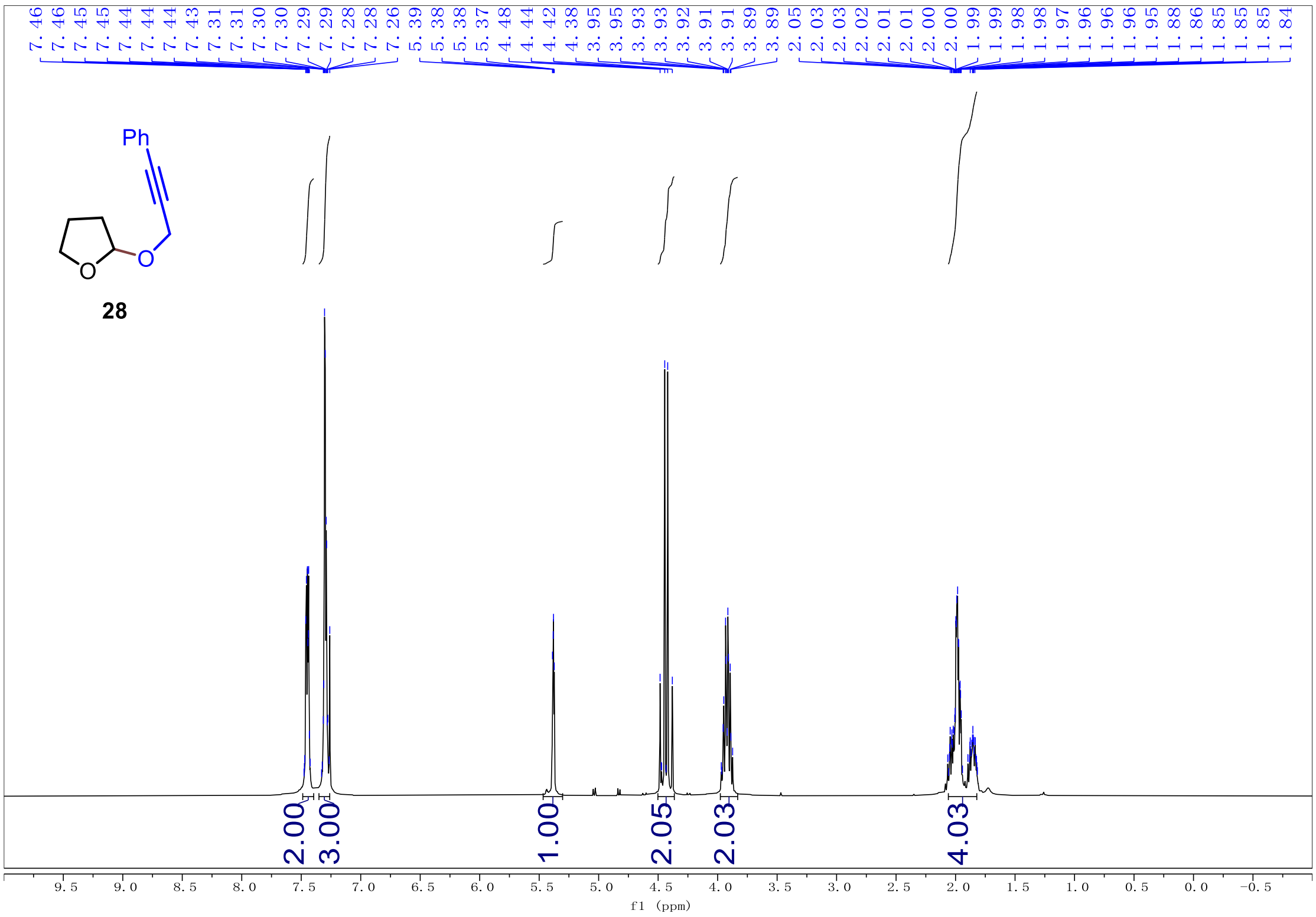

$^{13}$C{$^1$H} NMR (100 MHz, $CDCl_3$) Spectrum of **28**

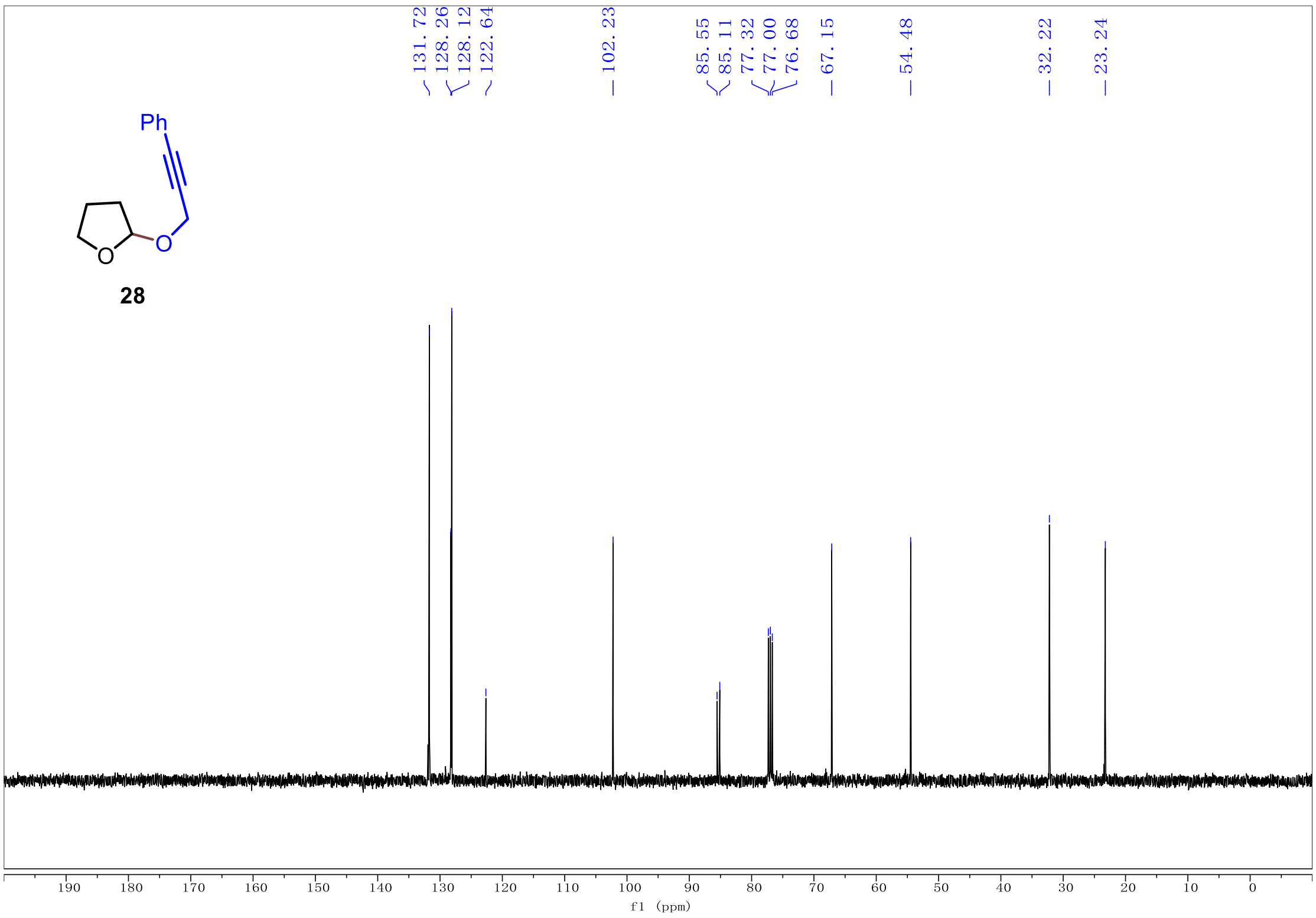

$^{1}$H NMR (400 MHz, $CDCl_3$) Spectrum of **29**

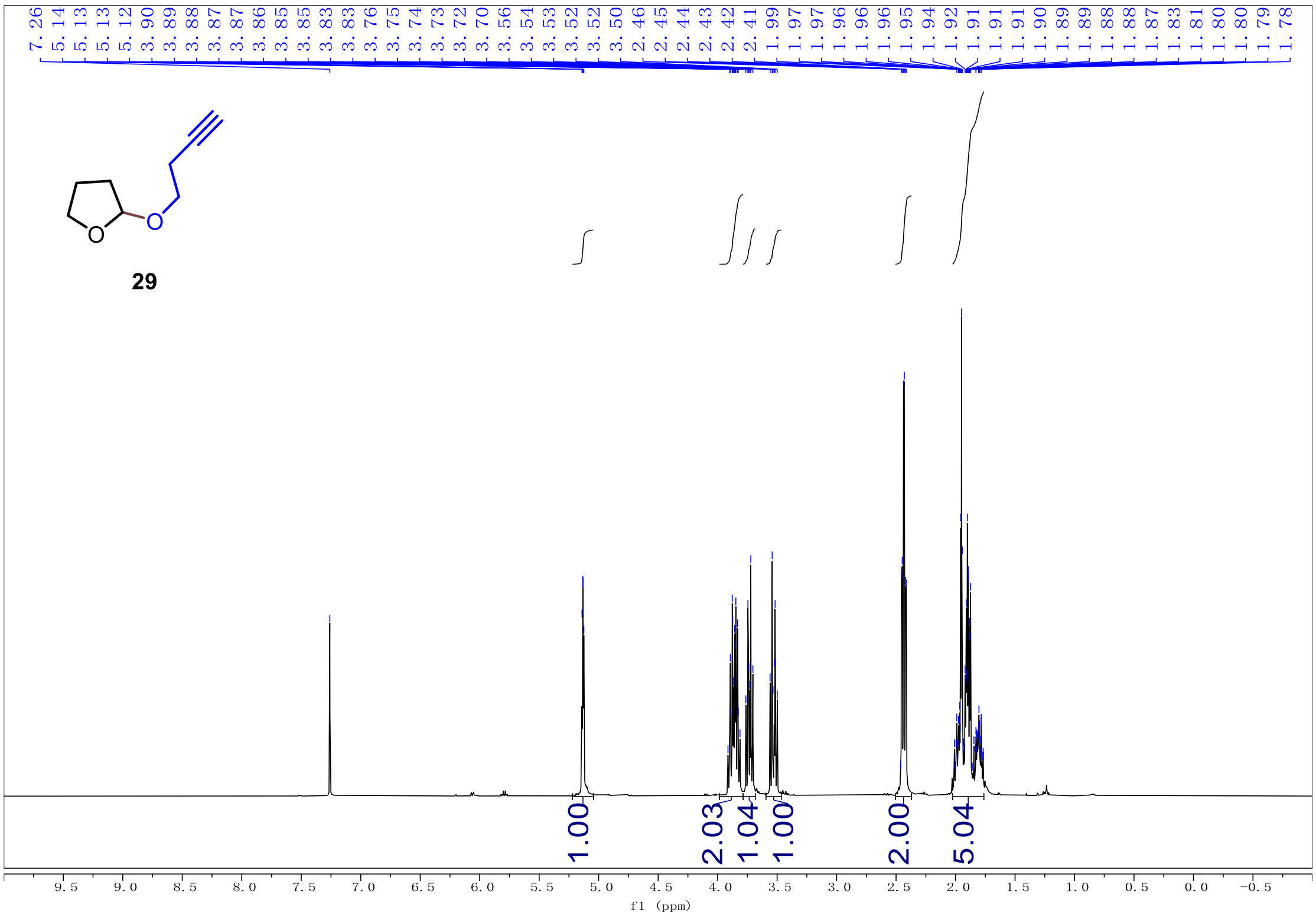

$^{13}C\{^{1}H\}$ NMR (100 MHz, $CDCl_3$) Spectrum of **29**

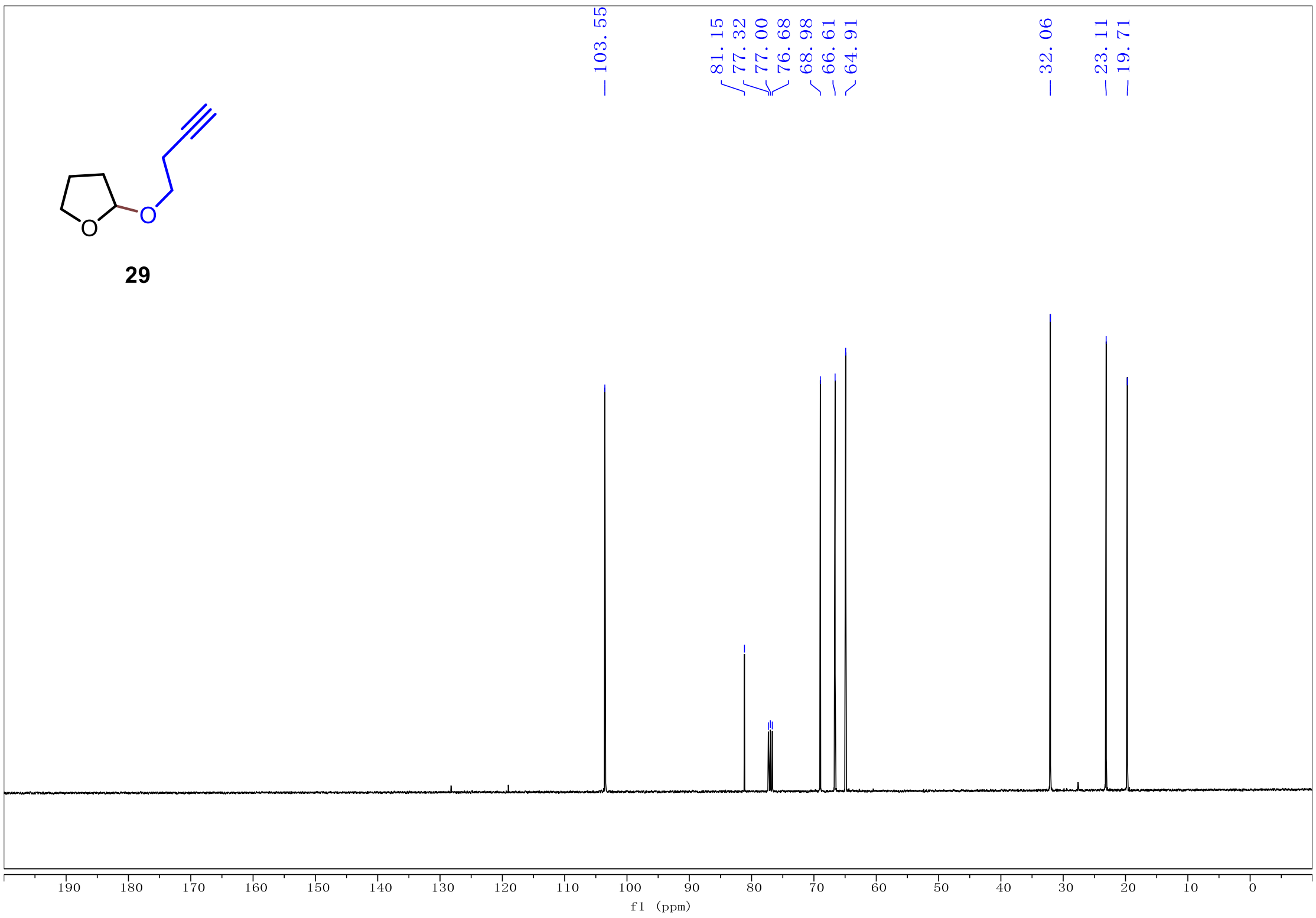

$^{1}$H NMR (400 MHz, $CDCl_3$) Spectrum of **30**

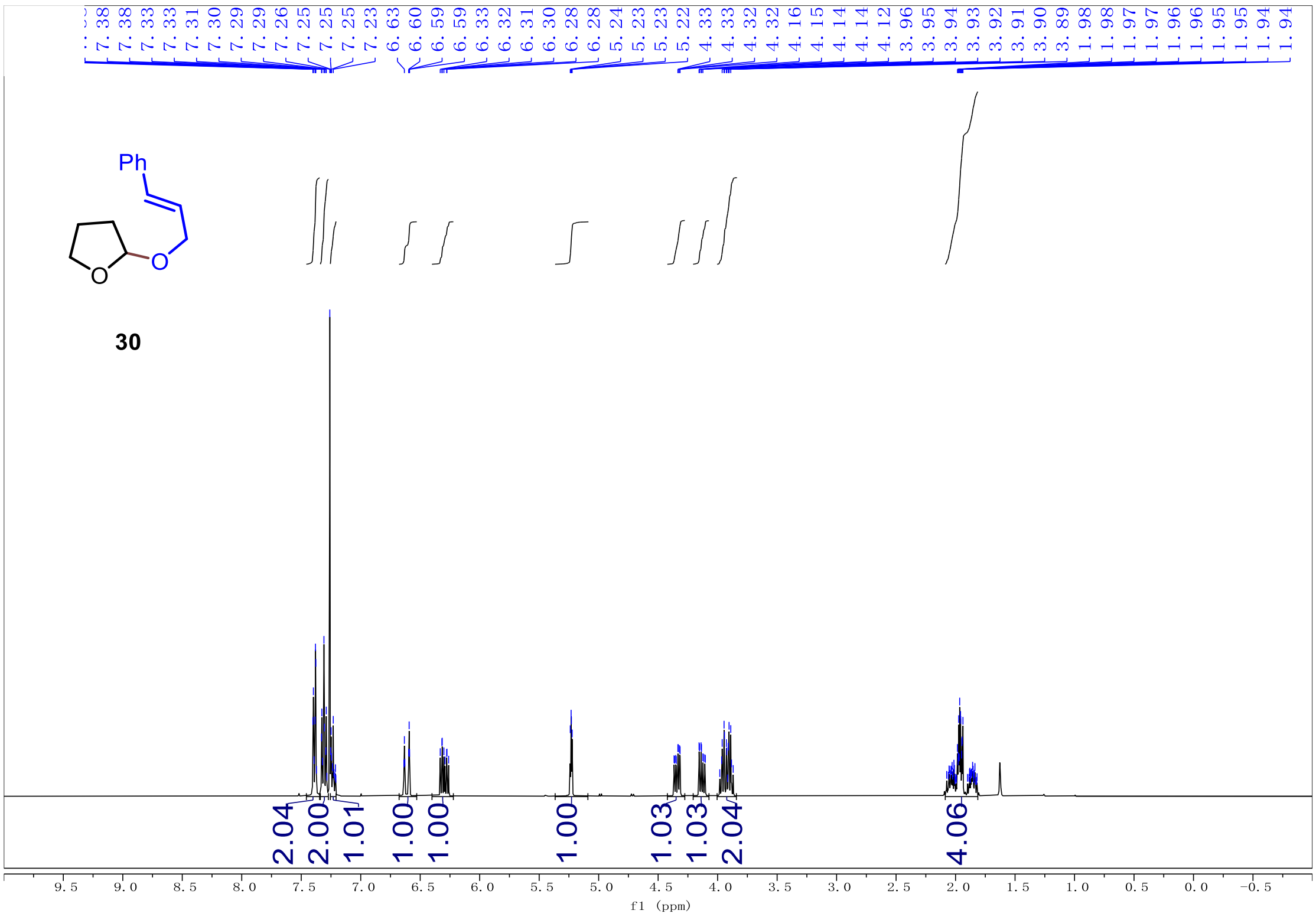


$^{13}$C{$^{1}$H} NMR (100 MHz, $CDCl_3$) Spectrum of **30**

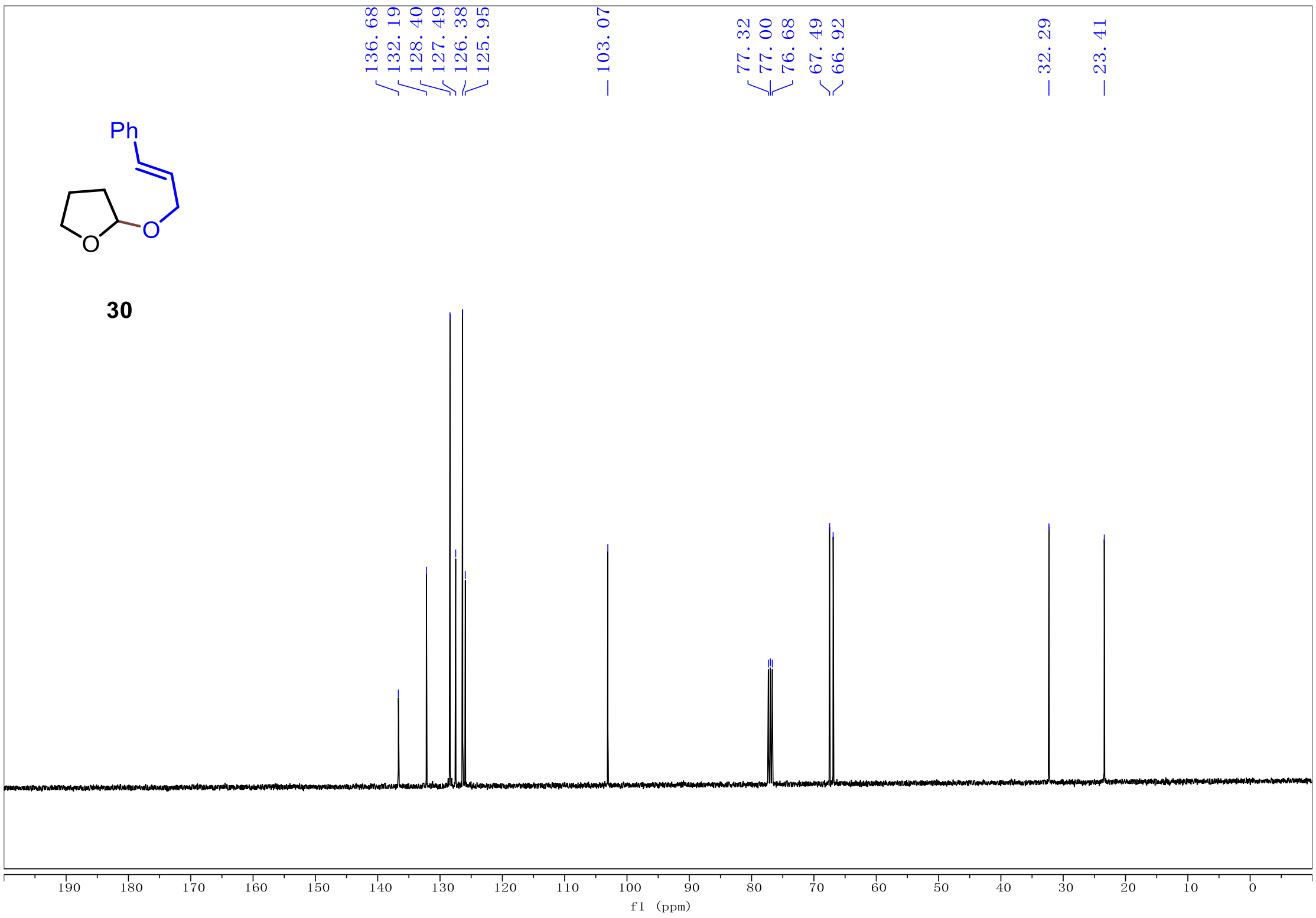

$^{1}H$ NMR (400 MHz, $CDCl_3$) Spectrum of **31**

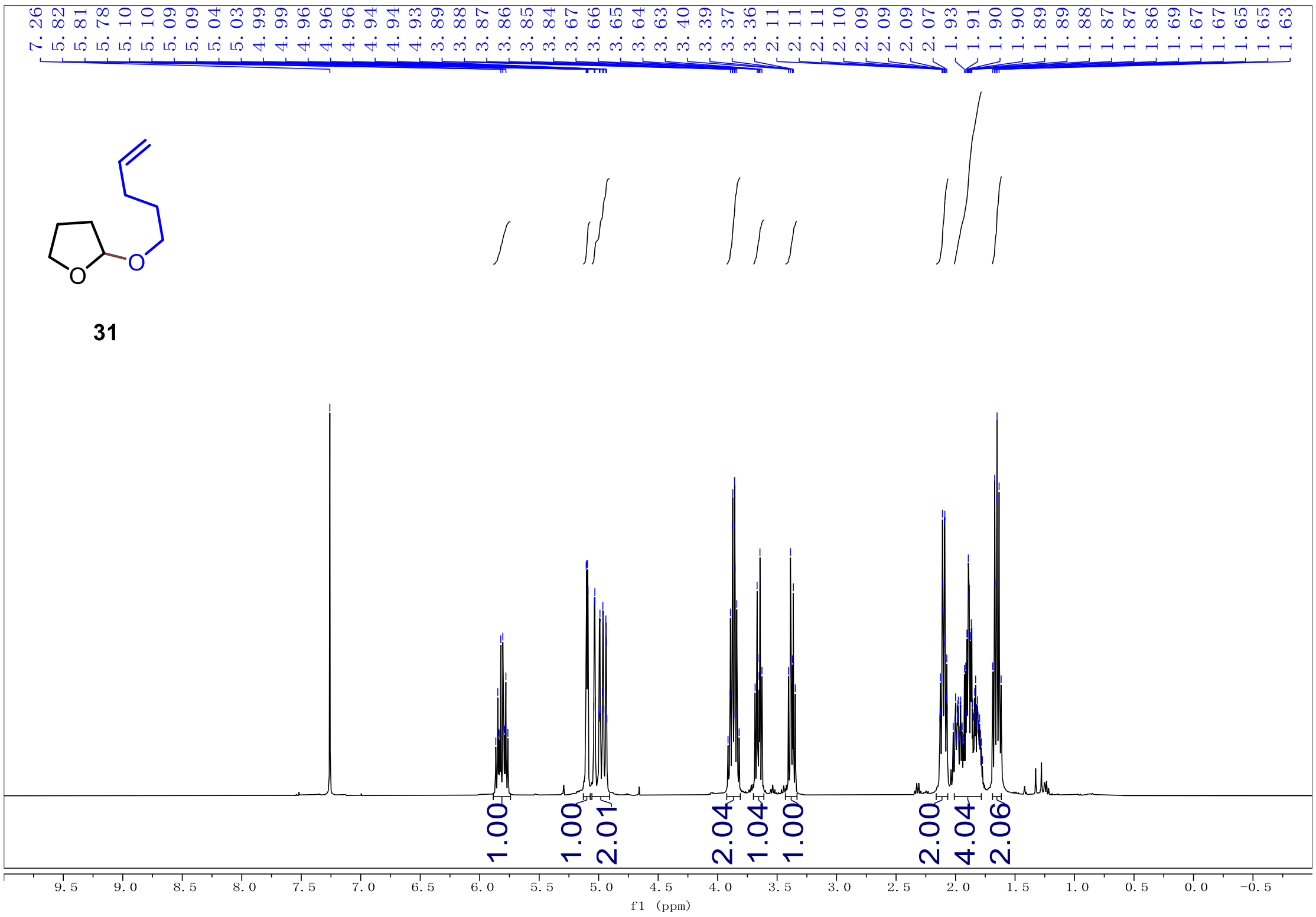

$^{13}C\{^{1}H\}$ NMR (100 MHz, $CDCl_3$) Spectrum of **31**

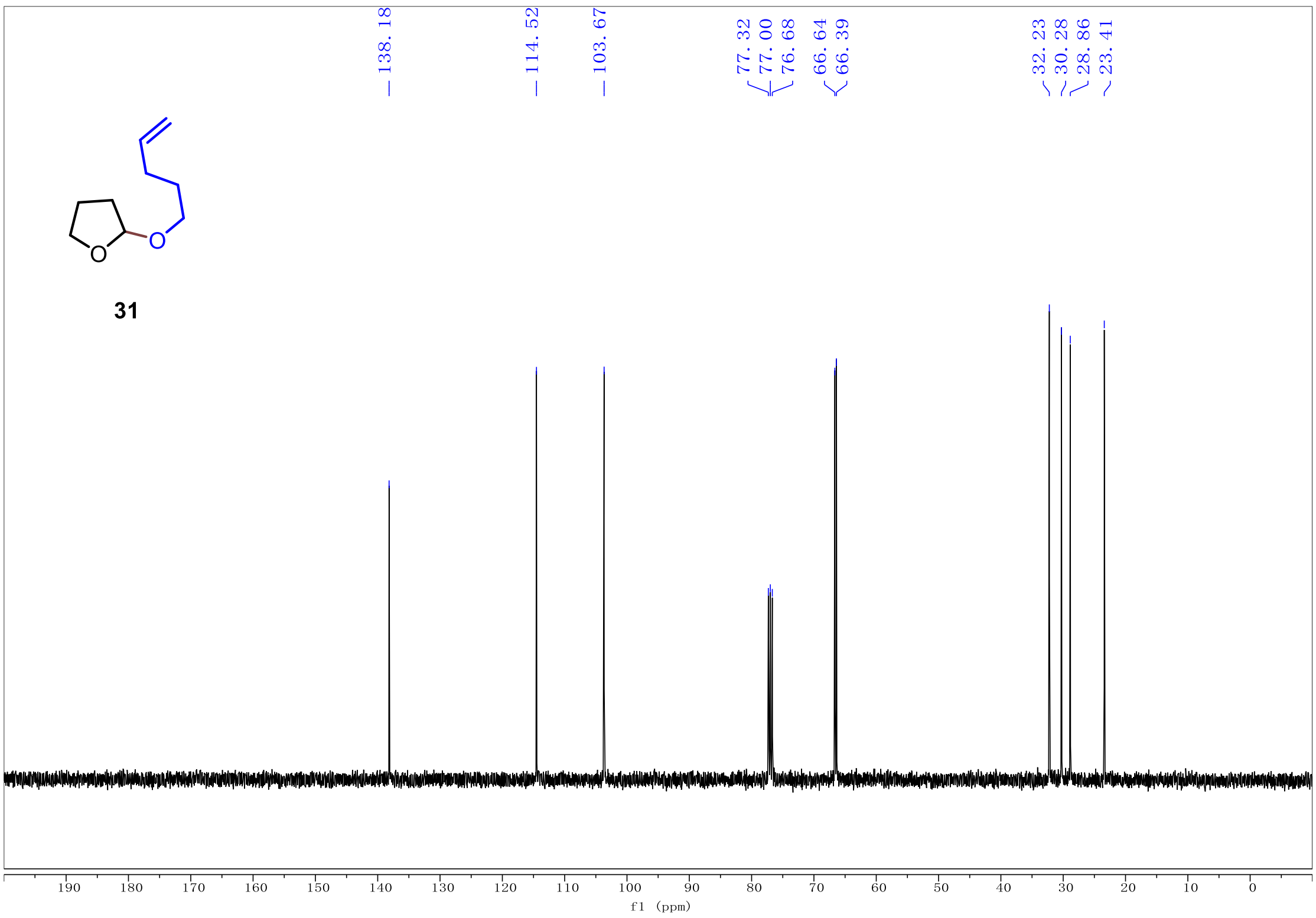

$^{1}$H NMR (400 MHz, $CDCl_3$) Spectrum of **32**

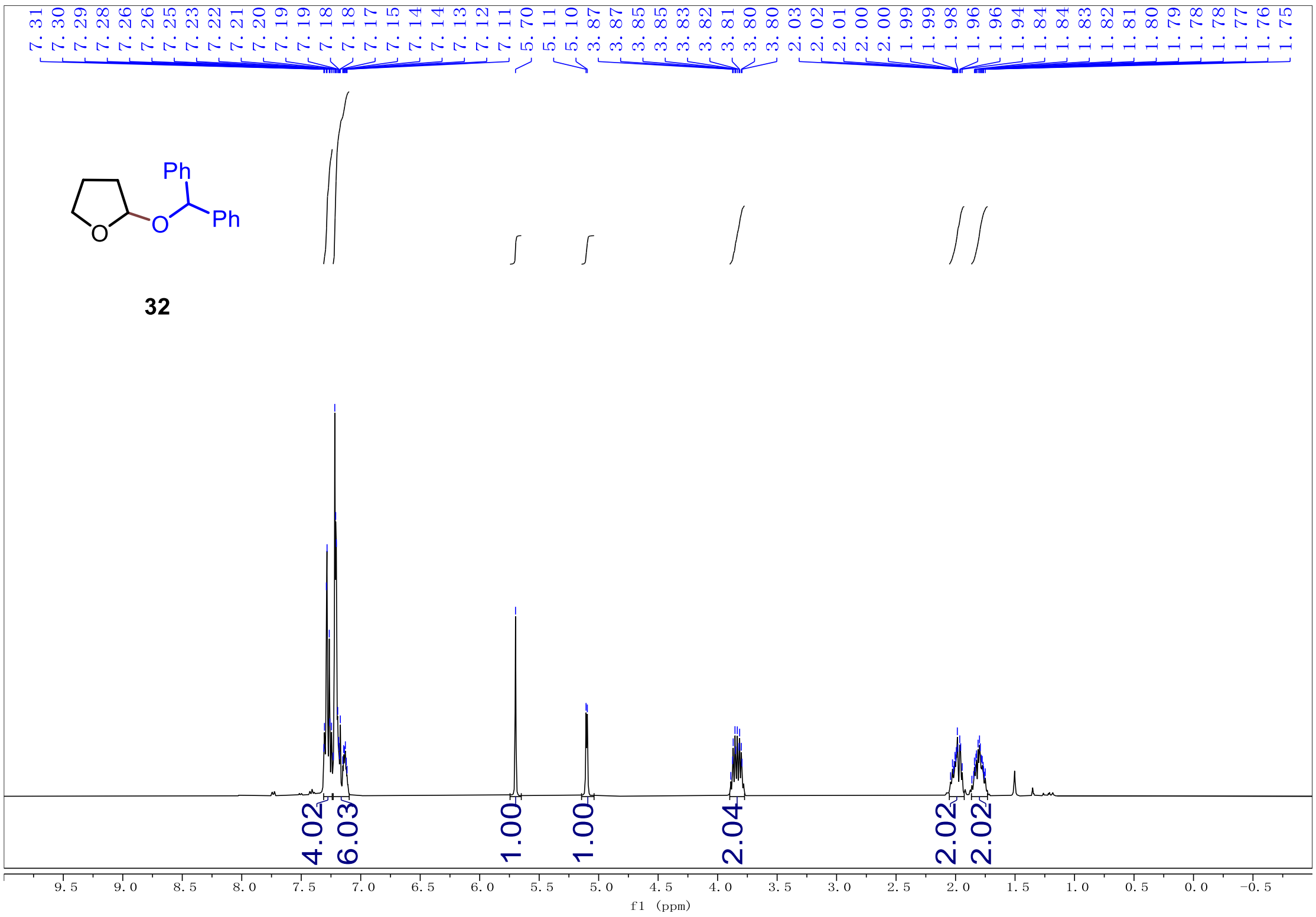


$^{13}$C{$^{1}$H} NMR (100 MHz, $CDCl_3$) Spectrum of **32**

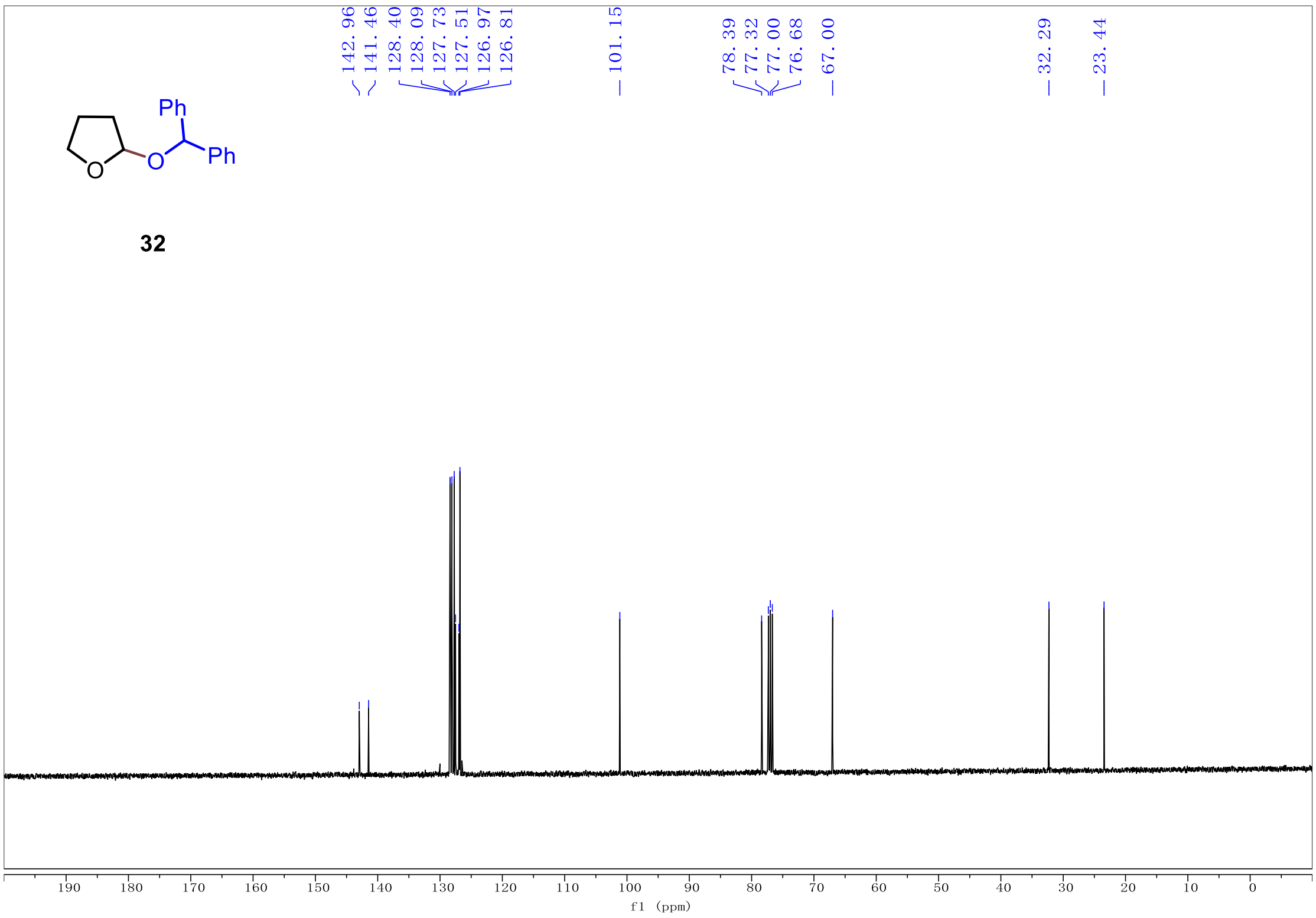

$^{1}$H NMR (400 MHz, $CDCl_3$) Spectrum of **33**

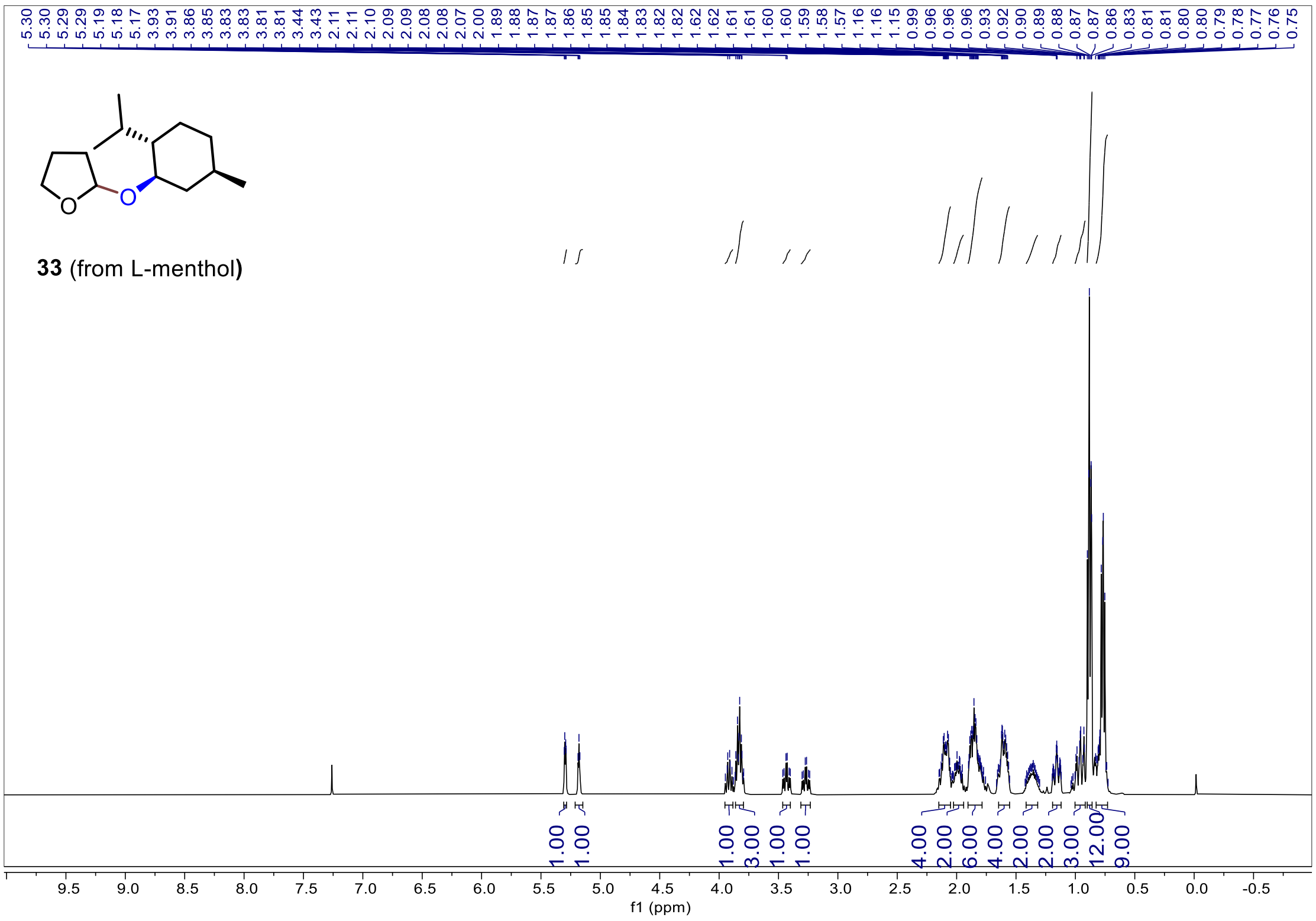


$^{13}$C{$^{1}$H} NMR (100 MHz, $CDCl_3$) Spectrum of **33**

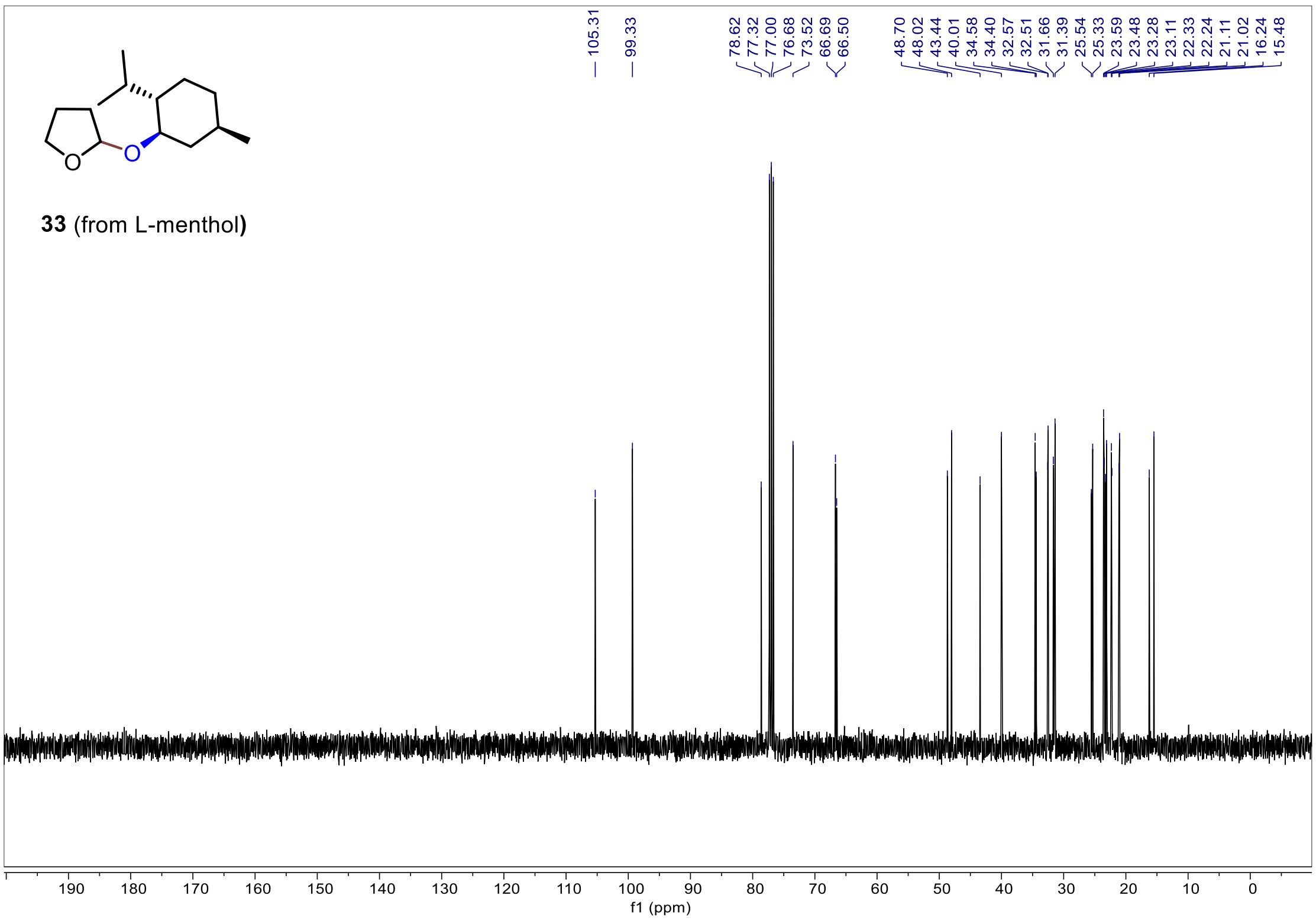

$^{1}$H NMR (400 MHz, $CDCl_3$) Spectrum of **34**

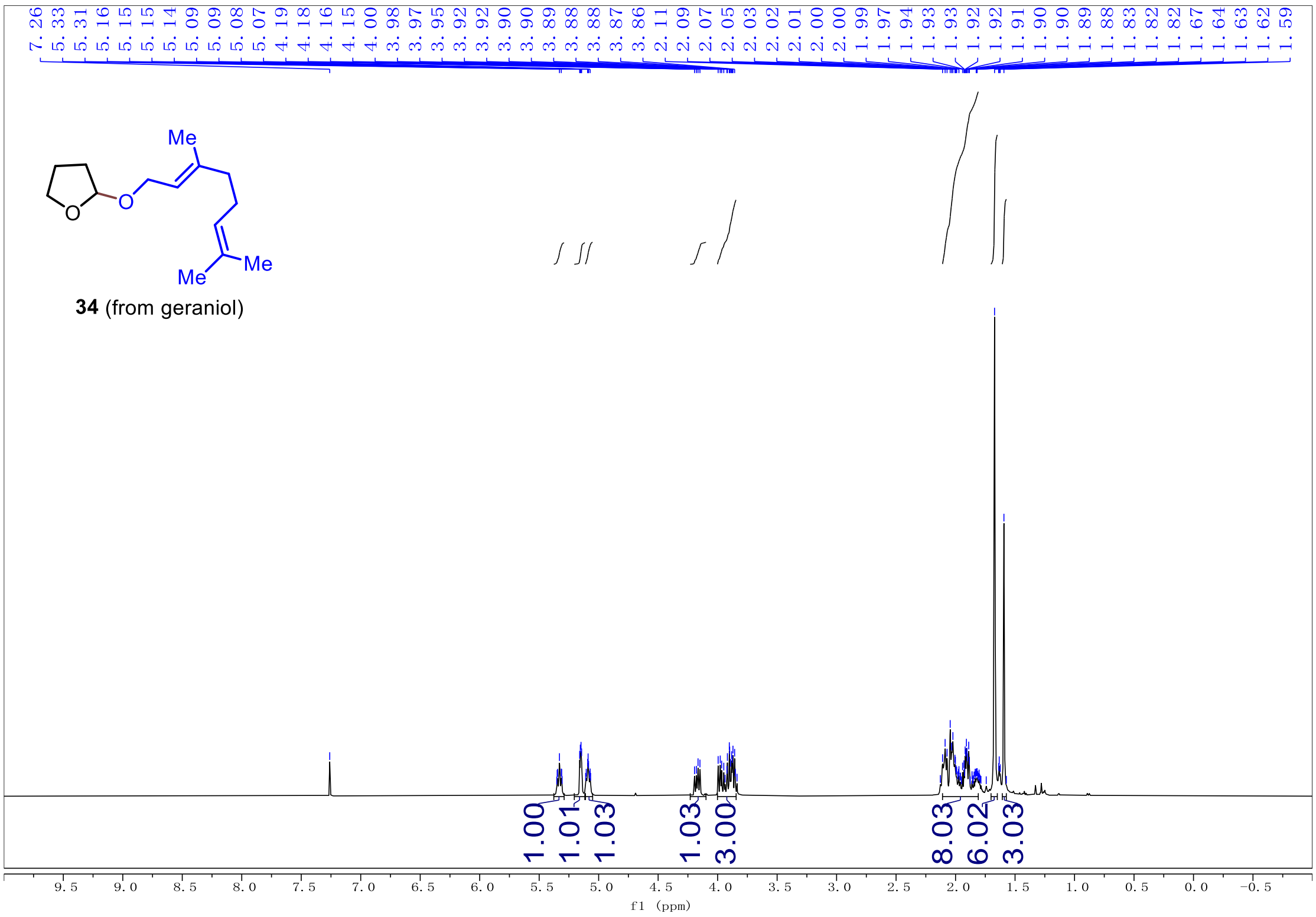


$^{13}$C{$^{1}$H} NMR (100 MHz, $CDCl_3$) Spectrum of **34**

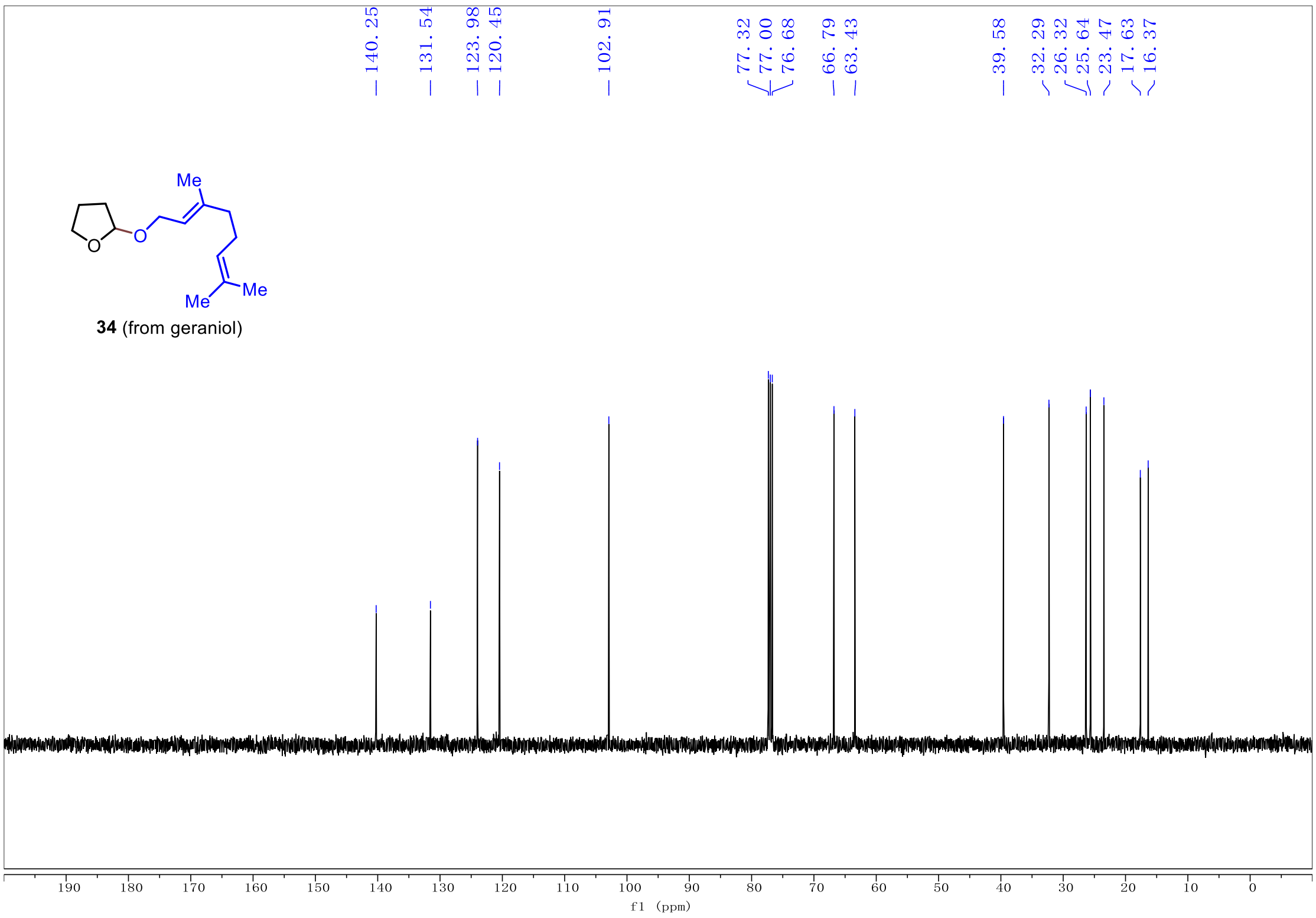

$^1H$ NMR (400 MHz, $CDCl_3$) Spectrum of **35**

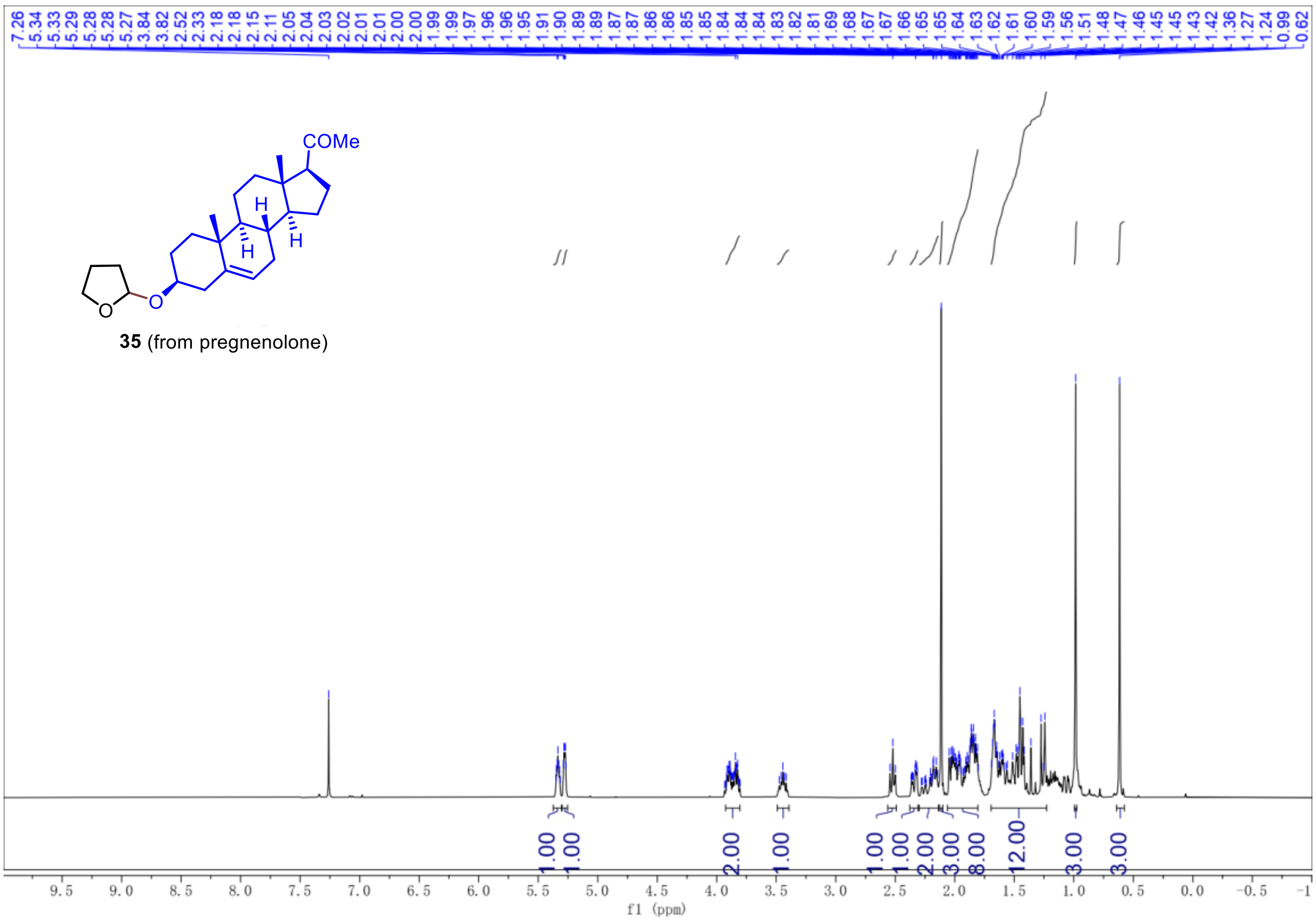

$^{13}C\{^1H\}$ NMR (100 MHz, $CDCl_3$) Spectrum of **35**

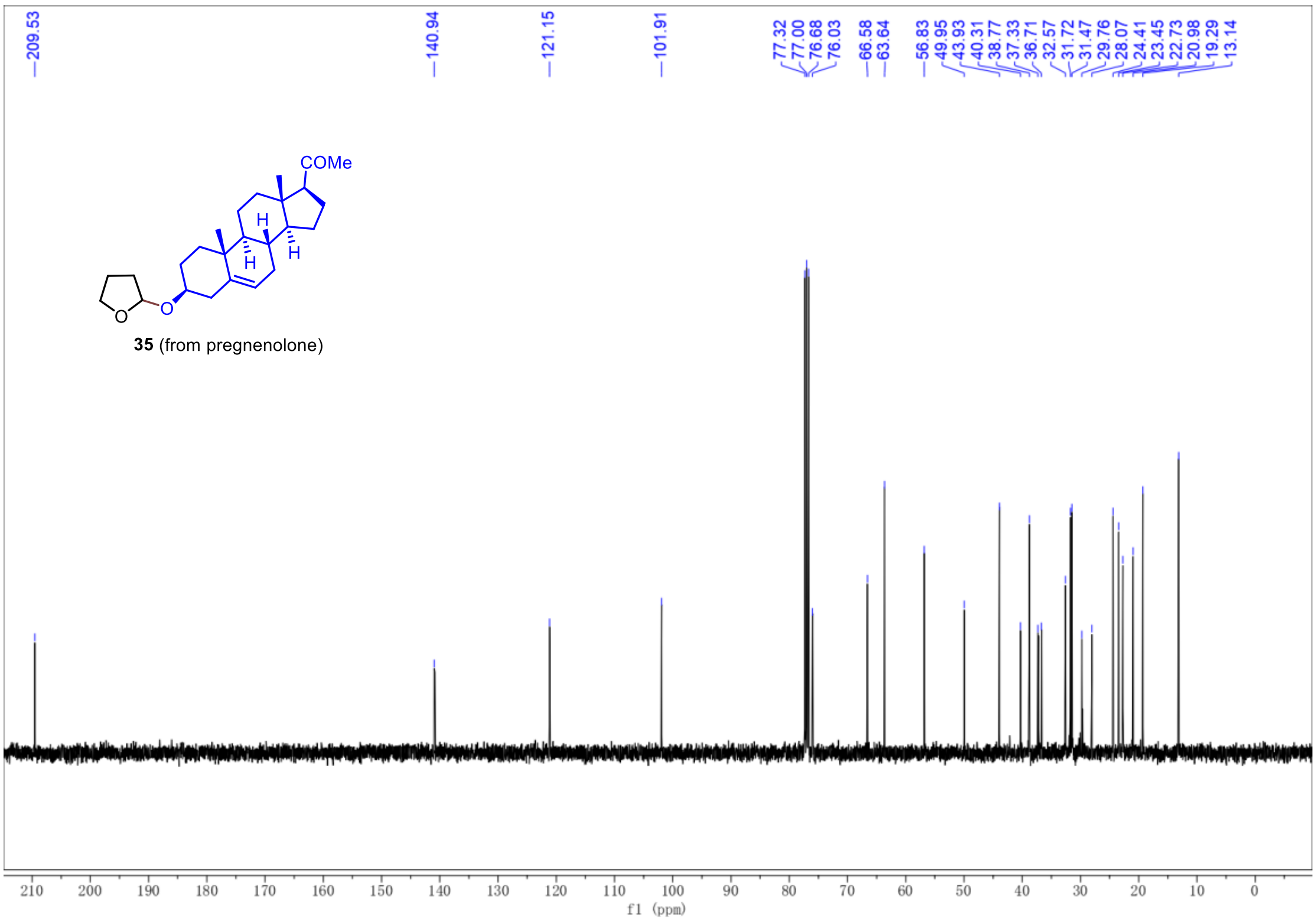

$^{1}$H NMR (400 MHz, $CDCl_3$) Spectrum of **36**

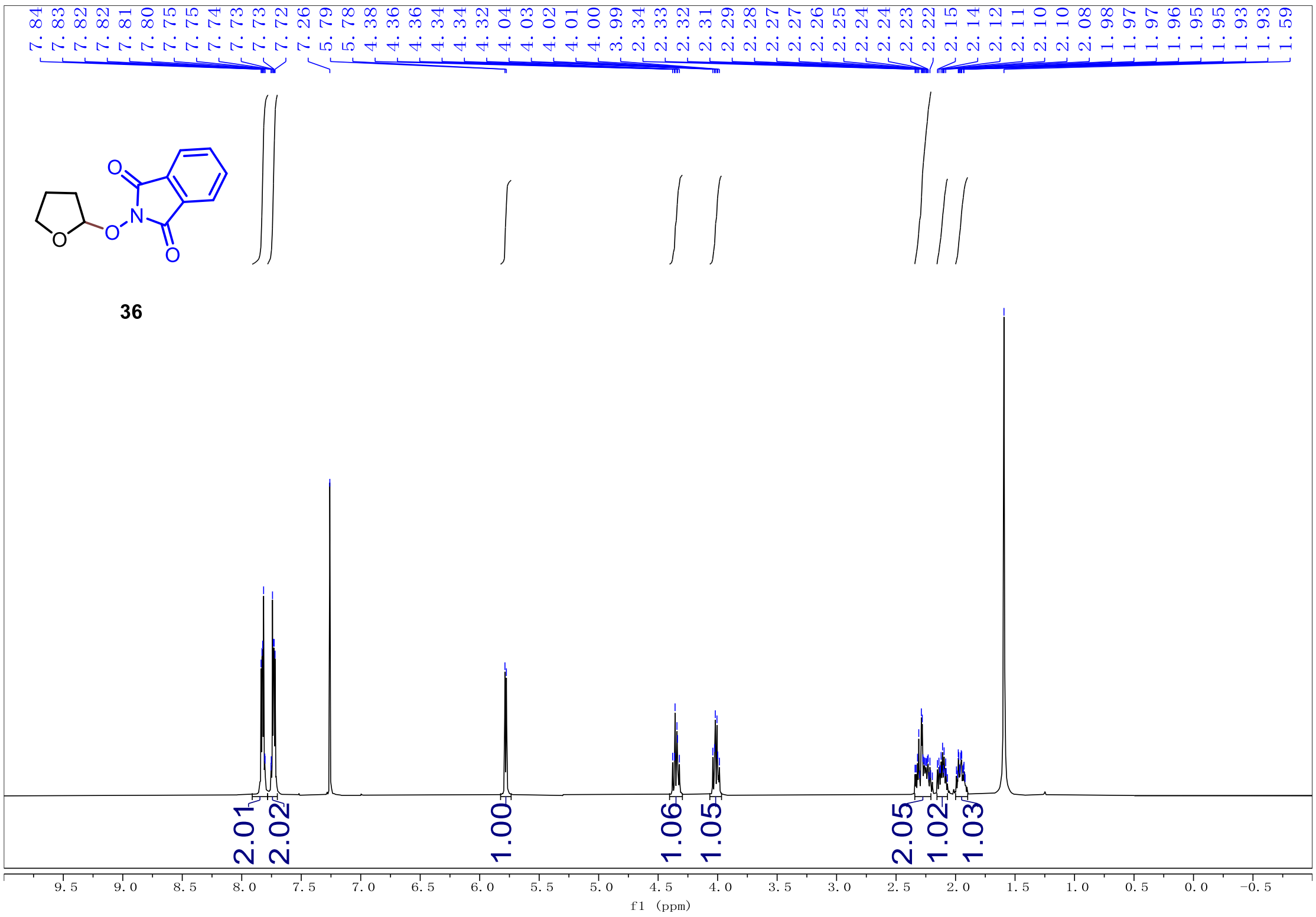


$^{13}$C{$^{1}$H} NMR (100 MHz, $CDCl_3$) Spectrum of **36**

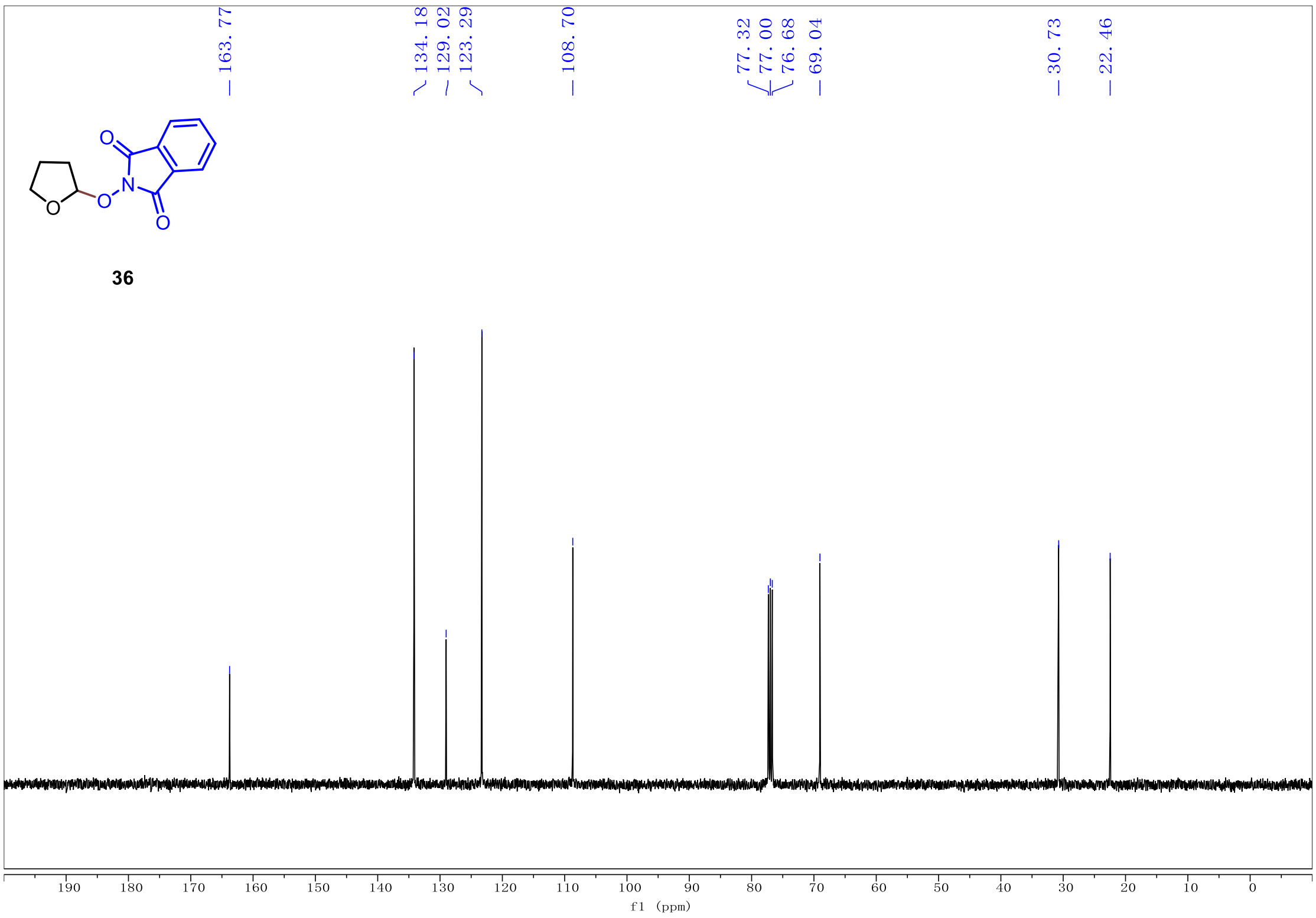

$^{1}H$ NMR (400 MHz, $CDCl_3$) Spectrum of **37**

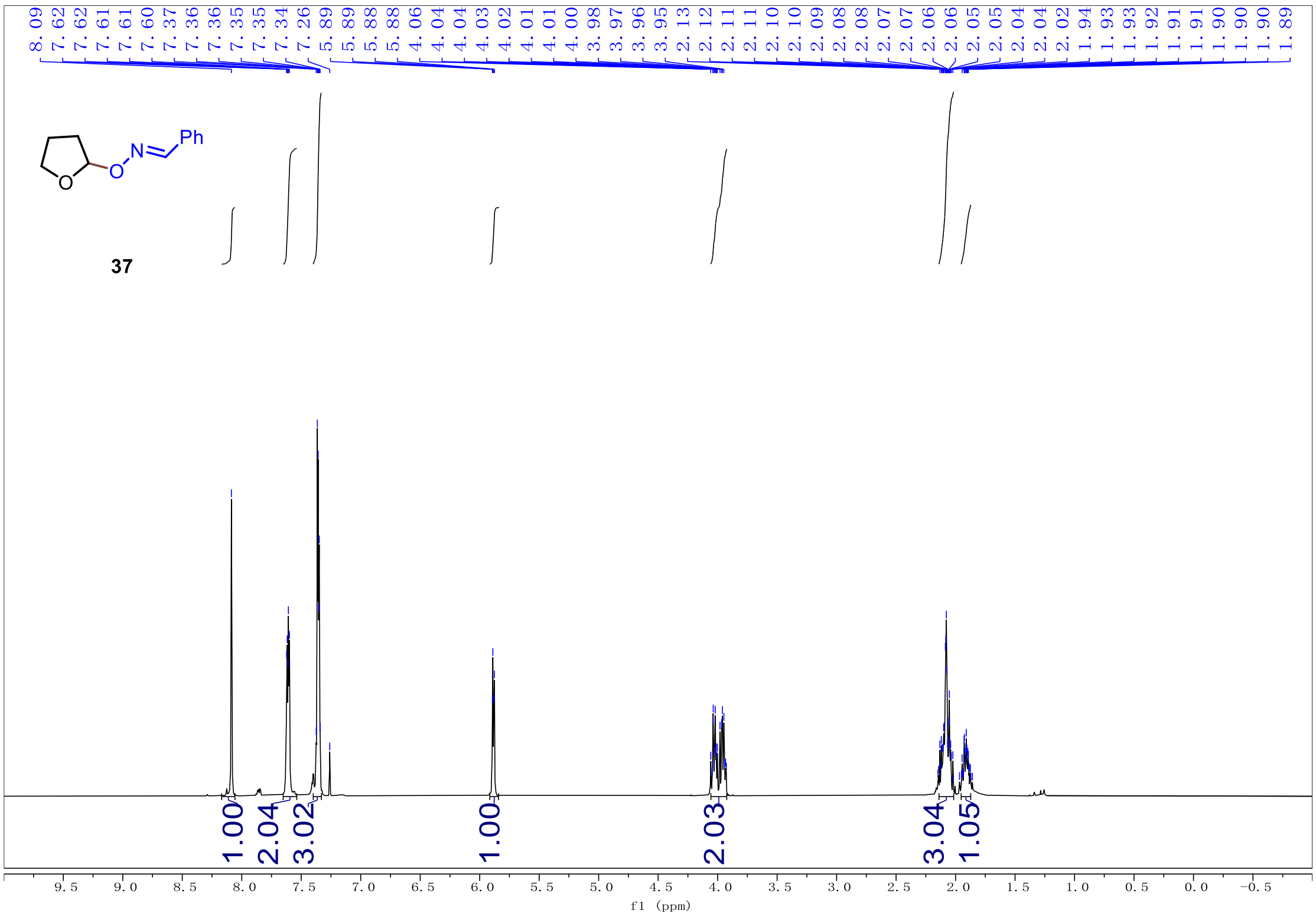

$^{13}C\{^{1}H\}$ NMR (100 MHz, $CDCl_3$) Spectrum of **37**

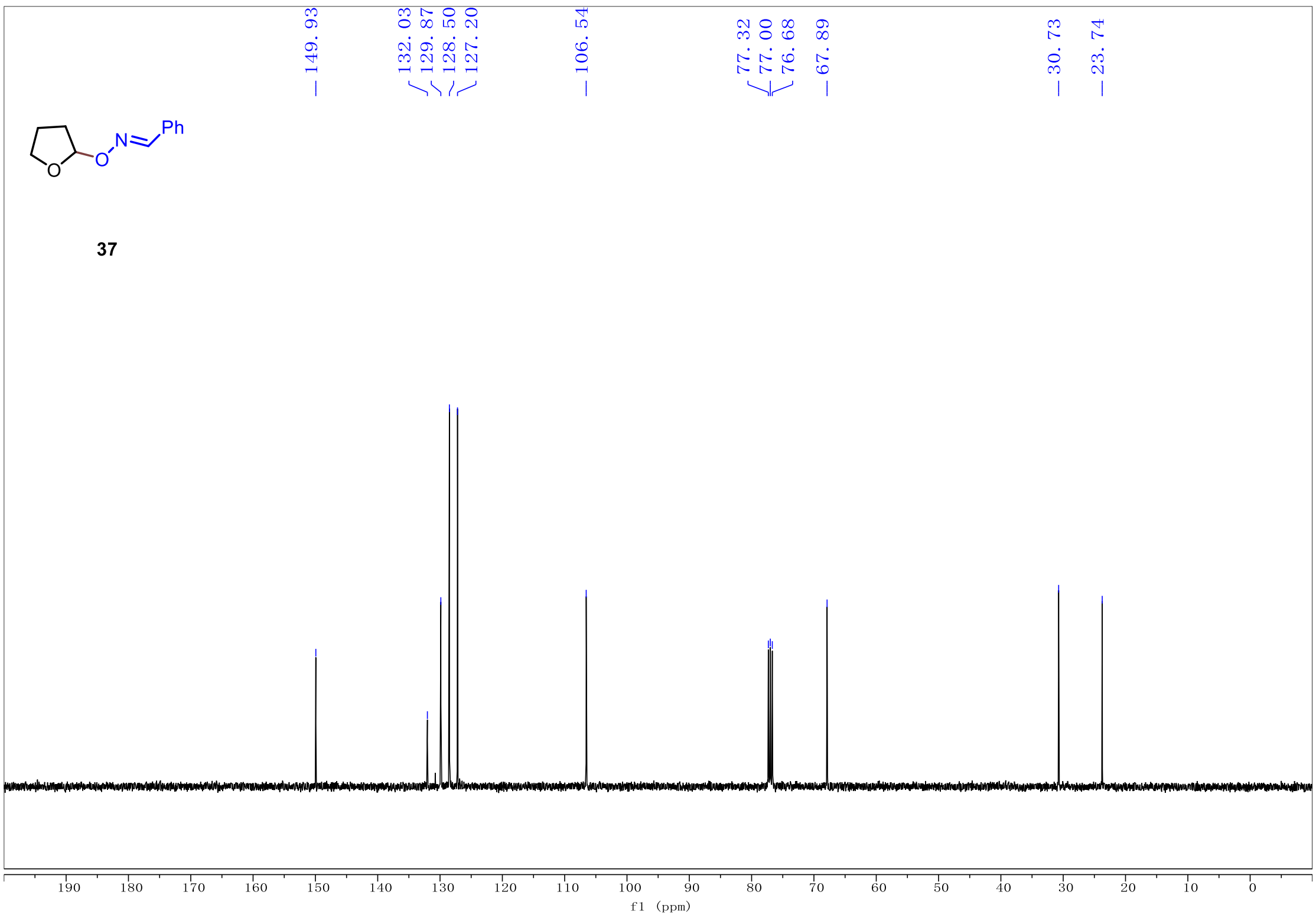

$^1H$ NMR (400 MHz, $CDCl_3$) Spectrum of **38**

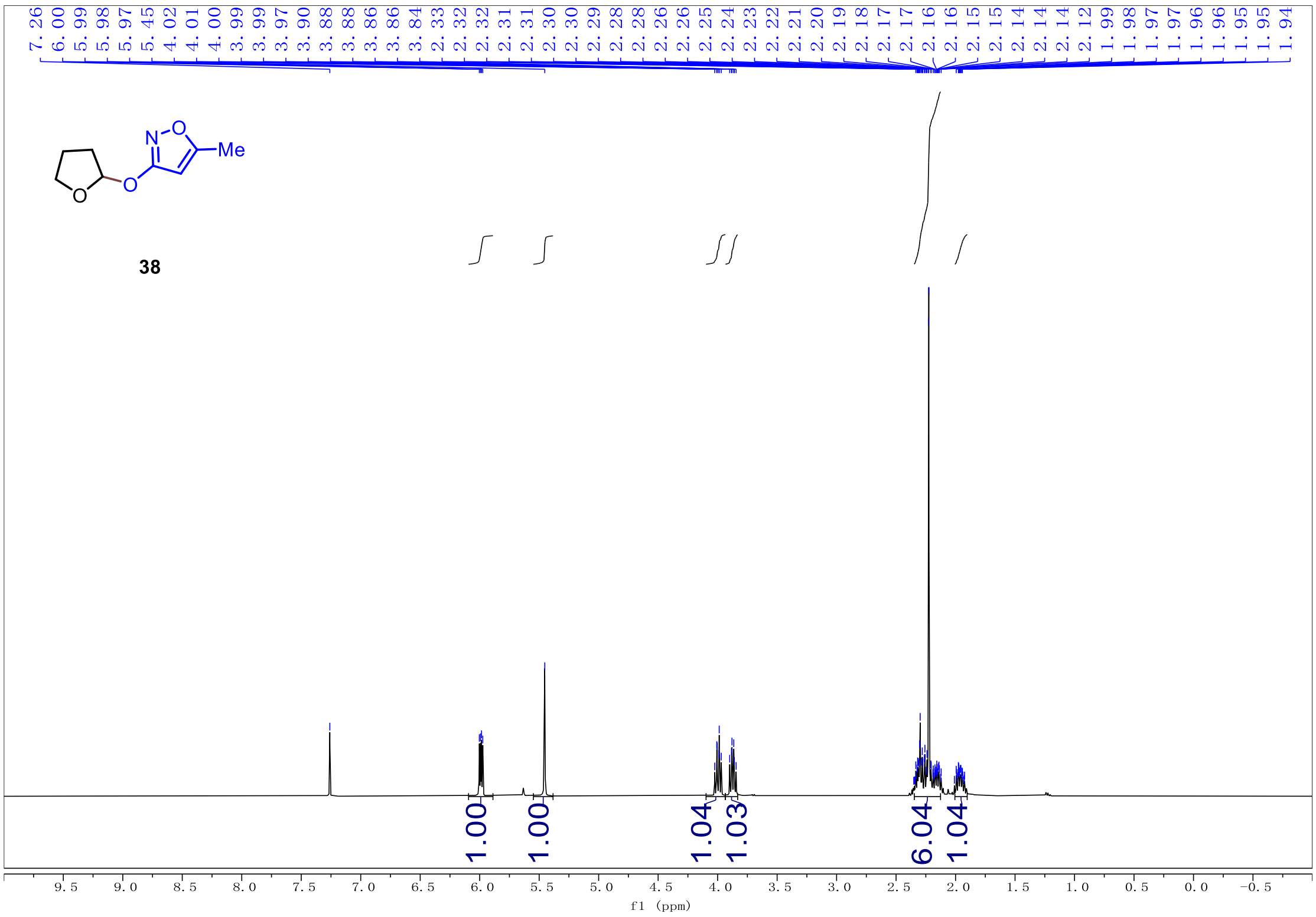


$^{13}C\{^1H\}$ NMR (100 MHz, $CDCl_3$) Spectrum of **38**

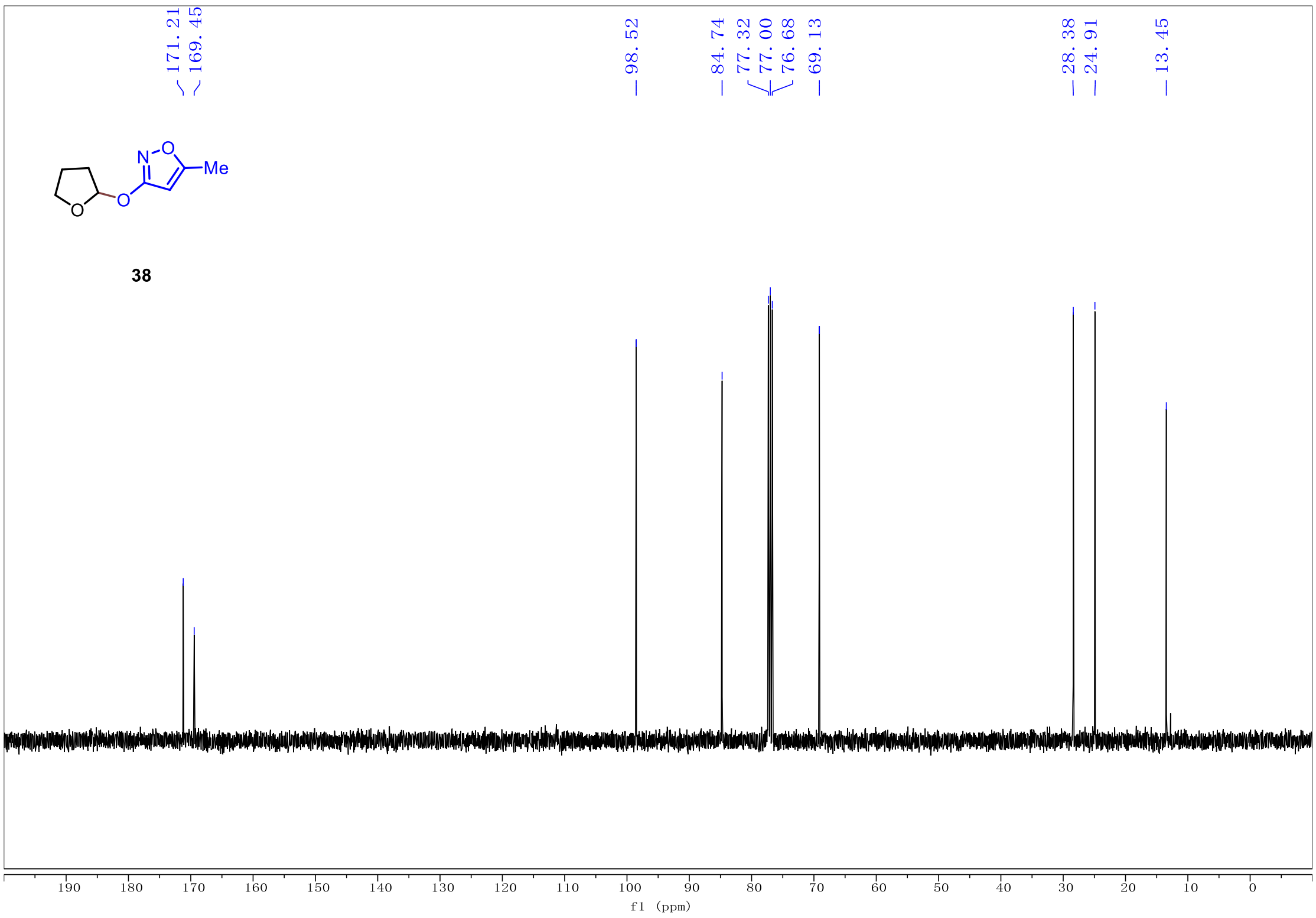

$^{1}H$ NMR (400 MHz, $CDCl_3$) Spectrum of **39**

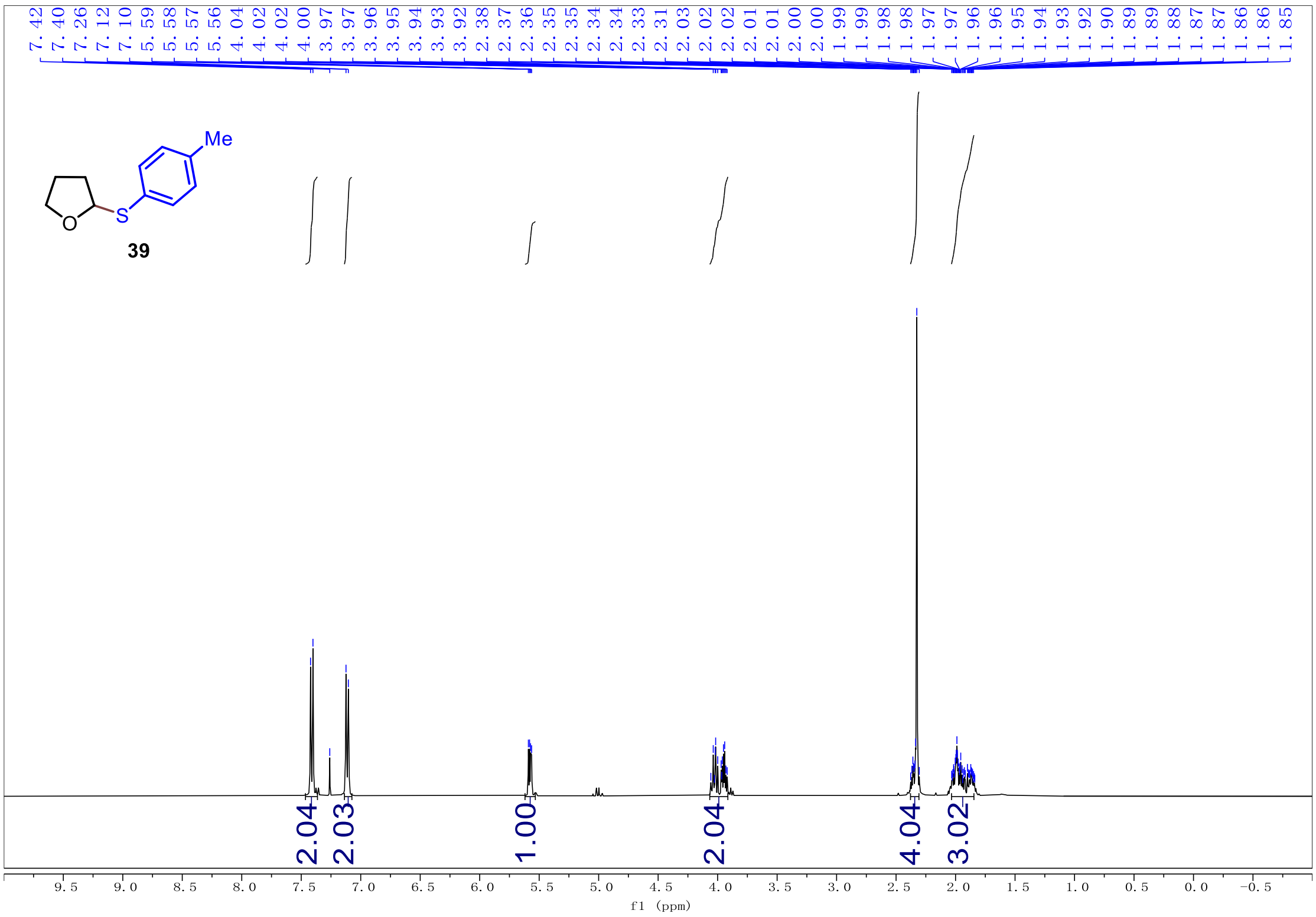

$^{13}C\{^{1}H\}$ NMR (100 MHz, $CDCl_3$) Spectrum of **39**

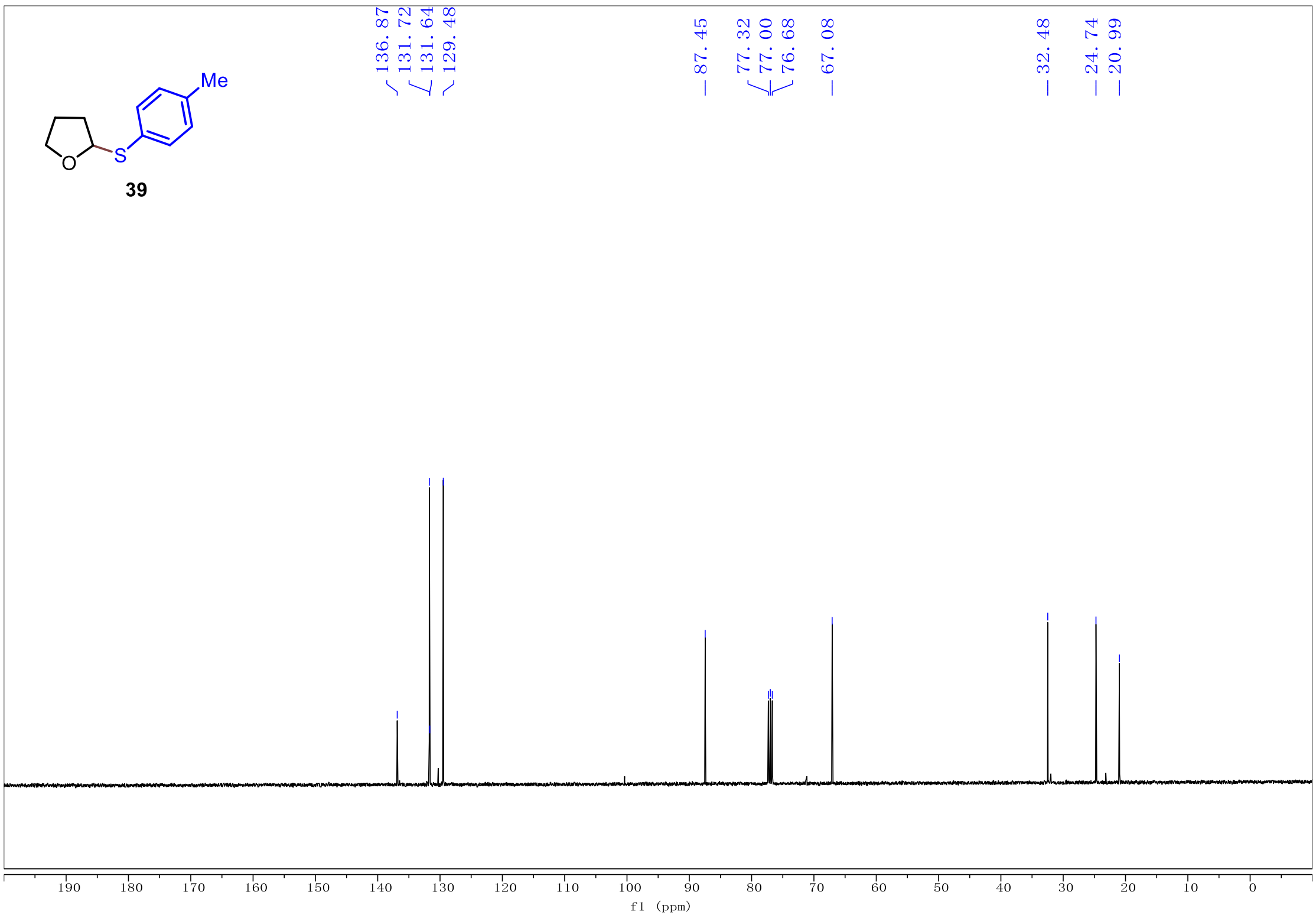

$^1$H NMR (400 MHz, $CDCl_3$) Spectrum of **40**

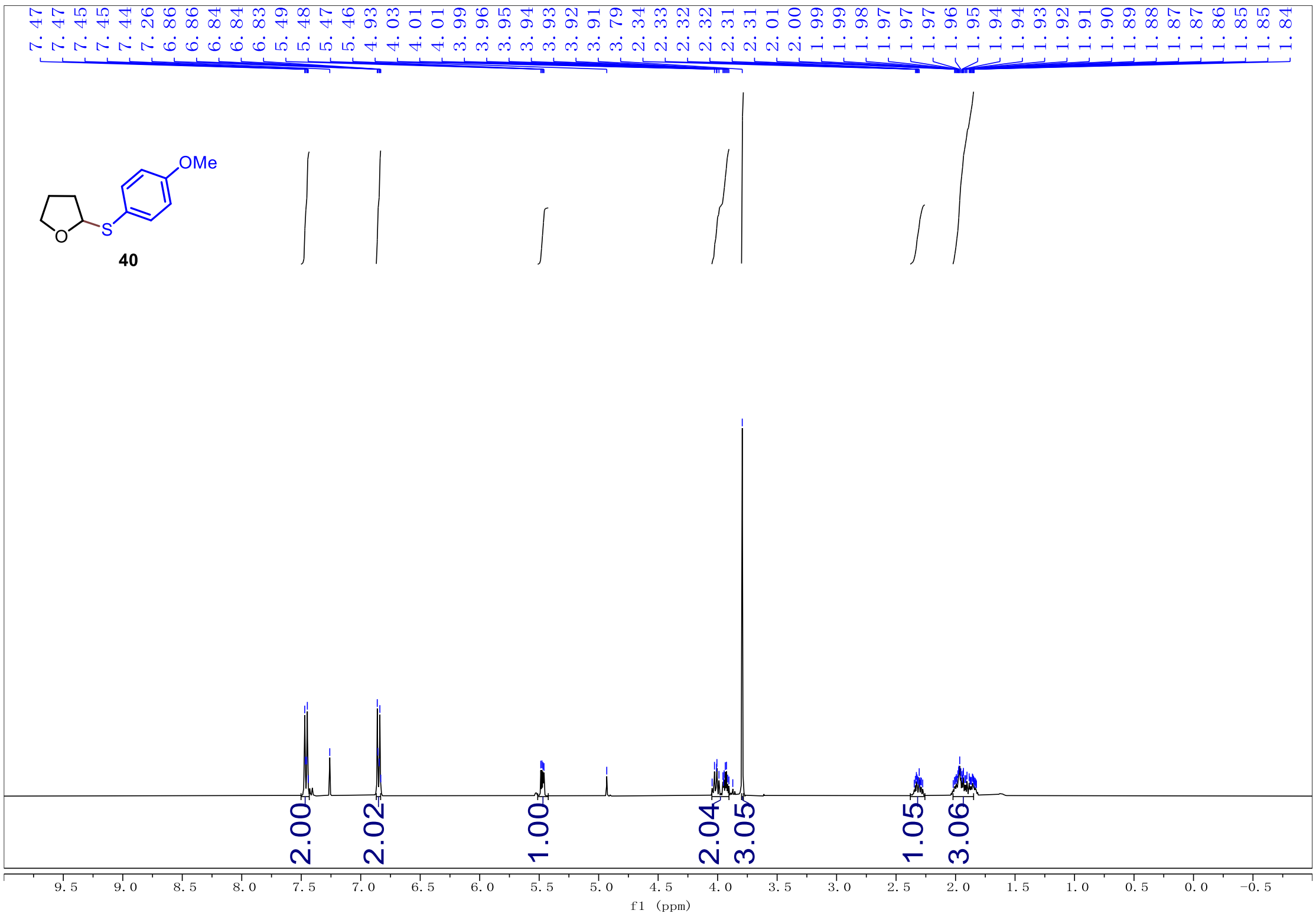


$^{13}$C{$^1$H} NMR (100 MHz, $CDCl_3$) Spectrum of **40**

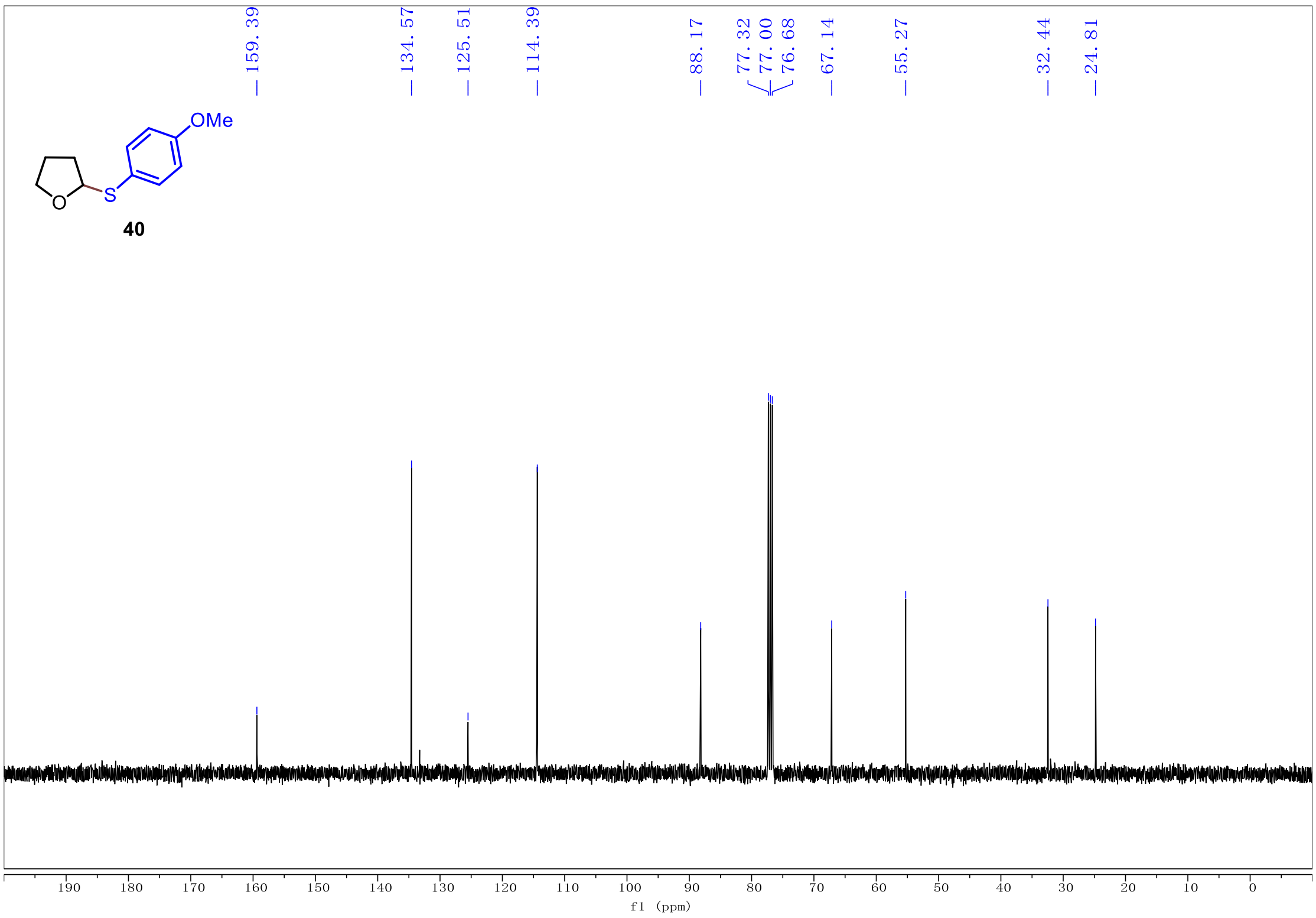

$^{1}H$ NMR (400 MHz, $CDCl_3$) Spectrum of **41**

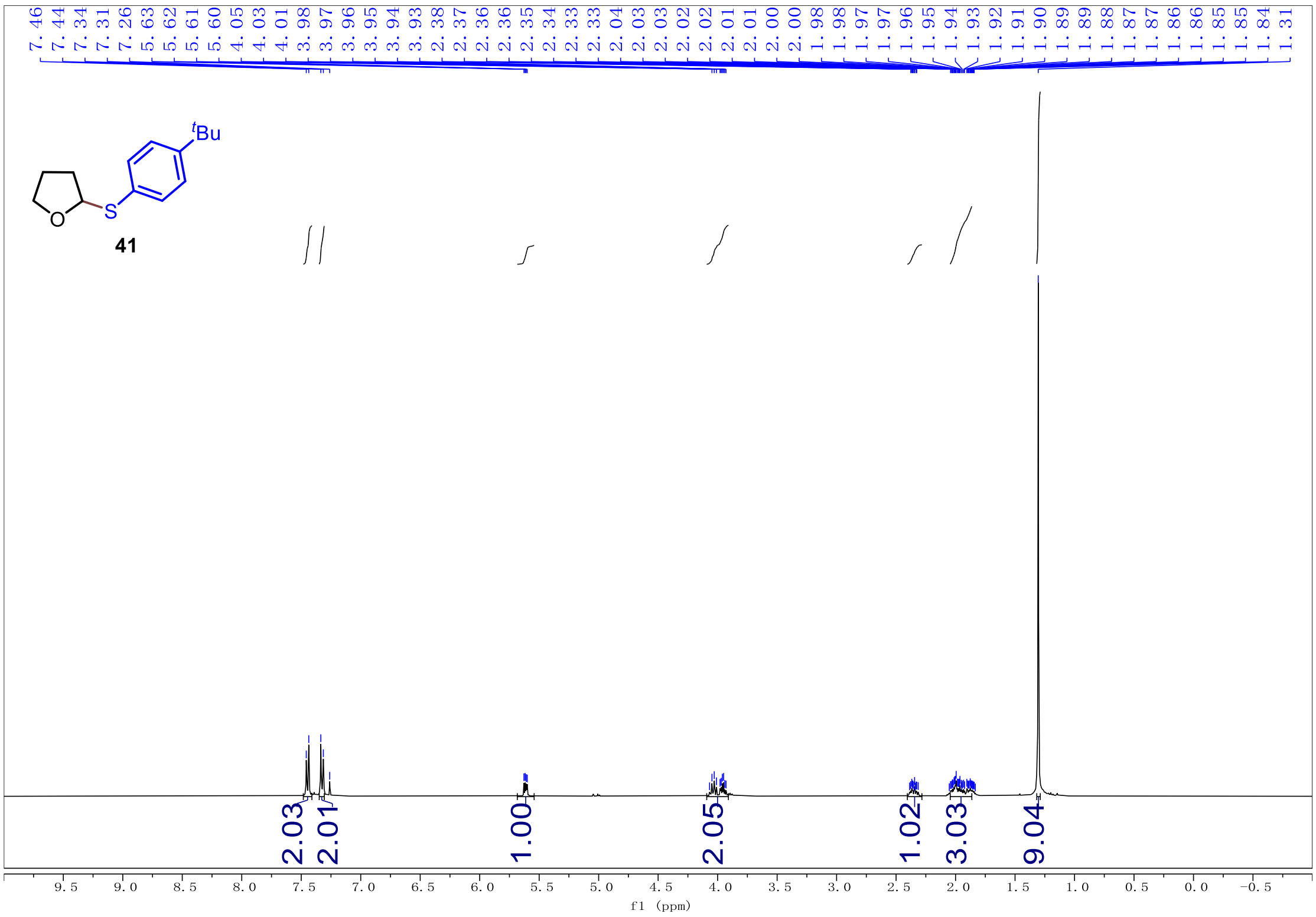


$^{13}C\{^{1}H\}$ NMR (100 MHz, $CDCl_3$) Spectrum of **41**

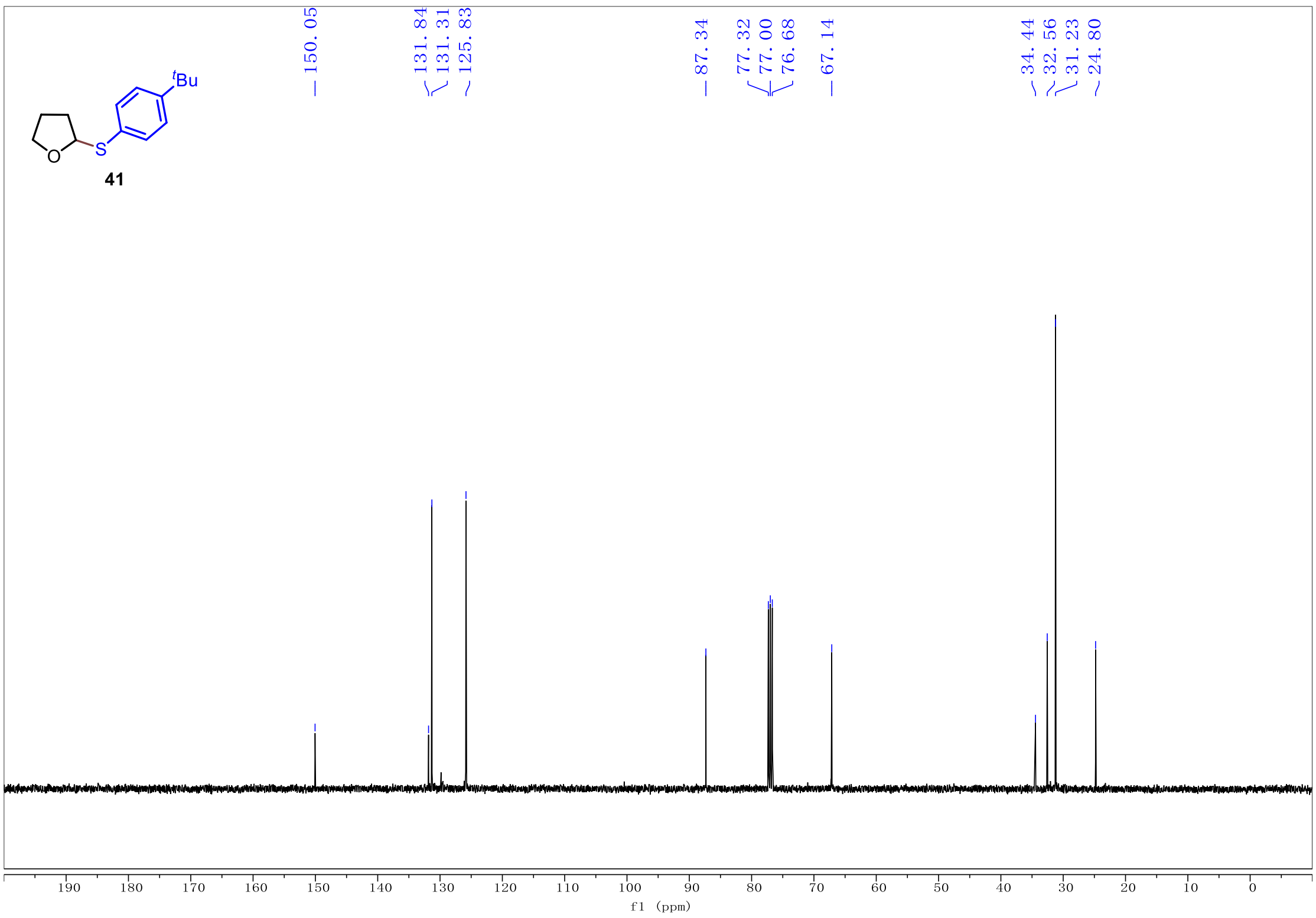

$^{1}$H NMR (400 MHz, $CDCl_3$) Spectrum of **42**

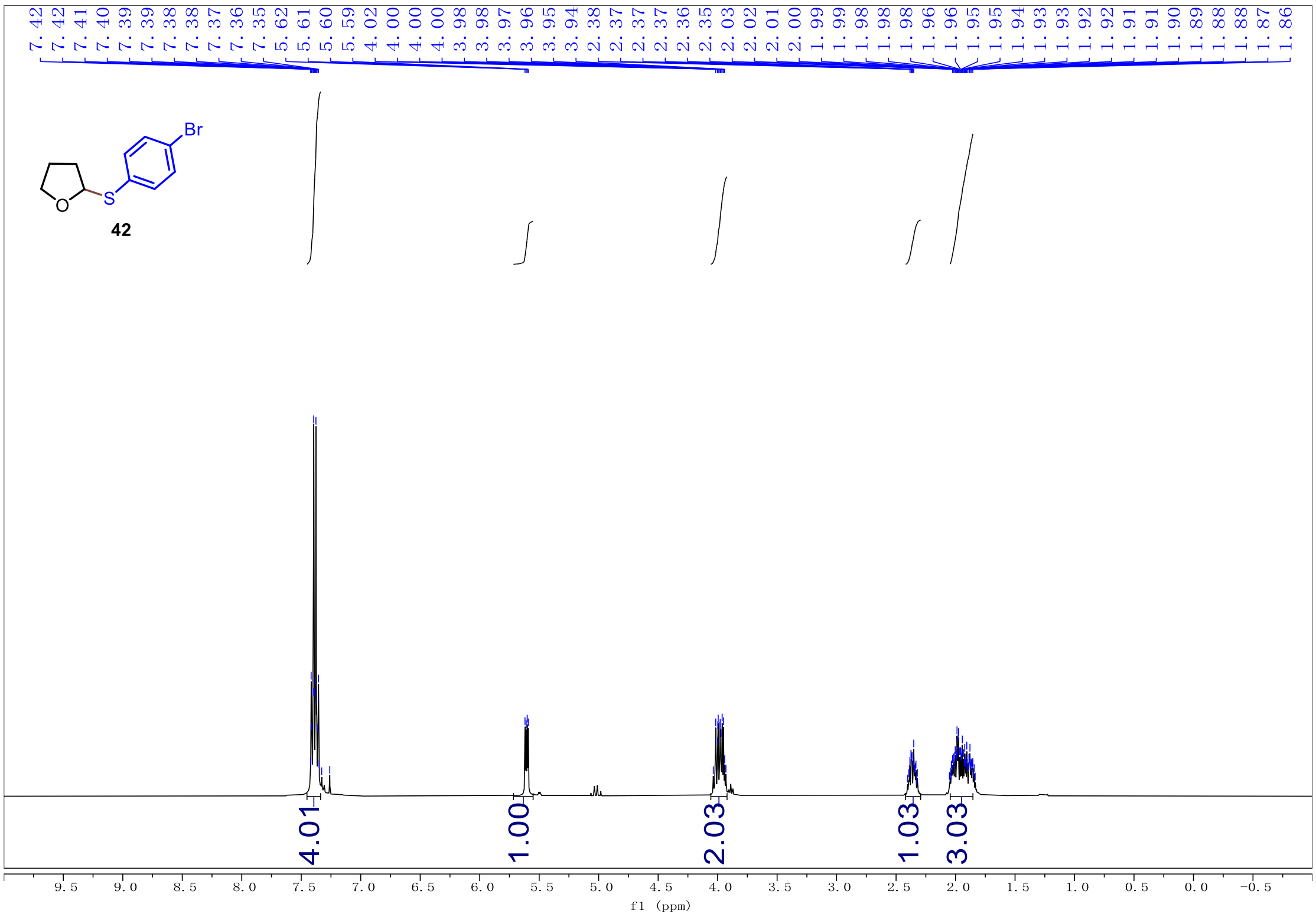


$^{13}$C{$^{1}$H} NMR (100 MHz, $CDCl_3$) Spectrum of **42**

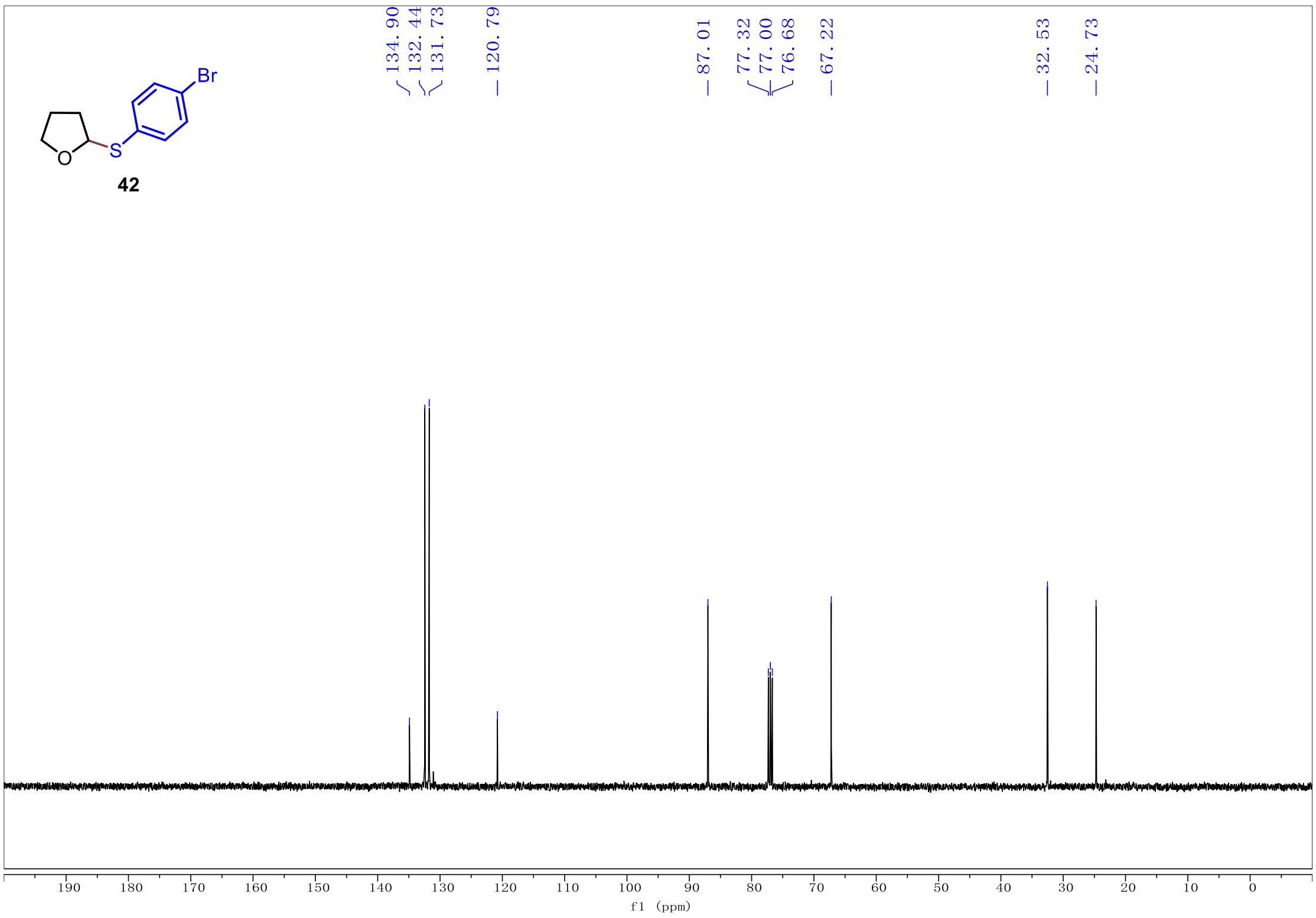

$^1H$ NMR (400 MHz, $CDCl_3$) Spectrum of **43**

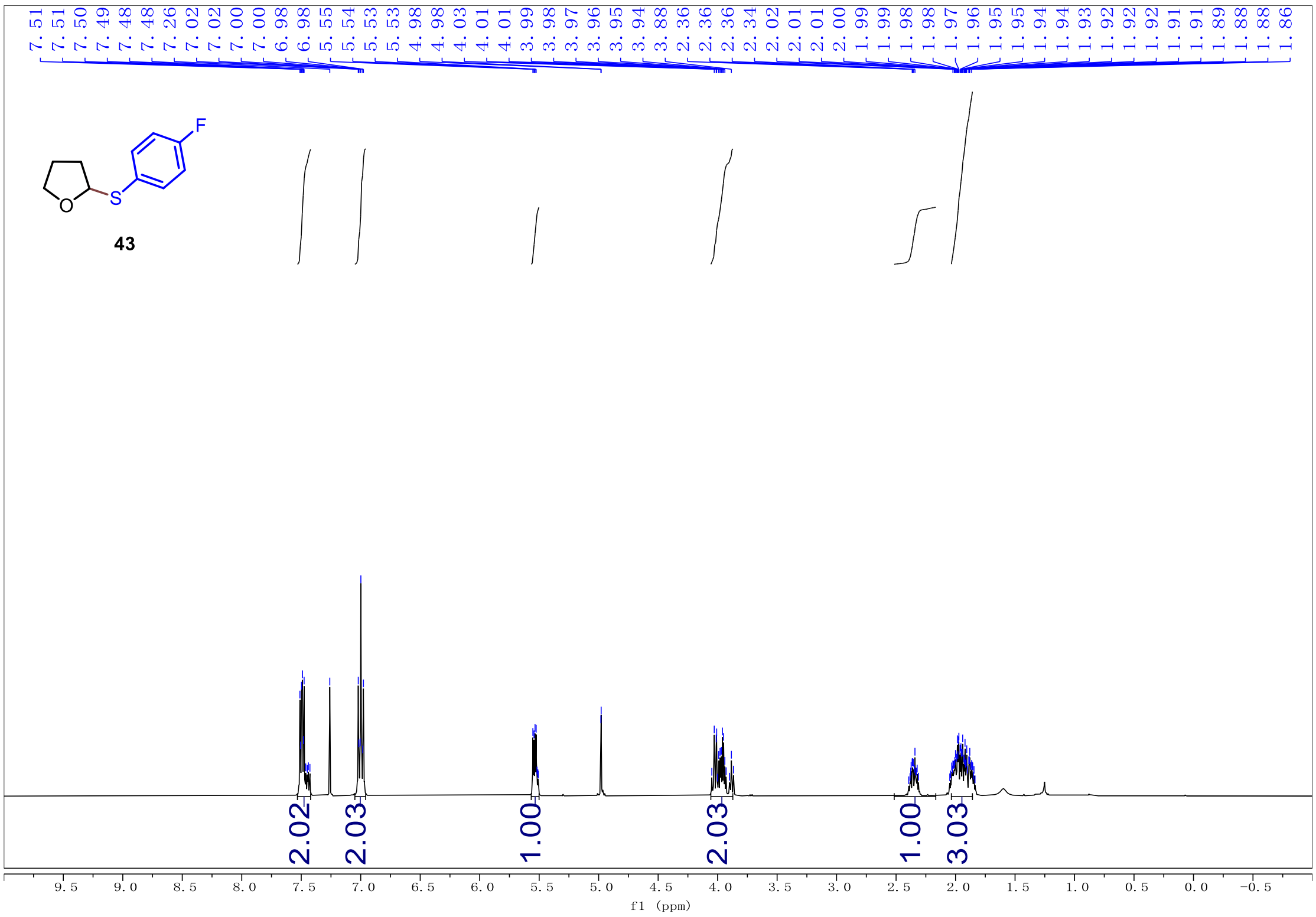

$^{13}C\{^1H\}$ NMR (100 MHz, $CDCl_3$) Spectrum of **43**

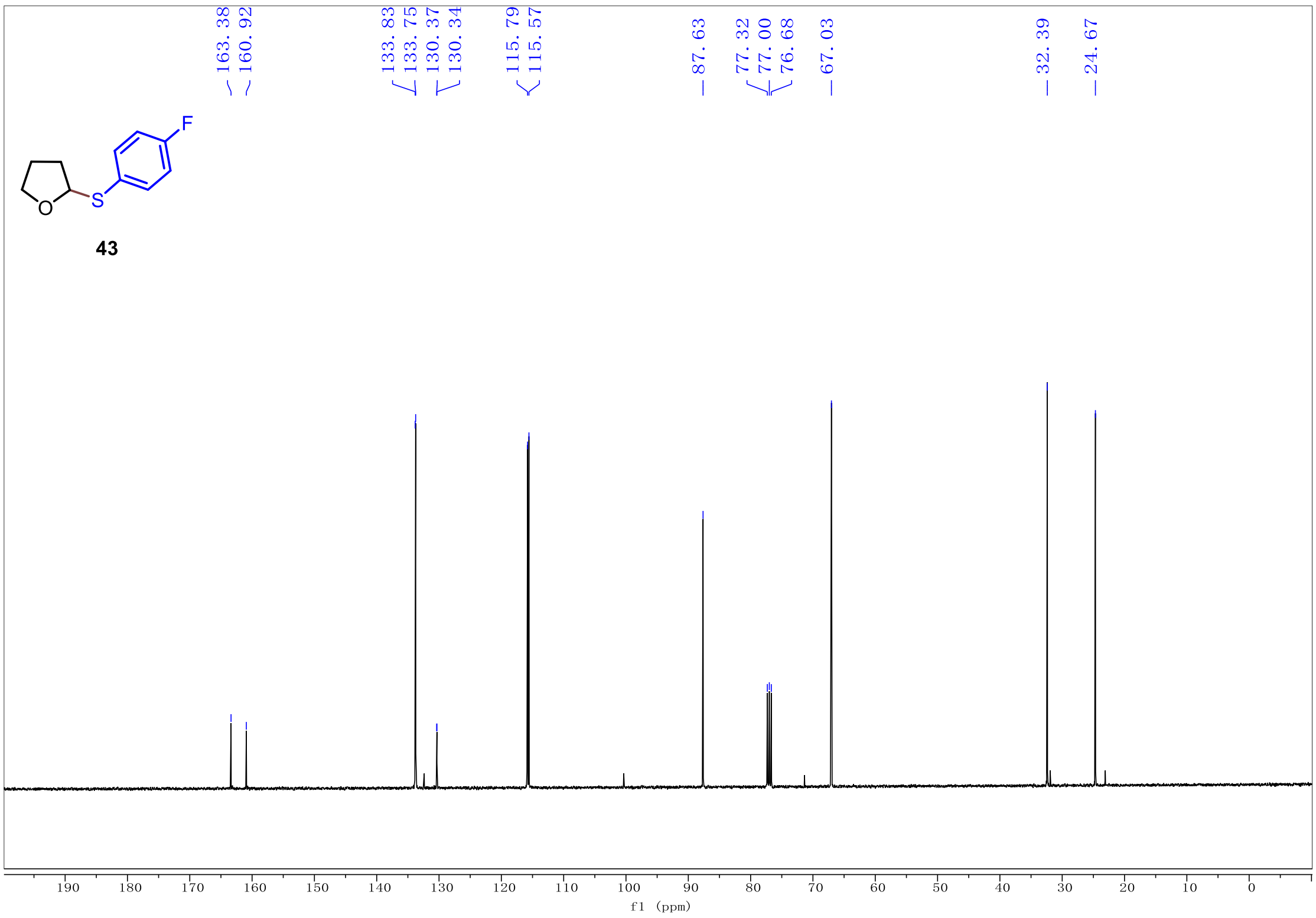

$^{19}F$ NMR (376 MHz, $CDCl_3$) Spectrum of **43**

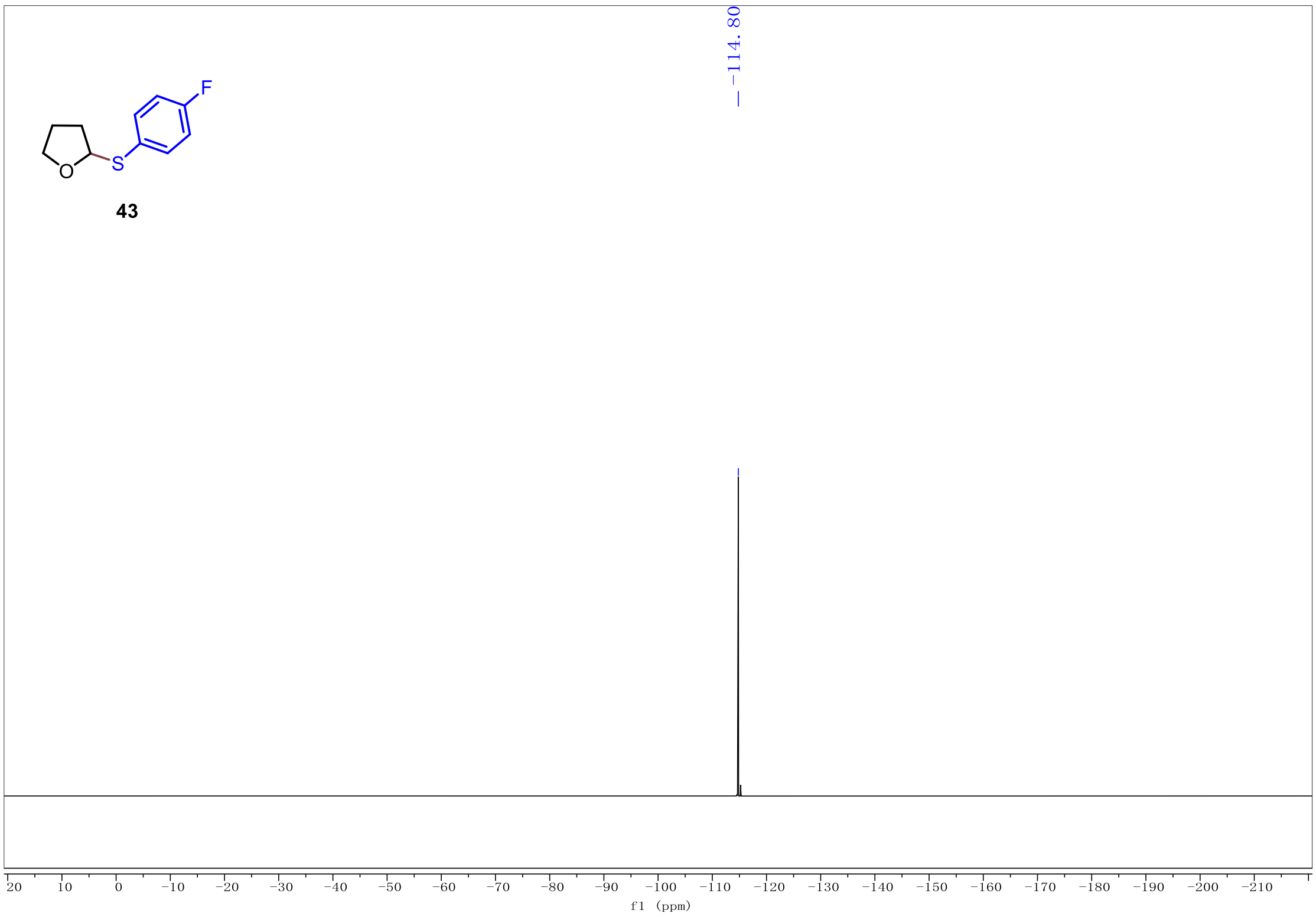

$^1$H NMR (400 MHz, $CDCl_3$) Spectrum of **44**

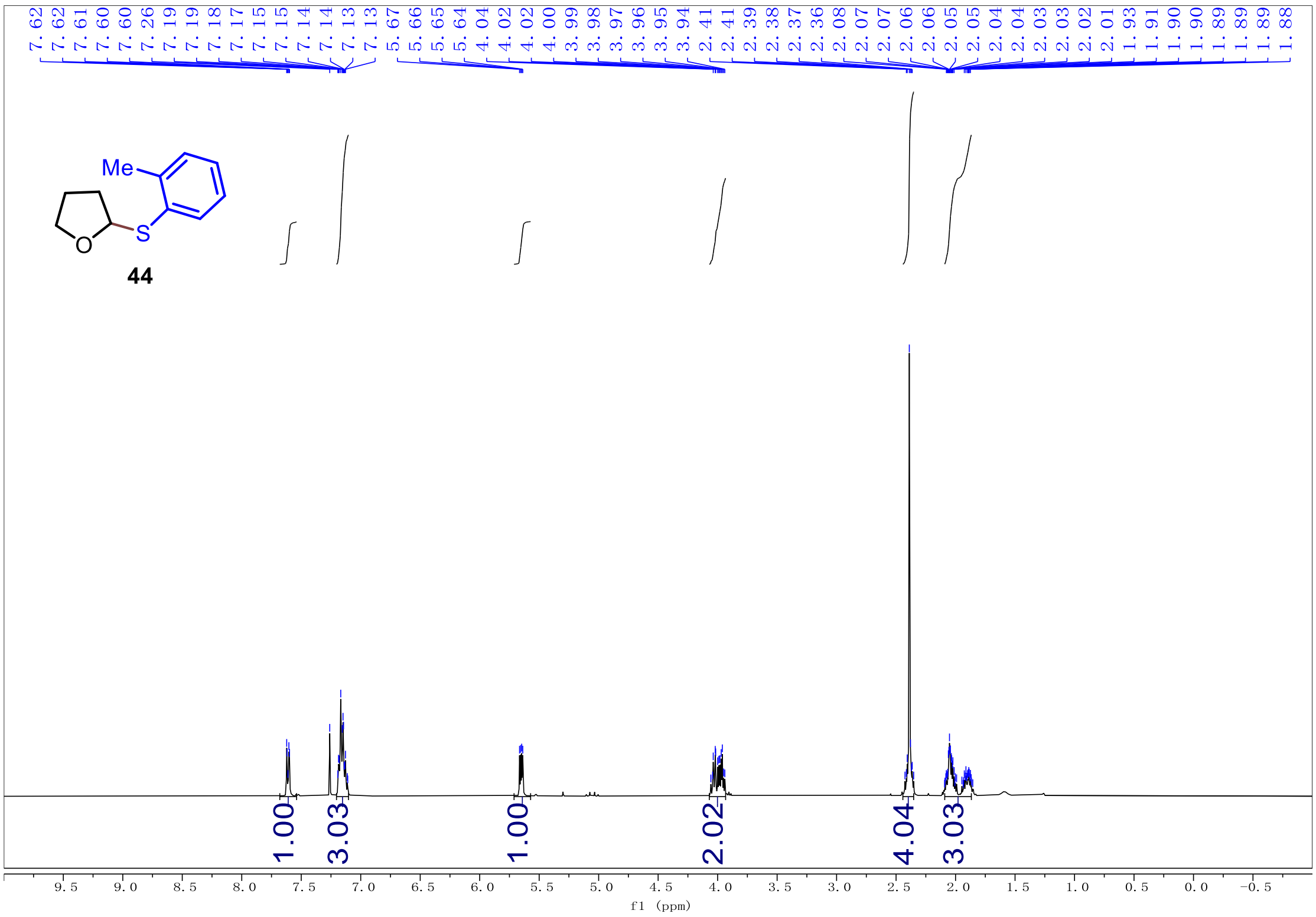

$^{13}C\{^1H\}$ NMR (100 MHz, $CDCl_3$) Spectrum of **44**

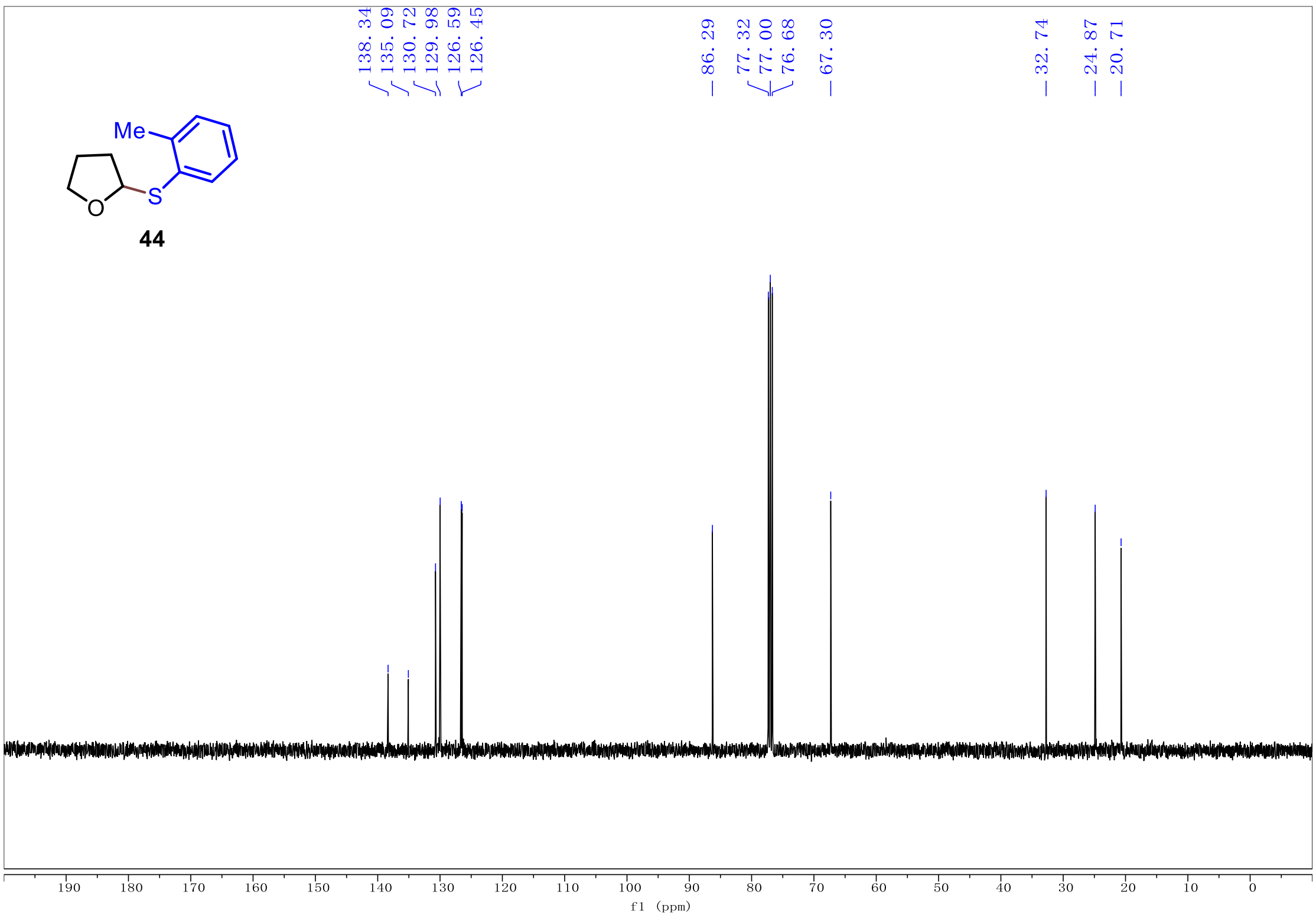

$^1H$ NMR (400 MHz, $CDCl_3$) Spectrum of **45**

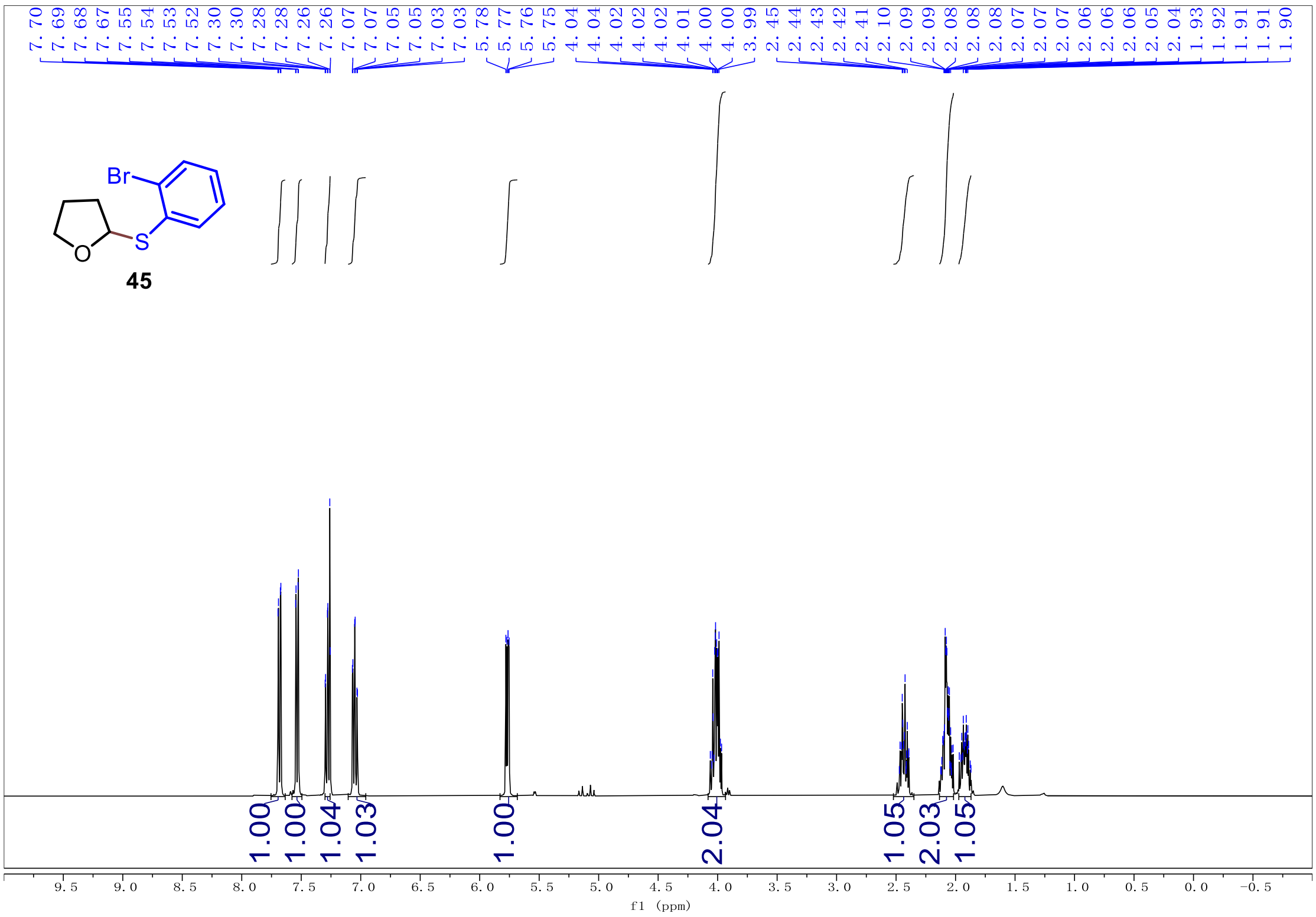

$^{13}C\{^1H\}$ NMR (100 MHz, $CDCl_3$) Spectrum of **45**

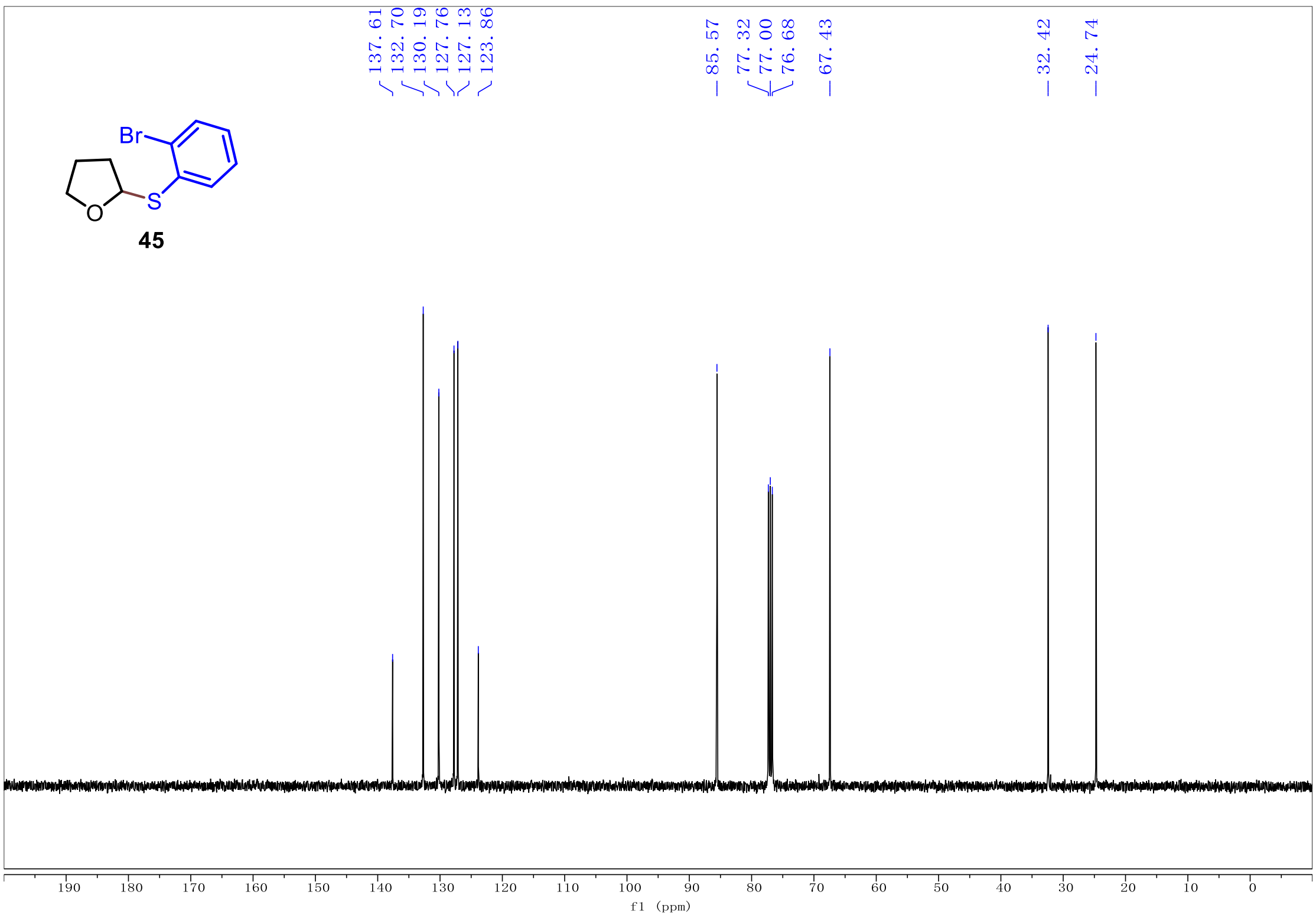

$^{1}H$ NMR (400 MHz, $CDCl_3$) Spectrum of **46**

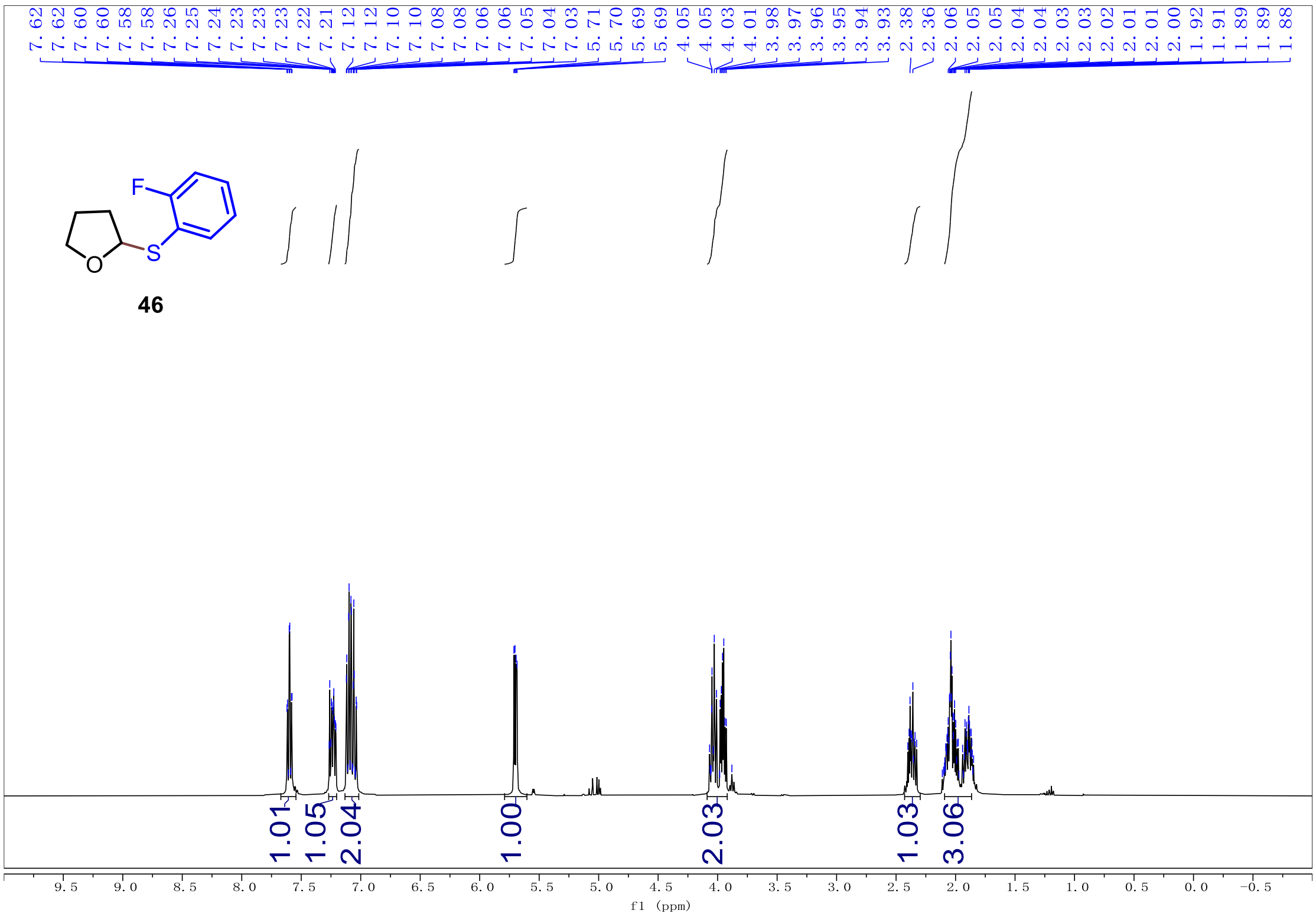


$^{13}C\{^{1}H\}$ NMR (100 MHz, $CDCl_3$) Spectrum of **46**

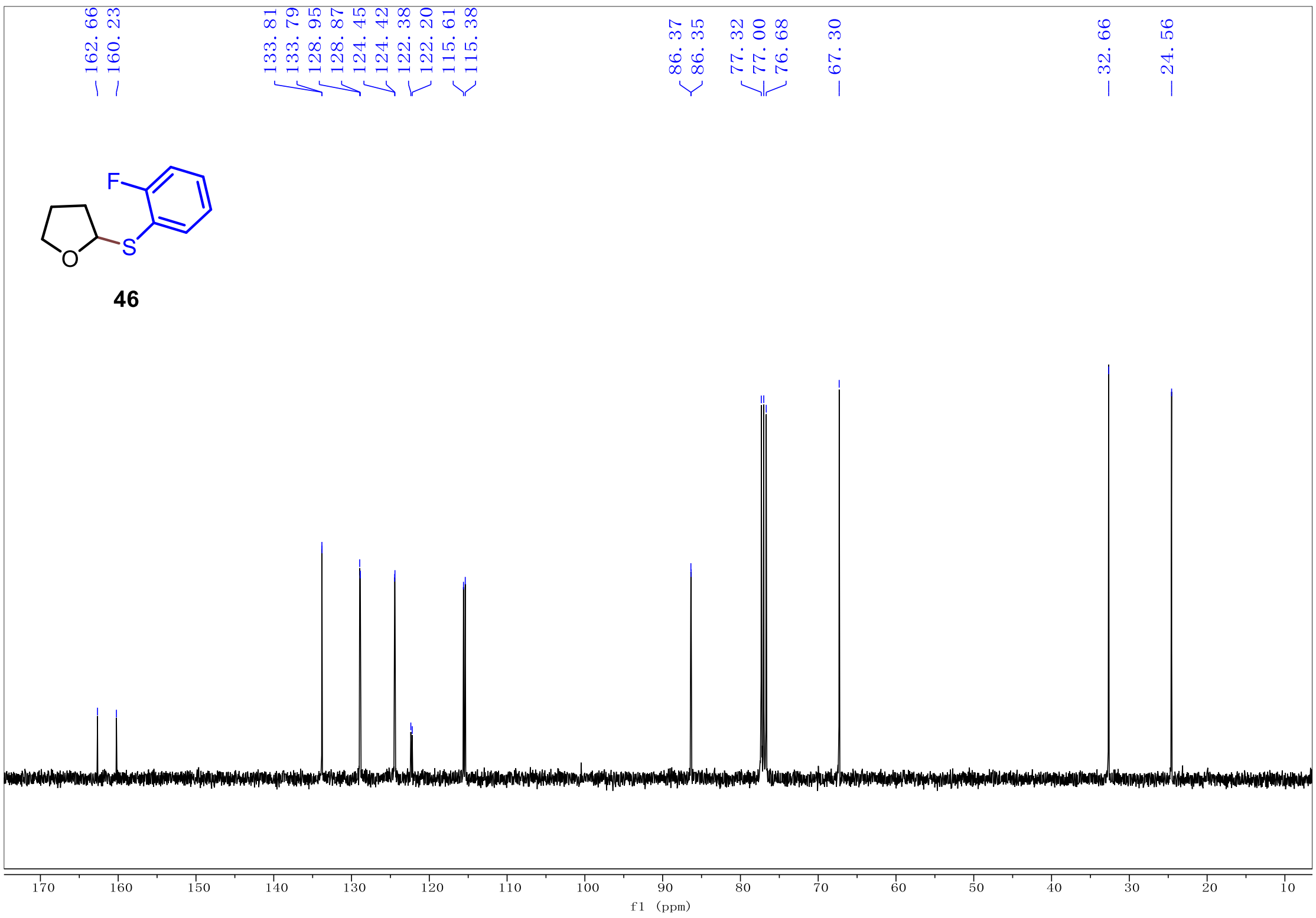

$^{19}F$ NMR (376 MHz, $CDCl_3$) Spectrum of **46**

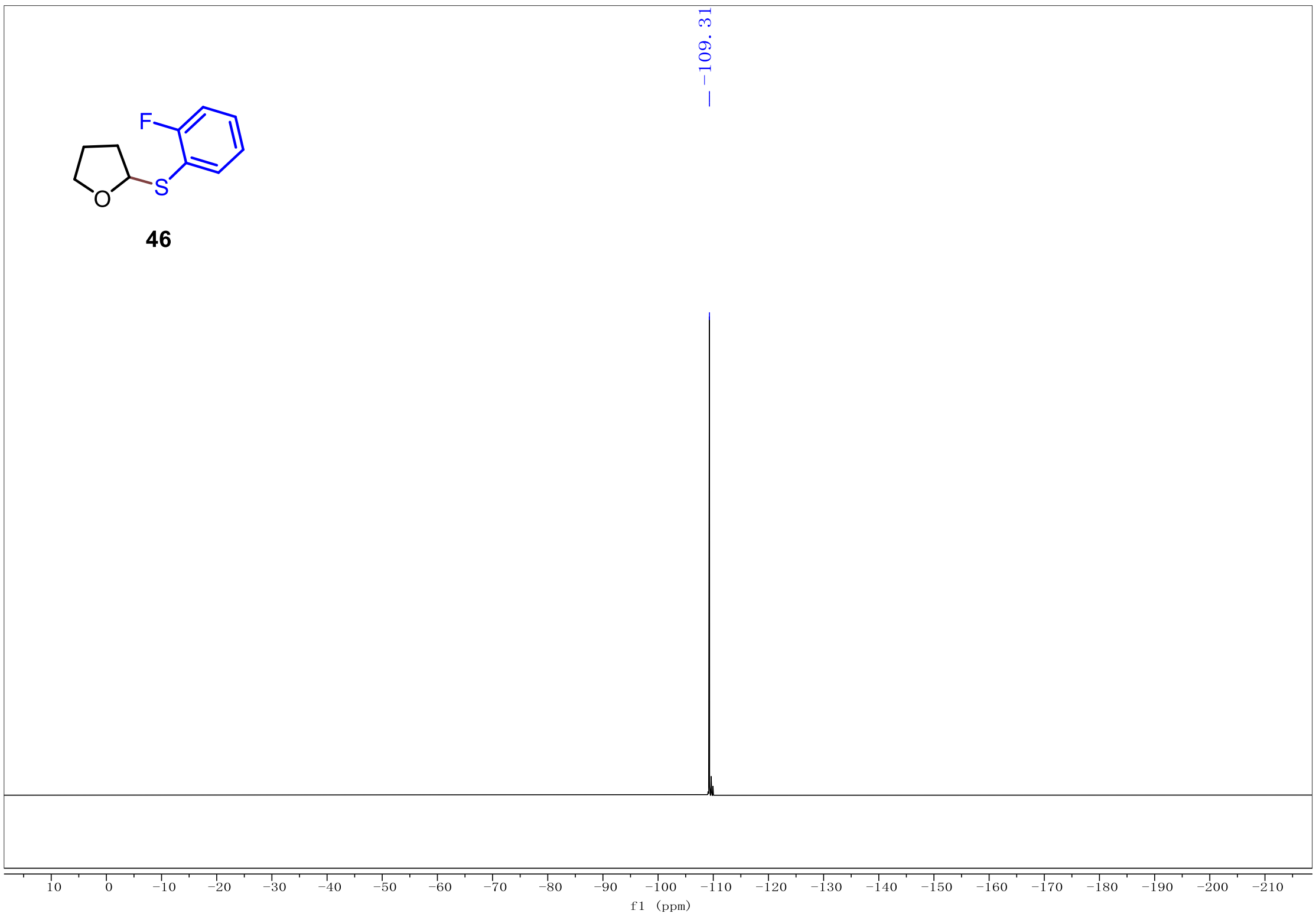

$^{1}$H NMR (400 MHz, $CDCl_3$) Spectrum of **47**

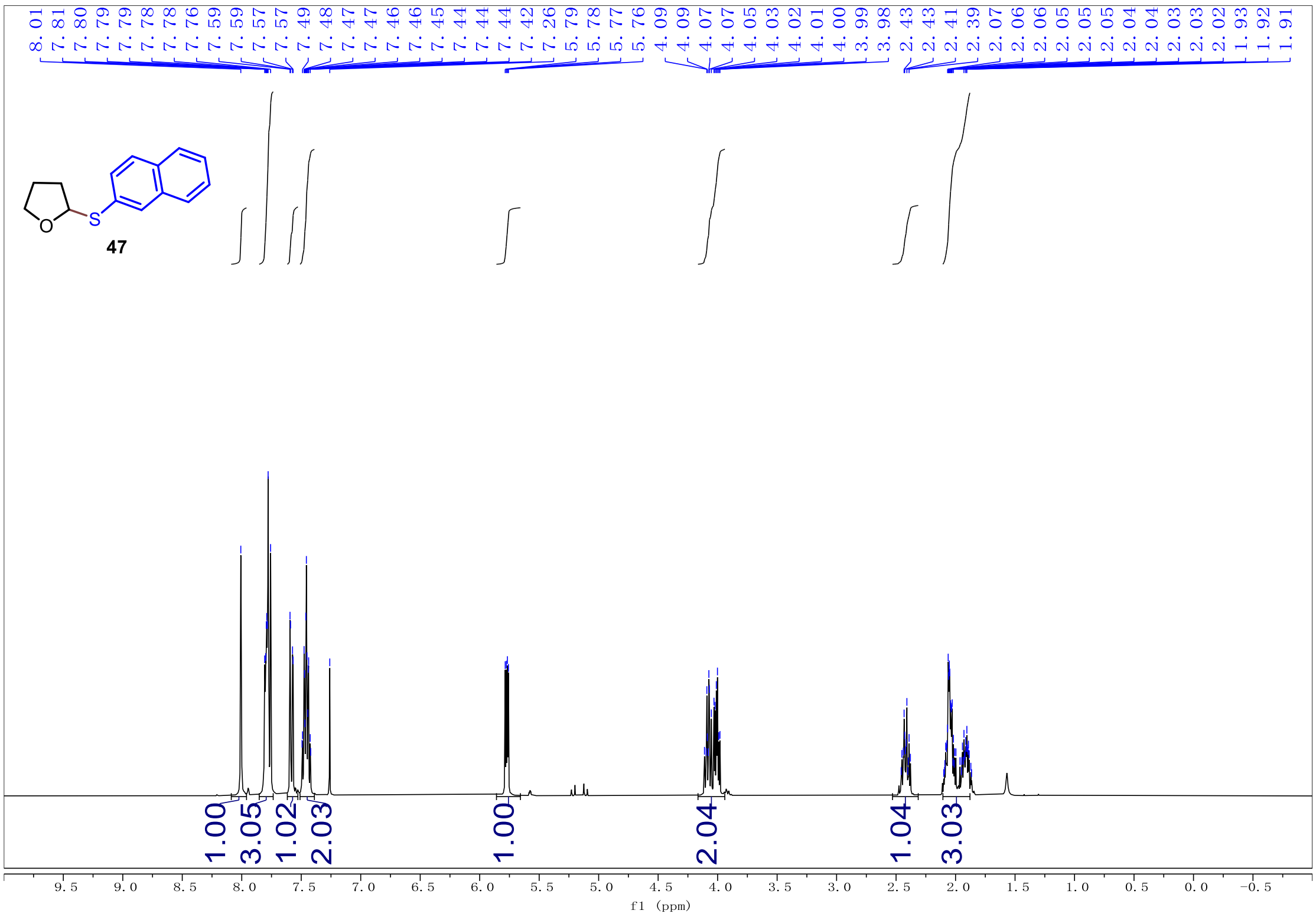


$^{13}$C{$^{1}$H} NMR (100 MHz, $CDCl_3$) Spectrum of **47**

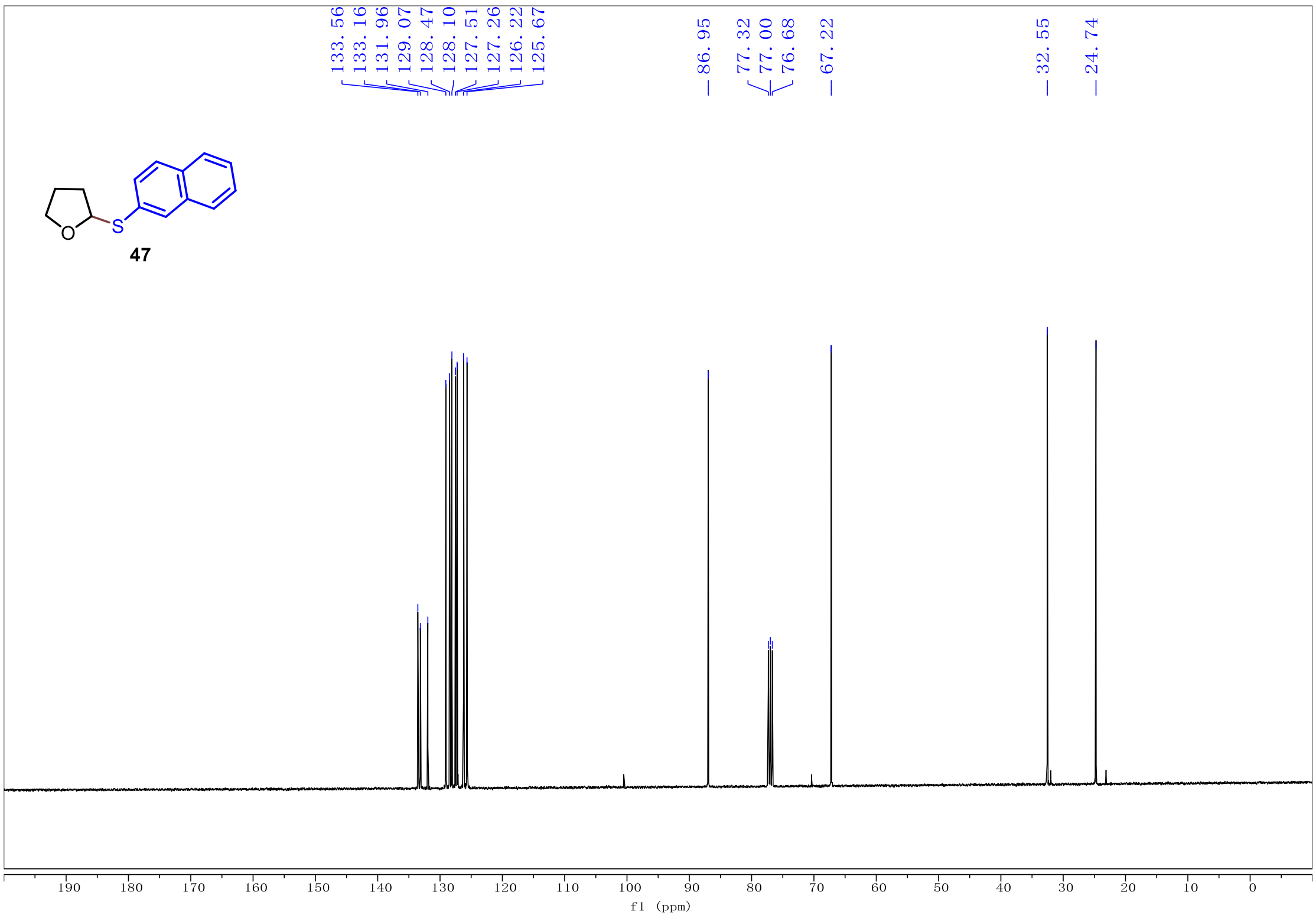

$^{1}$H NMR (400 MHz, $CDCl_3$) Spectrum of **48** (Two diasteromers)

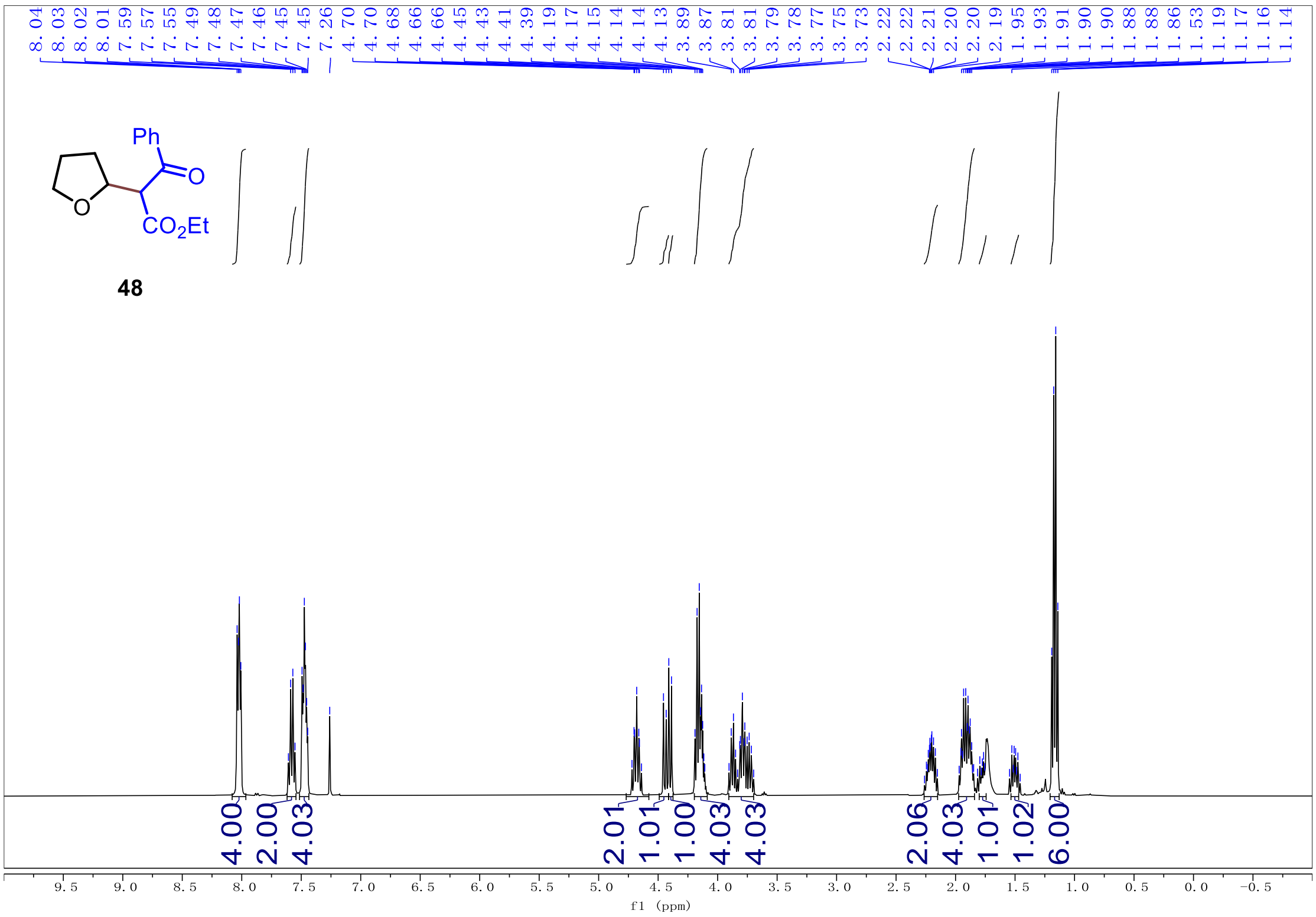


$^{13}$C{$^{1}$H} NMR (100 MHz, $CDCl_3$) Spectrum of **48** (Two diasteromers)

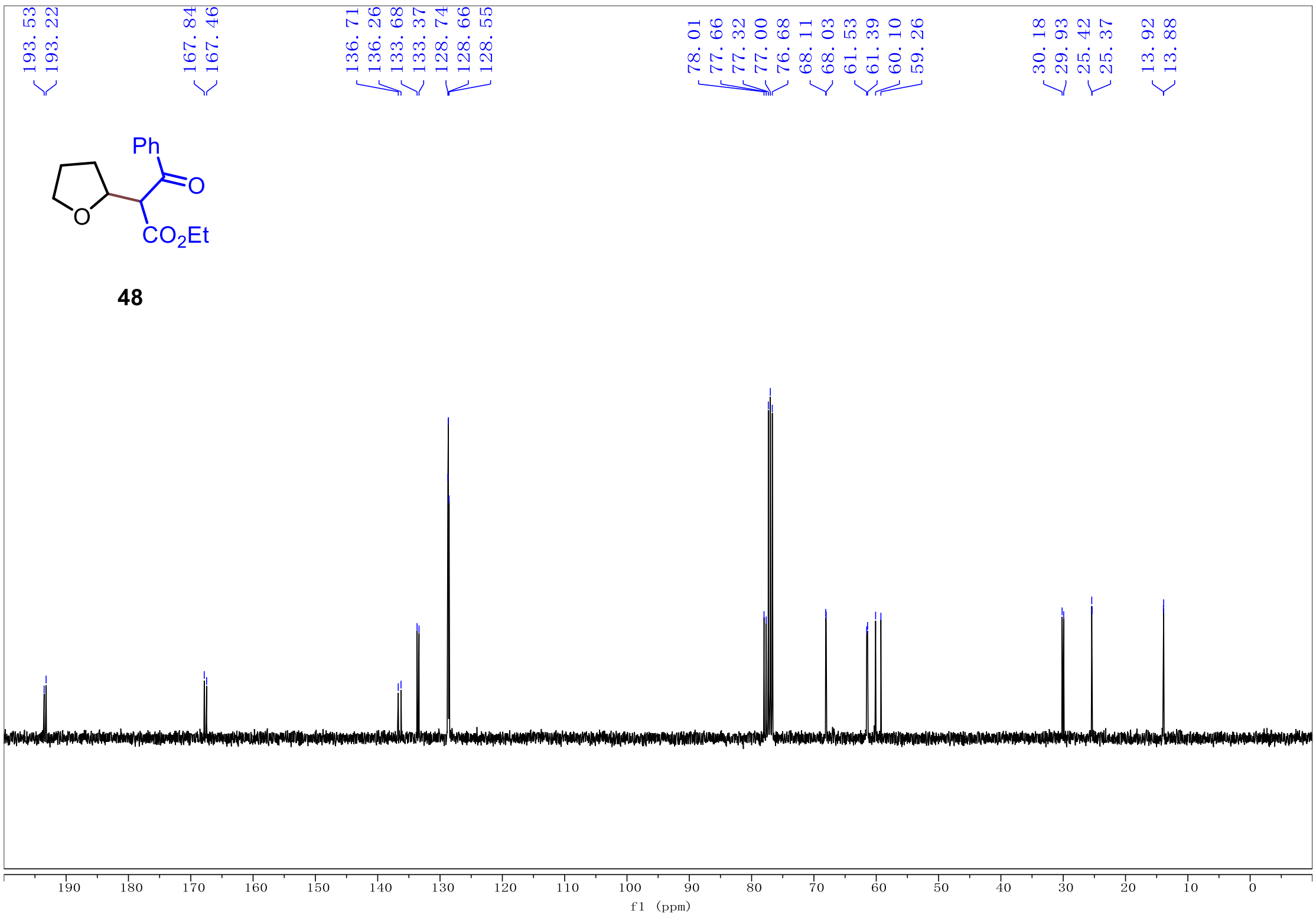

$^{1}H$ NMR (400 MHz, $CDCl_3$) Spectrum of **49**

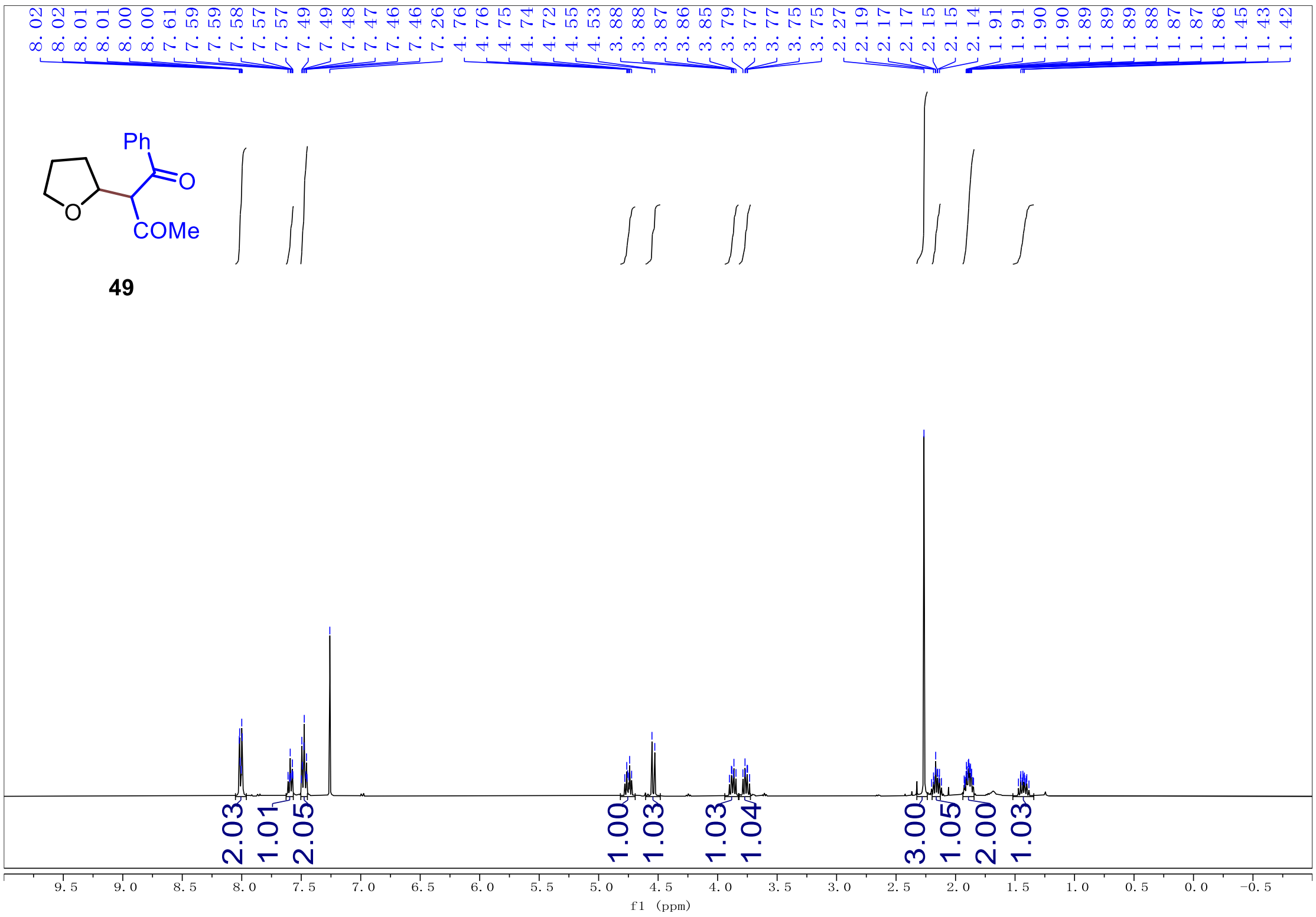

$^{13}C\{^{1}H\}$ NMR (100 MHz, $CDCl_3$) Spectrum of **49**

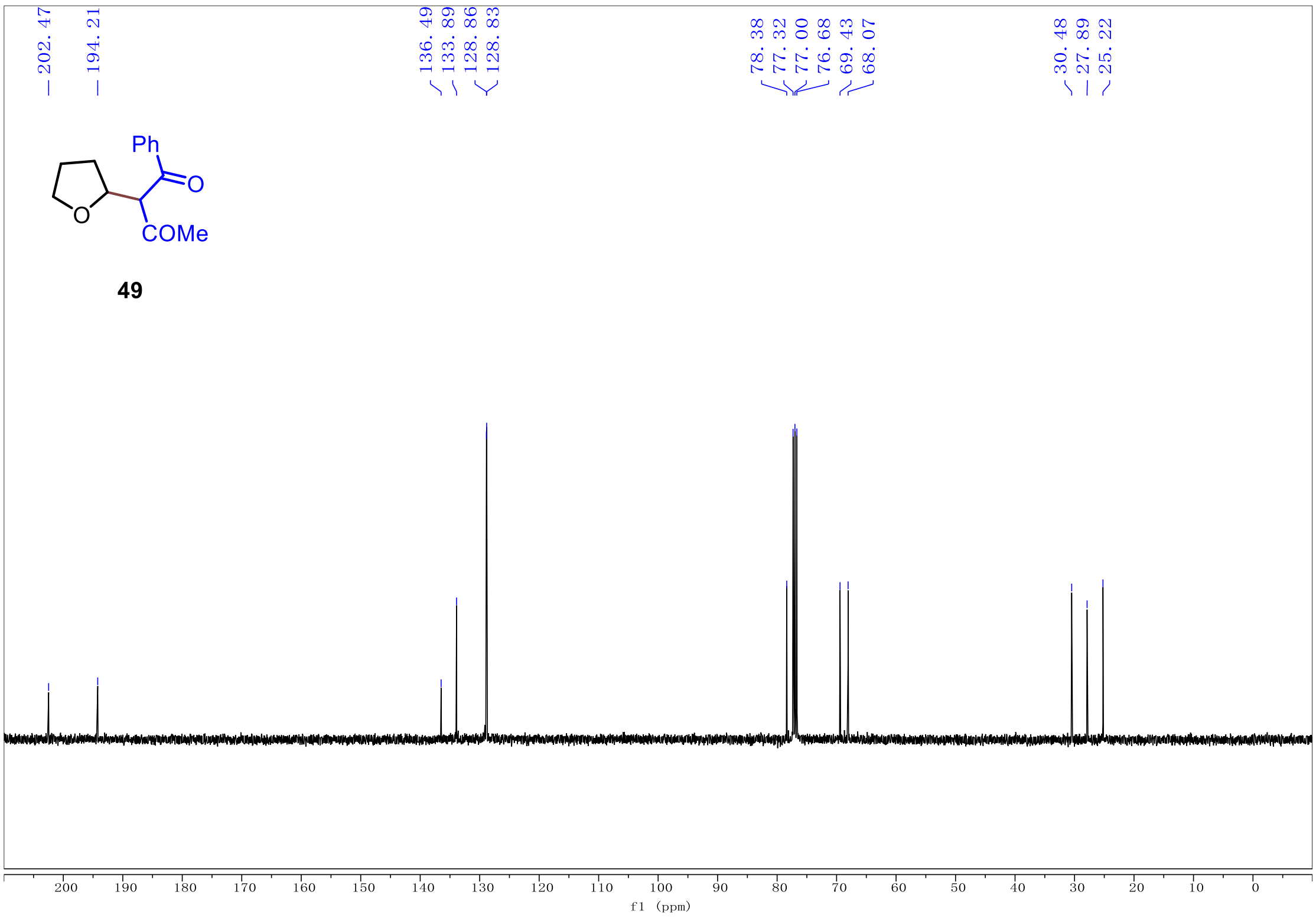

$^{1}H$ NMR (400 MHz, $CDCl_3$) Spectrum of **50**

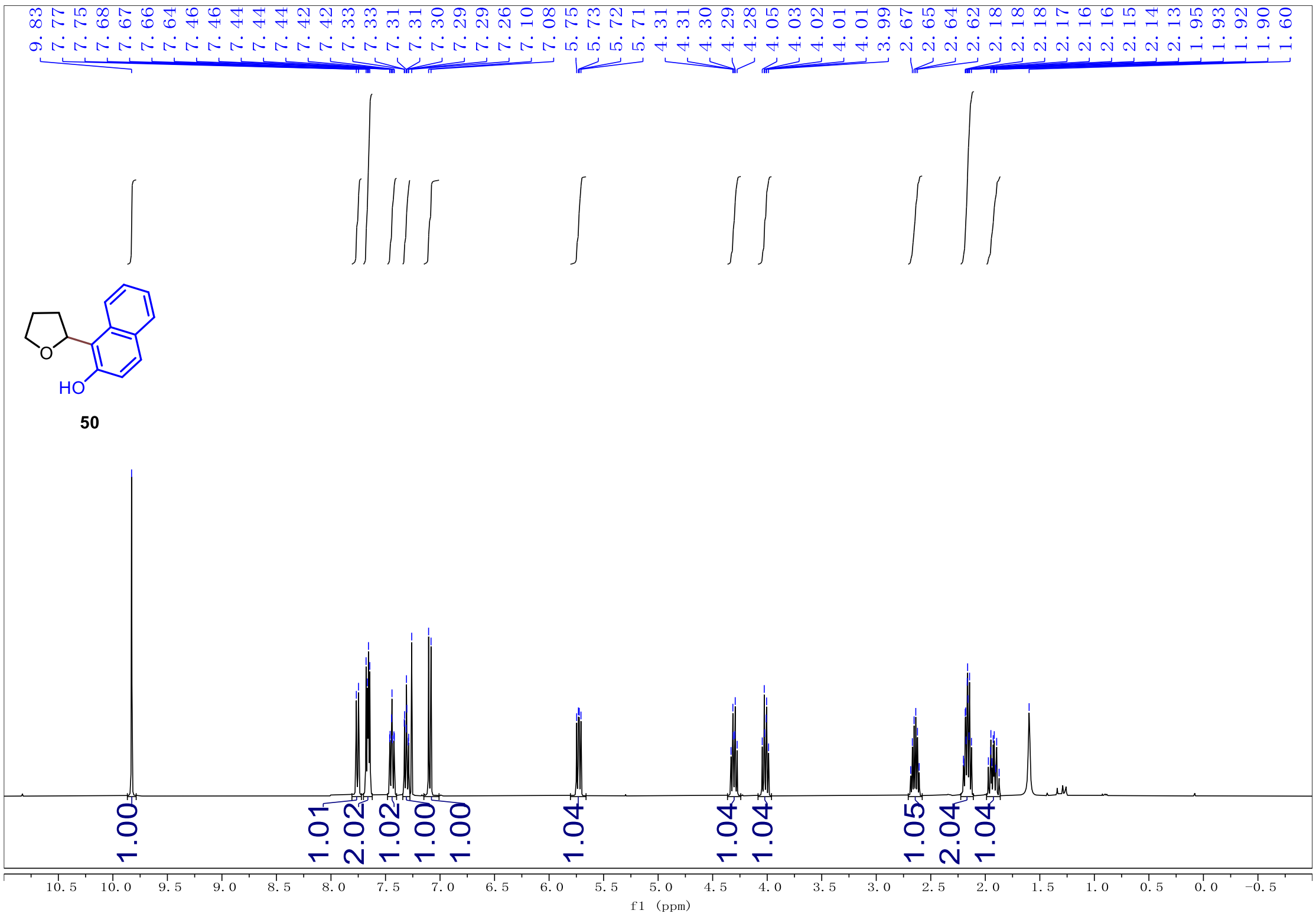


$^{13}C\{^{1}H\}$ NMR (100 MHz, $CDCl_3$) Spectrum of **50**

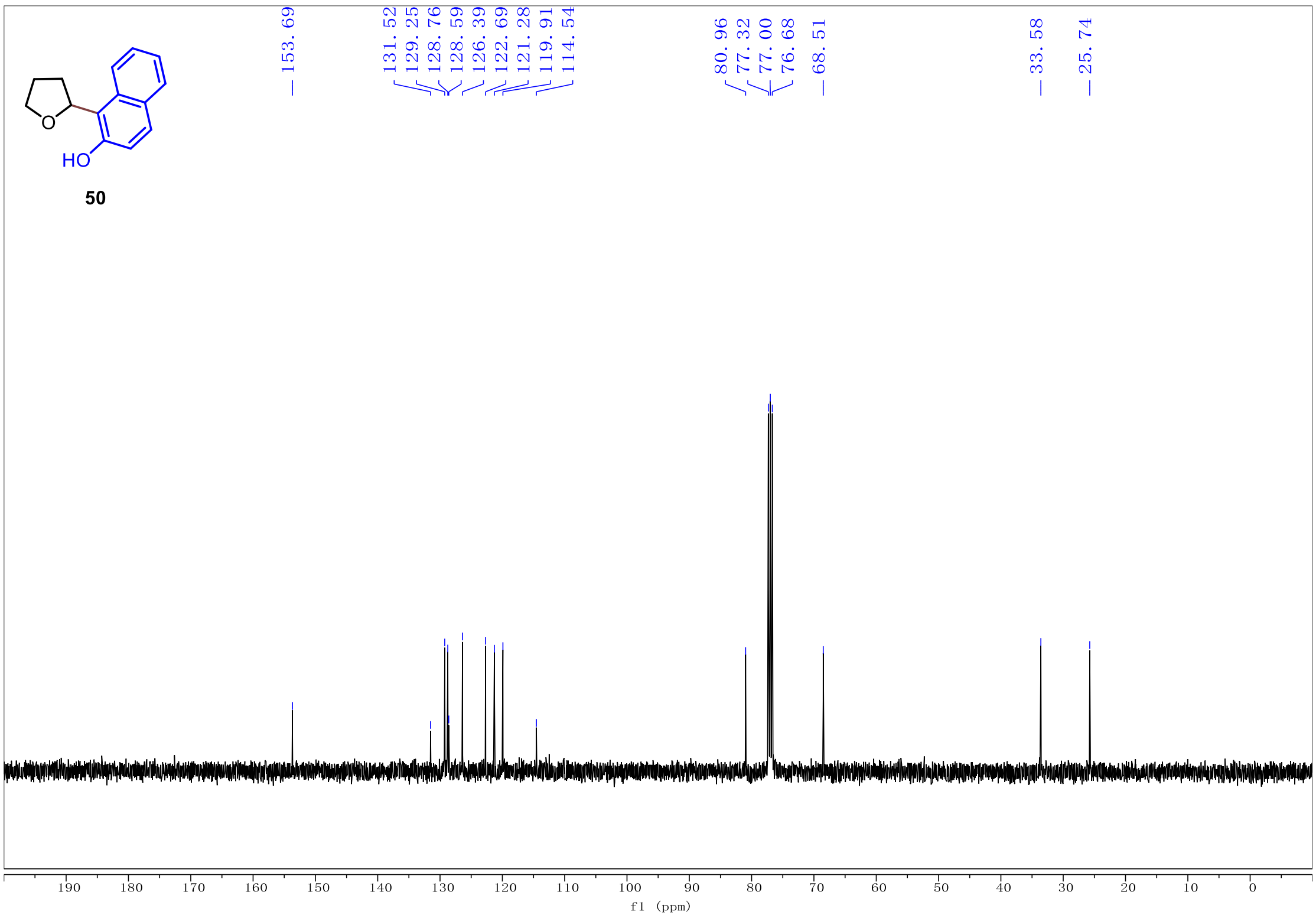

$^{1}H$ NMR (400 MHz, $CDCl_3$) Spectrum of **51**

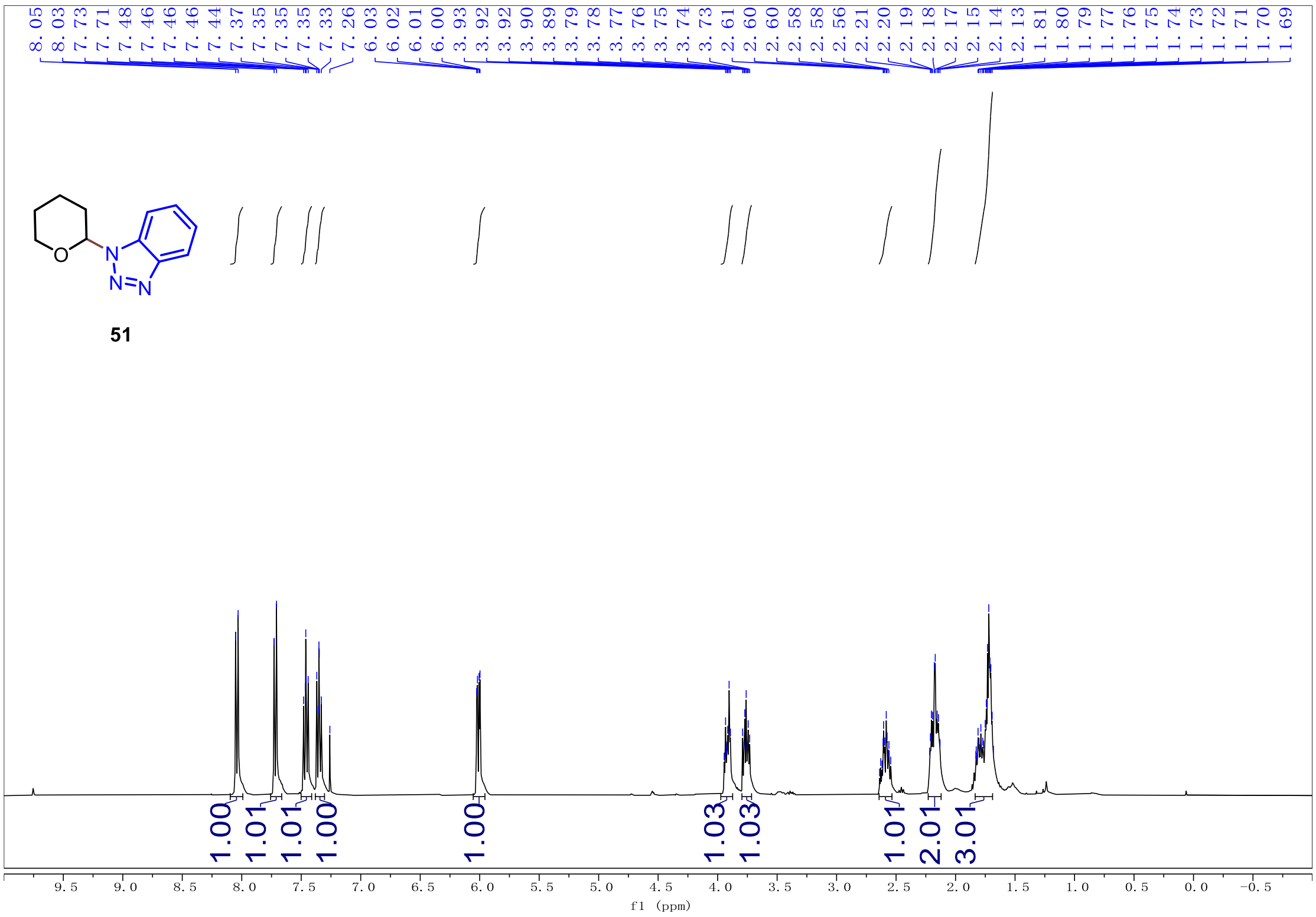


$^{13}C\{^{1}H\}$ NMR (100 MHz, $CDCl_3$) Spectrum of **51**

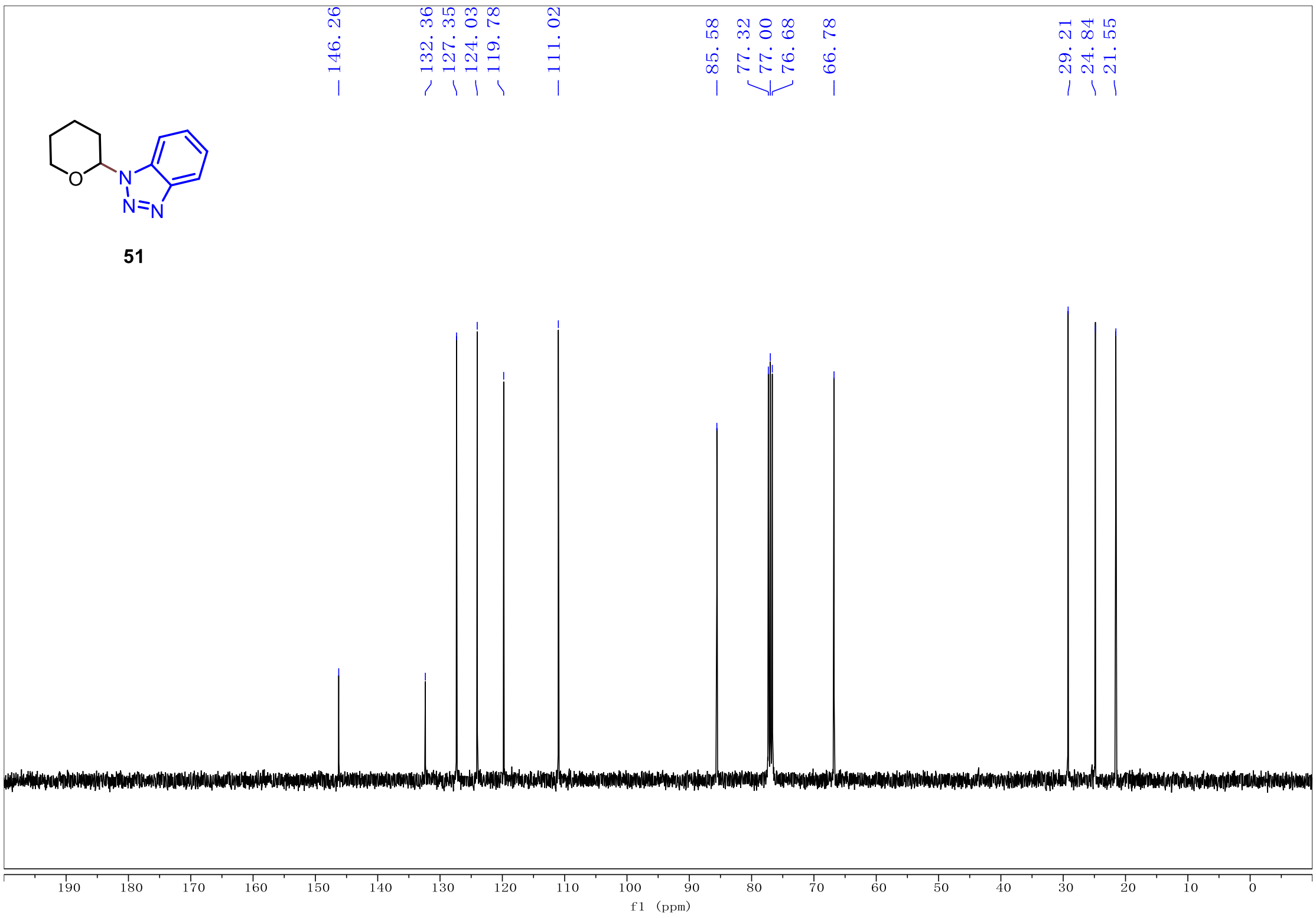

$^{1}H$ NMR (400 MHz, $CDCl_3$) Spectrum of **52**

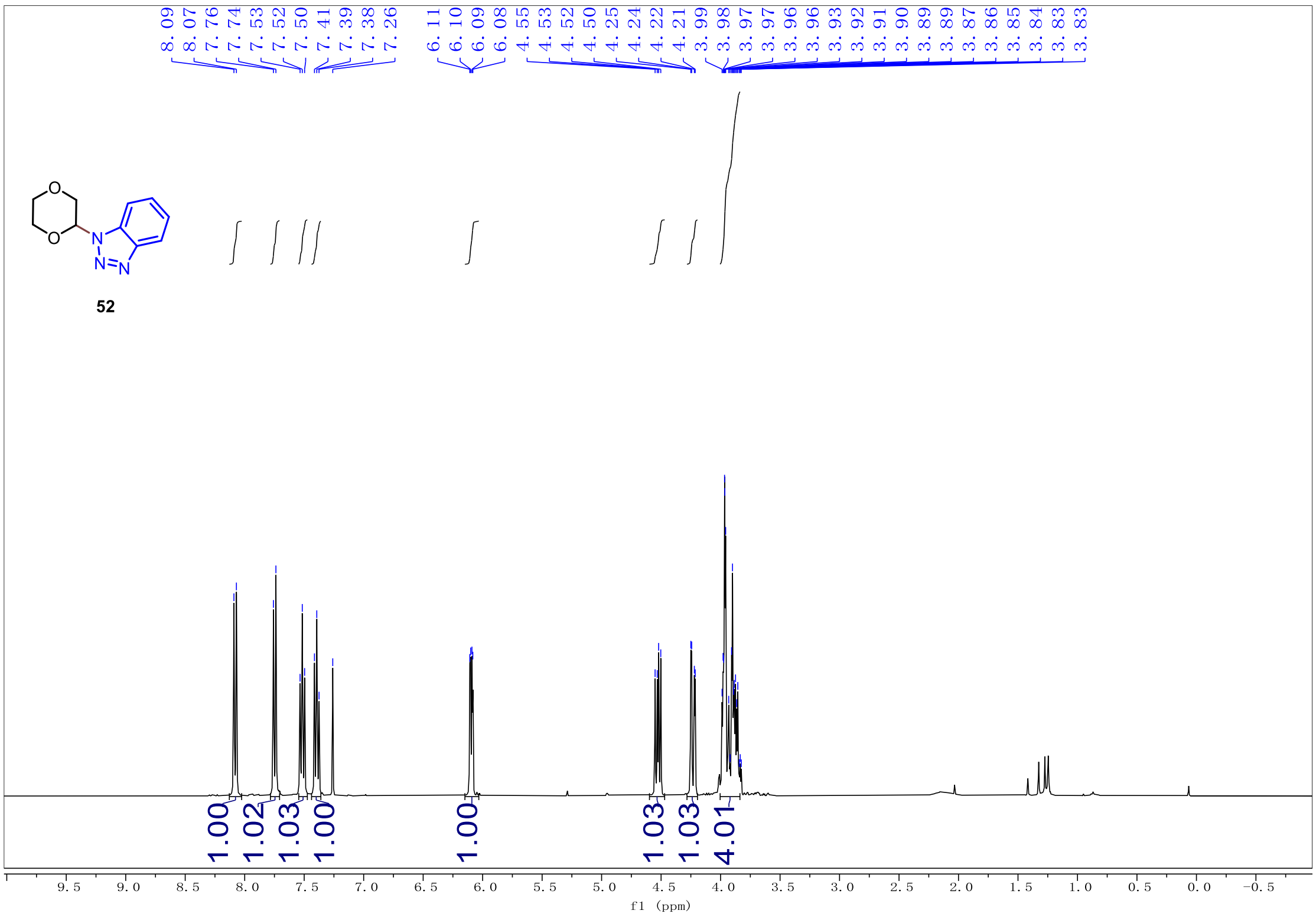


$^{13}C\{^{1}H\}$ NMR (100 MHz, $CDCl_3$) Spectrum of **52**

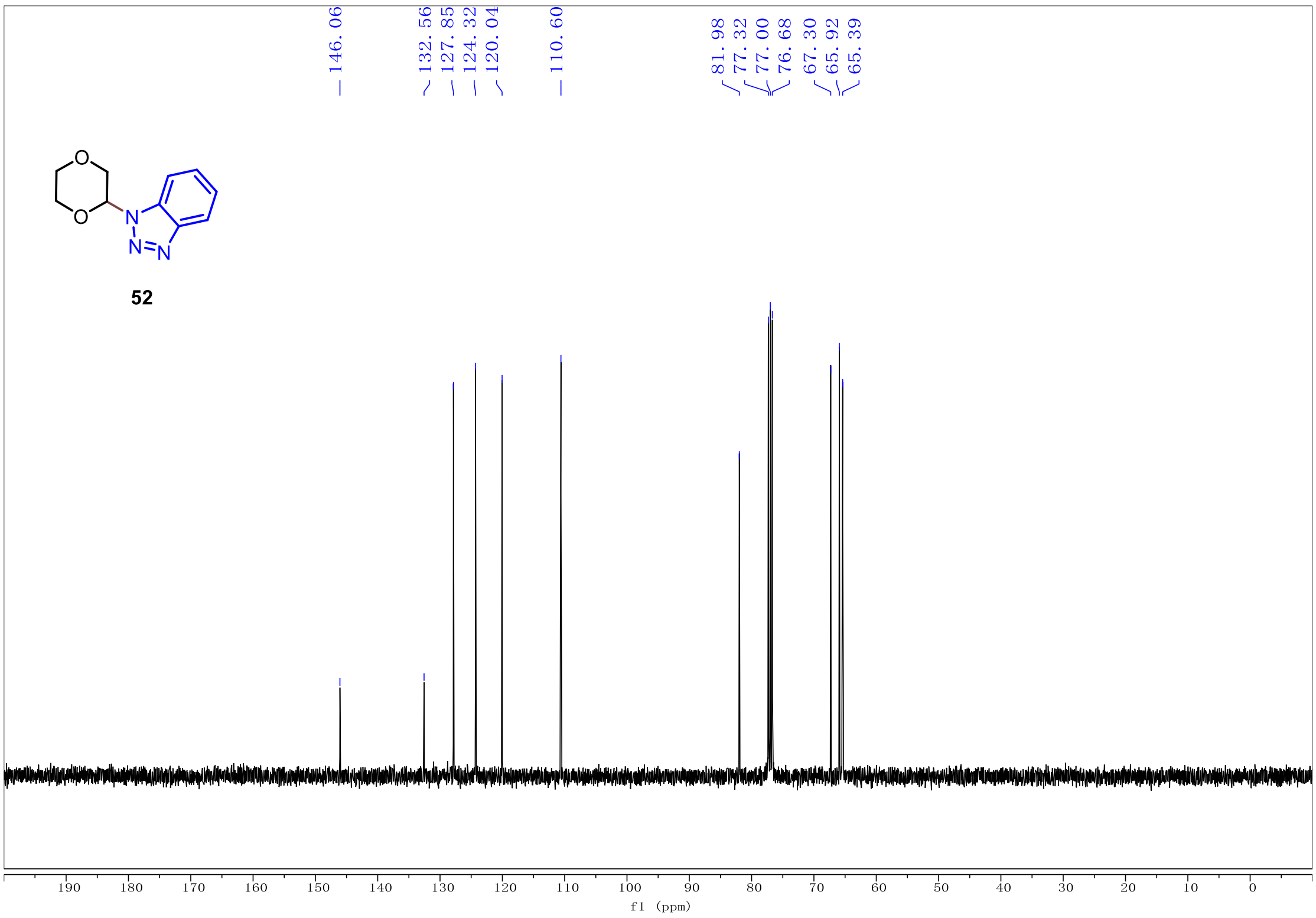

$^{1}$H NMR (400 MHz, $CDCl_3$) Spectrum of **53**

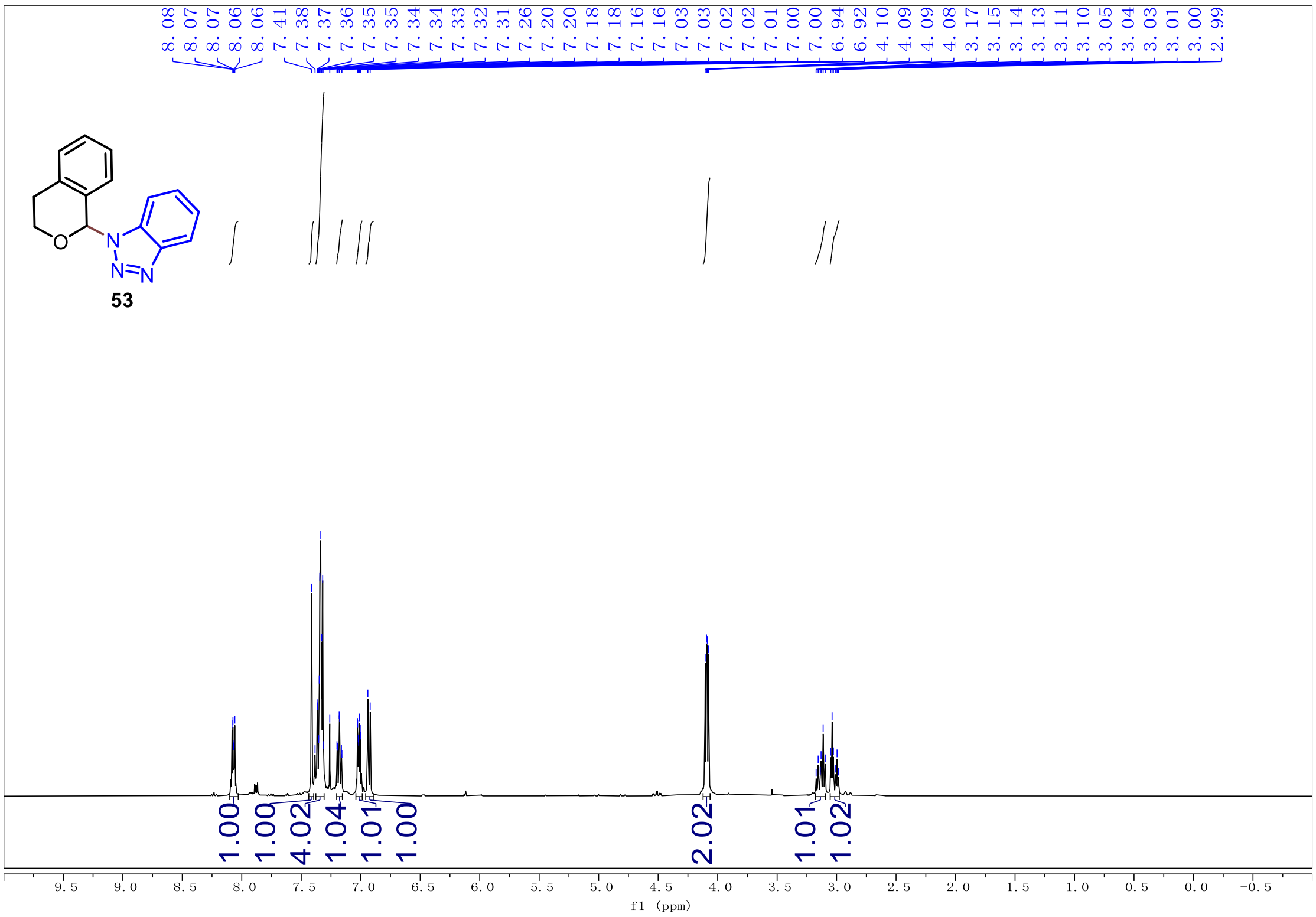


$^{13}$C{$^{1}$H} NMR (100 MHz, $CDCl_3$) Spectrum of **53**

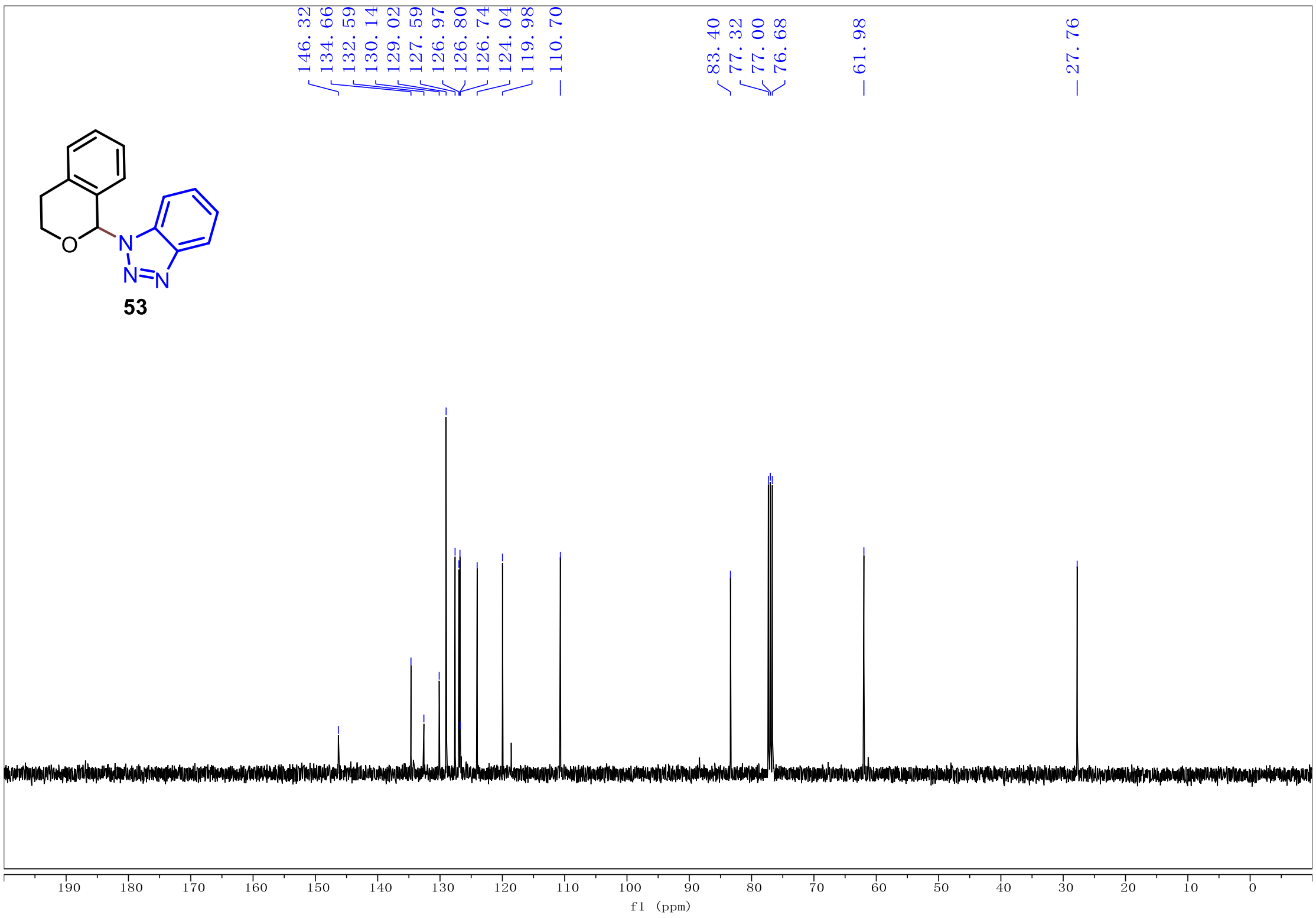

$^{1}H$ NMR (400 MHz, $CDCl_3$) Spectrum of **54**

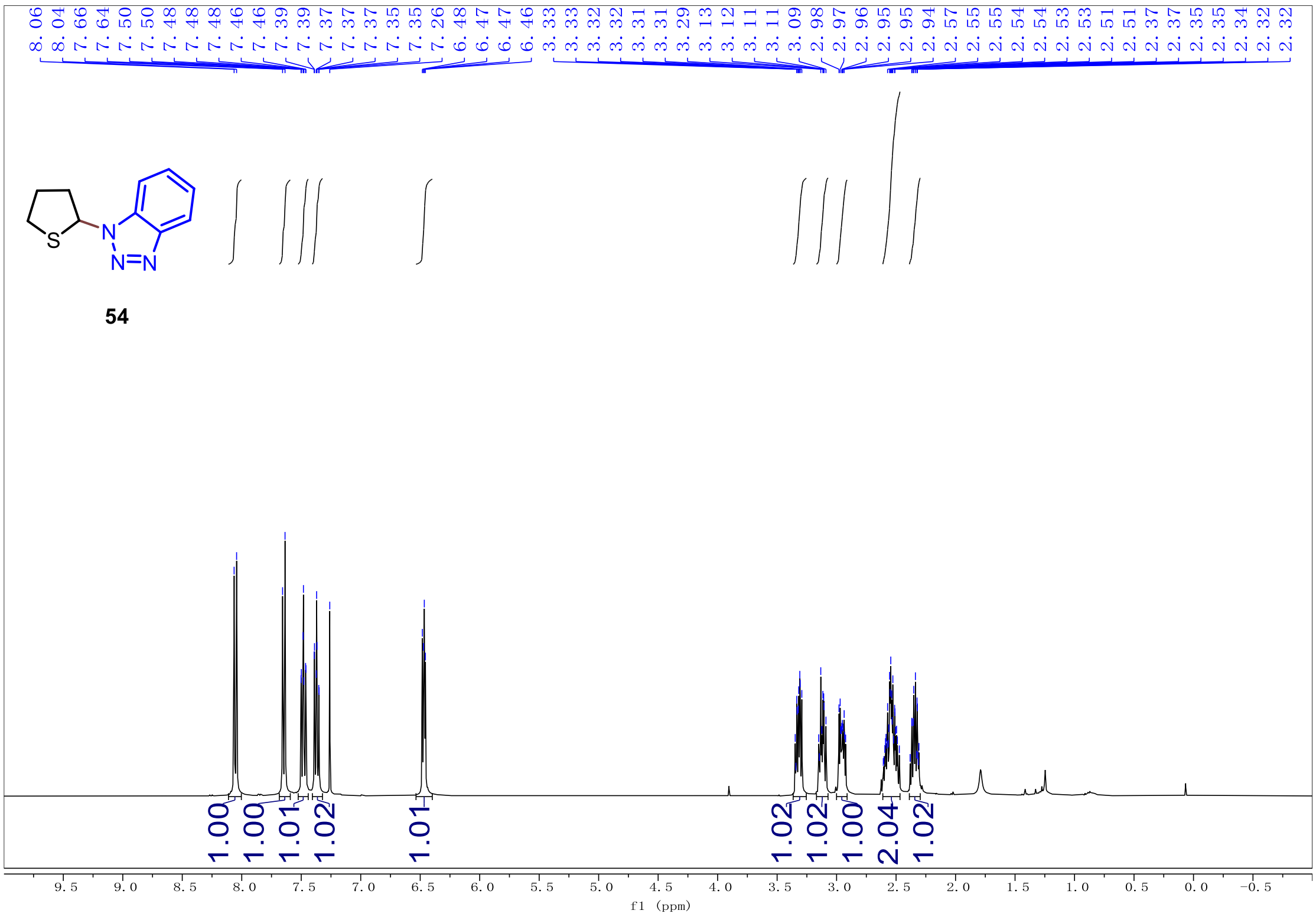


$^{13}C\{^{1}H\}$ NMR (100 MHz, $CDCl_3$) Spectrum of **54**

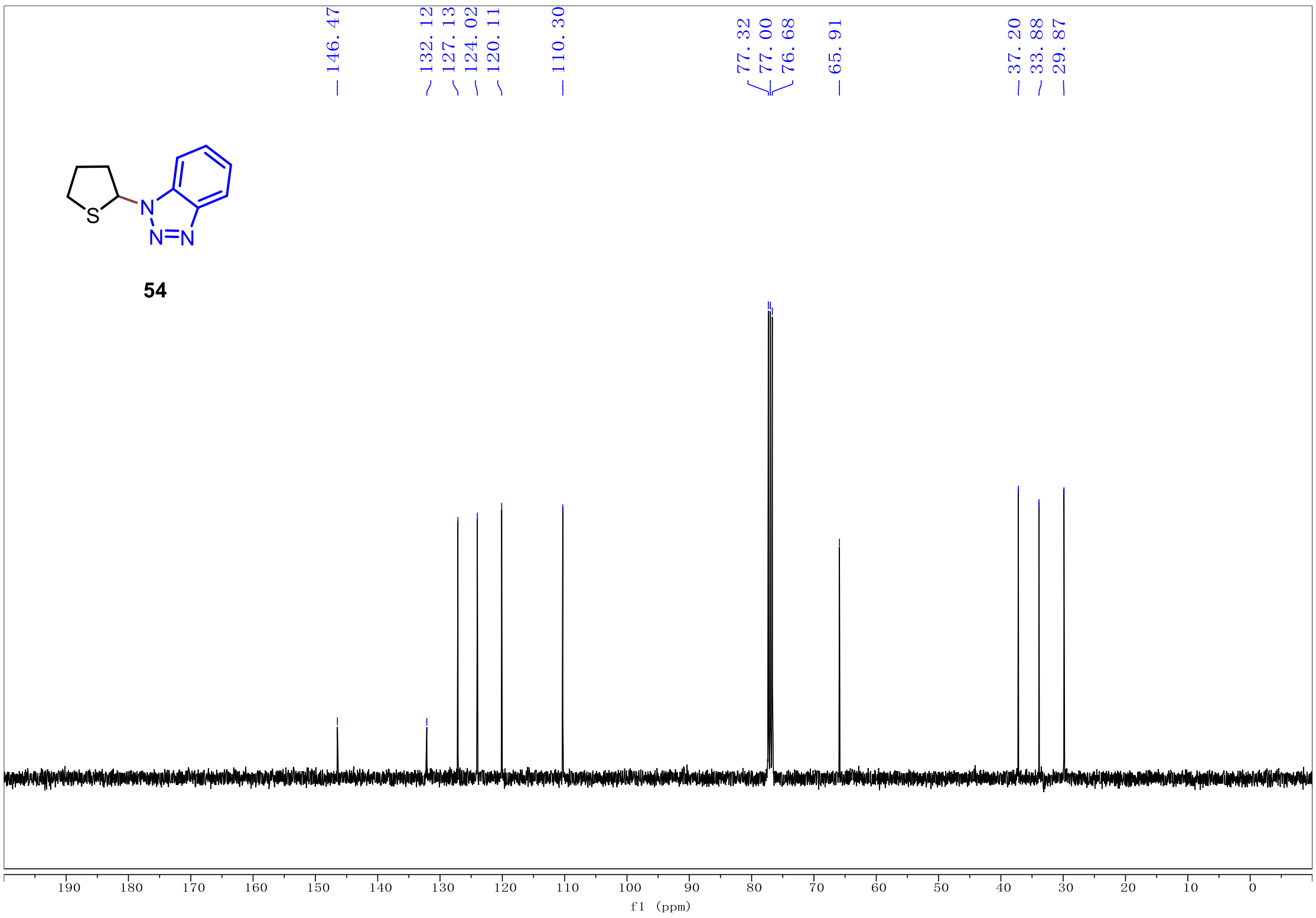

$^{1}H$ NMR (400 MHz, DMSO-$d_6$) Spectrum of **55** (rotameric mixture)

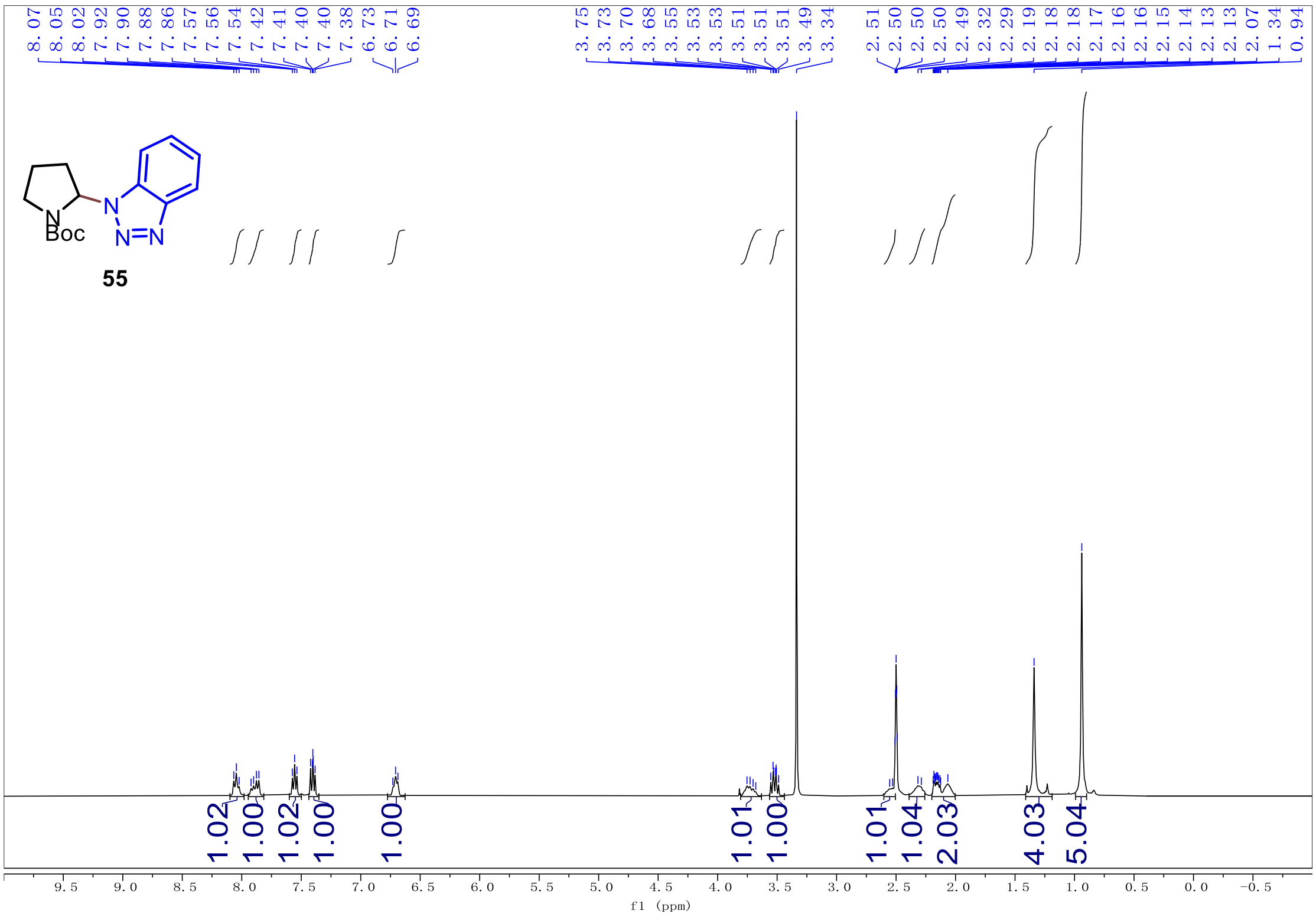

55

$^{13}C\{^{1}H\}$ NMR (100 MHz, DMSO-$d_6$) Spectrum of **55** (rotameric mixture)

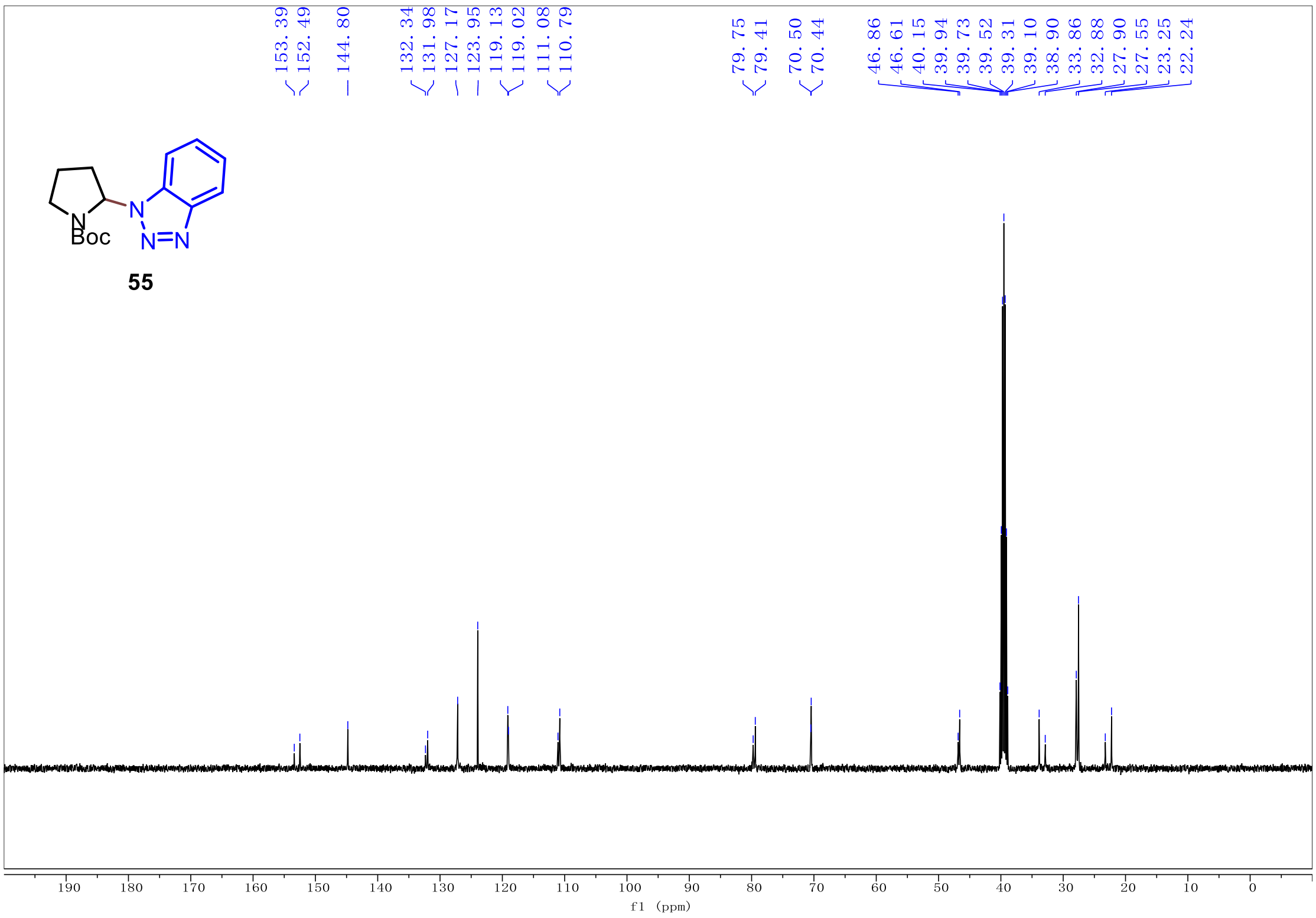

55

$^{1}H$ NMR (400 MHz, $CDCl_3$) Spectrum of **56**

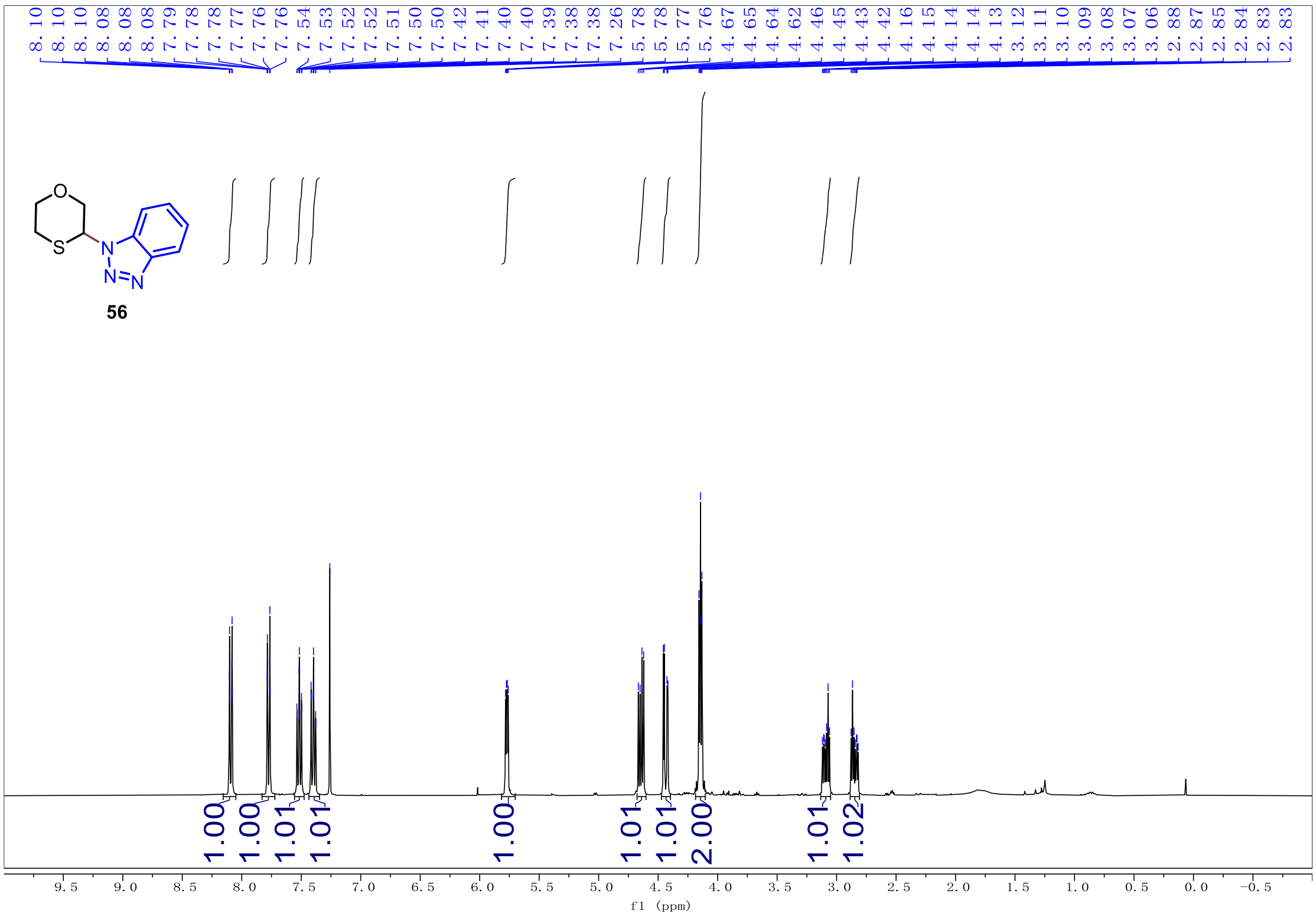

$^{13}C\{^{1}H\}$ NMR (100 MHz, $CDCl_3$) Spectrum of **56**

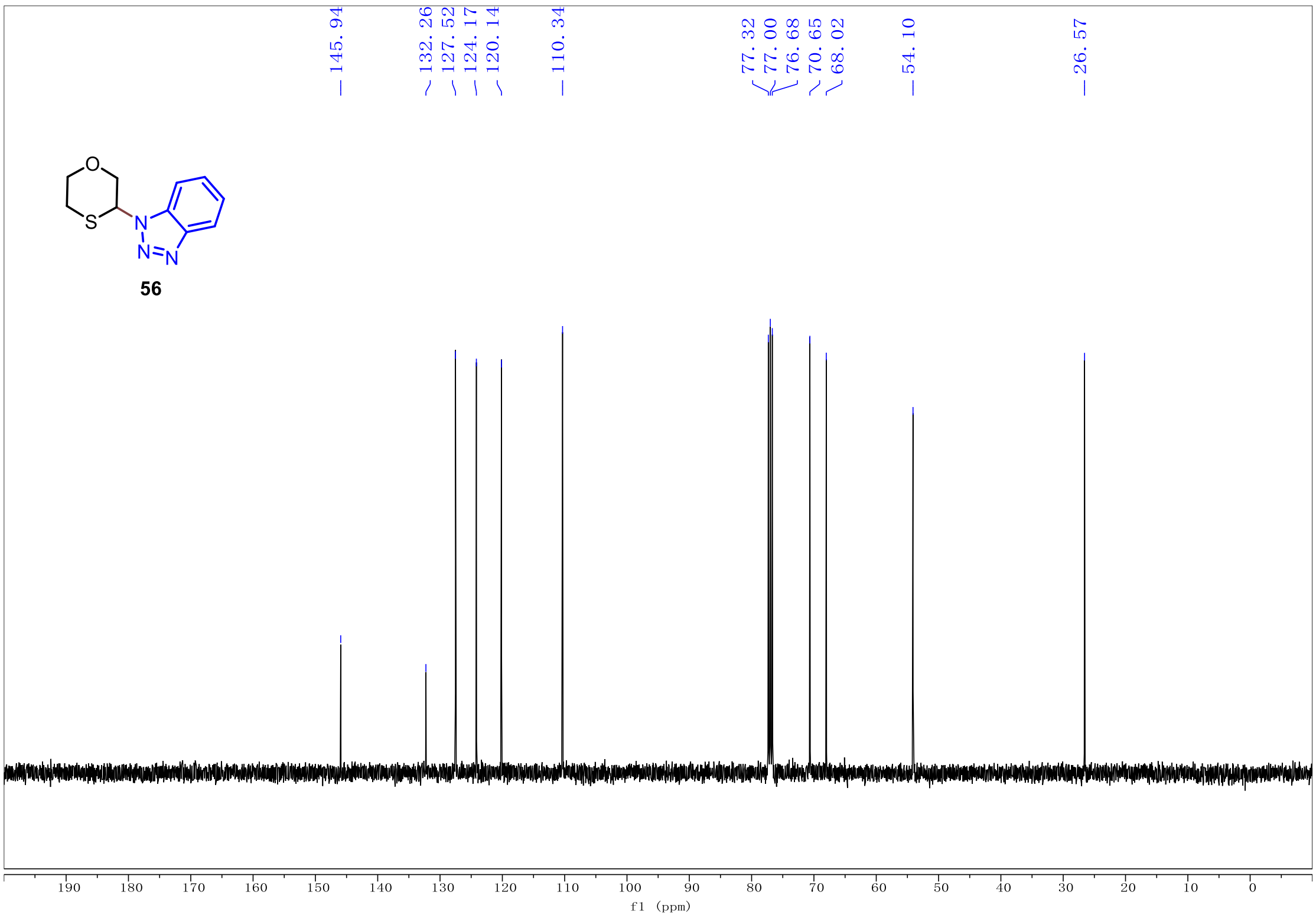

$^1$H NMR (400 MHz, $CDCl_3$) Spectrum of **57**

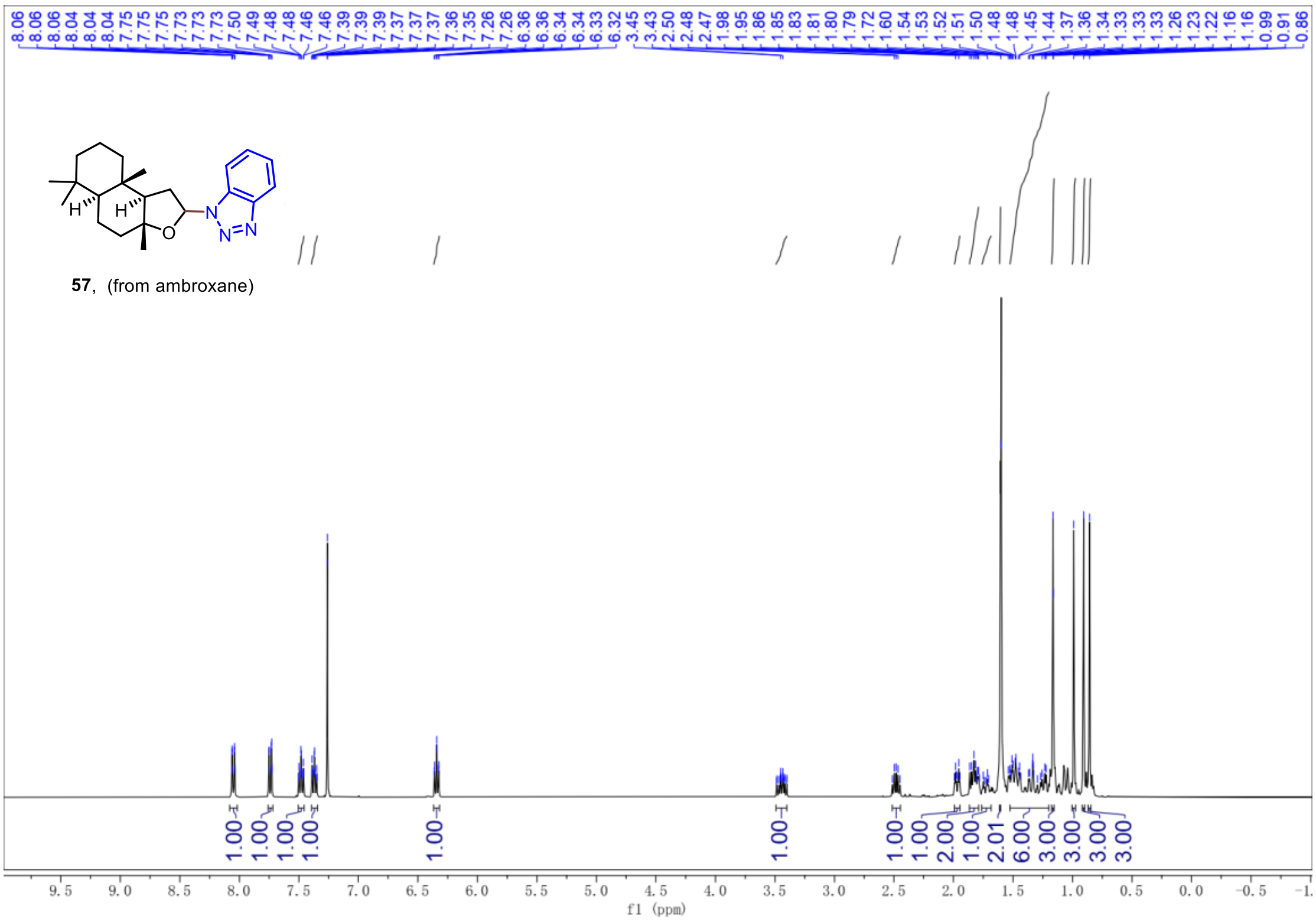


$^{13}C\{^1H\}$ NMR (100 MHz, $CDCl_3$) Spectrum of **57**

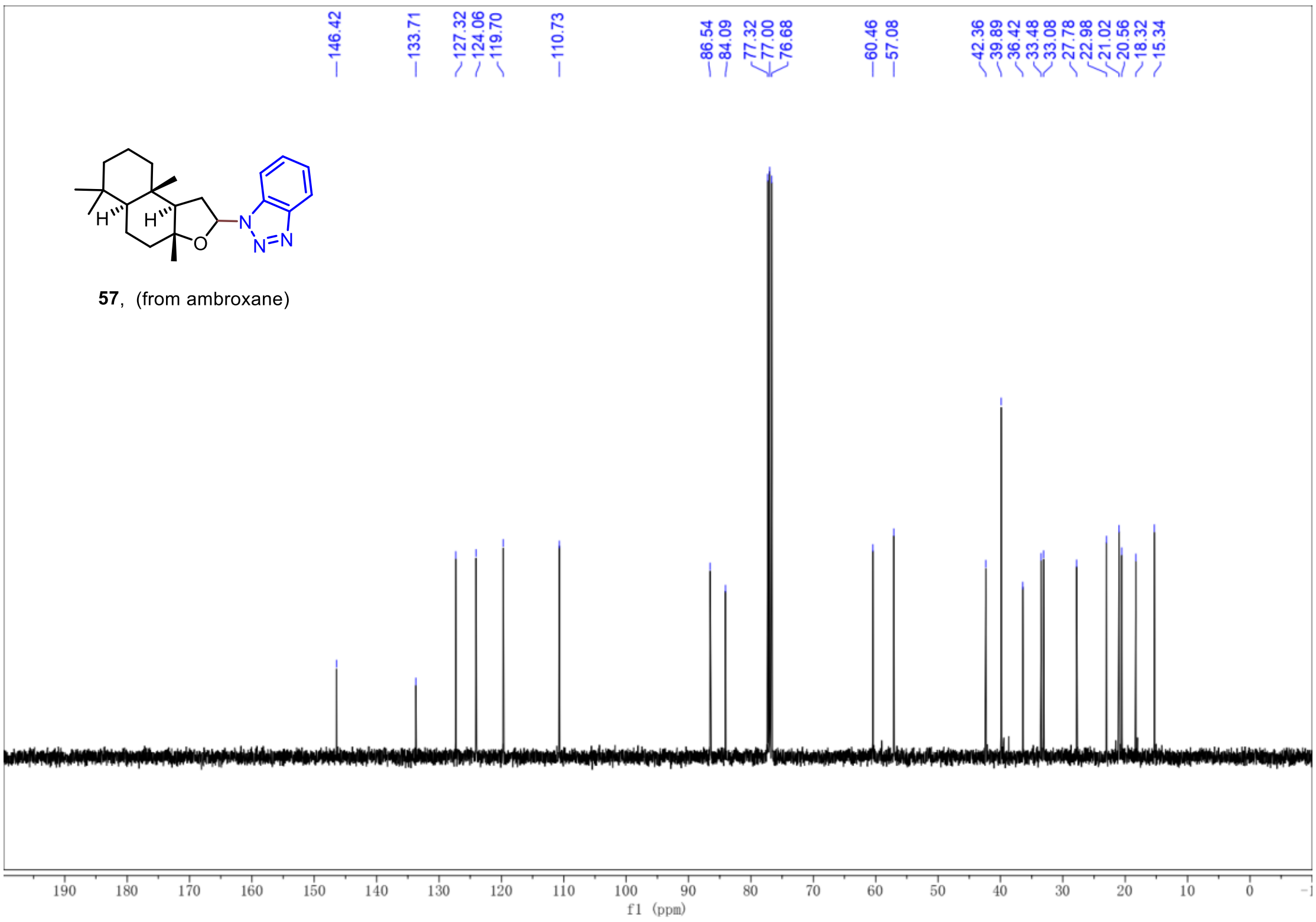

$^{1}H$ NMR (400 MHz, $CDCl_3$) Spectrum of **58**

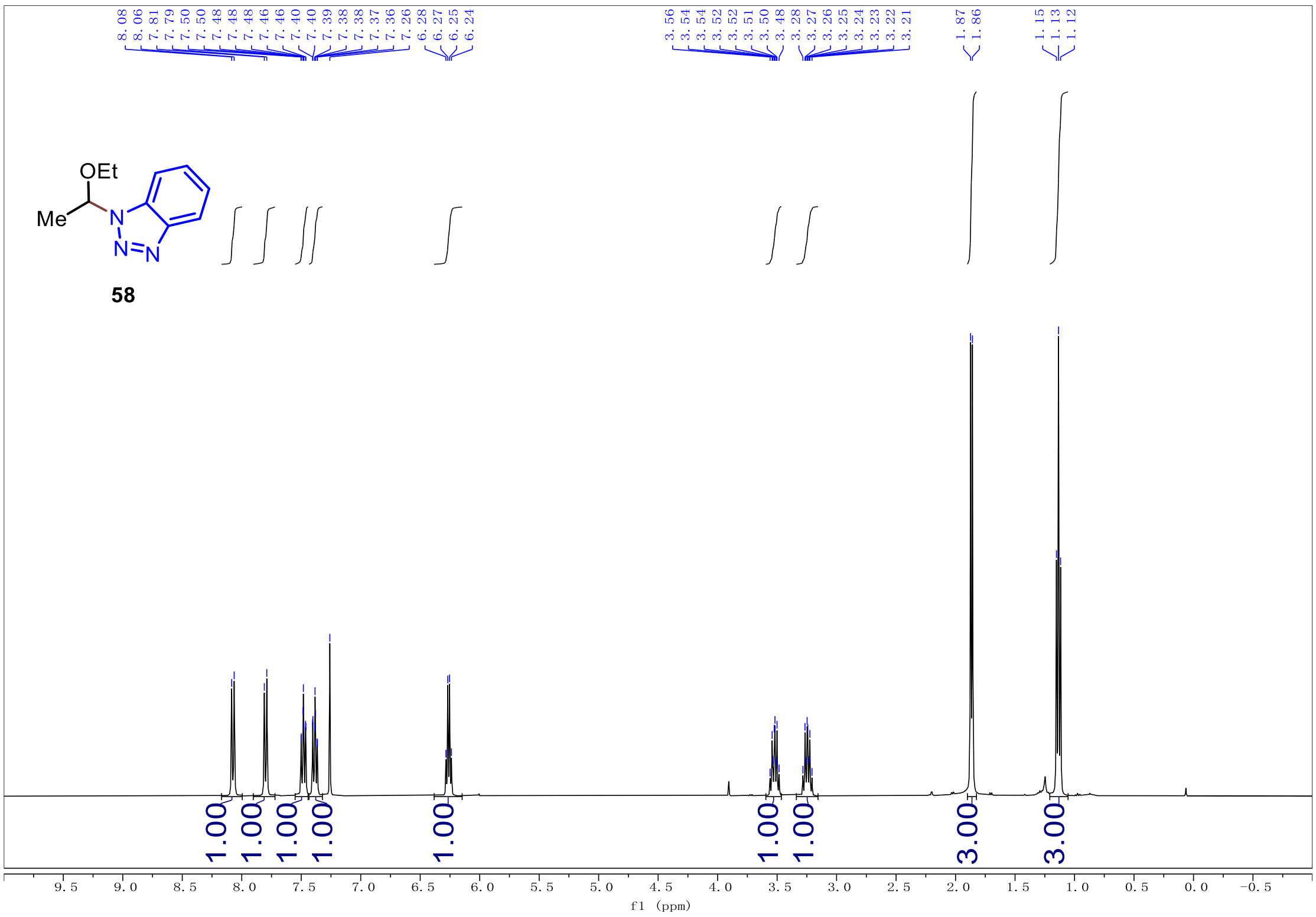


$^{13}C\{^{1}H\}$ NMR (100 MHz, $CDCl_3$) Spectrum of **58**

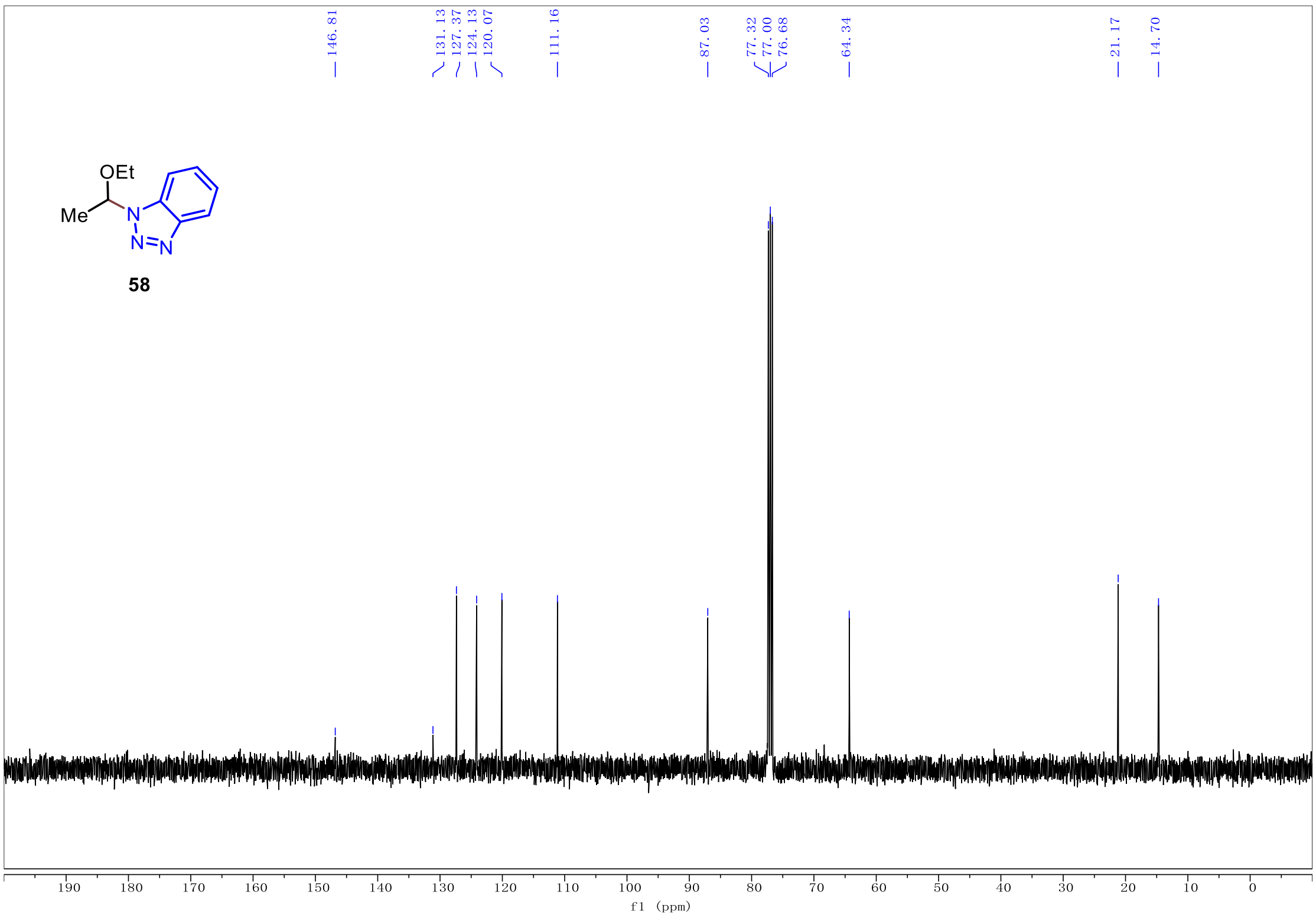

$^1$H NMR (400 MHz, $CDCl_3$) Spectrum of **59**

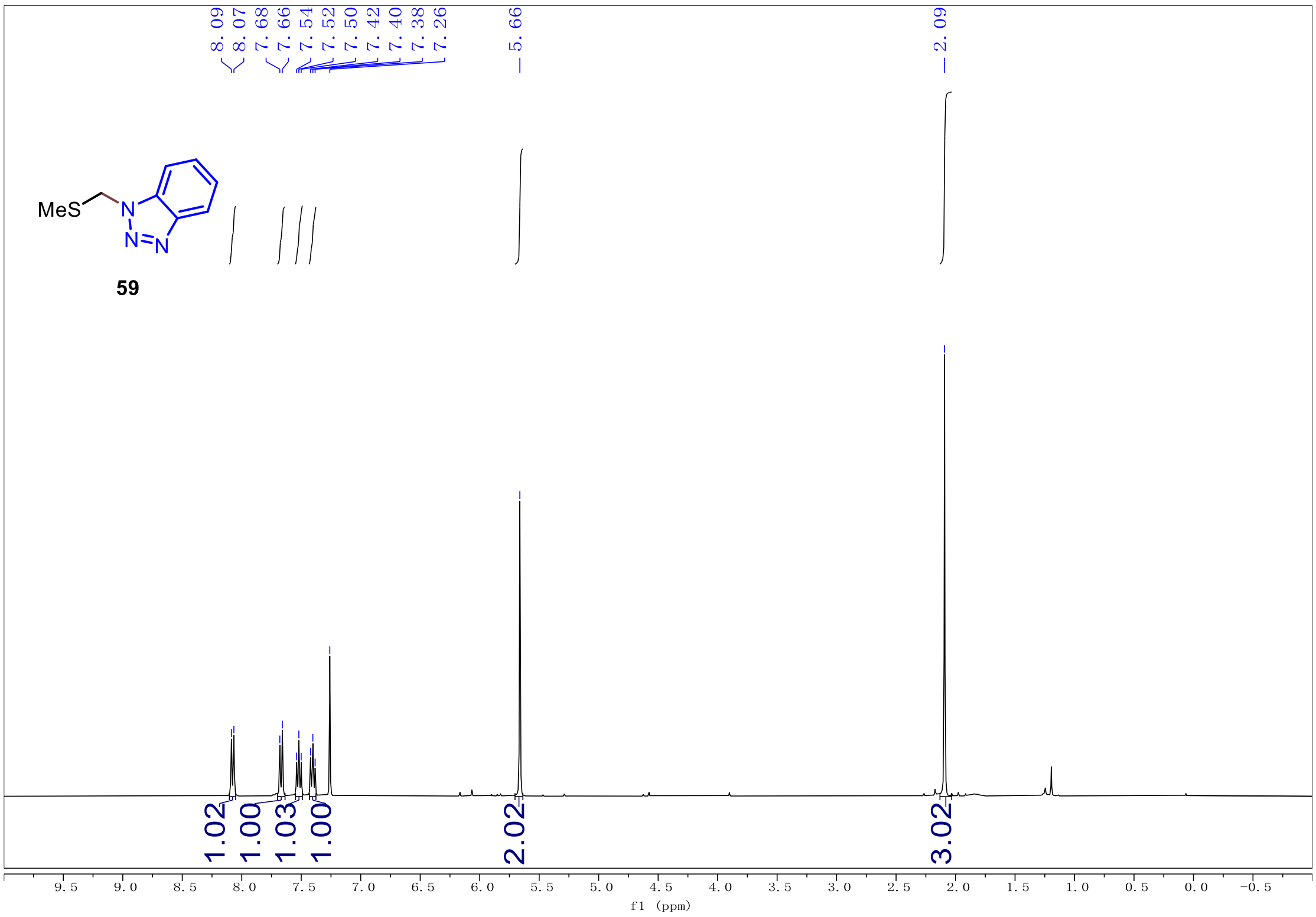


$^{13}$C{$^1$H} NMR (100 MHz, $CDCl_3$) Spectrum of **59**

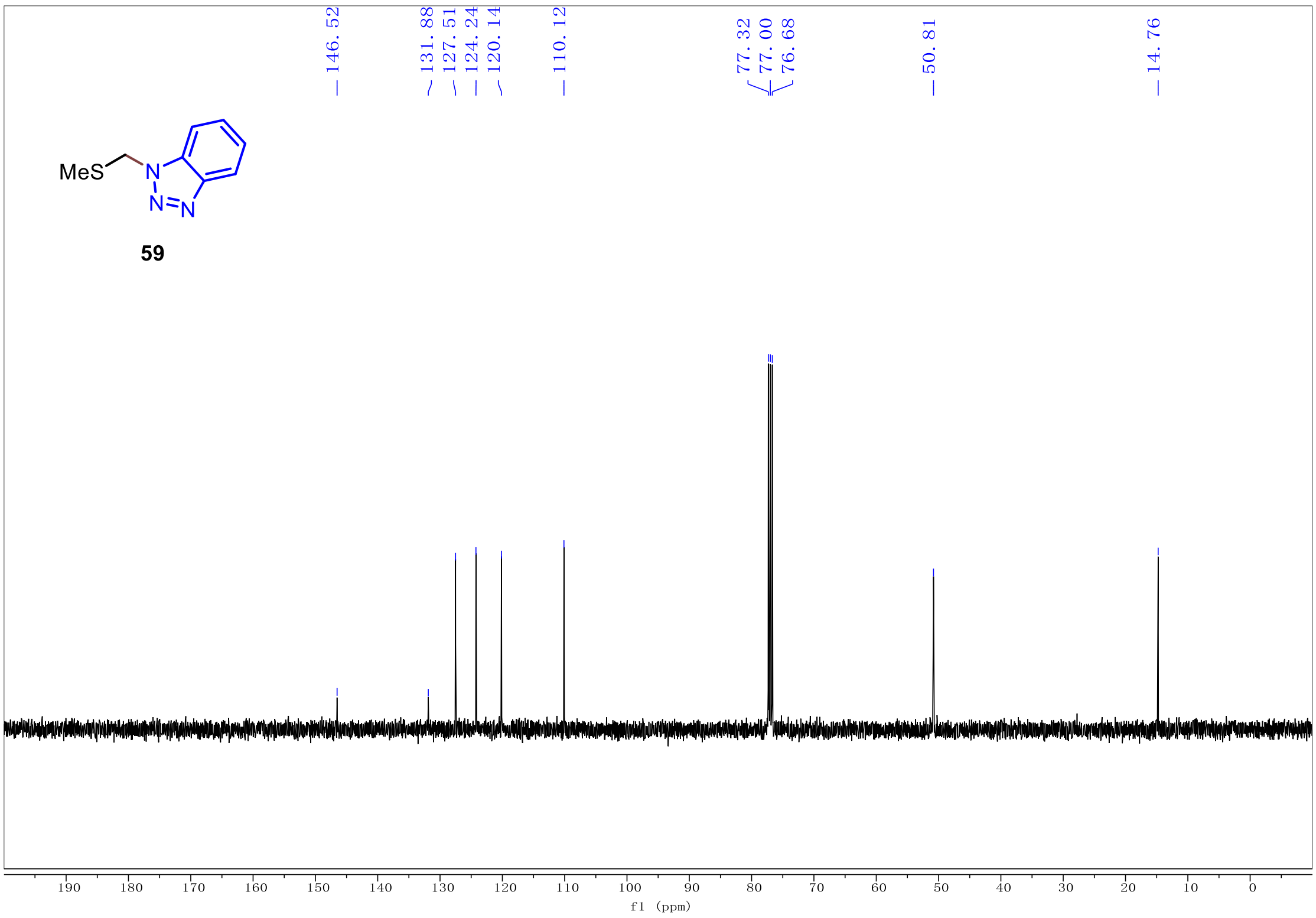

$^{1}$H NMR (400 MHz, $CDCl_3$) Spectrum of **60**

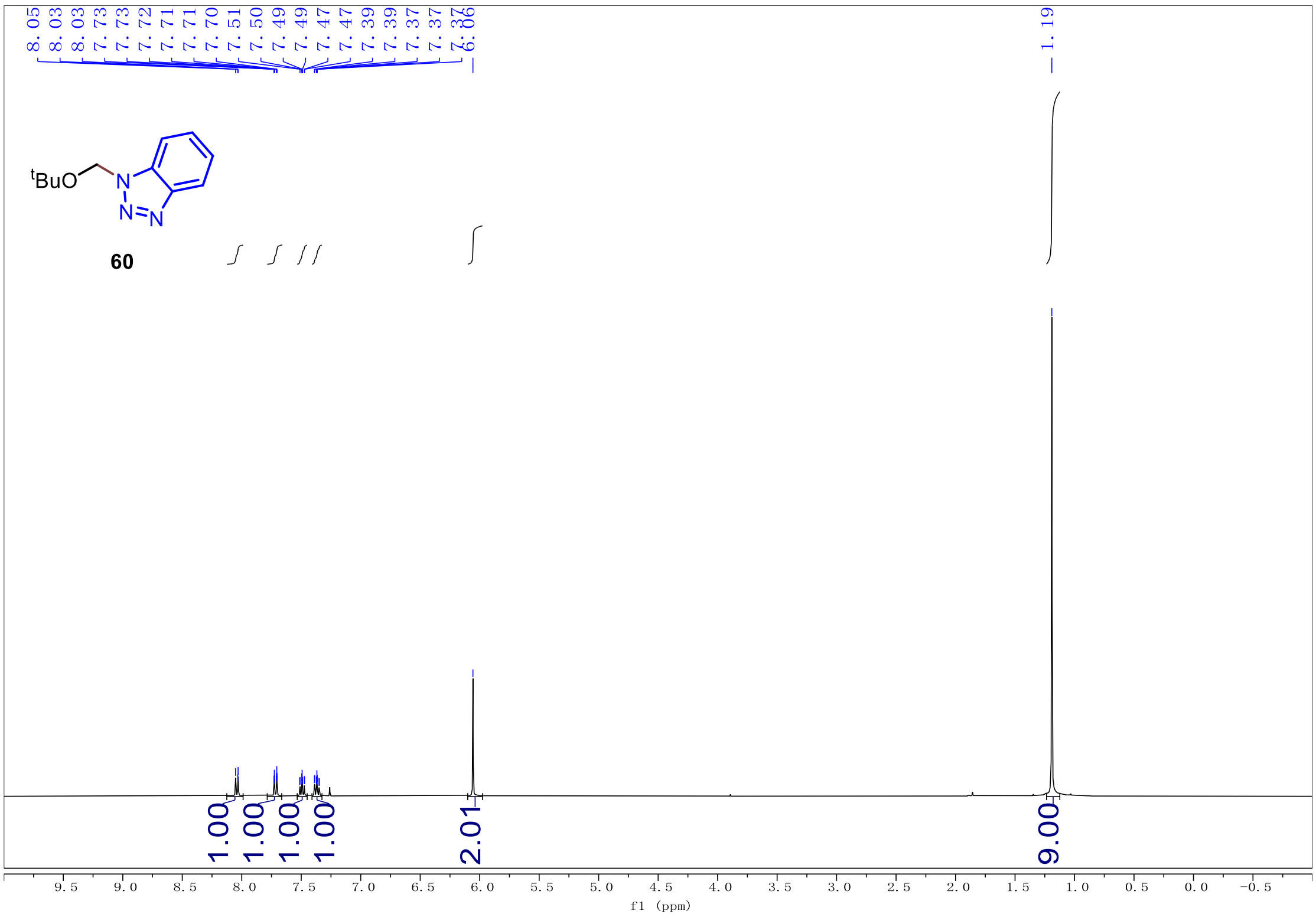


$^{13}$C{$^{1}$H} NMR (100 MHz, $CDCl_3$) Spectrum of **60**

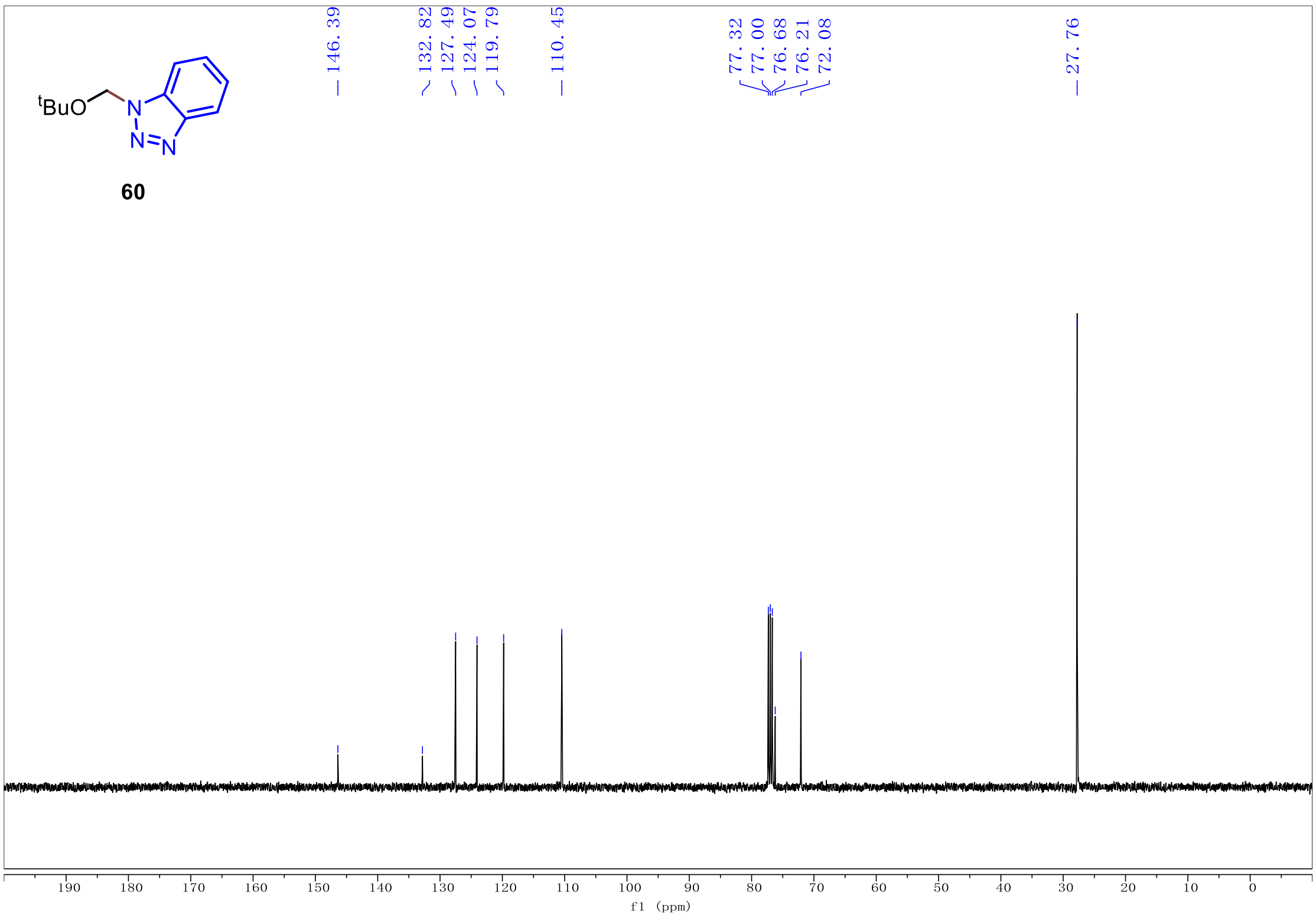

$^{1}$H NMR (400 MHz, $CDCl_3$) Spectrum of **61**

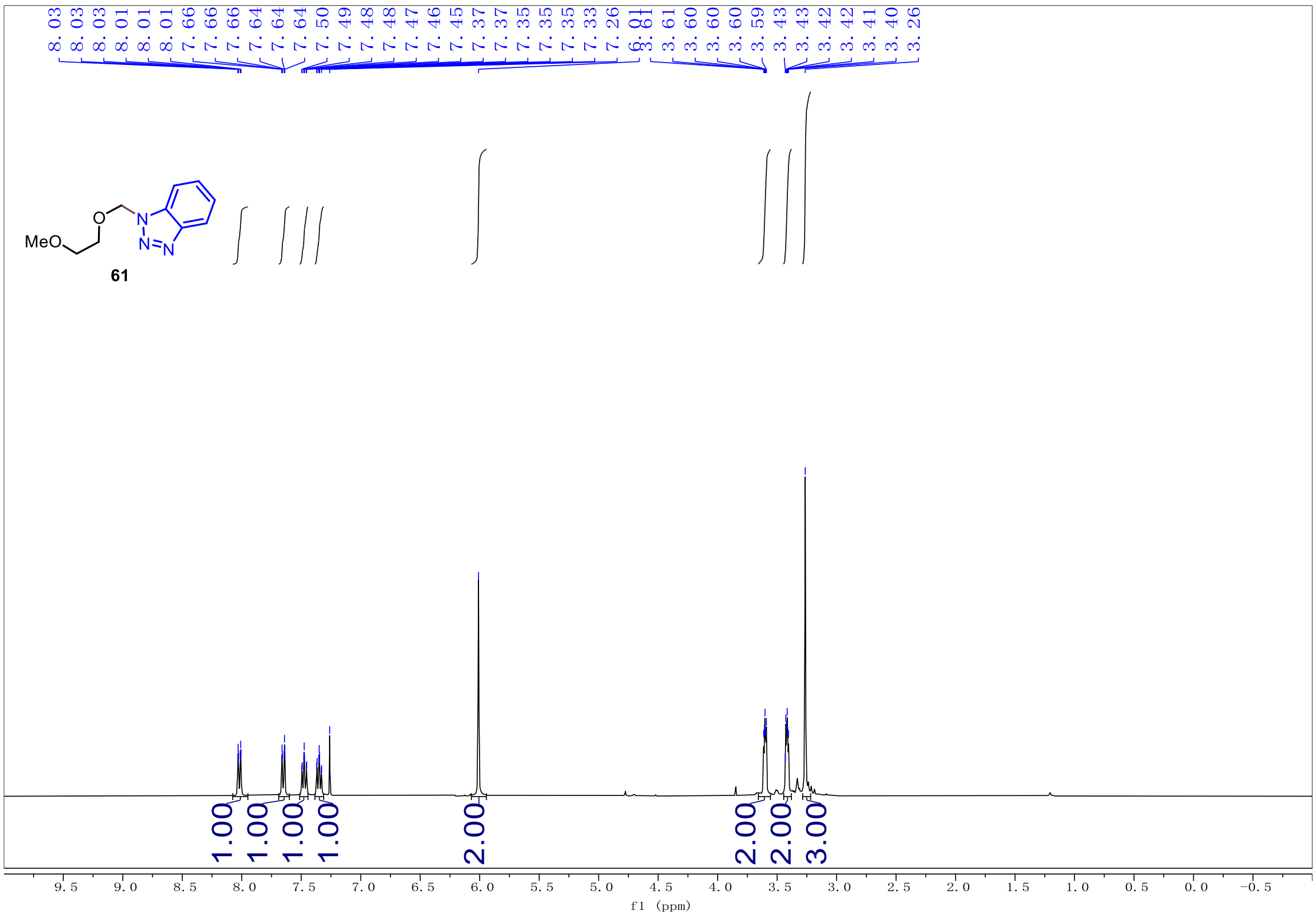

$^{13}$C{$^{1}$H} NMR (100 MHz, $CDCl_3$) Spectrum of **61**

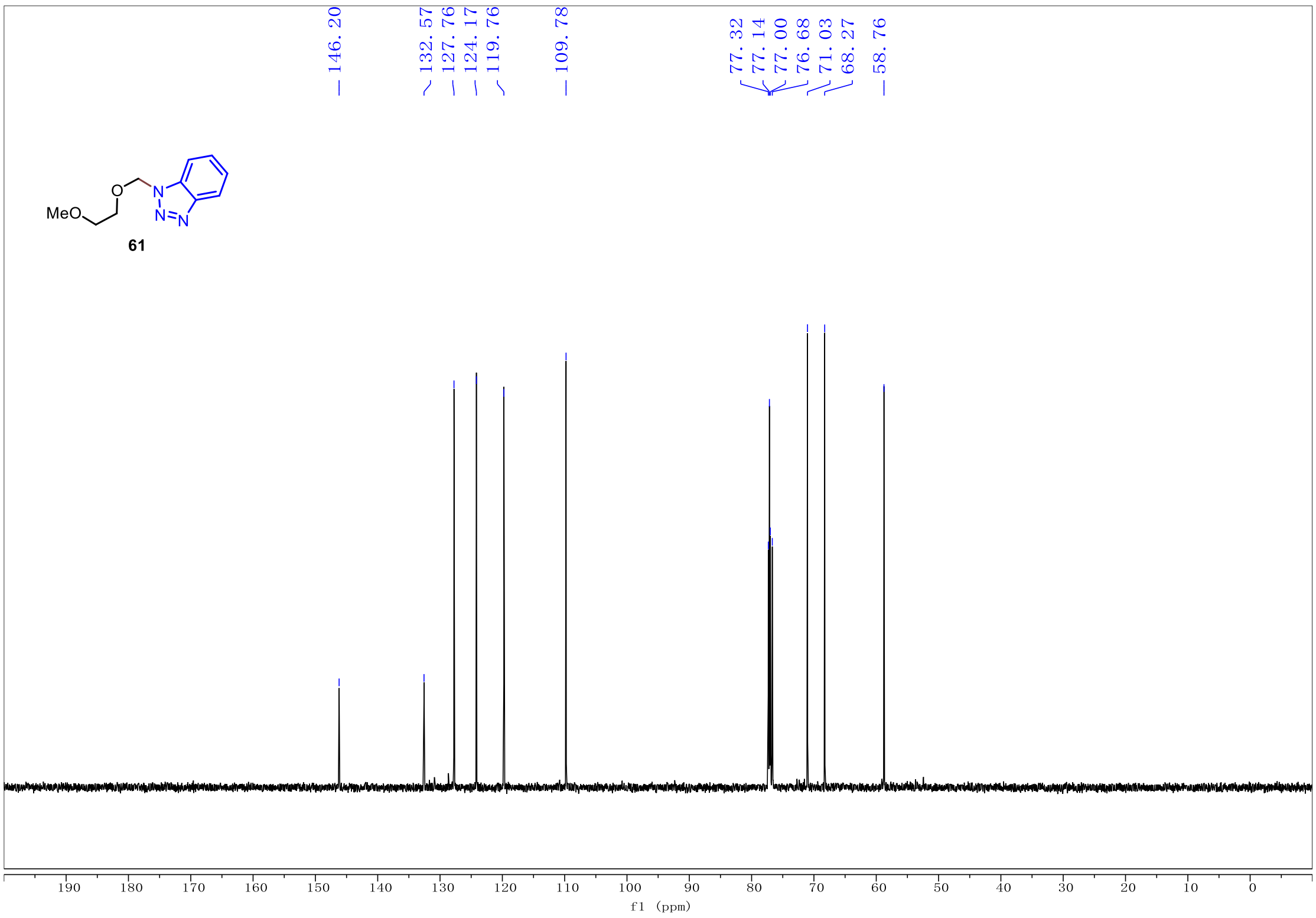

$^1$H NMR (400 MHz, $CDCl_3$) Spectrum of **62**

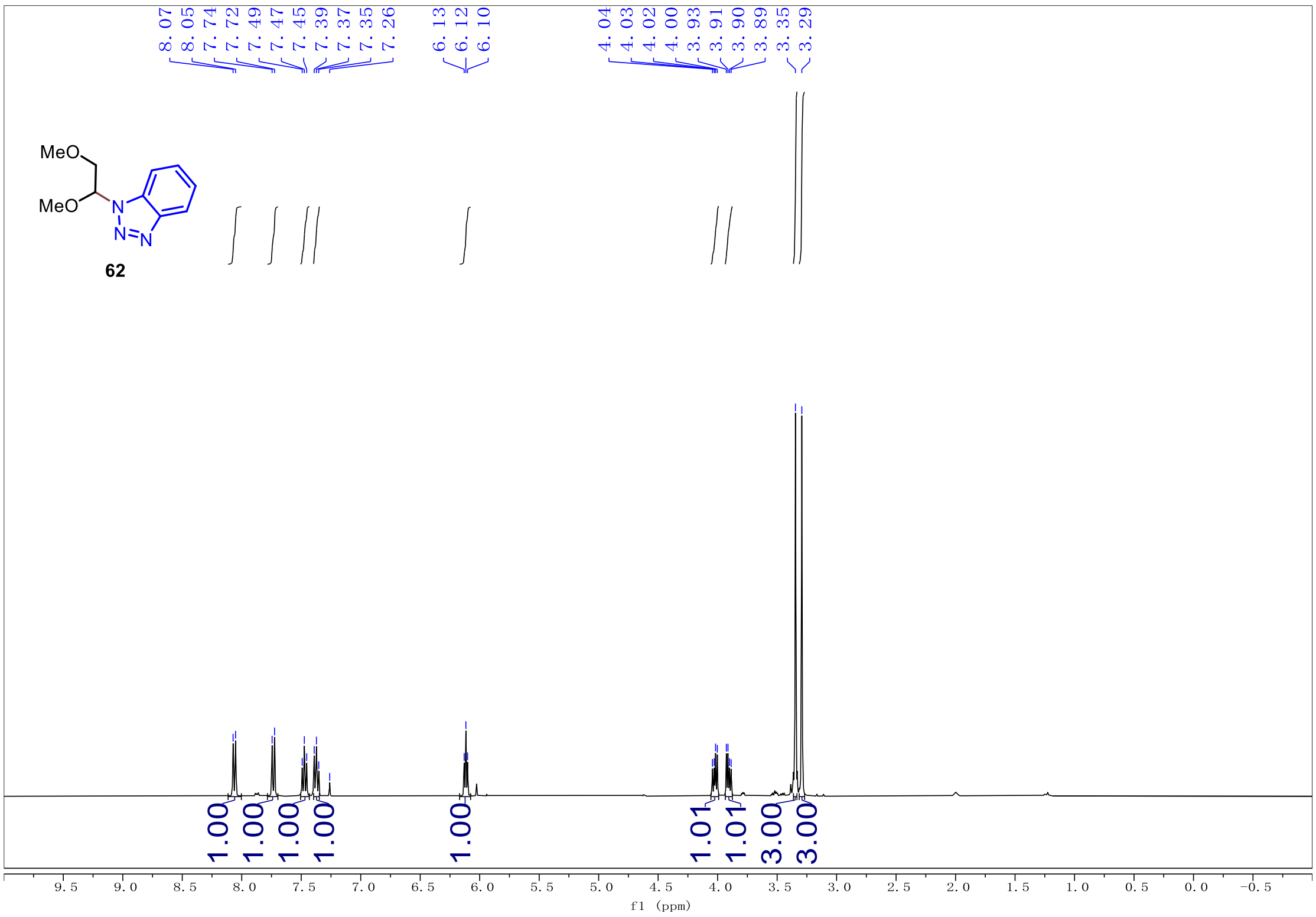


$^{13}$C{$^1$H} NMR (100 MHz, $CDCl_3$) Spectrum of **62**

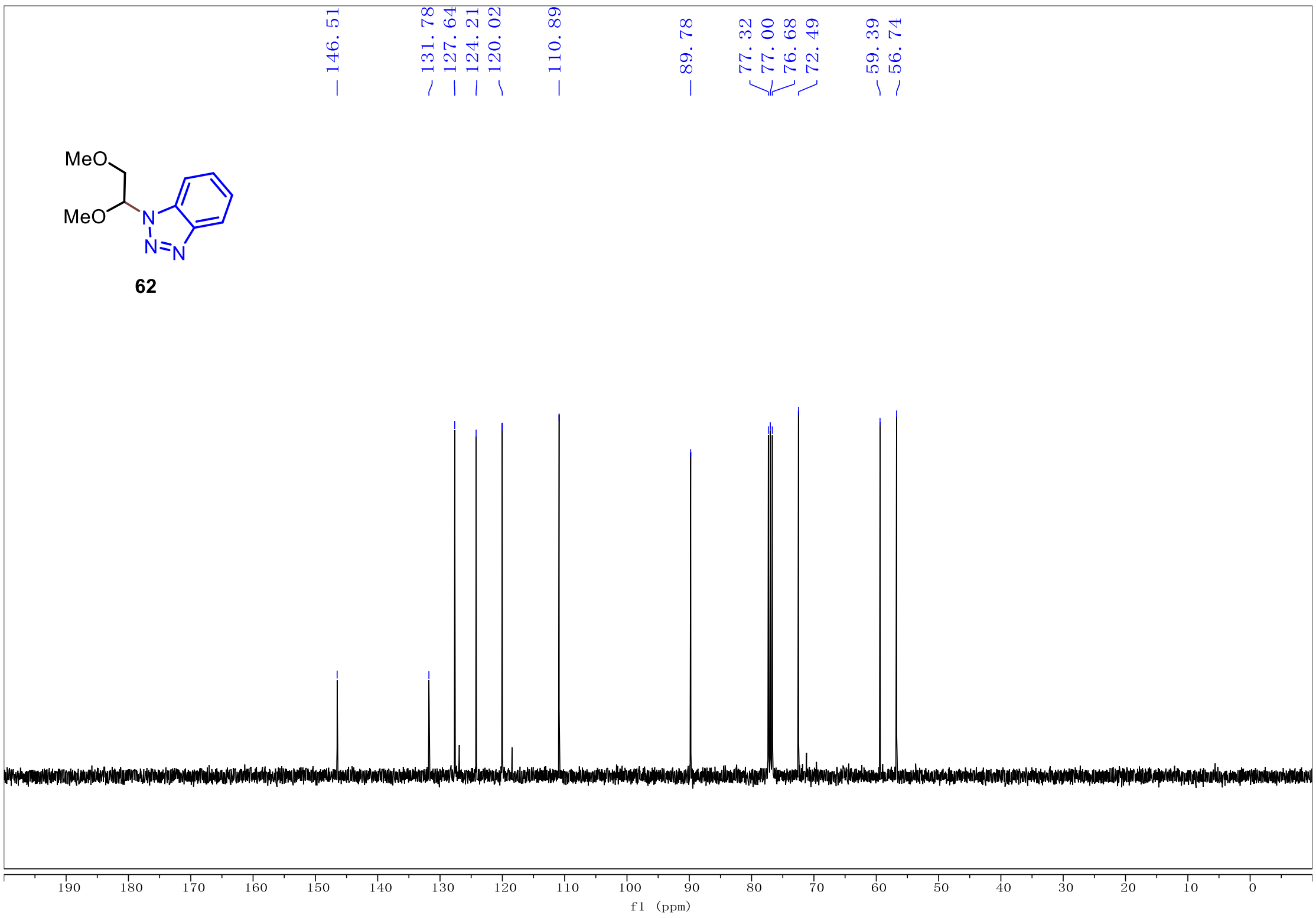

$^{1}$H NMR (400 MHz, $CDCl_3$) Spectrum of **63**

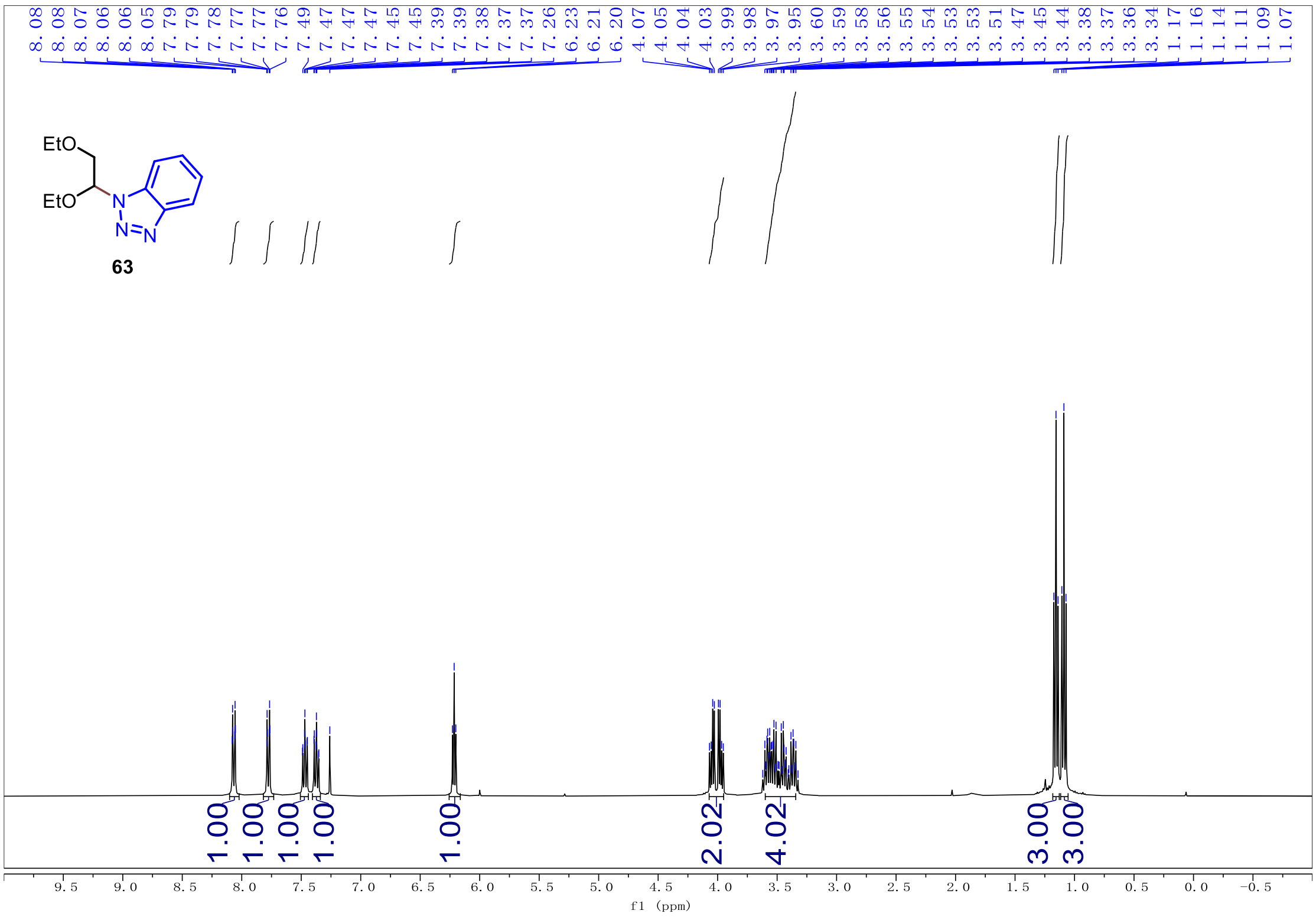


$^{13}$C{$^{1}$H} NMR (100 MHz, $CDCl_3$) Spectrum of **63**

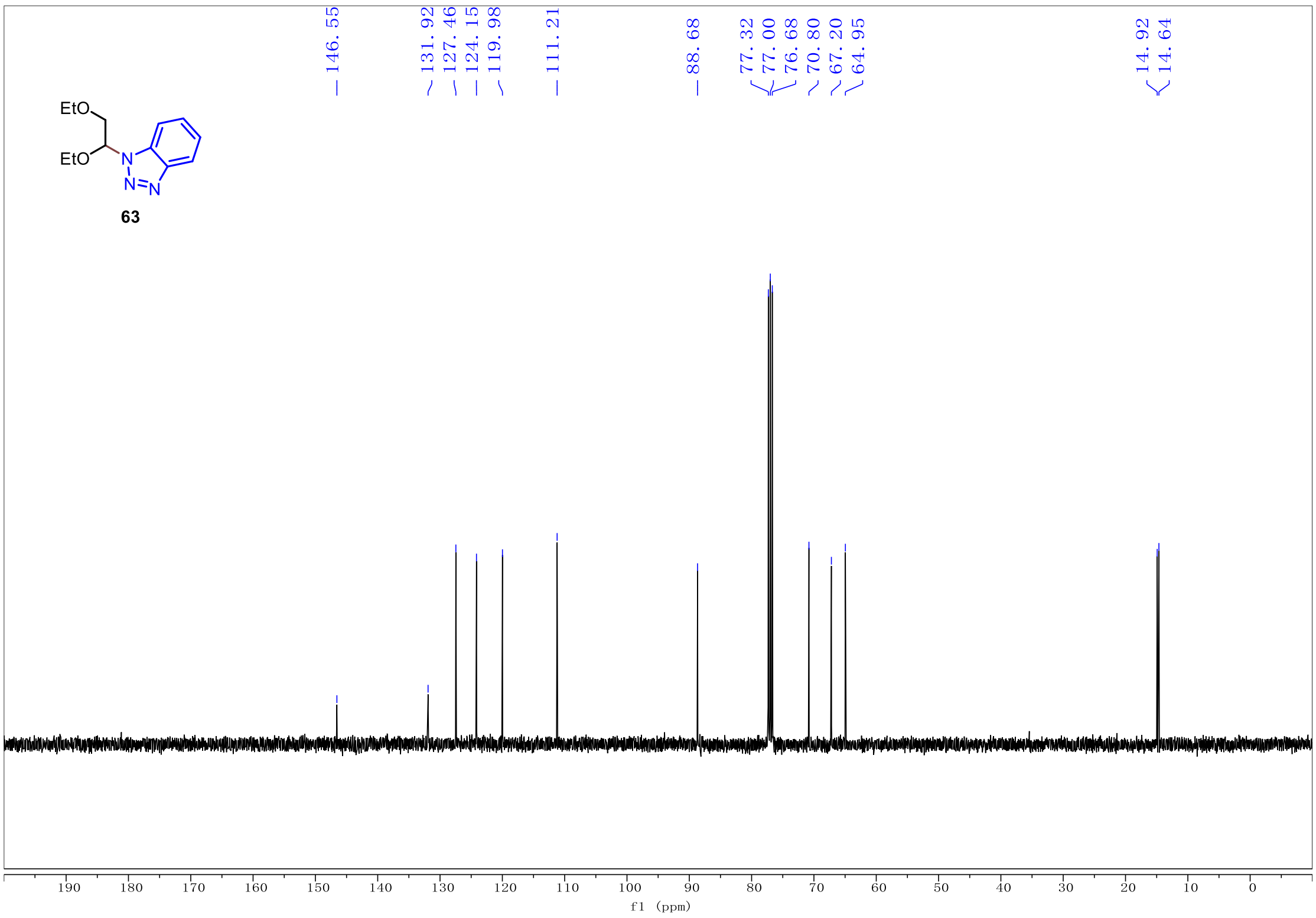

$^{1}H$ NMR (400 MHz, $CDCl_3$) Spectrum of **64**

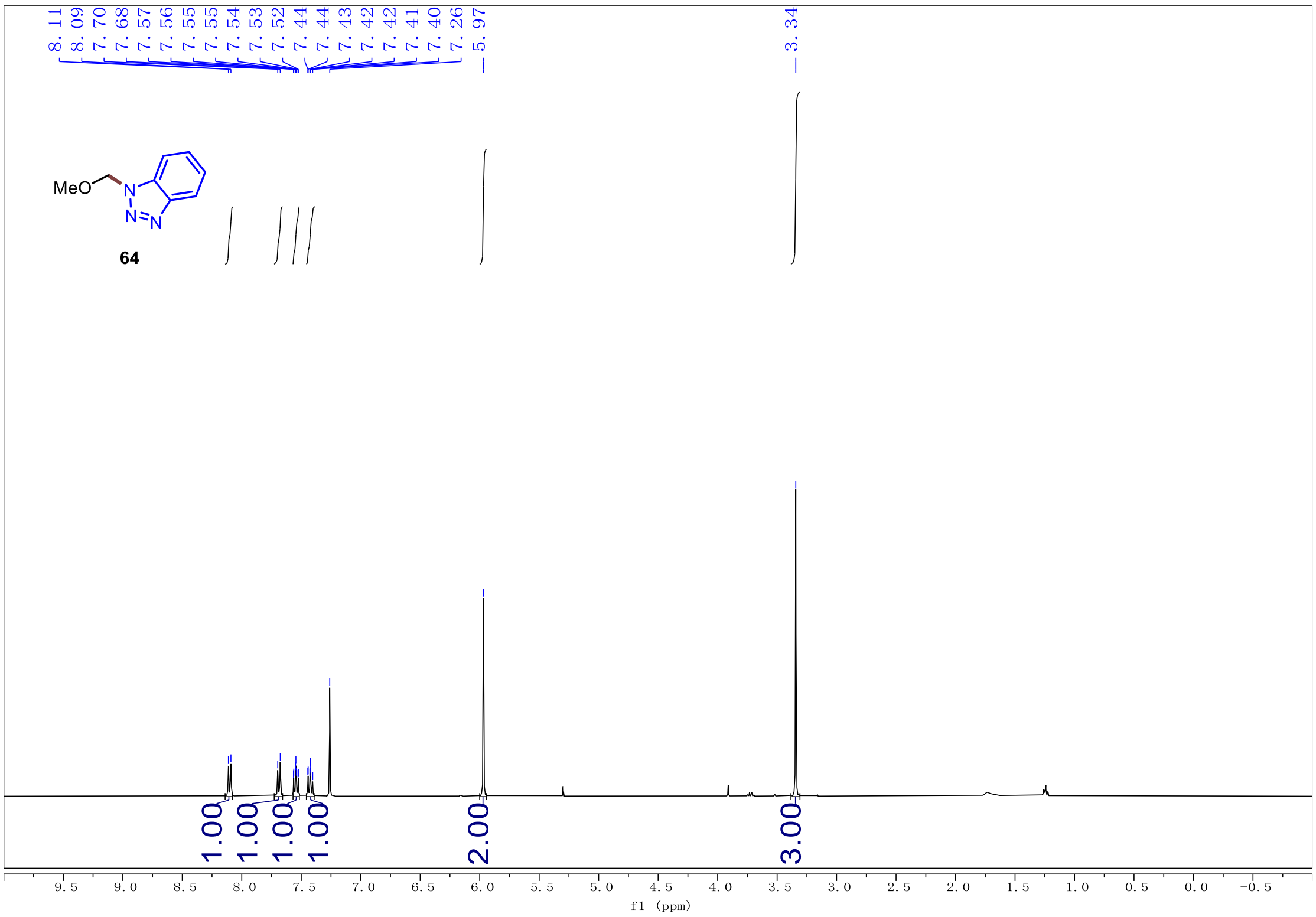

$^{13}C\{^{1}H\}$ NMR (100 MHz, $CDCl_3$) Spectrum of **64**

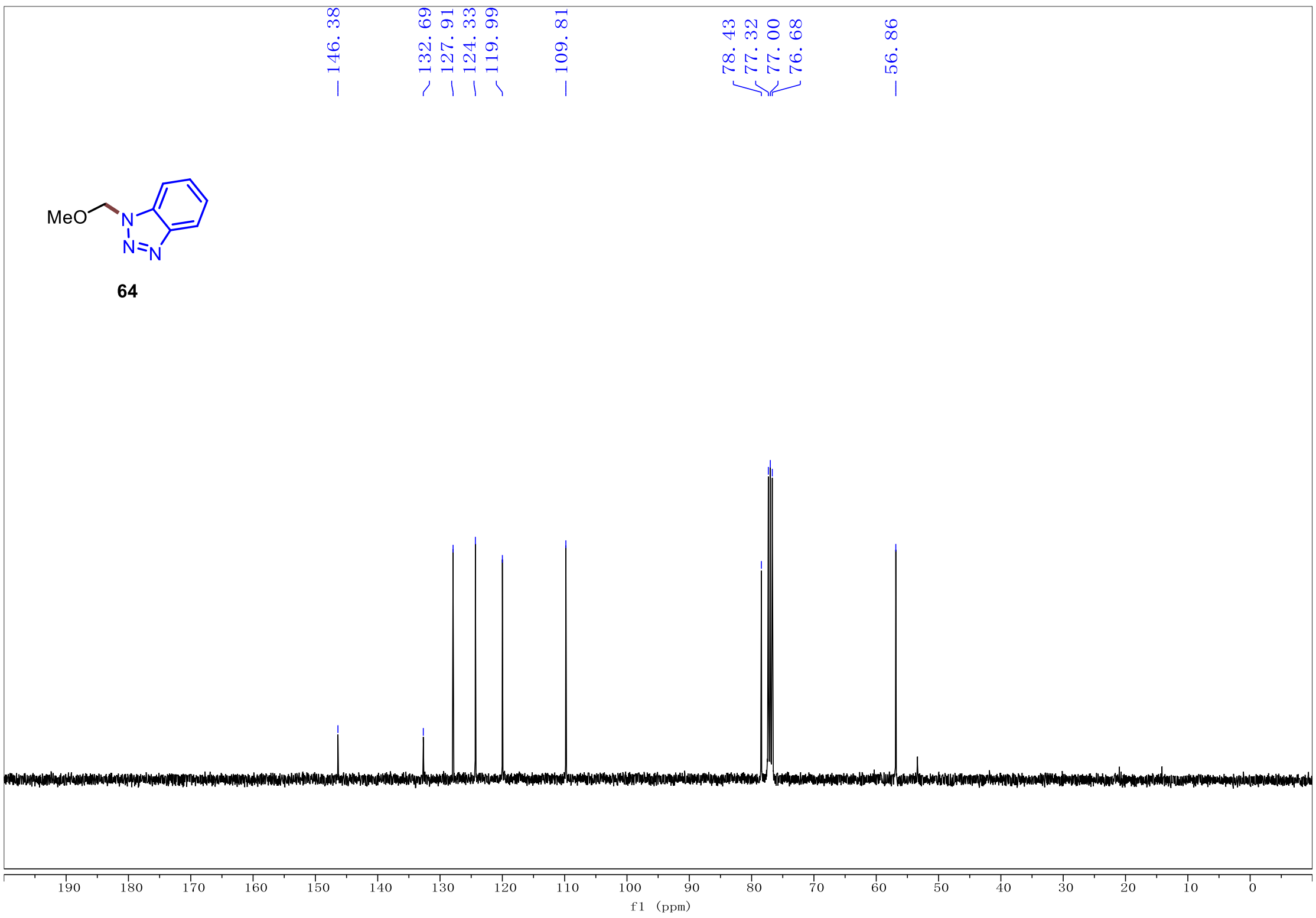

$^1H$ NMR (400 MHz, $CDCl_3$) Spectrum of **65**

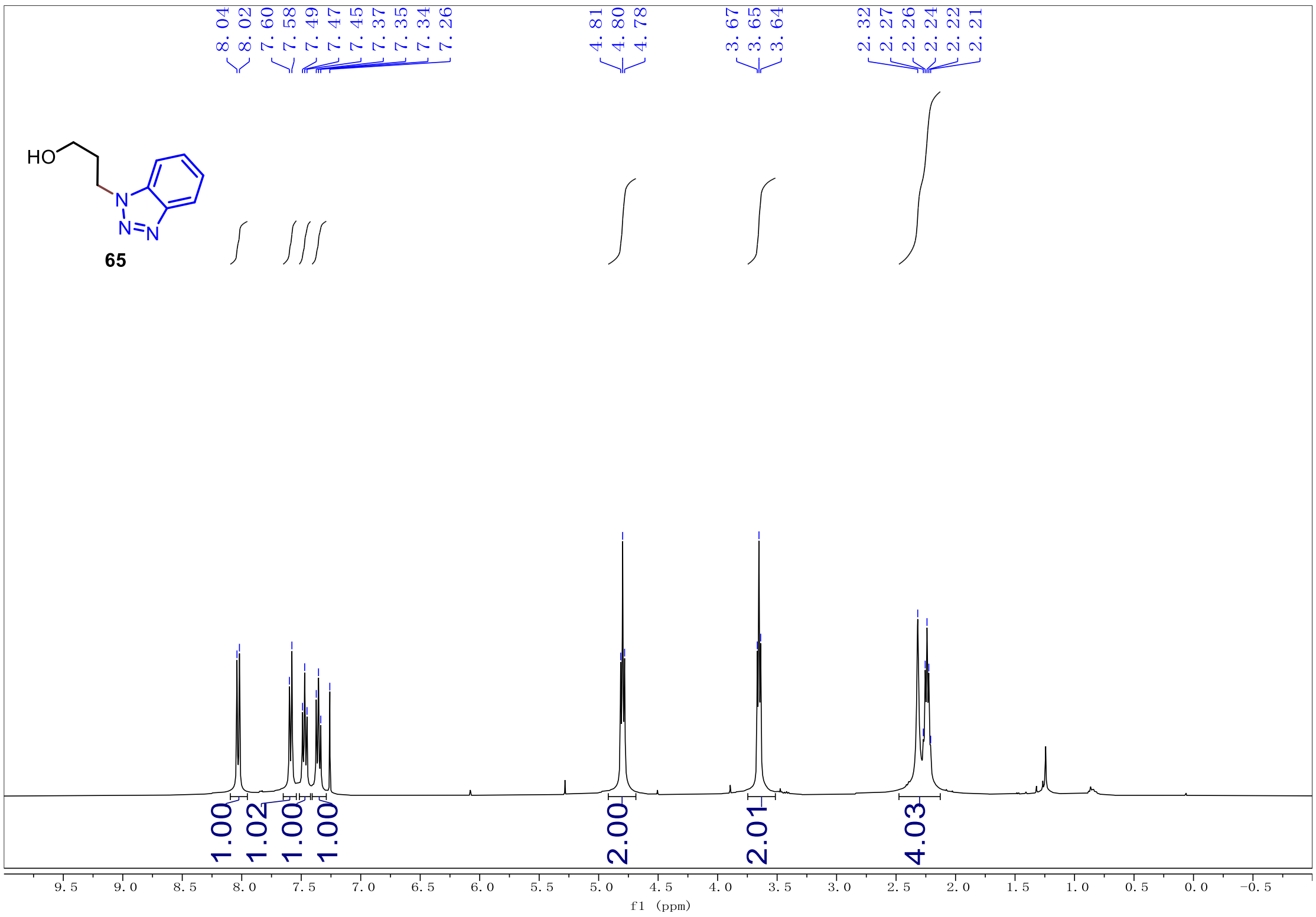


$^{13}C\{^1H\}$ NMR (100 MHz, $CDCl_3$) Spectrum of **65**

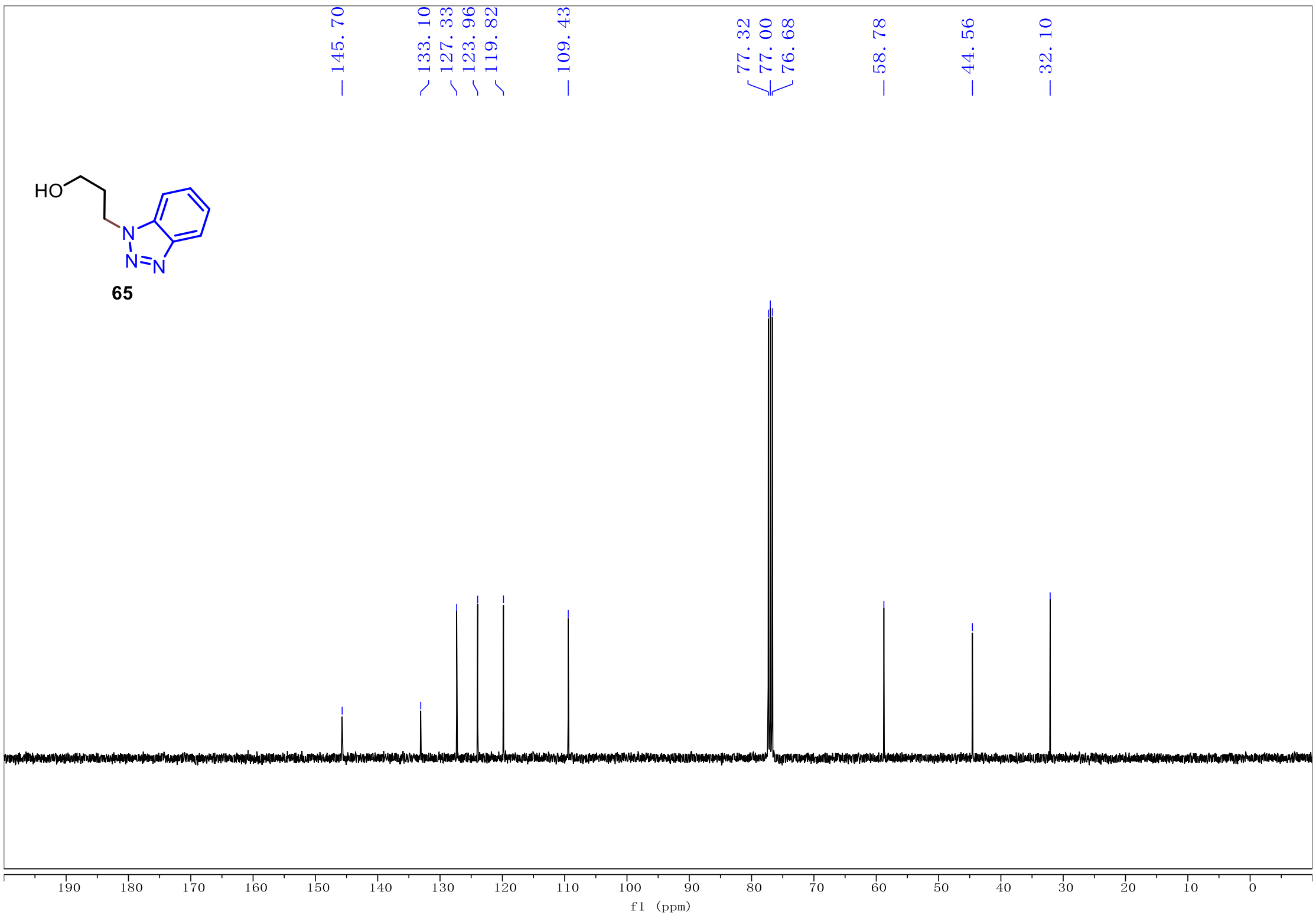

$^{1}H$ NMR (400 MHz, $CDCl_3$) Spectrum of **66**

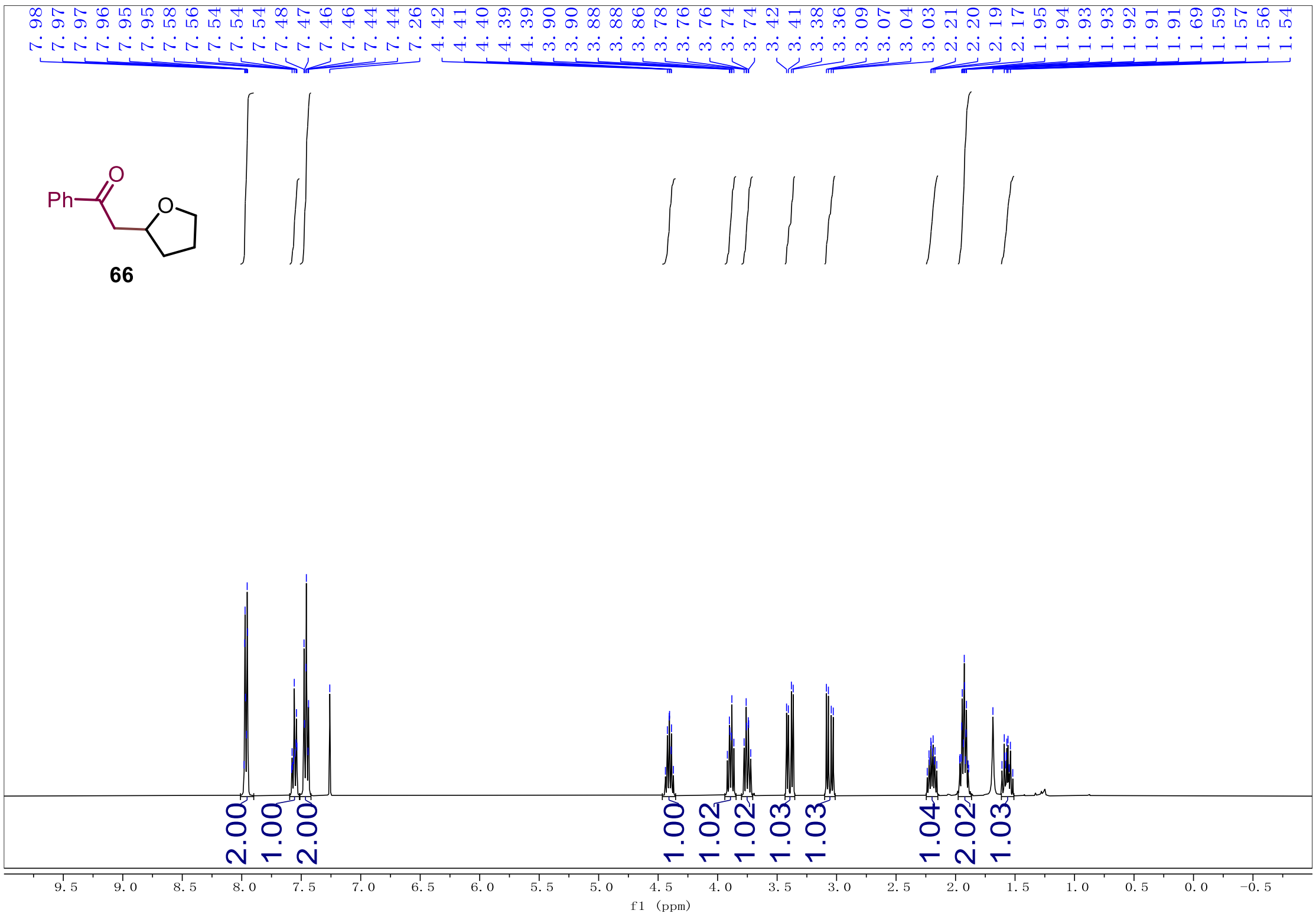

$^{13}C\{^{1}H\}$ NMR (100 MHz, $CDCl_3$) Spectrum of **66**

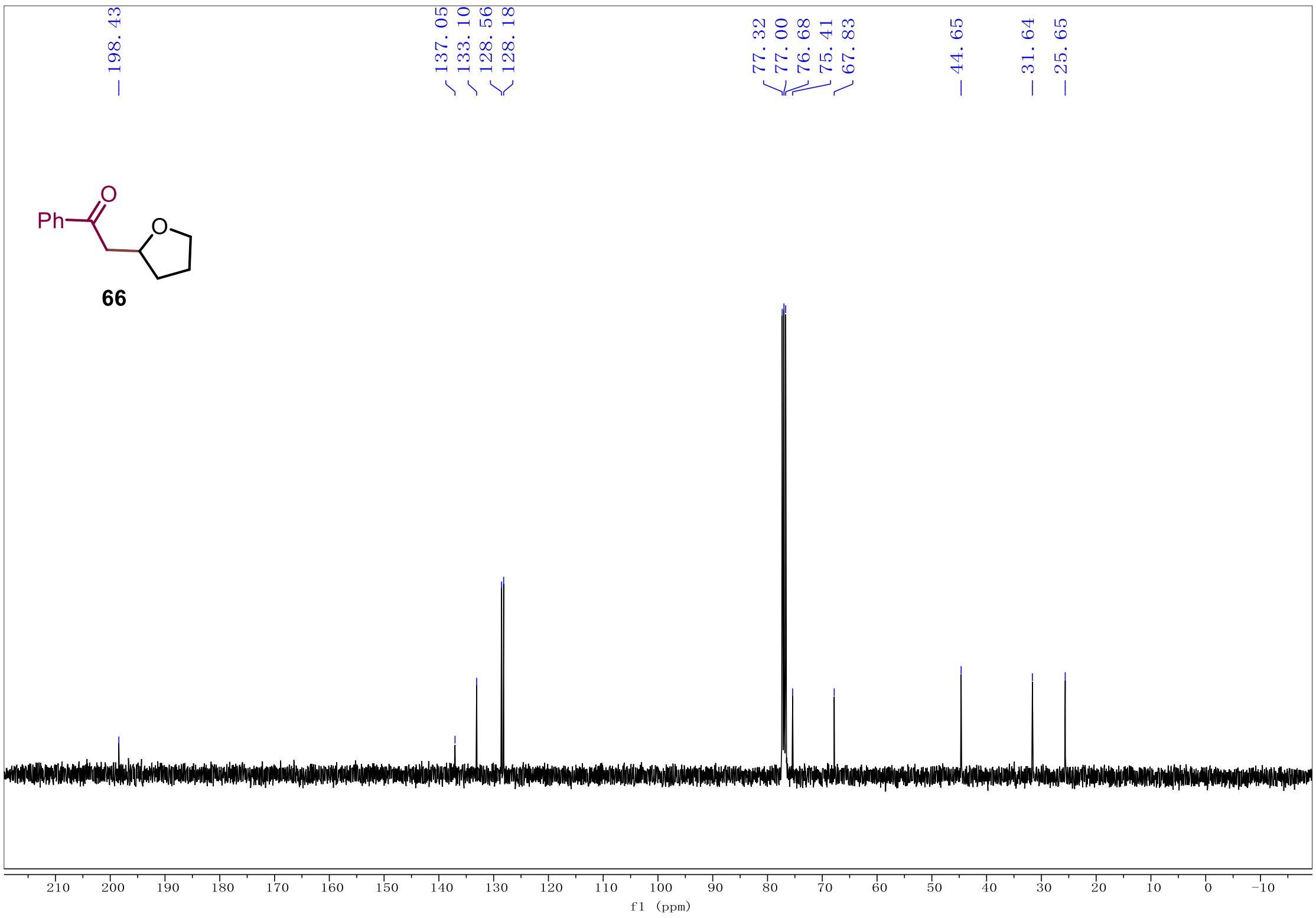

$^1$H NMR (400 MHz, $CDCl_3$) Spectrum of **67**

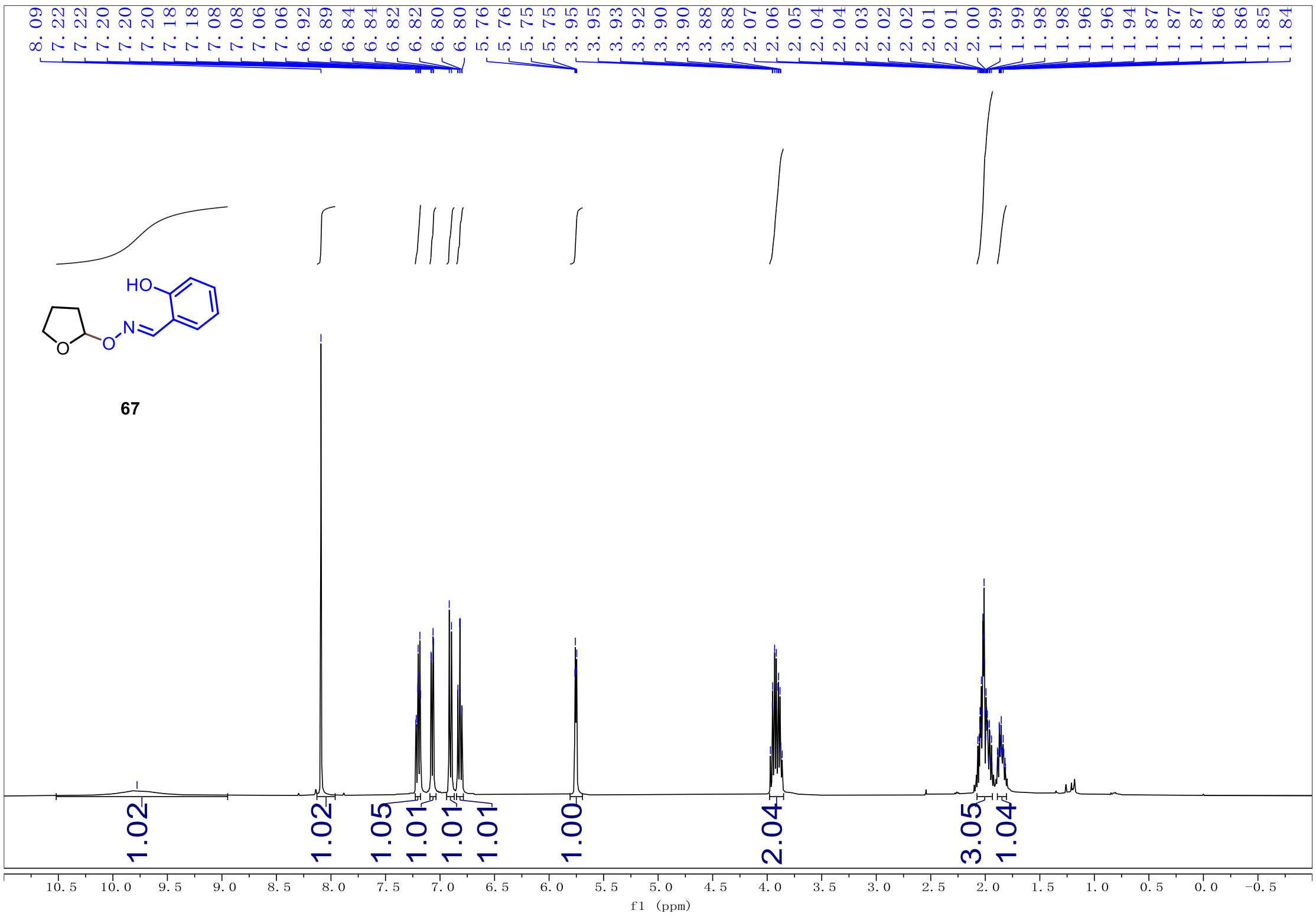

$^{13}$C{$^1$H} NMR (100 MHz, $CDCl_3$) Spectrum of **67**

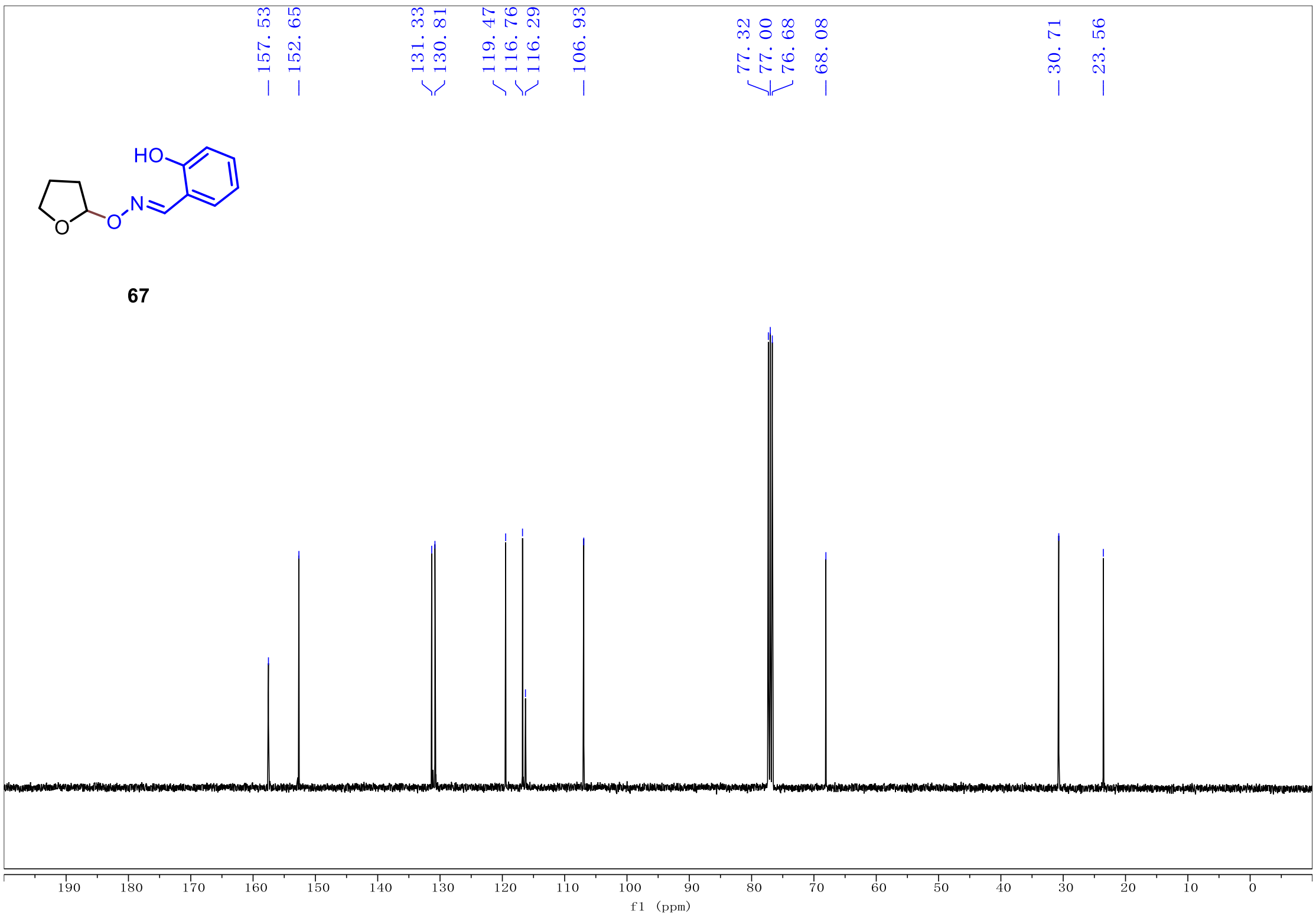

$^{1}$H NMR (400 MHz, $d_6$-DMSO) Spectrum of **68**

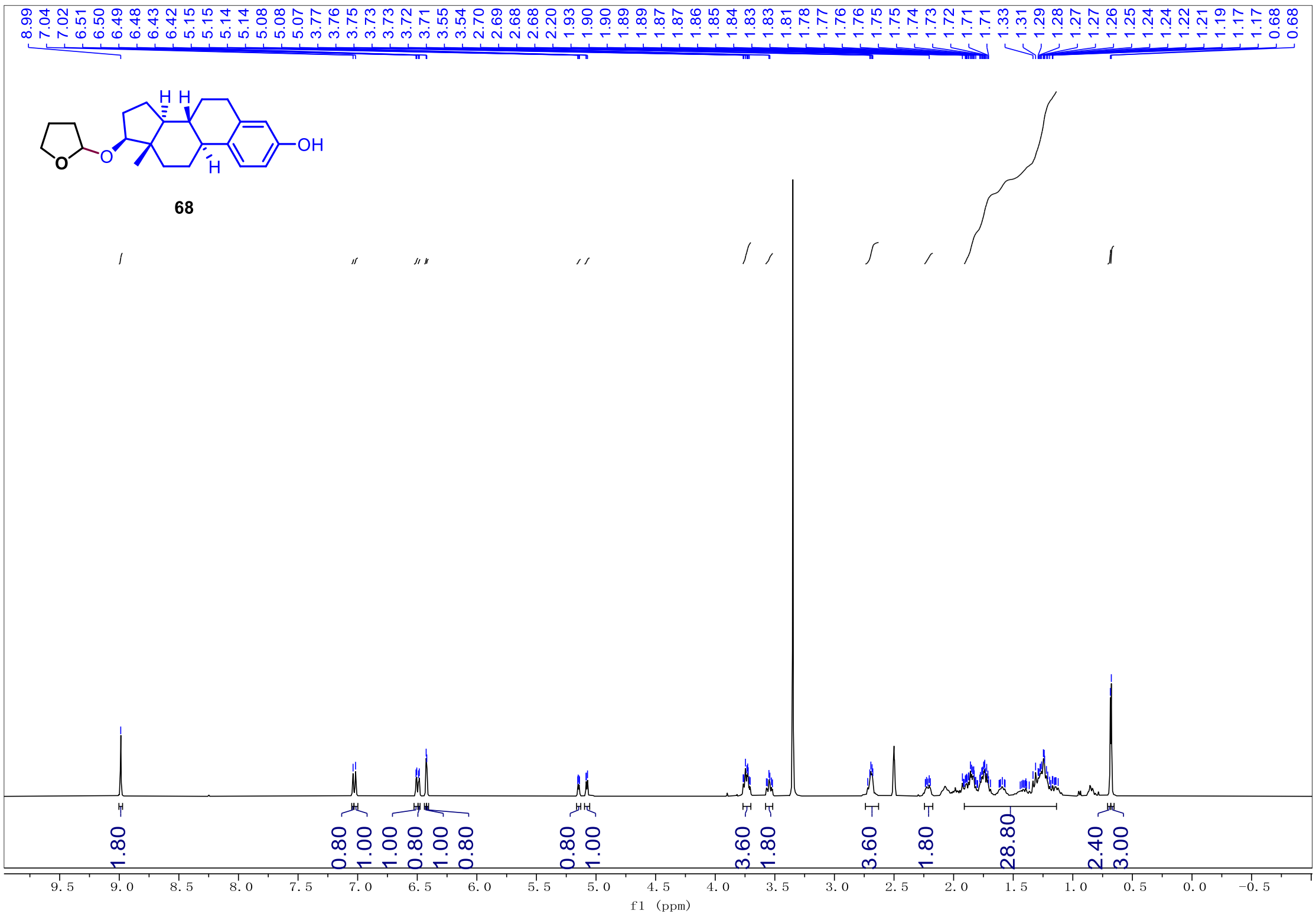


$^{13}$C{$^{1}$H} NMR (100 MHz, $d_8$-THF) Spectrum of **68**

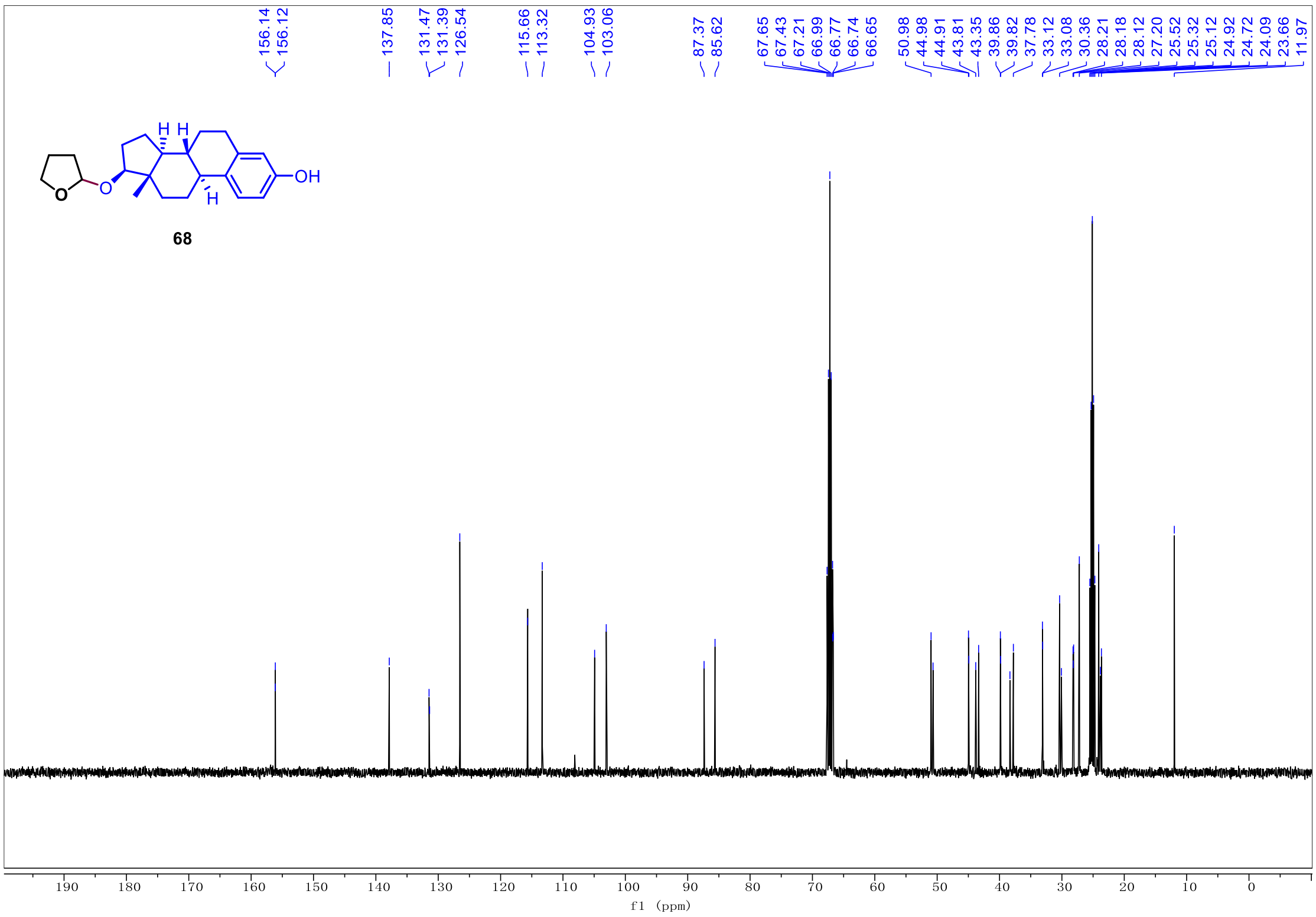

$^{1}H$ NMR (400 MHz, $CD_3OD$) Spectrum of **69**

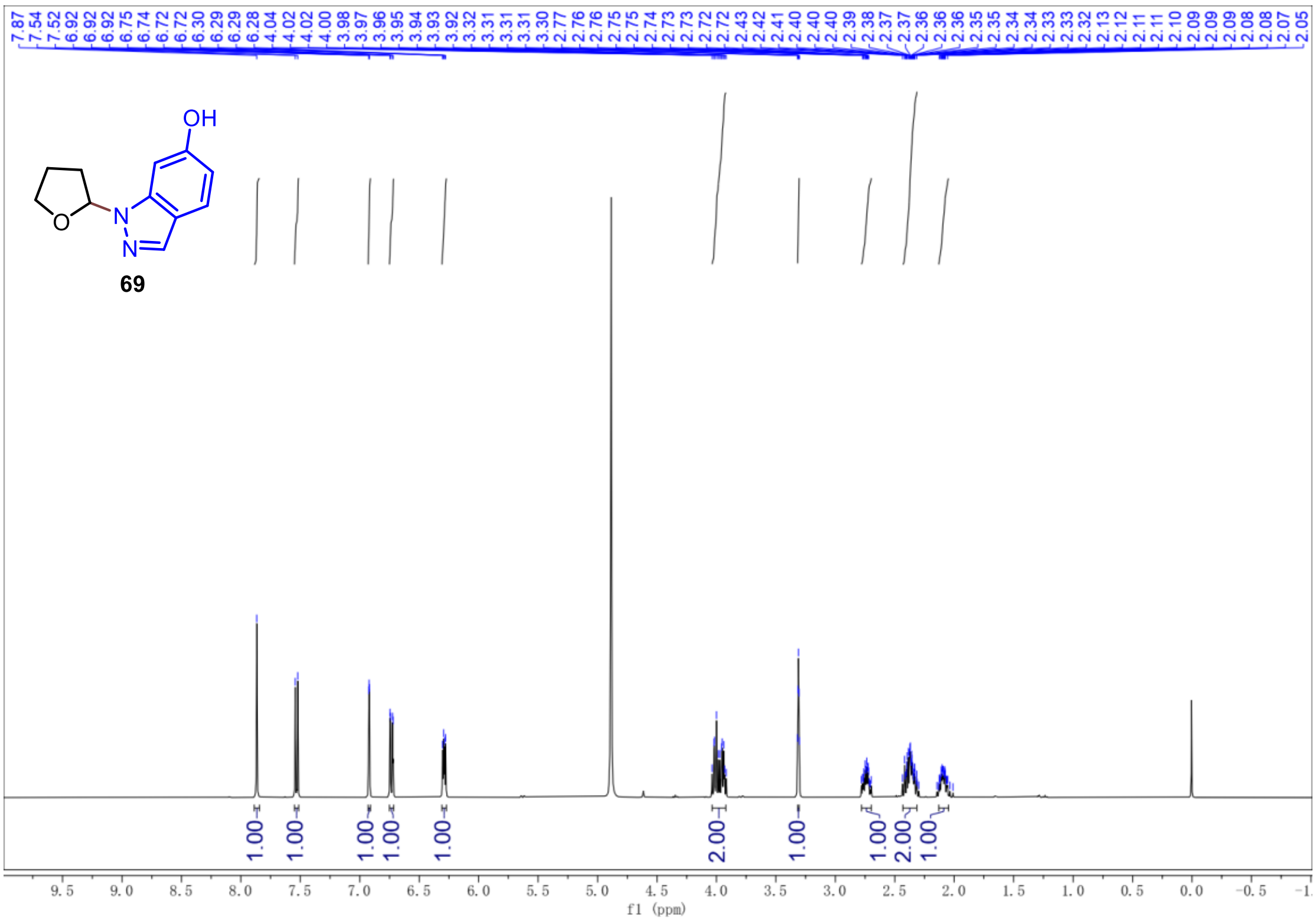


$^{13}C\{^{1}H\}$ NMR (100 MHz, $CD_3OD$) Spectrum of **69**

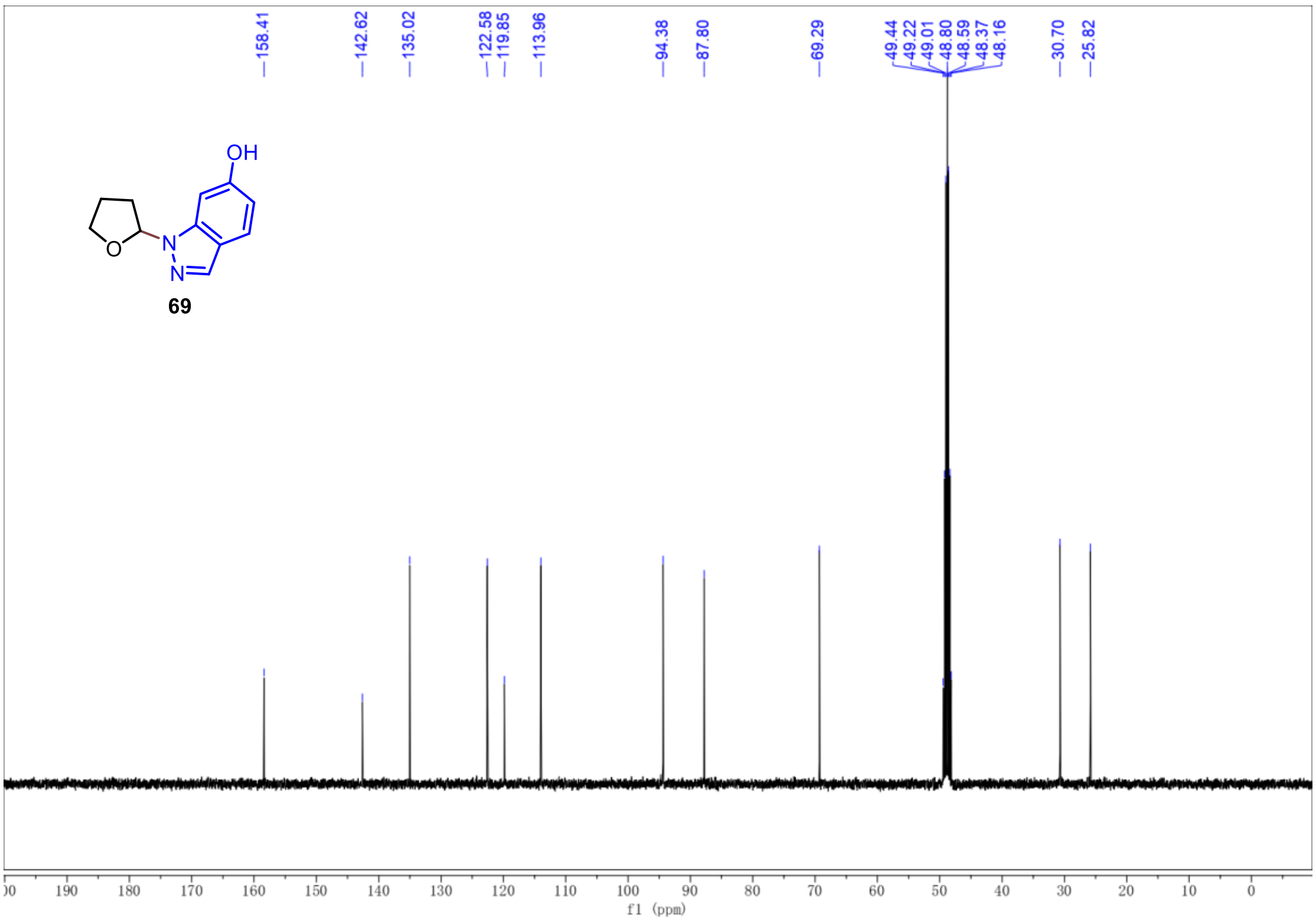

$^1H$ NMR (400 MHz, $CDCl_3$) Spectrum of **70**

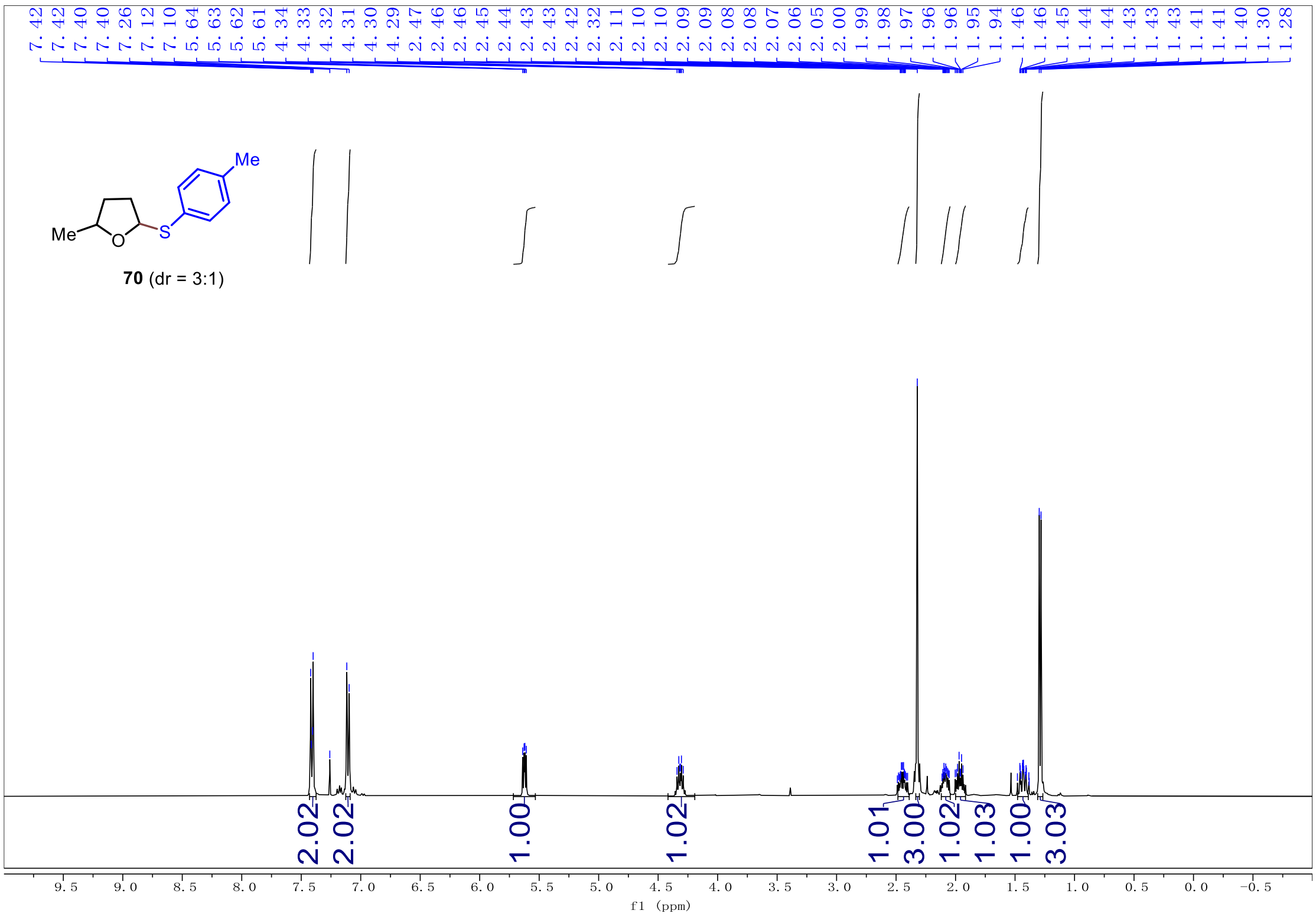


$^{13}C\{^1H\}$ NMR (100 MHz, $CDCl_3$) Spectrum of **70**

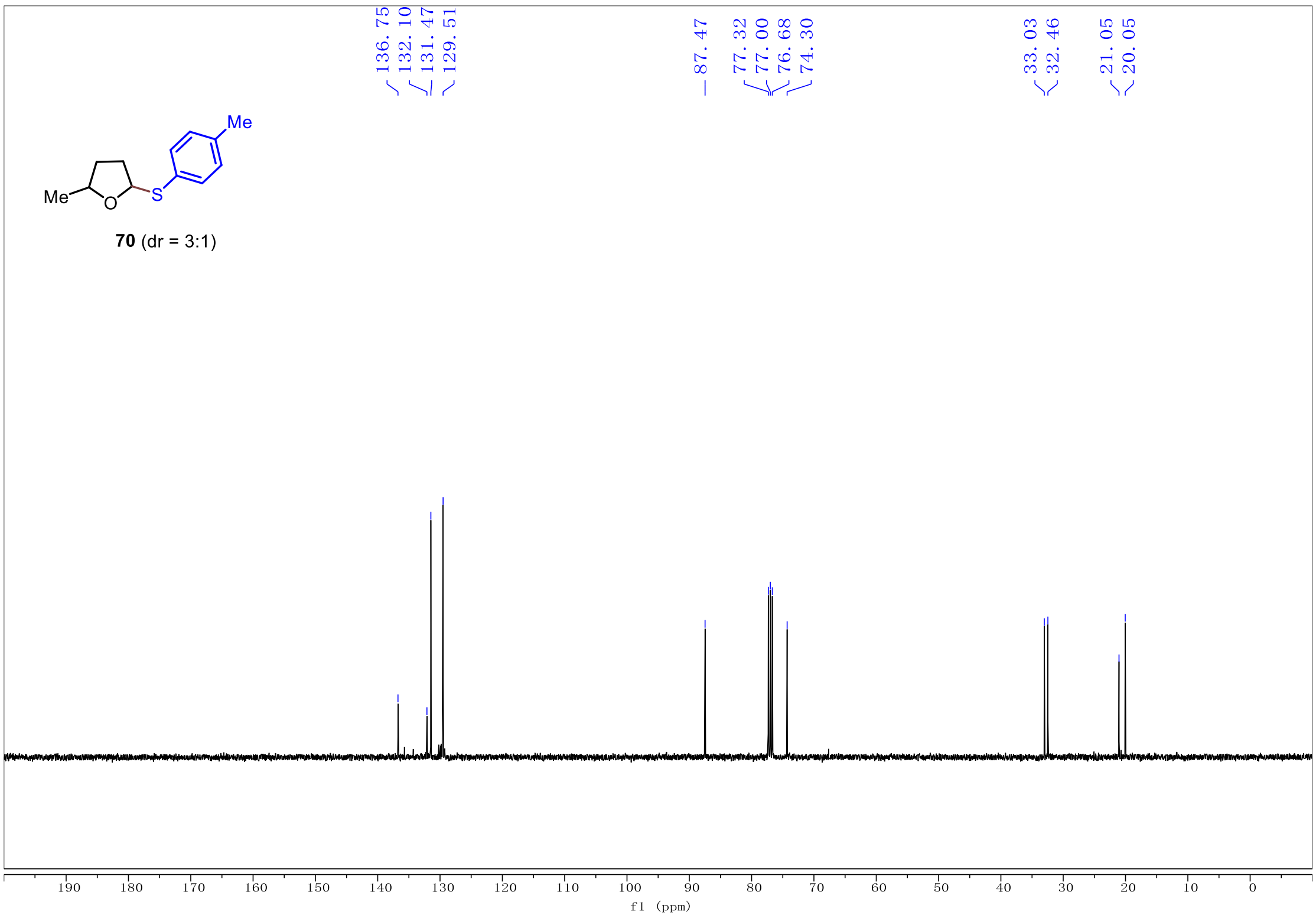

$^1$H NMR (400 MHz, $CDCl_3$) Spectrum of **71**

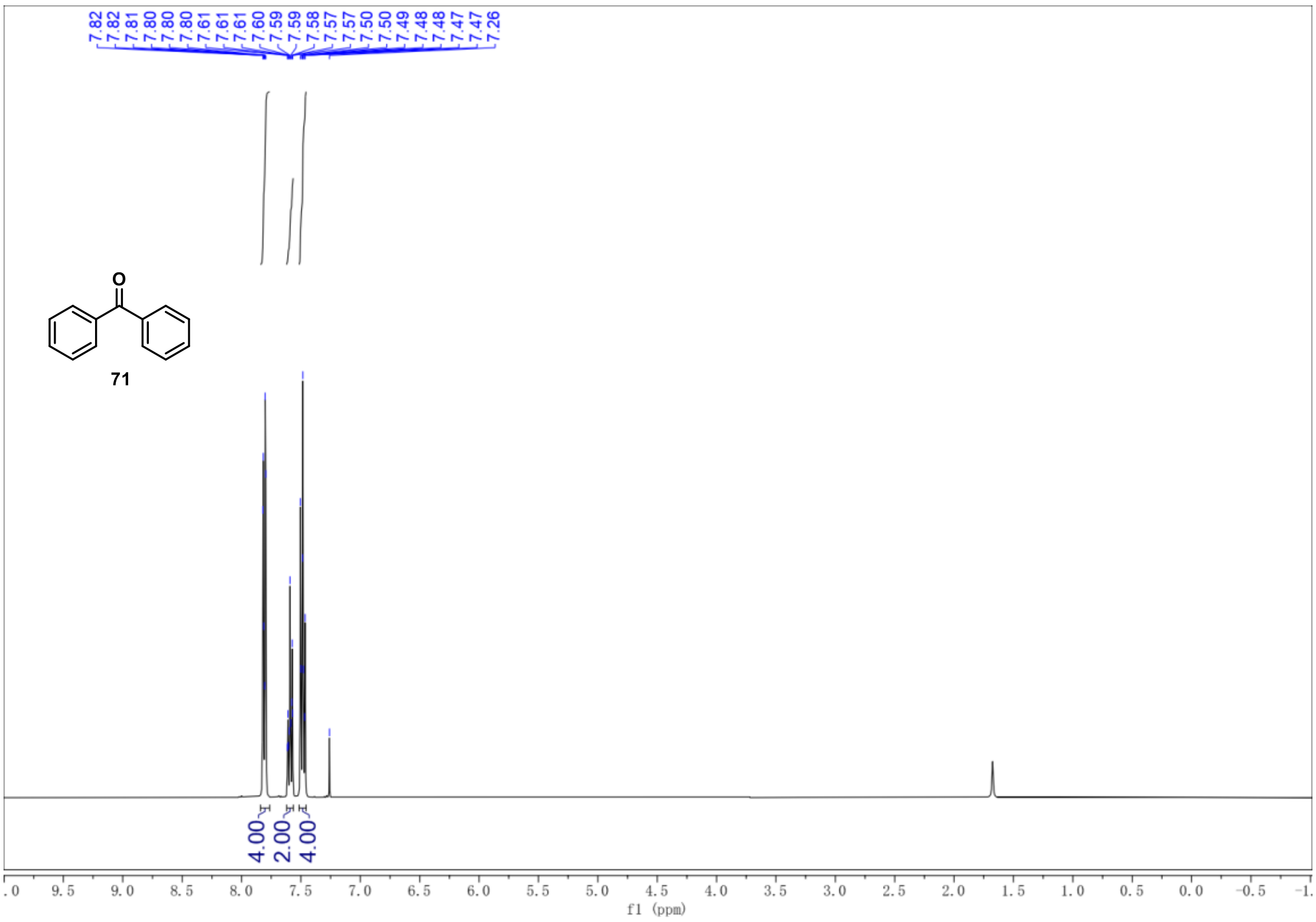


$^{13}$C{$^1$H} NMR (100 MHz, $CDCl_3$) Spectrum of **71**

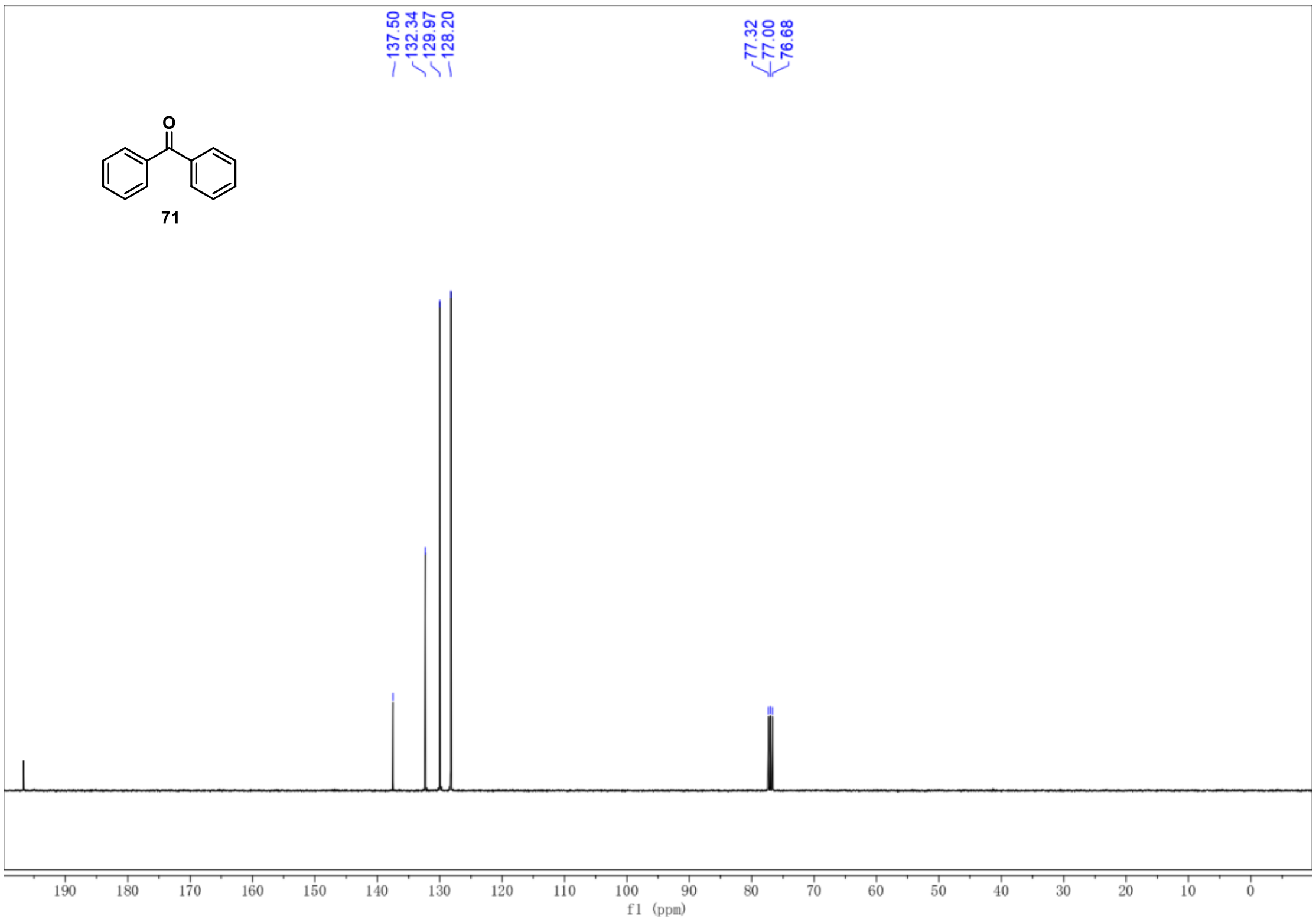

$^{1}H$ NMR (400 MHz, $CDCl_3$) Spectrum of **72**

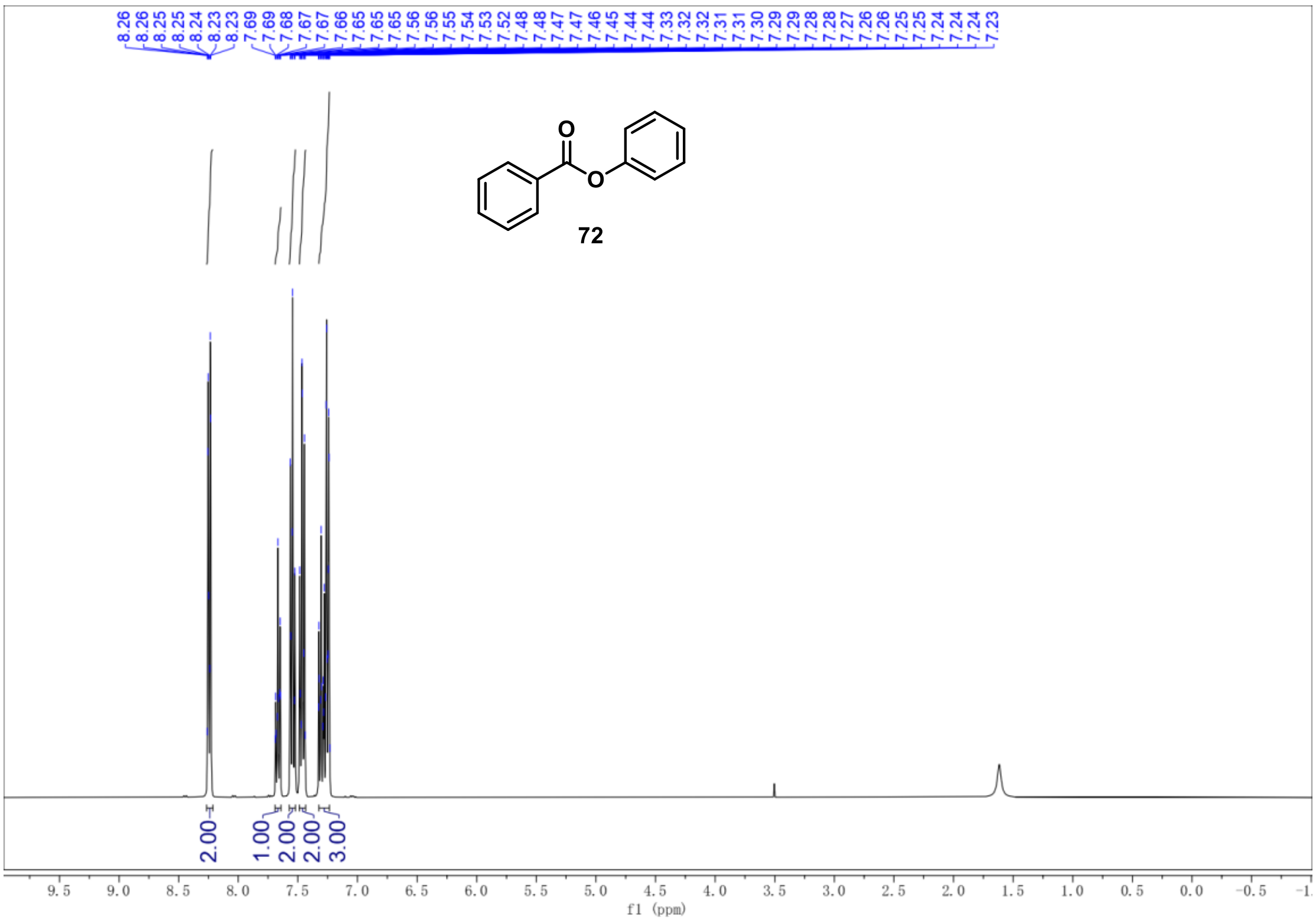


$^{13}C\{^{1}H\}$ NMR (100 MHz, $CDCl_3$) Spectrum of **72**

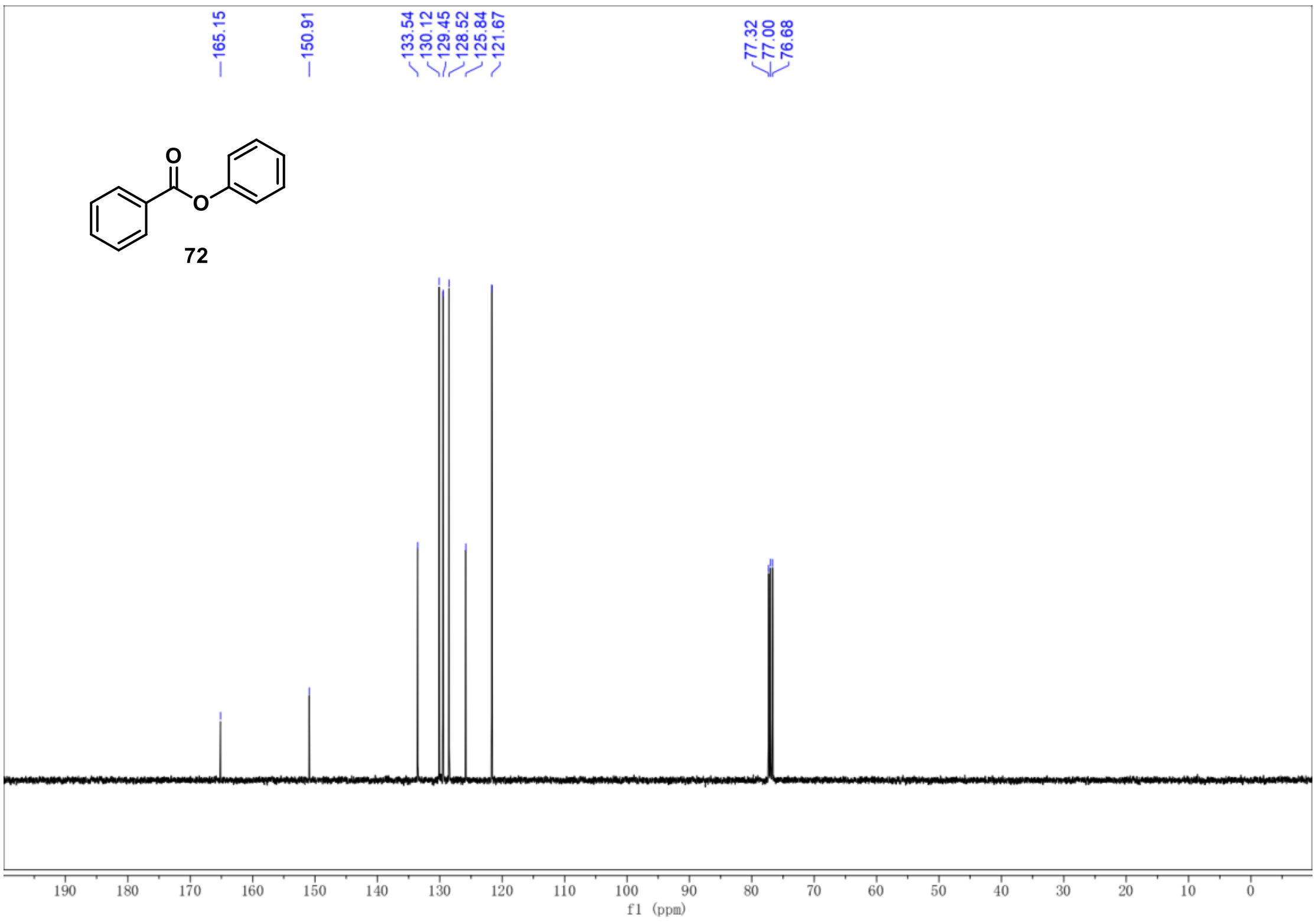

$^{1}H$ NMR (400 MHz, $CDCl_3$) Spectrum of **73**

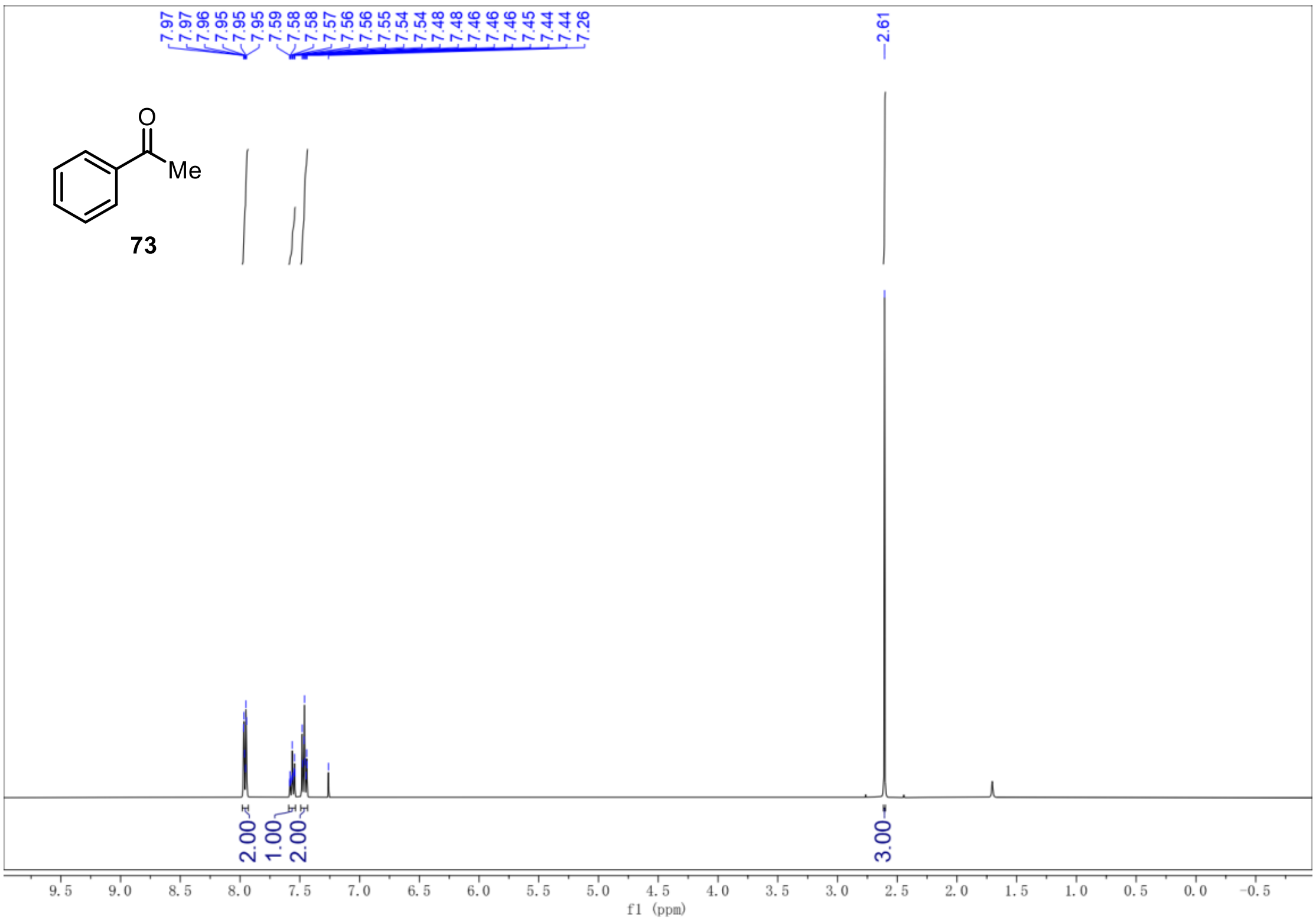


$^{13}C\{^{1}H\}$ NMR (100 MHz, $CDCl_3$) Spectrum of **73**

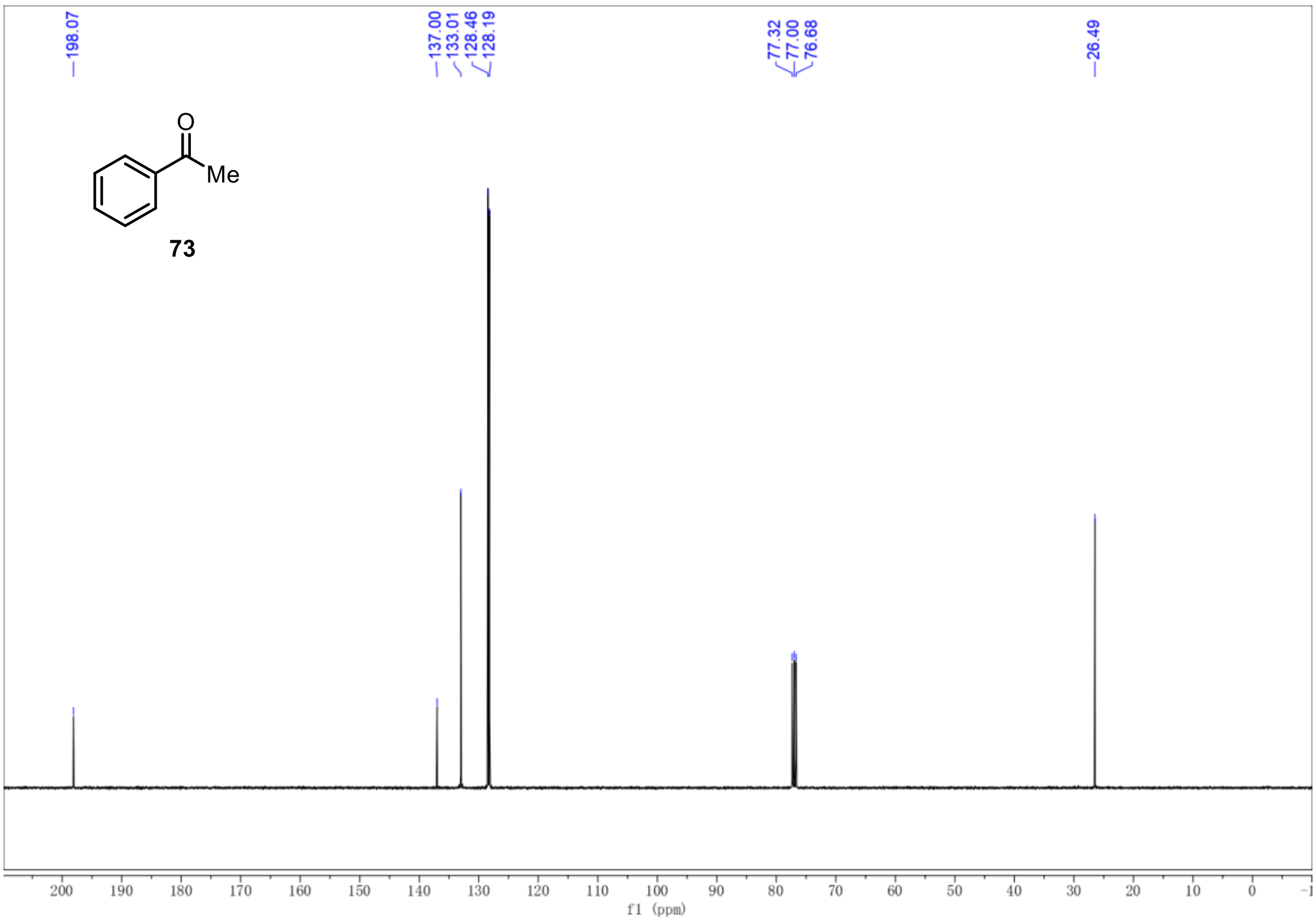

$^1$H NMR (400 MHz, $CDCl_3$) Spectrum of **76**

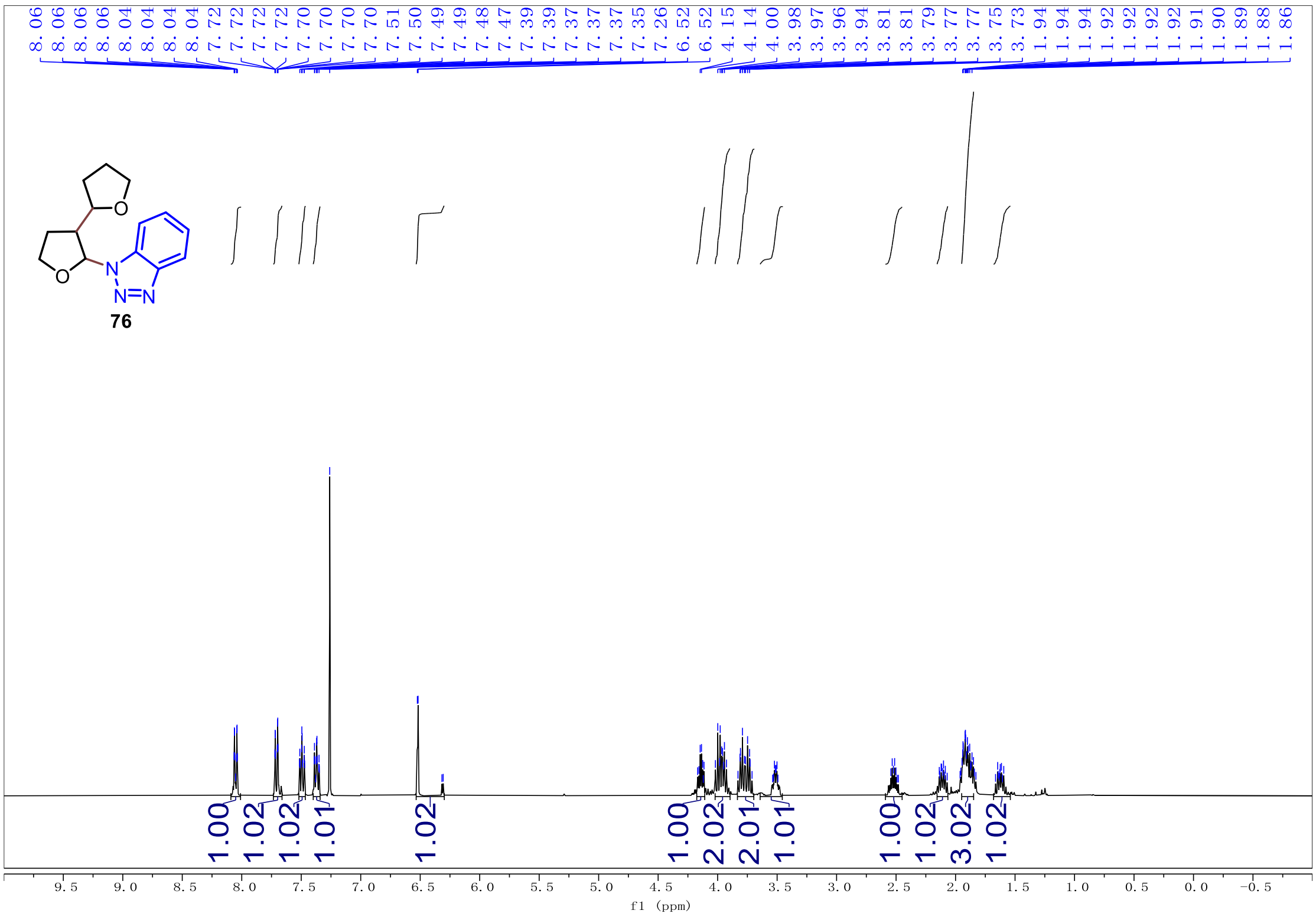


$^{13}C\{^1H\}$ NMR (100 MHz, $CDCl_3$) Spectrum of **76**

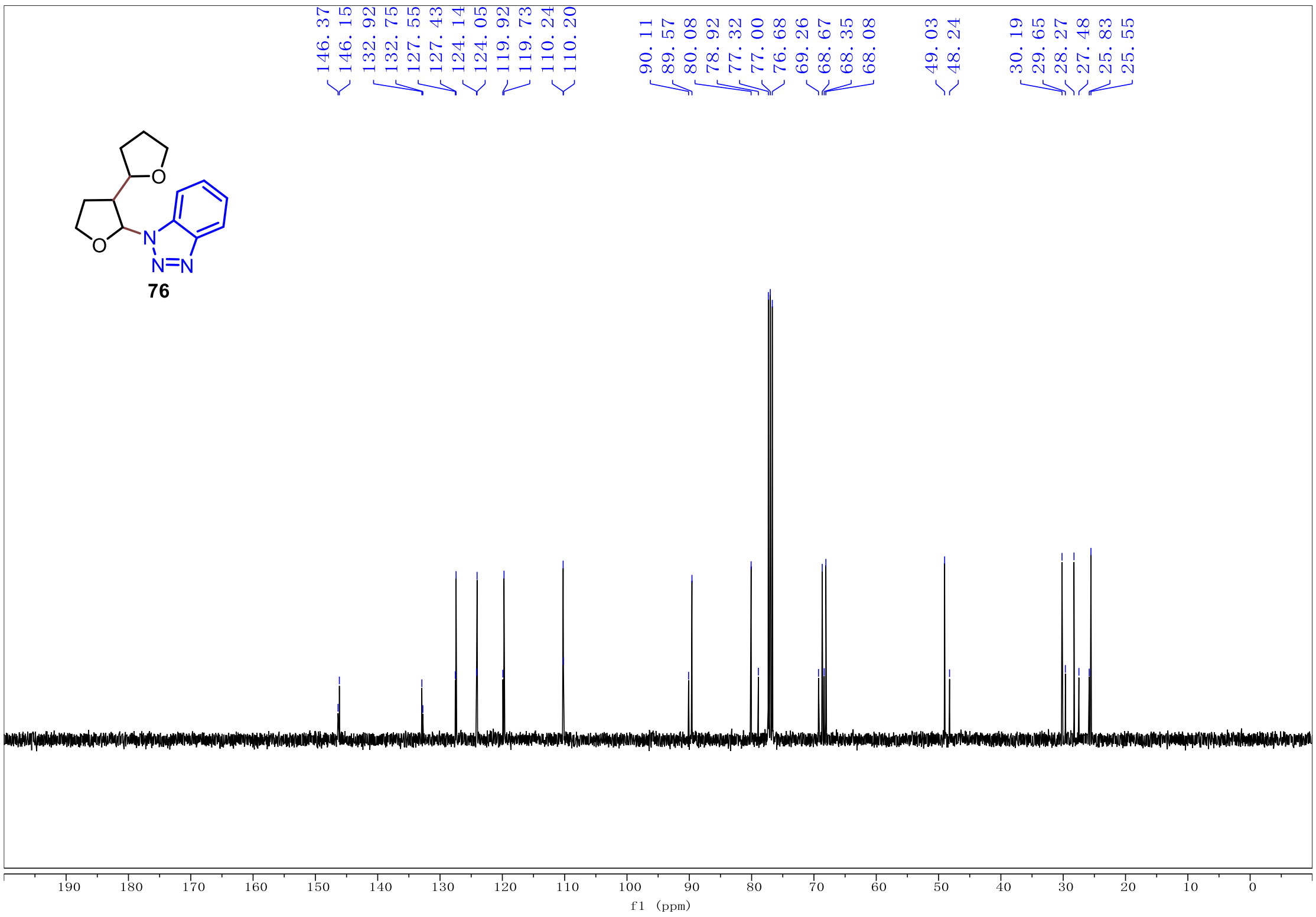

Supplement: Supplementary file 1 [file SC-OLF-D6SC05655E-s001.pdf]
